# Synthesis, Antiproliferative Activity and Molecular Docking Studies of Novel Doubly Modified Colchicine Amides and Sulfonamides as Anticancer Agents

**DOI:** 10.3390/molecules25081789

**Published:** 2020-04-14

**Authors:** Julia Krzywik, Witold Mozga, Maral Aminpour, Jan Janczak, Ewa Maj, Joanna Wietrzyk, Jack A. Tuszyński, Adam Huczyński

**Affiliations:** 1 Department of Medical Chemistry, Faculty of Chemistry, Adam Mickiewicz University, Uniwersytetu Poznańskiego 8, 61–614 Poznań, Poland; julia.krzywik@amu.edu.pl; 2 TriMen Chemicals, Piłsudskiego 141, 92–318 Łódź, Poland; mozga@trimen.pl; 3 Department of Oncology, University of Alberta, Edmonton, AB T6G 1Z2, Canada; aminpour@ualberta.ca (M.A.); jack.tuszynski@gmail.com (J.A.T.); 4 Institute of Low Temperature and Structure Research, Polish Academy of Sciences, PO Box 1410, 50–950 Wrocław, Poland; j.janczak@intibs.pl; 5 Hirszfeld Institute of Immunology and Experimental Therapy, Polish Academy of Sciences, Rudolfa Weigla 12, 53–114 Wrocław, Poland; ewa.maj@hirszfeld.pl (E.M.); joanna.wietrzyk@hirszfeld.pl (J.W.); 6 DIMEAS, Politecnico di Torino, Corso Duca degli Abruzzi, 24, 10129 Torino, Italy

**Keywords:** anticancer agents, colchicine amide, colchicine sulfonamide, tubulin inhibitors, docking studies, crystal structure

## Abstract

Colchicine is a well-known compound with strong antiproliferative activity that has had limited use in chemotherapy because of its toxicity. In order to create more potent anticancer agents, a series of novel colchicine derivatives have been obtained by simultaneous modification at C7 (amides and sulfonamides) and at C10 (methylamino group) positions and characterized by spectroscopic methods. All the synthesized compounds have been tested in vitro to evaluate their cytotoxicity toward A549, MCF-7, LoVo, LoVo/DX and BALB/3T3 cell lines. Additionally, the activity of the studied compounds was investigated using computational methods involving molecular docking of the colchicine derivatives to β-tubulin. The majority of the obtained derivatives exhibited higher cytotoxicity than colchicine, doxorubicin or cisplatin against tested cancer cell lines. Furthermore, molecular modeling studies of the obtained compounds revealed their possible binding modes into the colchicine binding site of tubulin.

## 1. Introduction

**Scheme 1:**
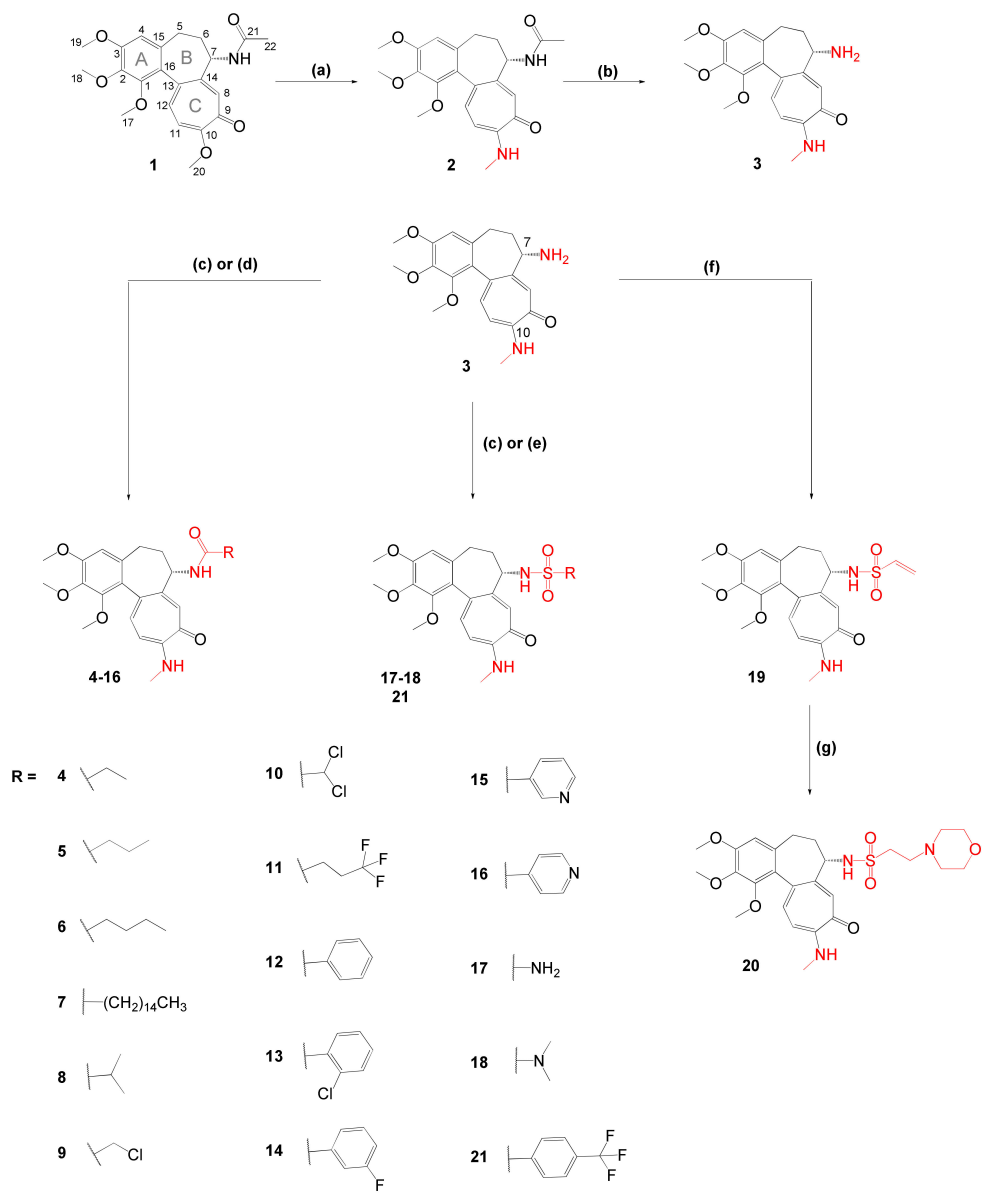
Synthesis of doubly modified colchicine derivatives (**2**–**21),** changes at C7 and C10 positions are highlighted in red. Reagents and conditions: (**a**) NHCH_3_/EtOH, reflux; (**b**) 2M HCl, reflux; (**c**) RC(O)Cl or RSO_2_Cl, Et_3_N, DCM, 0 °C to RT for **4**, **8**, **13**, **15**–**16, 18** and **21**; (**d**) RCOOH, EDCI, DCM, RT for **5**–**7, 9**–**12** and **14**; (**e**) (1) *N*-(*tert*-butoxycarbonyl)-*N*-[4-(dimethylazaniumylidene)-1,4-dihydropyridin-1-ylsulfonyl]azanide, DCM, RT, (2) 4M HCl/EtOAc, RT for **17**; (**f**) ClCH_2_CH_2_SO_2_Cl, Et_3_N, DCM, 0 °C to RT; (**g**) morpholine, DCM, RT.

Microtubules, which are composed of α- and β-tubulin heterodimers, are involved in a large number of processes, such as intracellular transport, cell shape development, cell division and cell motility. During cell division microtubules form the mitotic spindle that in normal cells correctly separates the chromosomes into two daughter cells. In cancer cells the rate of mitosis is typically increased but chromosome segregation is imperfect leading to aneuploidy. Microtubules formed during mitosis are considered as an ideal target for anticancer drugs since no cell can divide without the force generated by microtubules. Therefore, many inhibitors of microtubule dynamics have been investigated for their potential use as cancer chemotherapy drugs [1,2,3,4,5,6,7]. One of these compounds is colchicine **1** (see Scheme 1), a well–known tropolone alkaloid isolated from *Colchicum autumnale*, which has been shown to exhibit very high cytotoxic effects. It binds to tubulin at the colchicine binding site and induces conformational change in the tubulin dimer making it incompetent for microtubule assembly. As a result, the cell cycle is blocked and apoptosis is induced [8,9,10,11,12,13,14]. Unfortunately, colchicine is too toxic to be useful as an antitumor agent [8,15,16,17,18,19,20,21]. Nevertheless, it has found use in therapy, e.g., for the treatment of familial Mediterranean fever, Behcet’s disease or acute gout [22,23,24,25,26,27].

Therefore, over the past few decades much interest has been focused on structural modifications of **1** in the hope of improving its therapeutic index [28,29,30,31]. Numerous double-modified colchicine derivatives have been synthesized with the group at position C7 substituted by various amide and sulfonamide moieties as well as with replacement of the methoxy group at position C10 by a group containing either a nitrogen or a sulfur atom and their biological activities have been determined [28,29,32,33,34,35,36]. These results have shown that new derivatives may have lower toxicity with respect to normal cells while maintaining high antitumor activity. In addition, from the chemical point of view, such a change at position C10 allows obtaining compounds more resistant to acid hydrolysis [29,30,33]. We have decided to check various amide and sulfonamide moieties because they have long been valued for their rich biological and chemical profiles and have emerged as a promising class of compounds in drug discovery [37,38,39,40,41].

Herein, we report the synthesis, crystallographic and spectroscopic analysis of a series of structurally different derivatives of colchicine obtained by its modification at position C7 (various amide, sulfonamide or sulfamide moieties) and at position C10 (methylamino group). We also describe the results of in vitro antiproliferative activity evaluation of colchicine (**1**) and the obtained colchicine derivatives (**2**–**21**) against four human cancer cell lines and one normal murine embryonic fibroblast cell line. To acquire more knowledge about the molecular mechanism of action of the investigated compounds (**1**–**21**), we also present results of *in silico* molecular docking study of the colchicine binding site (CBS) of β-tubulin.

## 2. Results and Discussion

### 2.1. Chemistry

To investigate the effect of methylamino group at position C10 and, at the same time, various amide, sulfonamide and sulfamide moieties at position C7 of colchicine **1** on its antiproliferative activity, eighteen new derivatives (**4**–**21**) were synthesized. To facilitate the structure-activity relationship analysis (SAR) we designed compounds with different side chains at position C7: alkyl chains of various length, straight and branched (**4**–**8**), unsaturated alkyl chain (**19**), alkyl chains of various lengths containing halogen atoms (**9**–**11**), an aromatic group without or with substituents (**12**–**16**, **21**), and compounds containing an amino group **17**–**18** and **20**.

The general route for the synthesis of colchicine derivatives **2**–**21** is depicted in Scheme 1. Colchicine (**1**) was treated with methylamine solution in ethanol to give 10-methylamino-10-demethoxycolchicine (**2**) with 80% yield, according to the method described earlier [42]. The replacement of water solution of methylamine by ethanol solution eliminated the work up after the reaction and permitted obtaining comparable final yields. Next, hydrolysis of **2** with 2M HCl yielded *N*-deacetyl-10-methylamino-10-demethoxycolchicine (**3**). Compounds **4**–**16**, **18**–**19** and **21** were readily available from **3** by treatment with respective acid/ sulfonamide/ sulfamide chloride in the presence of triethylamine or with the corresponding carboxylic acid and carbodiimide as a condensing agent. Compound **17** was prepared using a *N*-(*tert*-butoxycarbonyl)-*N*-[4-(dimethylazaniumylidene)-1,4-dihydropyridin-1-ylsulfonyl]azanide and further modified by removal of the *tert*-butoxycarbonyl group from amine with HCl [43]. Vinylsulfonamide **19** was synthesized from 2-chloroethanesulfonyl chloride and **3** through sulfonylation and in situ β-elimination of HCl. Compound **19** was used as the Michael acceptor with an electron-deficient double bond for the reaction with morpholine to produce compound **20 [44,45]**. All synthesized compounds were isolated in pure form after column chromatography.

The purity and structures of the obtained compounds **2**–**21** were determined using the LC-MS, ^1^H and ^13^C NMR methods and are shown in the Supplementary Materials and discussed below. The characteristic signals of -OCH_3_ group at position C10 of **1** in the ^1^H NMR and ^13^C NMR spectra were observed as a singlet at 4.0 ppm and at 56.5 ppm, respectively. These signals vanish after the reaction of colchicine with methylamine proving the substitution of the -OCH_3_ group in the tropolone ring of **1**. After the introduction of -NHCH_3_ at position C10 the signals of this group in the obtained derivatives (**2**–**21**) were visible approx. at 3.1 ppm as a doublet and approx. at 7.4 ppm as a quartet in ^1^H NMR and approx. at 29.5 ppm in ^13^C NMR. The chemical shifts of the amide moiety can be found in the range 7.9–9.7 ppm in ^1^H NMR and in the range 164.2–175.2 ppm in ^13^C NMR, depending on the substituent used. The ESI mass spectrometry confirmed the structure of the obtained compounds by the presence of an *m*/*z* signals assigned to the corresponding pseudomolecular ions of these compounds.

### 2.2. X-ray Crystal Analysis

Structural characterization of the colchicine derivatives is very important in order to understand their anticancer properties stemming from their interaction with tubulin as well as to enable structure–activity relationship analysis (SAR) and related investigation. Therefore, structural analyses of all crystals that were suitable for X-ray analysis of single crystals were performed. Crystals of **6**, **11**, **12**, **14**, **18** and **19** suitable for the X-ray single crystal analysis were obtained by recrystallization of the respective colchicine derivatives from acetonitrile, whereas crystals **15** and **16** from ethyl acetate solutions. All crystals were measured at room (295 K) and low (100 K) temperature. Details of the data collection parameters, crystallographic data and the final agreement parameters are listed in Supplementary Table S1. In the temperature range from 295 K to 100 K, no structural phase transitions were observed in the crystals studied, although for colchicine derivative **11** at low temperature some disorder of the -CF_3_ group in the -CH_2_-CH_2_-CF_3_ group at atom C21 could be observed. Colchicine derivatives **6**, **11**, **12**, **14**, **15,** and **16** crystallize in the P3_2_21 space group of the trigonal system while derivative **18** crystallizes in the P2_1_2_1_2_1_ space group of the orthorhombic system and derivative **19** crystallizes in the P2_1_ space group of the monoclinic system. These space groups are chiral since the compounds contain an asymmetric carbon (C7) atom. The absolute configuration at the C7 atom is *S* in all structures. The molecular structures of all colchicine derivatives (**6**, **11**, **12**, **14**, **15**, **16**, **18** and **19**) are illustrated in Supplementary Figure S60. The planar phenyl A and tropolone C rings in all colchicine derivatives (**6**, **11**, **12**, **14**, **15**, **16**, **18** and **19**) are twisted around the C13–C16 bond with the torsion angle describing the twisting conformation C1–C16–C13–C12 between ~53° and ~56° at 100 K and they do not differ significantly from the values at room temperature (Table 1). Ring B in all colchicine derivatives exhibits a similar puckering pattern and the extent of its non-planarity is such that it adopts a conformation, which is close to the twist-boat with a flattening caused by the fusion of rings A and C (see Supplementary Figure S60). So the conformation of the fused A, B and C rings of colchicine skeleton in the investigated derivatives is quite similar to that in colchicine itself [46].

**Figure 1:**
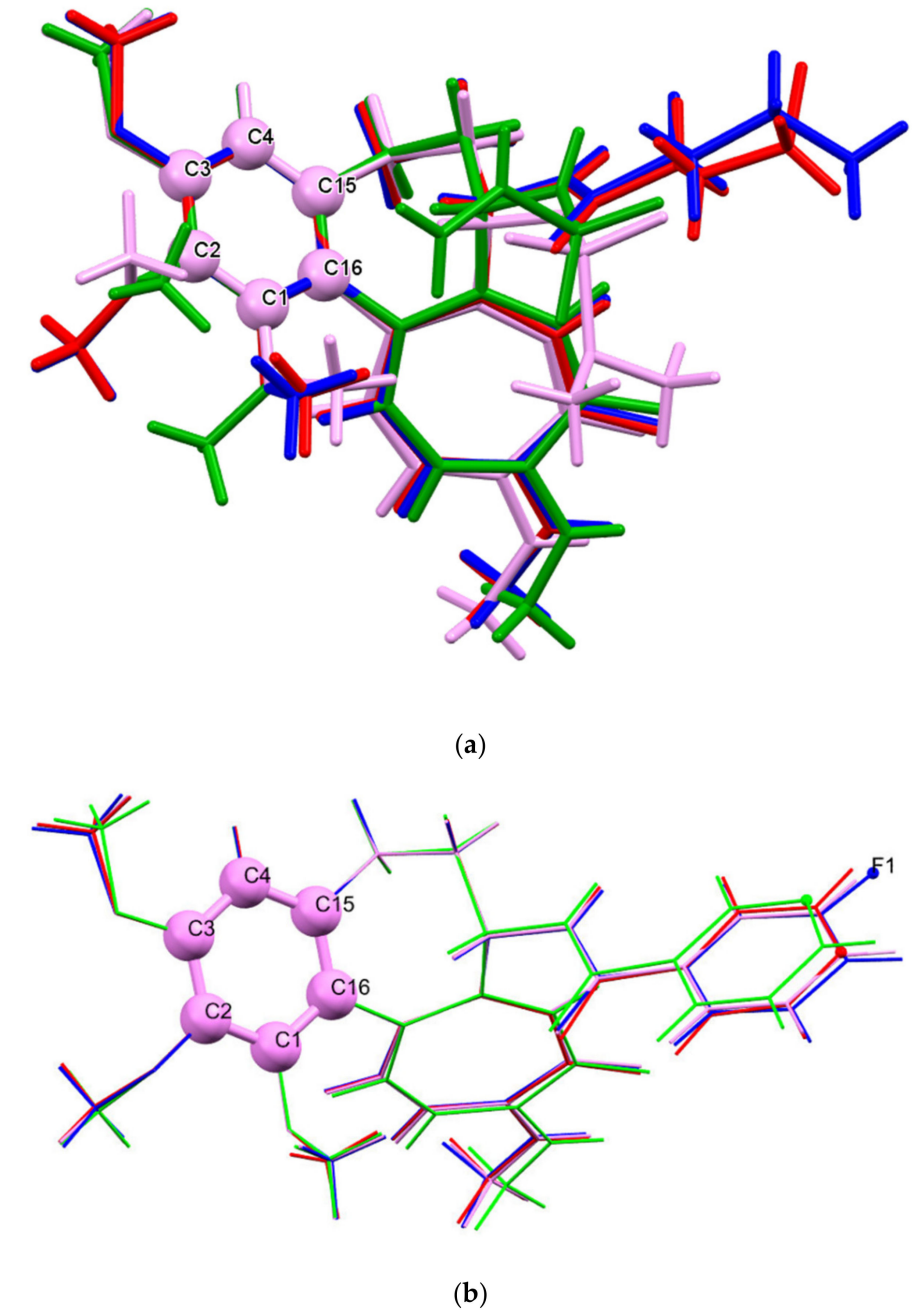
Comparison of the X-ray structure of (**a**) derivatives with a chain substituent at position C7: **6** (blue), **11** (red), **18** (pink) and **19** (green) and (**b**) derivatives with a substituent containing an aromatic ring at position C7: **12** (pink), **14** (blue), **15** (green) and **16** (red) showing the conformation of the colchicine skeleton. The molecules are overlapped one over another so that the ring C1-C2-C3-C4-C15-C16 is at the same position.

The methoxy group -OCH_3_ linked to the phenyl ring at C3 is almost coplanar with the ring in all structures of colchicine derivatives, whereas the other two methoxy groups linked at C1 and C2 atoms of the phenyl ring have different orientations in some colchicine derivatives (Table 1). The *N*-methylamino group (-NHCH_3_) linked to tropolone C ring at C10 atom is almost coplanar with the ring. The differences between the conformations of the investigated colchicine derivatives are illustrated in Figure 1 and Table 1. For clarity, the colchicine derivatives have been divided into two groups, according to the substituent in ring B at position C7; one group comprises the derivatives with a chain substituent (Figure 1a) and the other group comprises the derivatives with a substituent containing an aromatic ring (Figure 1b). Analysis of these results reveals a significant difference in the torsion angle C17-O1-C1-C2 in compounds 18 (~−109°) and 19 (~66°) or the difference between the calculated (~−103°) and measured (~−77°) torsion angle C18-O2 -C2-C3 in compound 19. These differences result from different approach to the description of the molecule conformations, the X-ray analysis values refer to the conformation of molecules in crystals, in which the intermolecular interactions play a significant role and leads crystallization and specific crystal packing, while the DFT values refer to a single isolated molecule in the gas state with the intermolecular interactions not taken into account.

### 2.3. Molecular Electrostatic Potential Map Analysis

The role of the trimethoxyphenyl ring A of colchicine as well as that of the tropolone ring C in tubulin binding have been studied in great details. The tropolonoic ring C of the colchicine skeleton is found to be crucial for the interaction with tubulin [47,48]. The molecular electrostatic potential map (MESP) correlated with the electronic density in a molecule and is a powerful tool for analyzing interactions [49,50,51]. It was, therefore, calculated for all structurally characterized colchicine derivatives. Additionally, the gas-phase structures of all molecules were determined using the DFT optimization with the Gaussian09 program package [52]. All calculations were carried out by the DFT method using the Becke3-Lee–Yang–Parr correlation functional (B3LYP) [53,54,55,56] with the 6–31 + G basis set, starting from the X-ray geometry of molecules. The gas-phase optimized conformations of all colchicine derivatives are, in general, in good agreement with those obtained from the X-ray single crystal investigation, however the optimized torsion angle C18–O2–C2–C3 describing the orientation of the methoxy group is significantly smaller than that provided by the X-ray analysis (Table 1 and for more details see Supplementary Table S2).

**Figure 2:**
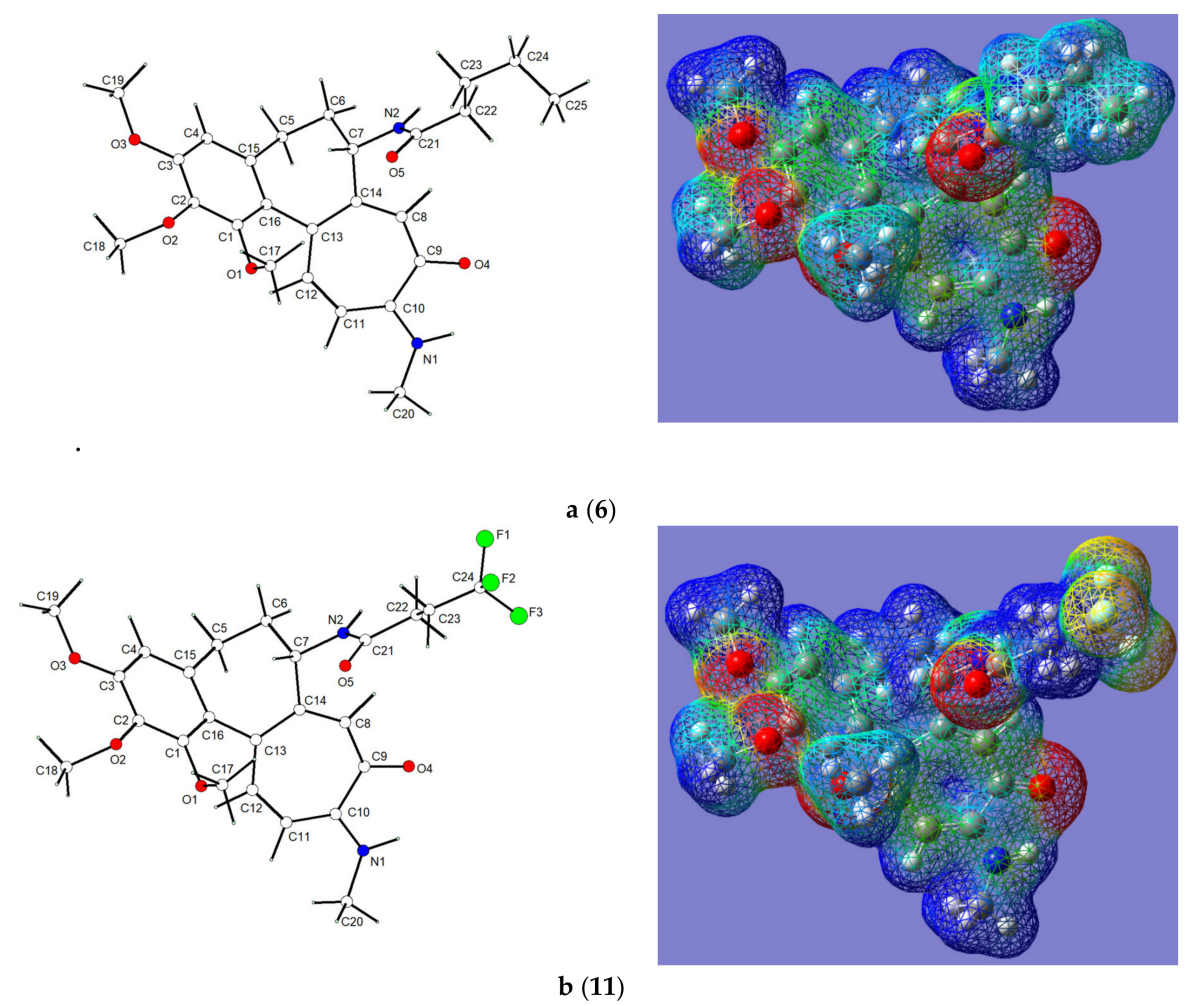

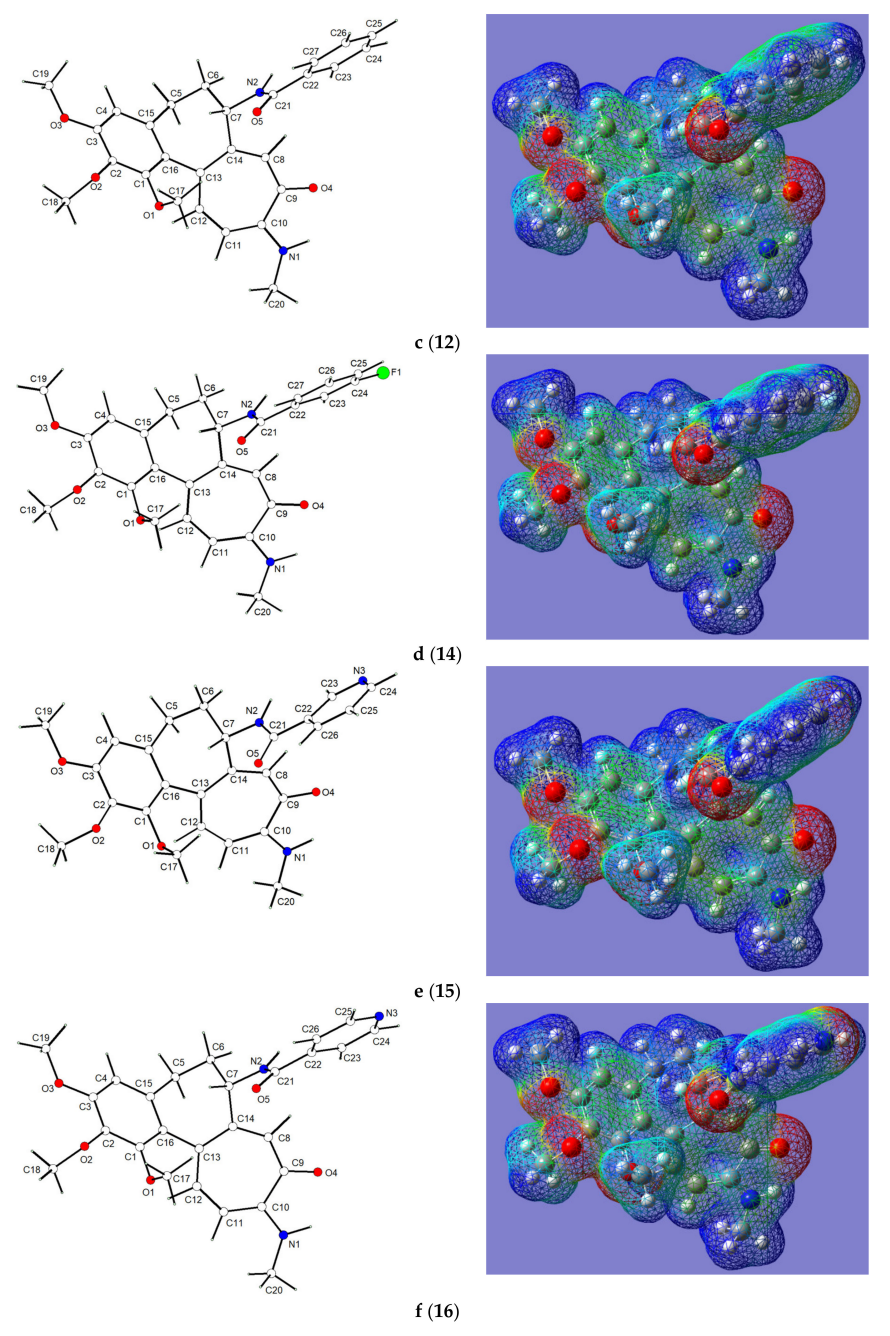

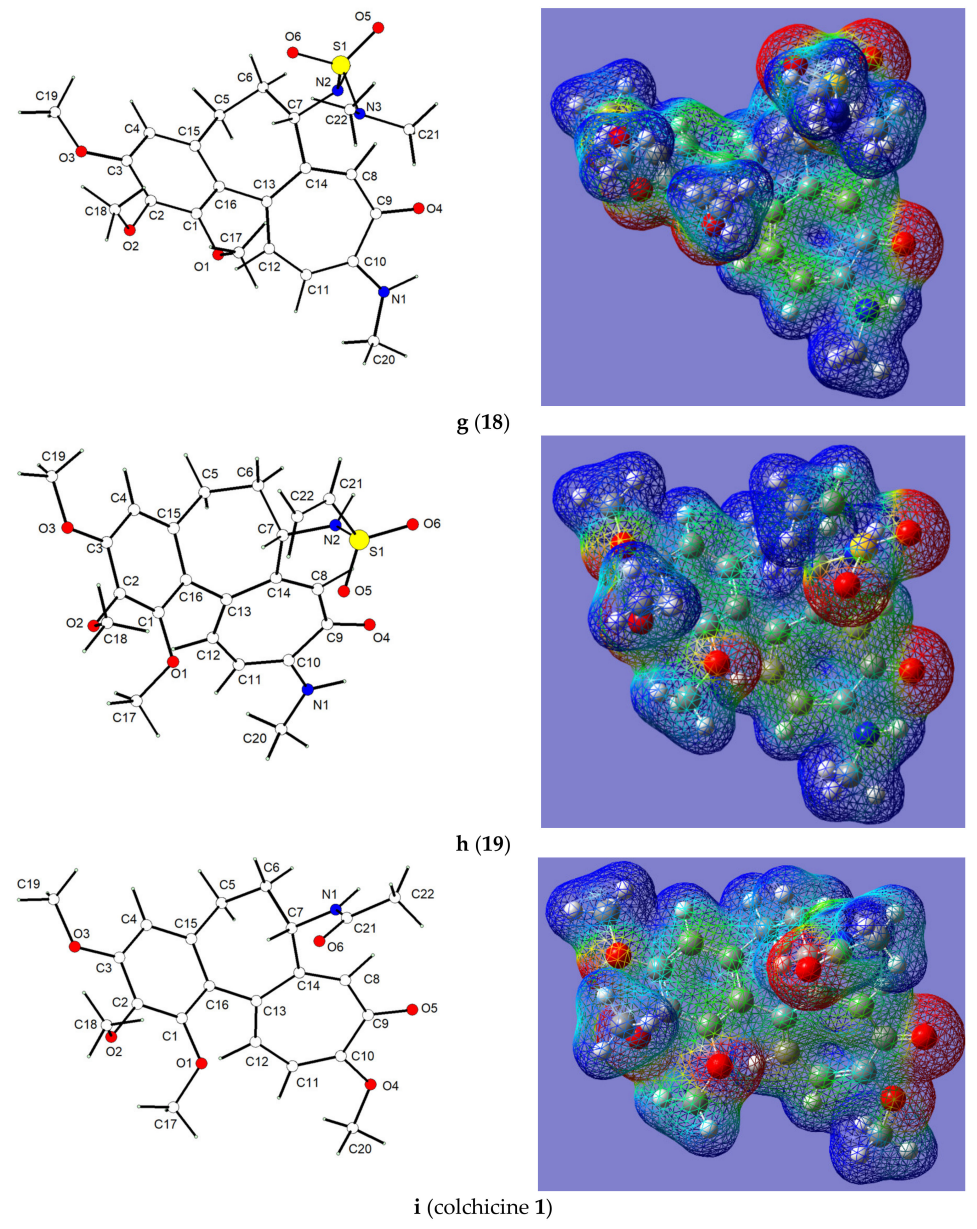
Optimized conformation (left) and three-dimensional molecular electrostatic potential map (right) for colchicine derivatives (**a**–**h**) and colchicine itself (**i**), for comparison. Color code: −0.05 eÅ^−1^ (red) to +0.05 eÅ^−1^ (blue).

The region of tubulin that interacts with colchicine is near the αβ-tubulin/dimer interface [57]. In order to better understand the interaction of the colchicine derivatives with tubulin, the molecular electrostatic potential was calculated for all structurally characterized colchicine derivatives as well as, for comparison, for colchicine itself. The three-dimensional MESP maps for colchicine derivatives and for colchicine itself were calculated on the basis of the DFT (B3LYP) optimized geometries of molecules and mapped onto the total electron density isosurface (0.008 eÅ^−3^) for both molecules using the GaussView 5.0 program (Figure 2). The color coding of MESP is in the range of −0.05 (red) to 0.05 eÅ^-1^ (blue). For all colchicine derivatives, the regions of negative MESP are usually associated with the lone pair of electronegative atoms (O and N), whereas the regions of positive MESP are associated with the electropositive atoms (Figure 2).

The nucleophilic regions in colchicine derivatives are observed near oxygen atoms of all methoxy and carbonyl groups. In addition, significantly less negative value of MESP than that near the oxygen atoms spreads across the aromatic phenyl rings. The planar conformation of tropolone ring C, showing the alternating single and double C-C bonds, is manifested as partial delocalization of the π electrons resulting in a slightly negative value of MESP on both sides of the planar fragment of colchicine derivatives. The molecular electrostatic potential for colchicine itself was also calculated for comparison (Figure 2i). In all colchicine derivatives the methoxy (-OCH_3_) substituent in the tropolone C ring is replaced by *N*-methylamino substituent (-NHCH_3_), therefore, MESP maps show a less negative area near this group (Figure 2a–h) compared to the MESP map of colchicine itself (Figure 2i). Additionally, the replacement in colchicine molecule of the -NHC(O)CH_3_ group in ring B at C7 atom with various substituents modified the size of the molecule and the maps of electrostatic potential. This is particularly visible for derivatives **11**, **14** and **18**, **19** in which the -NHC(O)CH_3_ group is replaced by -NHC(O)C_2_H_4_CF_3_, -NHC(O)C_6_H_4_F in **11** and **14**, respectively, and in **18** and **19** by the substituents containing sulfonyl group (-SO_2_R).

The DFT results, especially the three-dimensional molecular electrostatic potential map (MESP) providing information on the distribution of the electron density of molecules are useful for predictions of interactions between the tested compounds and homology modeled tubulin βI. The formation of guest-host complexes (colchicine derivatives as guest and tubulin as host) depends on the guest’s fit into the host cavity and their interactions that result from the mutual matching of electrostatic interactions.

### 2.4. In Vitro Determination of Drug-Induced Inhibition of Human Cancer Cell Line Growth

The synthesized colchicine derivatives **2**–**21** and starting material **1** were evaluated for their in vitro antiproliferative effect on four human cancer cell lines, including one cell line displaying various levels of drug resistance and additionally one normal murine embryonic fibroblast cells.

The majority of new derivatives of **1** showed antiproliferative activity in the nanomolar range and were characterized by lower IC_50_ values than unmodified colchicine **1**, as well as doxorubicin and cisplatin, commonly used as antitumor agents in cancer chemotherapy (Table 2). From the set of tested compounds, the ones most active against A549 tumor cell line were **2**, **4**–**6**, **8**–**14** and **18**–**19** (IC_50_ ≤ 15 nM), against MCF-7 tumor cell line-were **2**, **9**–**17** and **19** (IC_50_ ≤ 13 nM), against LoVo tumor cell line-were **2**, **4**–**6**, **8**–**16**, **18**–**19** (IC_50_ ≤ 11 nM) of which the lowest IC_50_ values were shown by compounds **9**–**10** and **13** (IC_50_ = 0.7–1.8 nM). Moreover, compound **13** was observed to be most active towards the LoVo/DX line (IC_50_ = 9.6 nM), approx. 170 times more potent than unmodified colchicine **1** (IC_50_ = 1646.6 nM). Compound **7** from amides and compound **20** from sulfonamides showed the weakest activity (the highest IC_50_ values) against all cancer cell lines tested. The decrease in cytotoxicity of compound **7** could be related to an increase in hydrophobicity (high calculated octanol/water partition coefficient clog*P* = 8.7, see Table 3). It is well known that high clog*P* value and therefore low hydrophilicity are responsible for poor absorption and permeation to the colchicine binding pocket in β-tubulin. The high IC_50_ value for compound **20** may be due to the presence of a morpholine ring which is a large volume substituent and can adopt different conformations. Although these compounds were the least potent out of the whole series of tested derivatives (**1**–**21**), their IC_50_ values were in the micromolar range (see Table 2).

Although the synthesized compounds are effective toward cancer cells, their potential is limited against cells with developed drug resistance. The data presented in Table 2 show that unmodified colchicine **1** and all of the colchicine derivatives **2**–**21** less effectively inhibited the proliferation of the doxorubicin-resistant subline LoVo/DX than the sensitive LoVo cell line. The calculated values of RI clearly confirmed that none of the tested amides and sulfonamides was able to overcome the drug resistance of LoVo/DX cell line (RI ranges from 11.8 to 306.0). It can be explained by the upregulated expression of efflux transporters in these cells, playing an important role in drug transport in many organs and determining the drug resistance of cancer cells. Because this type of resistance is one of the mechanisms of cancer resistance [58], compounds **1**–**2** and **4**–**21** are probably good substrates for such pumps. Increased efflux of compounds makes it impossible to reach their adequate concentrations in the cell and consequently to exert efficient cytotoxic effect. The only compound which showed RI < 10 was derivative **3**, having at position C7 a free amino group. Keeping in mind its good activity (IC_50_ < 20 nM for three cancer cells, see Table 2) it can be still considered as a good starting point for the chase after antitumor agents active against drug resistant lines.

**Figure 3:**
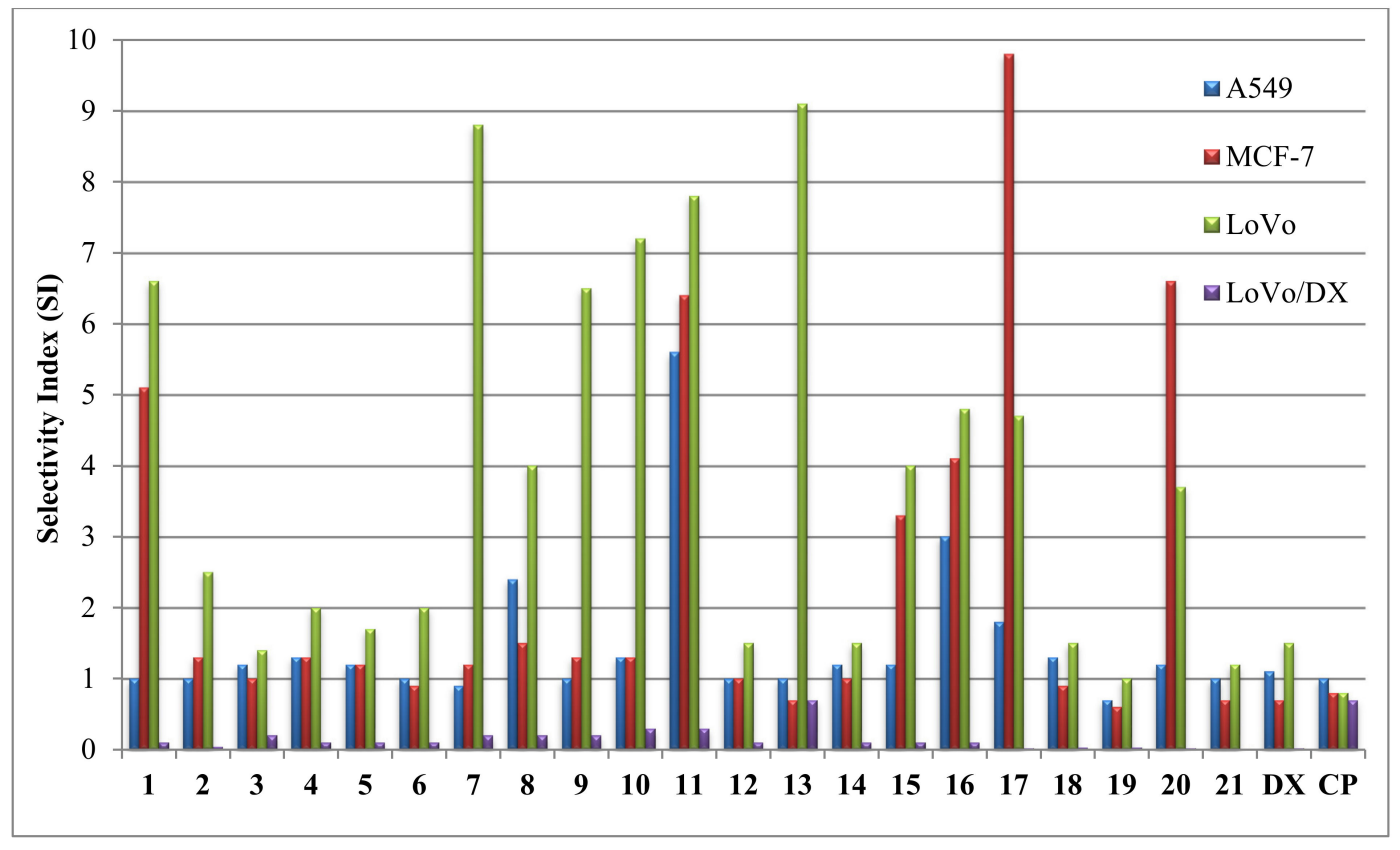
Comparison of selectivity index (SI) values of the tested compounds. The SI was calculated for each compound using the formula: SI = (IC_50_ for normal cell line BALB/3T3)/(IC_50_ for respective cancerous cell line). A favorable SI > 1.0 indicates a drug with efficacy against tumor cells greater than the toxicity against normal cells.

The selectivity index (SI) was calculated to evaluate the toxicity of the compounds studied against normal cells and to predict their therapeutic potential (see Figure 3). High SI values result from large differences between the cytotoxicity against cancer and normal cells and this means that cancer cells will be killed at a higher rate than normal ones. From the set of tested compounds with methylamino group at position C10 and alkyl chains at position C7 (**2**, **4**–**8, 19**) the best selectivity index for A549, MCF-7 and LoVo cells (SI = 1.5–4.0) showed **8** with short and branched substituent (isobutyric acid derivative). The 4,4,4-trifluorobutyric acid derivative **11** (from compounds with alkyl chains of various lengths containing halogen atoms **9**–**11**) showed outstanding selectivity for three out of four cancer cells (SI = 5.6–7.8). It should be emphasized that compound **11** was the only compound with SI values greater than that of unmodified colchicine **1** for all tumor cell lines. Despite the fact that compounds **9**, **10,** and **13** stood out from the synthesized compounds in terms of IC_50_ values, their selectivity indices were high only for LoVo lines (SI = 6.5–9.1), they were very cytotoxic also to non-cancerous BALB/3T3 cells (IC_50_ = 6.2–11.6 nM). Among aryl amides and sulfonamides **12**–**16** and **21**, distinctive SI values were derived for colchicine derivatives containingnicotinic and isonicotinic amide residue (SI ranges 3.0 to 4.8). These results are noteworthy and suggest that extended and more detailed research of similar derivatives is necessary to determine the importance of ring aromaticity, its size or heteroatom type. Especially high SI values for MCF-7 were obtained for sulfamide **17** (SI = 9.8) and sulfonamide containing a morpholine ring **20** (SI = 6.6). These results deserve special attention and require further studies to determine whether the conversion of an amide bond to a sulfonamide bond with an appropriate substituent would allow obtaining compounds highly selective towards MCF7 cells, compared to colchicine **1**. As many as thirteen of the obtained derivatives exhibited SI ≥ 2 for LoVo cell line (see Figure 3). The results indicated that properly designed doubly modified (at C7 and C10 positions) colchicine derivatives can have greater selectivity towards cancer cells than the parental compound.

### 2.5. In Silico Determination of the Molecular Mode of Action

In the present study, computational investigation including molecular docking, molecular dynamics (MD) simulations, molecular mechanics generalized Born/surface area (MM/GBSA) binding free energy calculations and decomposition of pair-wise free energy on a per-residue basis were conducted to deeply explore the molecular basis for the binding of twenty novel double modified colchicine amides and sulfonamides to β-tubulin. The latter is one of the subunits of microtubules in the cytoskeleton structure of every eukaryotic cell, which is the target of many anticancer drugs. The twenty structures of colchicine derivatives described above were docked into the βІ-tubulin (the most abundant isotype in most cancer tumors) colchicine binding site.

**Figure 4:**
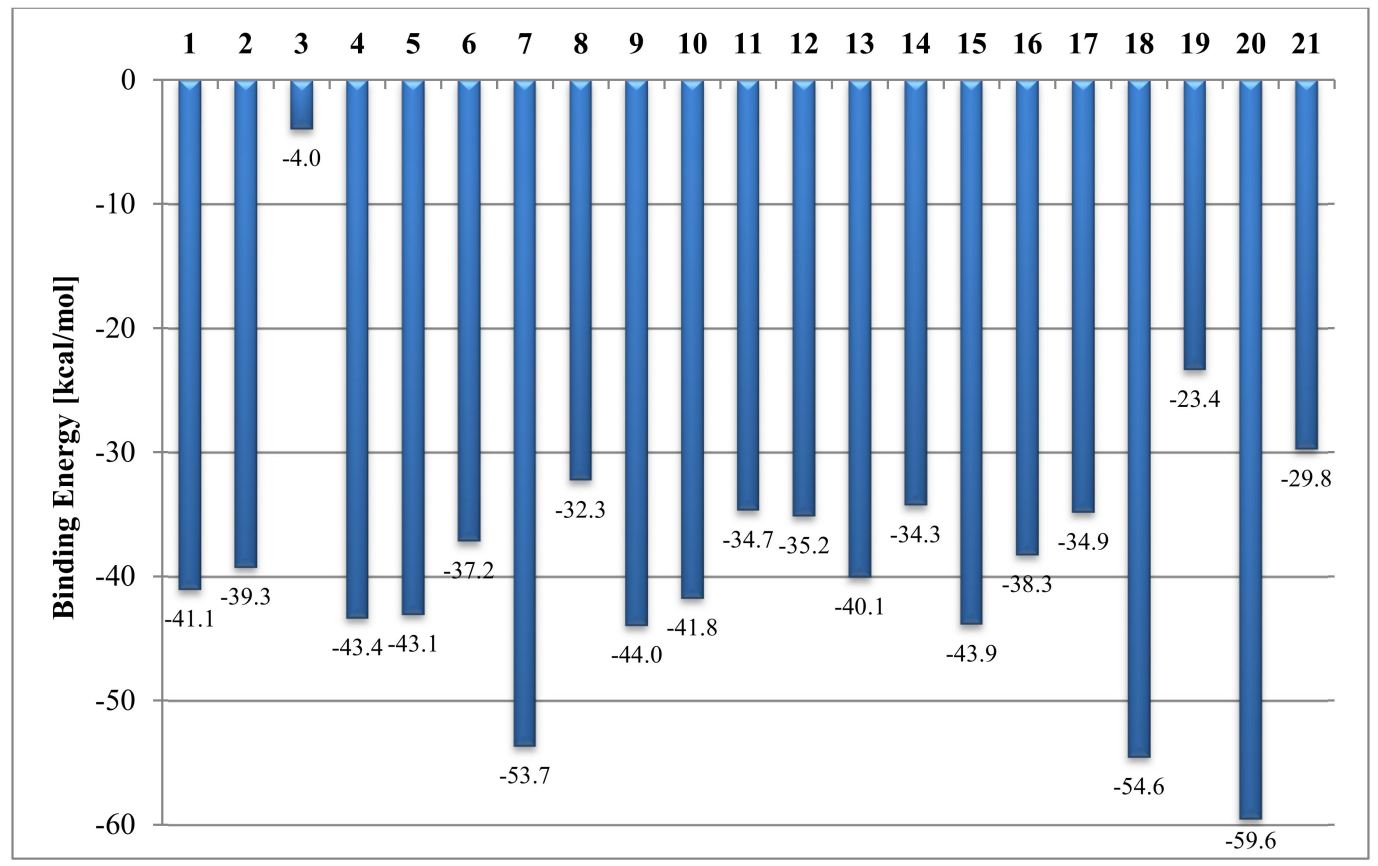
Comparison of binding energies of the tested compounds complexed with tubulin βI. Binding energies have been estimated using the MM/GBSA method.

On the basis of our computational predictions, according to increasing binding energy, the compounds are ordered as follows: **20** (−59.6), **18** (−54.6), **7** (−53.7), **9** (−44.0), **15** (−43.9), **4** (−43.4), **5** (−43.1), **10** (−41.8), **1** (−41.1), **13** (−40.1), **2** (−39.3), **16** (−38.3), **6** (−37.2), **12** (−35.2), **17** (−34.9), **11** (−34.7), **14** (−34.3), **8** (−32.3), **21** (−29.8), **19** (−23.4), **3** (−4.0) kcal/mol with the binding energies in the parenthesis given in units of kcal/mol. Binding energies of these compounds are shown in Figure 4 and Table 3.

In view of the calculated binding energies, we can conclude that there is no strong correlation (the lower the binding energy, the more biologically active the chemical compound) between *in silico* computer calculations (BE values, Figure 4) and in vitro activity results (IC_50_ values, Table 2).

The lowest binding energies, −59.6 and −53.7 kcal/mol, were shown by sulfonamide **20** (derivative with the morpholine ring) and amide **7** (derivative of palmitic acid), respectively. However, these compounds showed the weakest antiproliferative activity (highest IC_50_ values) among all compounds tested (**1**–**21**).

Compound **18** has the third lowest energy (−54.6 kcal/mol) and good cytotoxicity with IC_50_ from 9.3 to 15.5 nM for LoVo, A549 and MCF-7 cancer cells. The molecular-level computations indicate that **18** fits well to βI-tubulin and probably uses this binding site as a target. The majority of the other derivatives **2**, **4**–**6**, **8**–**17** exhibited binding energy less than −30.0 kcal/mol, so close to the energy of unmodified colchicine **1** (BE = −41.1 kcal/mol), which is a compound known to inhibit tubulin polymerization. These compounds bind to the active sites of βI-tubulin isotype and may therefore have a mechanism of action similar to **1** and may be colchicine binding site inhibitors, although their in vitro activities towards various cells characterized by an IC_50_ value (Table 2) were lower than that of colchicine **1**.

Derivative **3** showed the highest binding energy value (BE = −4.0 kcal/mol), therefore its antiproliferative activity is not exclusively the result of interaction with tubulin. This is an important finding because compound **3** was the only one which showed any possibility of breaking the drug resistance of the LoVo/DX line (RI = 9.2, see Table 2).

Therefore, we can see that the results only partly correlate with in vitro determined biological activity of these compounds as indicated though the corresponding IC_50_ values. This may be explained by several additional effects taking place in living cells compared to the computational simulations that focus only on the binding mode of the compounds to the target. Primarily off-target interactions involving efflux transporters with different affinities for the individual compounds and differences in solubility of these colchicine derivatives or their membrane permeability. Additionally, the lack of significant correlation may be due to the fact that in various cancer cells the expression of specific β-tubulin isotypes significantly varies [59]. We propose the inclusion of binding affinity calculations for these compounds with regard to not only other tubulin isotypes but also with respect to most important efflux transporters in order to minimize them and hence increase the activity of the drug in vitro and *in vivo*.

Schematic interactions of the compounds with CBS of βІ-tubulin residues are shown in Table 3.

In 3D representation, the interacting residues predicted from pairwise per-residue binding free energy decomposition calculations (E < −2 kcal/mol) are shown in stick presentation and their carbons and the ribbon are colored as green. Tubulin is shown in cartoon representation. Hydrogen bonds and their directionality are represented as black dashed arrows. The structures are color-coded as follows: tubulin αI, brown; tubulin βI, beige. Compounds are displayed with sticks and the atoms are colored as O (red), C (gray), N (blue), S (yellow), Cl (green) and F (pink). Binding energy defines the affinity of binding for colchicine derivatives complexed with tubulin βI. Binding energies are predicted by MM/GBSA method. The last column contains information about the active residues with the binding free energy decomposition (E_decomp_) less than −2 kcal/mol (the residues with E_decomp_ < −3 kcal/mol are highlighted in boldface). The last line contains the graphical key to help interpret the 2D part of the ligand interactions panel.

Derivative compounds are designed with different side chains at C7 position of colchicine **1**. Compounds **2**, **4**–**8** have alkyl chains of various lengths, straight and branched and compound **19** has an unsaturated alkyl chain. The second lowest binding energy compound **7** within all the compounds is in this group (BE = −53.6 kcal/mol). Compound **7** appears to be engaged in some interactions with α-tubulin (−3 < E_decomp_ < −2 kcal/mol). Most of the compounds in this group are bound through Lys785, Lys687 and Leu688 residues.

Compounds **9**–**11** were designed with alkyl chains of various length containing halogen atoms side chains at position C7 of colchicine **1**. In this group, the common binding residues with (−3 < E_decomp_ < −2 kcal/mol) are Ala179, Cys674, Asn691, while the strongest binding residues (E_decomp_ < −3 kcal/mol) are Leu688 and Lys785.

Compounds **12**–**16, 21** were designed with an aromatic group side chain without or with substituents at C7 position of colchicine **1**. The same trend of strong binding of Leu688 and Lys785 residues with the compounds is seen in this group. The binding energies in this group are on the higher side compared to the other compounds.

As the last group, compounds **17**–**18** and **20** contain the sulfonamide moieties at position C7 of colchicine **1**. The strongest binding residues in this group belong to both α-tubulin (Gln10 and Ser177) in compound **17**, (Gln10, Gly142, Gly143, Thr144 and Ser177) in compound **18** and (Asp68 and Asn100) in compound **20** with (E_decomp_ < −3 kcal/mol) and β-tubulin (Asp684, Asn691, Lys785) in compound **17** and Lys687 in compound **20**. The lowest binding energy between all the compounds characterizes compounds **20** and **18**, which mainly bind to α-tubulin.

In the experimental part of the study, the highest IC_50_ values (the weakest activity) were found for compound **7** from the amides and compound **20** from the sulfonamides studied against all cancer cell lines tested. According to our computational analysis, although these compounds show the lowest binding energies, they do not bind to the CBS, which is located in β-tubulin and has the tendency to go beyond and also bind to α-tubulin.

## 3. Materials and Methods

### 3.1. General

All solvents, substrates and reagents were obtained from TriMen Chemicals (Poland) or Sigma Aldrich and were used without further purification. CDCl_3_ and CD_2_Cl_2_ spectral grade solvents were stored over 3 Å molecular sieves for several days. TLC analysis was performed using pre-coated glass plates (0.2 mm thickness, GF-254, pore size 60 Å) from Agela Technologies and spots were visualized by UV-light. Products were purified by flash chromatography using high-purity grade silica gel (pore size 60 Å, 230–400 mesh particle size, 40–63 μm particle size) from SiliCycle Inc. Preparative HPLC was performed on LC-20AP Shimadzu with ELSD-LTII detector equipped with Phenomenex Luna C18 250 × 21 mm, 5 μm column eluted with 20 mL/min flow over 20 min of acetonitrile in water. Solvents were removed using a rotary evaporator.

### 3.2. Spectroscopic Measurements

NMR spectra were recorded on Bruker Avance DRX 500 (^1^H NMR at 500 MHz and ^13^C NMR at 126 MHz) magnetic resonance spectrometers. ^1^H NMR spectra are reported in chemical shifts downfield from TMS using the respective residual solvent peak as internal standard (CDCl_3_ δ 7.26 ppm, CD_2_Cl_2_ δ 5.32 ppm, (CD_3_)_2_SO δ 2.50 ppm). ^1^H NMR spectra are described as follows: chemical shift (δ, ppm), multiplicity (s = singlet, d = doublet, t = triplet, q = quartet, dd = doublet of doublets, dt = doublet of triplets, dq = doublet of quartets, m = multiplet), coupling constant (*J*) in Hz, and integration. ^13^C NMR spectra are described in chemical shifts downfield from TMS using the respective residual solvent peak as internal standard (CDCl_3_ δ 77.16 ppm, CD_2_Cl_2_ δ 53.84 ppm and (CD_3_)_2_SO δ 39.52 ppm).

Electrospray ionization (ESI) mass spectra were obtained on a Waters Alliance 2695 separation module with a PDA 2996 UV detector and Waters Micromass ZQ 2000 mass detector equipped with Restek Ultra Biphenyl 50 × 3 mm, 3 μm column eluted with 0.3 mL/min flow of 3–100% gradient (over 6 min) of acetonitrile in water.

### 3.3. Synthesis

#### 3.3.1. Synthesis of **2**

To a solution of **1** (1.0 equiv.) in EtOH, a methylamine (solution 33% in EtOH, 10.0 equiv.) was added. The mixture was stirred at reflux for 24 h and then concentrated under reduced pressure to dryness. The residue was purified using column flash chromatography (silica gel; DCM/MeOH) and next lyophilized from dioxane to give the pure product **2** as a yellow solid with a yield of 80%.

ESI-MS for C_22_H_26_N_2_O_5_ (*m*/*z*): [M + H]^+^ 399, [M + Na]^+^ 421, [2M + H]^+^ 797, [2M + Na]^+^ 819, [M – H]^−^ 397, [M + HCOO^−^]^−^ 443.

^1^H NMR (500 MHz, CDCl_3_) δ 8.70 (d, *J* = 6.4 Hz, 1H), 7.58 (s, 1H), 7.46 (d, *J* = 11.1 Hz, 1H), 7.28–7.25 (m, 1H), 6.58 (d, *J* = 11.3 Hz, 1H), 6.52 (s, 1H), 4.73–4.64 (m, 1H), 3.93 (s, 3H), 3.88 (s, 3H), 3.61 (s, 3H), 3.08 (d, *J* = 5.4 Hz, 3H), 2.47–2.43 (m, 1H), 2.37–2.31 (m, 1H), 2.29–2.22 (m, 1H), 2.02–1.96 (m, 1H), 1.94 (s, 3H).

^13^C NMR (126 MHz, CDCl_3_) δ 175.11, 170.19, 155.23, 152.91, 151.61, 151.10, 141.51, 139.37, 134.66, 130.42, 126.93, 122.81, 108.28, 107.20, 61.46, 61.40, 56.16, 52.72, 37.06, 30.15, 29.53, 22.74.

#### 3.3.2. Synthesis of **3**

To a solution of compound **2** (1.0 equiv.) in dioxane, 2M HCl (10.0 equiv.) was added and the mixture was stirred at reflux. Reaction progress was monitored by LC-MS. Then the reaction mixture was neutralized with 4M NaOH to pH~10 and extracted four times with EtOAc. The organic layers were combined, washed with brine, dried over Na_2_SO_4_, filtered and evaporated under reduced pressure. The residue was purified using column flash chromatography (silica gel; DCM/MeOH) and next lyophilized from dioxane to give the pure product **3** as a yellow solid with a yield of 73%.

ESI-MS for C_20_H_24_N_2_O_4_ (*m*/*z*): [M + H]^+^ 357, [M + Na]^+^ 379, [2M + Na]^+^ 735.

^1^H NMR (500 MHz, CDCl_3_) δ 7.61 (s, 1H), 7.33 (d, *J* = 11.1 Hz, 1H), 7.23–7.21 (m, 1H), 6.50 (s, 1H), 6.50 (d, *J* = 11.4 Hz, 2H), 3.87 (s, 3H), 3.87 (s, 3H), 3.75–3.72 (m, 1H), 3.59 (s, 3H), 3.05 (d, *J* = 5.5 Hz, 3H), 2.41–2.37 (m, 1H), 2.33–2.31 (m, 2H), 2.25 (s, 2H), 1.71–1.61 (m, 1H).

^13^C NMR (126 MHz, CDCl_3_) δ 175.57, 154.99, 153.25, 152.76, 150.59, 141.06, 138.73, 135.41, 129.74, 126.63, 123.68, 107.35, 106.94, 61.19, 60.84, 56.06, 54.01, 40.90, 30.69, 29.48.

#### 3.3.3. General Procedure for the Synthesis of Colchicine Derivatives **4**, **8**, **13**, **15**–**16**, **18** and **21**

Compounds **4**, **8**, **13**, **15**–**16**, **18** and **21** were obtained directly from compound **3**. To a solution of compound **3** (1.0 equiv.) and Et_3_N (3.0 equiv.) in DCM in an ice bath, the corresponding acid/sulfonyl/sulfamide chloride (1.1 equiv.) diluted with DCM was added slowly. Next the ice bath was removed and the reaction mixture was stirred at RT. Reaction progress was monitored by LC-MS. Then the reaction mixture was diluted with EtOAc, washed with H_2_O, 1M K_2_CO_3_, brine and dried over Na_2_SO_4_. The residue was purified using column flash chromatography (silica gel; DCM/MeOH) and next lyophilized from dioxane to give respective compound.

##### Compound **4**

Yellow solid, yield 86%.

ESI-MS for C_23_H_28_N_2_O_5_ (*m*/*z*): [M + H]^+^ 413, [M + Na]^+^ 435, [2M + H]^+^ 825, [2M + Na]^+^ 847, [M−H]^−^ 411, [M + HCOO^−^]^−^ 457.

^1^H NMR (500 MHz, CDCl_3_) δ 8.46 (s, 1H), 7.53 (s, 1H), 7.44 (d, *J* = 11.1 Hz, 1H), 7.25–7.21 (m, 1H), 6.55 (d, *J* = 11.4 Hz, 1H), 6.50 (s, 1H), 4.72–4.65 (m, 1H), 3.91 (s, 3H), 3.86 (s, 3H), 3.61 (s, 3H), 3.06 (d, *J* = 5.4 Hz, 3H), 2.44–2.40 (m, 1H), 2.33–2.30 (m, 1H), 2.26–2.17 (m, 3H), 1.94–1.88 (m, 1H), 1.05 (t, *J* = 7.6 Hz, 3H).

^13^C NMR (126 MHz, CDCl_3_) δ 175.09, 173.89, 155.20, 152.86, 151.64, 151.09, 141.49, 139.23, 134.67, 130.33, 126.95, 122.94, 108.14, 107.20, 61.44, 56.14, 52.37, 37.20, 30.18, 29.53, 29.11, 9.72.

##### Compound **8**

Yellow solid, yield 96%.

ESI-MS for C_24_H_30_N_2_O_5_ (*m*/*z*): [M + H]^+^ 427, [M + Na]^+^ 449, [2M + H]^+^ 853, [2M + Na]^+^ 875, [M−H]^−^ 425, [M + HCOO^−^]^−^ 471.

^1^H NMR (500 MHz, CDCl_3_) δ 7.48 (d, *J* = 11.2 Hz, 1H), 7.45 (s, 1H), 7.31–7.28 (m, 1H), 6.67 (d, *J* = 7.3 Hz, 1H), 6.59 (d, *J* = 11.3 Hz, 1H), 6.53 (s, 1H), 4.75–4.63 (m, 1H), 3.94 (s, 3H), 3.89 (s, 3H), 3.63 (s, 3H), 3.10 (d, *J* = 5.4 Hz, 3H), 2.53–2.43 (m, 2H), 2.38–2.32 (m, 1H), 2.28–2.20 (m, 1H), 1.93–1.82 (m, 1H), 1.14 (t, *J* = 6.7 Hz, 6H).

^13^C NMR (126 MHz, CDCl_3_) δ 176.86, 175.21, 155.19, 152.85, 151.15, 141.56, 139.08, 134.55, 130.09, 126.97, 123.07, 107.91, 107.22, 61.51, 61.45, 56.13, 51.96, 37.56, 35.21, 30.19, 29.54, 19.70, 19.60.

##### Compound **13**

Yellow solid, yield 86%.

ESI-MS for C_27_H_27_ClN_2_O_5_ (*m*/*z*): [M + H]^+^ 495/497, [M + Na]^+^ 517, [2M + H]^+^ 989/991, [M-H]^−^ 493/495, [M + HCOO^−^]^−^ 540.

^1^H NMR (500 MHz, CD_2_Cl_2_) δ 8.12 (d, *J* = 7.5 Hz, 1H), 7.60 (s, 1H), 7.42–7.39 (m, 2H), 7.29–7.22 (m, 2H), 7.19 (t, *J* = 7.5 Hz, 1H), 7.06 (t, *J* = 7.4 Hz, 1H), 6.60 (s, 1H), 6.53 (d, *J* = 11.3 Hz, 1H), 4.88–4.83 (m, 1H), 3.91 (s, 3H), 3.89 (s, 3H), 3.71 (s, 3H), 3.03 (d, *J* = 5.2 Hz, 3H), 2.52–2,48 (m, 1H), 2.42–2.31 (m, 2H), 2.14–2.04 (m, 1H).

^13^C NMR (126 MHz, CD_2_Cl_2_) δ 174.51, 165.99, 155.24, 153.11, 150.94, 150.51, 141.52, 139.19, 135.71, 134.71, 130.64, 130.43, 130.01, 129.60, 129.58, 126.83, 126.79, 123.26, 107.90, 107.46, 61.08, 61.07, 56.02, 52.86, 37.83, 30.11, 29.35.

##### Compound **15**

Yellow solid, yield 23%.

ESI-MS for C_26_H_27_N_3_O_5_ (*m*/*z*): [M + H]^+^ 462, [M + Na]^+^ 484, [2M + H]^+^ 923, [2M + Na]^+^ 945, [M-H]^−^ 460, [M + HCOO^−^]^−^ 506.

^1^H NMR (500 MHz, CDCl_3_) δ 9.35 (d, *J* = 7.0 Hz, 1H), 9.07 (d, *J* = 1.8 Hz, 1H), 8.48 (dd, *J* = 4.8, 1.5 Hz, 1H), 8.13–8.06 (m, 1H), 7.65 (s, 1H), 7.51 (d, *J* = 11.2 Hz, 1H), 7.31 (q, *J* = 5.1 Hz, 1H), 7.05 (dd, *J* = 7.9, 4.9 Hz, 1H), 6.61 (d, *J* = 11.4 Hz, 1H), 6.54 (s, 1H), 4.99–4.86 (m, 1H), 3.95 (s, 3H), 3.89 (s, 3H), 3.70 (s, 3H), 3.08 (d, *J* = 5.4 Hz, 3H), 2.51–2.45 (m, 1H), 2.40–2.34 (m, 1H), 2.33–2.27 (m, 1H), 2.17–2.09 (m, 1H).

^13^C NMR (126 MHz, CDCl_3_) δ 174.69, 165.09, 155.37, 153.01, 151.82, 151.42, 151.15, 148.87, 141.59, 139.62, 134.89, 134.61, 130.41, 129.34, 126.86, 123.08, 122.79, 108.49, 107.27, 61.53, 61.48, 56.15, 53.22, 36.83, 30.28, 29.58.

##### Compound **16**

Yellow solid, yield 39%.

ESI-MS for C_26_H_27_N_3_O_5_ (*m*/*z*): [M + H]^+^ 462, [M + Na]^+^ 484, [2M + H]^+^ 923, [2M + Na]^+^ 945, [M-H]^−^ 460, [M + HCOO^−^]^−^ 506.

^1^H NMR (500 MHz, CDCl_3_) δ 9.71 (d, *J* = 6.6 Hz, 1H), 8.37 (d, *J* = 5.8 Hz, 2H), 7.69 (s, 1H), 7.64 (d, *J* = 5.8 Hz, 2H), 7.54 (d, *J* = 11.2 Hz, 1H), 7.32–7.28 (m, 1H), 6.65 (d, *J* = 11.4 Hz, 1H), 6.53 (s, 1H), 4.98–4.81 (m, 1H), 3.95 (s, 3H), 3.87 (s, 3H), 3.71 (s, 3H), 3.10 (d, *J* = 5.4 Hz, 3H), 2.50–2.41 (m, 1H), 2.41–2.25 (m, 2H), 2.16–2.11 (m, 1H).

^13^C NMR (126 MHz, CDCl_3_) δ 174.51, 164.91, 155.37, 153.05, 151.64, 151.17, 150.18, 141.59, 140.46, 139.81, 134.59, 130.64, 126.78, 122.68, 120.99, 108.68, 107.27, 61.55, 61.47, 56.14, 53.46, 36.50, 30.29, 29.60.

##### Compound **18**

Yellow solid, yield 38%.

ESI-MS for C_22_H_29_N_3_O_6_S (*m*/*z*): [M + H]^+^ 464, [M + Na]^+^ 486, [2M + H]^+^ 927, [2M + Na]^+^ 949, [M-H]^−^ 462.

^1^H NMR (500 MHz, CDCl_3_) δ 7.85 (s, 1H), 7.45–7.41 (m, 2H), 6.58 (d, *J* = 11.3 Hz, 1H), 6.54 (s, 1H), 6.49–6.46 (m, 1H), 4.33–4.19 (m, 1H), 3.93–3.89 (m, 6H), 3.57 (s, 3H), 3.10 (d, *J* = 5.1 Hz, 3H), 2.58 (s, 6H), 2.49–2.39 (m, 2H), 2.35–2.26 (m, 1H), 2.01–1.90 (m, 1H).

^13^C NMR (126 MHz, CDCl_3_) δ 174.73, 155.46, 153.08, 150.77, 150.08, 141.39, 139.41, 134.59, 129.64, 126.34, 124.53, 108.22, 107.43, 61.34, 60.80, 56.48, 56.05, 39.91, 37.95, 30.50, 29.57.

##### Compound **21**

Yellow solid, yield 83%.

ESI-MS for C_27_H_27_F_3_N_2_O_6_S (*m*/*z*): [M + H]^+^ 565, [M + Na]^+^ 587, [2M + H]^+^ 1129, [2M + Na]^+^ 1151, [M-H]^−^ 563.

^1^H NMR (500 MHz, CDCl_3_) δ 8.72 (d, *J* = 7.5 Hz, 1H), 8.07 (s, 1H), 7.92 (s, 1H), 7.84 (d, *J* = 8.2 Hz, 2H), 7.53 (d, *J* = 8.3 Hz, 2H), 7.48 (d, *J* = 11.5 Hz, 1H), 6.66 (d, *J* = 11.8 Hz, 1H), 6.46 (s, 1H), 4.16 (q, *J* = 8.8 Hz, 1H), 3.91 (s, 3H), 3.86 (s, 3H), 3.58 (s, 3H), 3.08 (d, *J* = 5.2 Hz, 3H), 2.45–2.31 (m, 1H), 2.31–2.18 (m, 2H), 2.16–2.10 (m, 1H).

^13^C NMR (126 MHz, CDCl_3_) δ 170.13, 155.43, 153.53, 150.65, 150.15, 144.53, 141.35, 134.75, 133.34, 131.89, 127.57, 125.82, 125.79, 125.49, 124.38, 123.51, 122.21, 111.26, 107.42, 61.29, 60.95, 56.63, 56.04, 38.98, 30.26, 29.78.

#### 3.3.4. General Procedure for the Synthesis of Colchicine Derivatives **5**–**7**, **9**–**12** and **14**

Compounds **5**–**7**, **9**–**12** and **14** were obtained directly from compound **3**. To a solution of compound **3** (1.0 equiv.) in DCM corresponding carboxylic acid (1.1 equiv.) and 1-(3-dimethylaminopropyl)-3-ethylcarbodiimide hydrochloride (EDCI, 1.1 equiv.) were added. Reaction progress was monitored by LC-MS. Then the reaction mixture was diluted with EtOAc, washed with H_2_O, 1M K_2_CO_3_, brine and dried over Na_2_SO_4_. The residue was purified using column flash chromatography (silica gel; DCM/MeOH) and next lyophilized from dioxane to give respective compound.

##### Compound **5**

Yellow solid, yield 93%.

ESI-MS for C_24_H_30_N_2_O_5_ (*m*/*z*): [M + H]^+^ 427, [M + Na]^+^ 449, [2M + H]^+^ 853, [2M + Na]^+^ 875, [M-H]^−^ 425, [M + HCOO^−^]^−^ 471.

^1^H NMR (500 MHz, CDCl_3_) δ 7.88 (s, 1H), 7.51 (s, 1H), 7.45 (d, *J* = 11.1 Hz, 1H), 7.27–7.25 (m, 1H), 6.56 (d, *J* = 11.3 Hz, 1H), 6.51 (s, 1H), 4.76–4.68 (m, 1H), 3.92 (s, 3H), 3.87 (s, 3H), 3.61 (s, 3H), 3.08 (d, *J* = 5.4 Hz, 3H), 2.45–2.41 (m, 1H), 2.35–2.27 (m, 1H), 2.25–2.17 (m, 3H), 1.91 –1.85 (m, 1H), 1.65–1.55 (m, 2H), 0.87 (t, *J* = 7.4 Hz, 3H).

^13^C NMR (126 MHz, CDCl_3_) δ 175.10, 172.96, 155.22, 152.87, 151.43, 151.13, 141.52, 139.20, 134.61, 130.31, 126.94, 123.10, 108.13, 107.22, 61.45, 56.13, 52.21, 38.22, 37.45, 30.18, 29.54, 19.09, 13.93.

##### Compound **6**

Yellow solid, yield 73%.

ESI-MS for C_25_H_32_N_2_O_5_ (*m*/*z*): [M + H]^+^ 441, [M + Na]^+^ 463, [2M + H]^+^ 881, [2M + Na]^+^ 903, [M-H]^−^ 439, [M + HCOO^−^]^−^ 485.

^1^H NMR (500 MHz, CDCl_3_) δ 7.86 (s, 1H), 7.50 (s, 1H), 7.44 (d, *J* = 11.1 Hz, 1H), 7.26–7.21 (m, 1H), 6.55 (d, *J* = 11.3 Hz, 1H), 6.51 (s, 1H), 4.76–4.68 (m, 1H), 3.93 (s, 3H), 3.88 (s, 3H), 3.61 (s, 3H), 3.08 (d, *J* = 5.4 Hz, 3H), 2.45–2.41 (m, 1H), 2.36–2.30 (m, 1H), 2.25–2.18 (m, 3H), 1.93–1.84 (m, 1H), 1.58–1.51 (m, 2H), 1.30–1.22 (m, 2H), 0.81 (t, *J* = 7.3 Hz, 3H).

^13^C NMR (126 MHz, CDCl_3_) δ 175.19, 173.02, 155.19, 152.85, 151.32, 151.15, 141.54, 139.11, 134.59, 130.19, 126.98, 123.13, 107.97, 107.20, 61.44, 56.13, 52.20, 37.46, 36.05, 30.19, 29.53, 27.70, 22.46, 13.76.

##### Compound **7**

Yellow solid, yield 24%.

ESI-MS for C_36_H_54_N_2_O_5_ (*m*/*z*): [M + H]^+^ 595, [2M + H]^+^ 1189, [M-H]^−^ 593, [M + HCOO^−^]^−^ 639.

^1^H NMR (500 MHz, CDCl_3_) δ 8.18–7.90 (m, 2H), 7.73–7.53 (m, 2H), 6.88 (d, *J* = 10.1 Hz, 1H), 6.55 (s, 1H), 4.74 (s, 1H), 3.93 (s, 3H), 3.89 (s, 3H), 3.62 (s, 3H), 3.19 (s, 3H), 2.47 (d, *J* = 7.4 Hz, 1H), 2.39–2.08 (m, 5H), 1.54–1.49 (m, 2H), 1.28–1.16 (m, 26H), 0.86 (t, *J* = 7.0 Hz, 3H).

^13^C NMR (126 MHz, CDCl_3_) δ 175.09, 173.07, 155.23, 152.89, 151.32, 151.14, 141.55, 139.25, 134.58, 130.35, 126.91, 123.12, 108.24, 107.23, 61.45, 56.12, 52.16, 37.56, 36.39, 31.95, 30.18, 29.73, 29.69, 29.54, 29.53, 29.43, 29.39, 25.64, 22.72, 14.16.

##### Compound **9**

Yellow solid, yield 83%.

ESI-MS for C_22_H_25_ClN_2_O_5_ (*m*/*z*): [M + H]^+^ 433/435, [M + Na]^+^ 455/457, [2M + H]^+^ 865, [2M + Na]^+^ 887, [M-H]^−^ 431.

^1^H NMR (500 MHz, CDCl_3_) δ 8.60–8.38 (m, 1H), 7.48–7.39 (m, 2H), 7.29 (q, *J* = 5.2 Hz, 1H), 6.56 (d, *J* = 11.4 Hz, 1H), 6.52 (s, 1H), 4.75–4.63 (m, 1H), 4.08–3.95 (m, 2H), 3.92 (s, 3H), 3.87 (s, 3H), 3.60 (s, 3H), 3.07 (d, *J* = 5.3 Hz, 3H), 2.50–2.43 (m, 1H), 2.40–2.31 (m, 1H), 2.28–2.21 (m, 1H), 2.04–1.93 (m, 1H).

^13^C NMR (126 MHz, CDCl_3_) δ 175.04, 166.12, 155.33, 152.97, 151.10, 150.18,141.57, 139.37, 134.41, 129.97, 126.80, 122.73, 108.18, 107.27, 61.45, 61.33, 56.14, 52.91, 42.63, 37.17, 30.02, 29.55.

##### Compound **10**

Yellow solid, yield 46%.

ESI-MS for C_22_H_24_Cl_2_N_2_O_5_ (*m*/*z*): [M + H]^+^ 467/469, [M + Na]^+^ 489/491, [2M + H]^+^ 933/935, [2M + Na]^+^ 957/959, [M-H]^−^ 465/467.

^1^H NMR (500 MHz, CDCl_3_) δ 9.53 (d, *J* = 7.2 Hz, 1H), 7.56 (s, 1H), 7.53 (d, *J* = 11.2 Hz, 1H), 7.34 (q, *J* = 5.2 Hz, 1H), 6.64 (d, *J* = 11.4 Hz, 1H), 6.52 (s, 1H), 6.20 (s, 1H), 4.77–4.69 (m, 1H), 3.94 (s, 3H), 3.89 (s, 3H), 3.63 (s, 3H), 3.12 (d, *J* = 5.4 Hz, 3H), 2.46–2.44 (m, 1H), 2.38–2.23 (m, 2H), 1.97–1.91 (m, 1H).

^13^C NMR (126 MHz, CDCl_3_) δ 174.73, 164.19, 155.51, 153.12, 151.17, 150.51, 141.69, 139.81, 134.31, 130.45, 126.66, 122.71, 108.80, 107.36, 66.56, 61.52, 61.48, 56.17, 53.28, 37.17, 30.06, 29.66.

##### Compound **11**

Yellow solid, yield 89%.

ESI-MS for C_24_H_27_F_3_N_2_O_5_ (*m*/*z*): [M + H]^+^ 481, [M + Na]^+^ 503, [2M + H]^+^ 961, [2M + Na]^+^ 983, [M-H]^−^ 479, [M + HCOO^−^]^−^ 525.

^1^H NMR (500 MHz, CDCl_3_) δ 8.77–8.65 (m, 1H), 7.56 (s, 1H), 7.49 (d, *J* = 11.2 Hz, 1H), 7.35–7.28 (m, 1H), 6.61 (d, *J* = 11.4 Hz, 1H), 6.51 (s, 1H), 4.75–4.70 (m, 1H), 3.94 (s, 3H), 3.88 (s, 3H), 3.62 (s, 3H), 3.10 (d, *J* = 5.4 Hz, 3H), 2.56–2.46 (m, 2H), 2.45–2.27 (m, 4H), 2.25–2.20 (m, 1H), 1.90–1.84 (m, 1H).

^13^C NMR (126 MHz, CDCl_3_) δ 174.99, 169.79, 155.36, 152.97, 151.33, 151.12, 141.57, 139.53, 134.54, 130.36, 127.98, 126.81, 122.89, 108.41, 107.25, 61.46, 61.34, 56.13, 52.52, 37.37, 30.16, 29.56, 29.33, 28.17.

##### Compound **12**

Yellow solid, yield 89%.

ESI-MS for C_27_H_28_N_2_O_5_ (*m*/*z*): [M + H]^+^ 461, [M + Na]^+^ 483, [2M + H]^+^ 921, [2M + Na]^+^ 943, [M-H]^−^ 459, [M + HCOO^−^]^−^ 505.

^1^H NMR (500 MHz, CDCl_3_) δ 8.36 (d, *J* = 7.3 Hz, 1H), 7.81 (d, *J* = 7.2 Hz, 2H), 7.68 (s, 1H), 7.49 (d, *J* = 11.2 Hz, 1H), 7.29–7.23 (m, 2H), 7.16 (t, *J* = 7.7 Hz, 2H), 6.57 (d, *J* = 11.4 Hz, 1H), 6.53 (s, 1H), 4.99–4.93 (m, 1H), 3.96 (s, 3H), 3.89 (s, 3H), 3.70 (s, 3H), 3.06 (d, *J* = 5.4 Hz, 3H), 2.50–2.45 (m, 1H), 2.41–2.37 (m, 1H), 2.34–2.26 (m, 1H), 2.11–2.06 (m, 1H).

^13^C NMR (126 MHz, CDCl_3_) δ 175.02, 167.10, 155.26, 152.91, 151.30, 151.20, 141.57, 139.18, 134.65, 133.84, 131.31, 130.17, 128.28, 127.17, 127.01, 123.23, 108.01, 107.28, 61.53, 61.49, 56.14, 52.85, 37.19, 30.30, 29.53.

##### Compound **14**

Yellow solid, yield 57%.

ESI-MS for C_27_H_27_FN_2_O_5_ (*m*/*z*): [M + H]^+^ 479, [M + Na]^+^ 501, [2M + H]^+^ 957, [2M + Na]^+^ 979, [M-H]^−^ 477, [M + HCOO^−^]^−^ 523.

^1^H NMR (500 MHz, CDCl_3_) δ 8.75 (s, 1H), 7.68 (s, 1H), 7.59 (d, *J* = 7.5 Hz, 1H), 7.53–7.47 (m, 2H), 7.31–7.26 (m, 1H), 7.10–7.05 (m, 1H), 6.97–6.92 (m, 1H), 6.61 (d, *J* = 11.4 Hz, 1H), 6.55 (s, 1H), 4.99–4.90 (m, 1H), 3.96 (s, 3H), 3.90 (s, 3H), 3.70 (s, 3H), 3.09 (d, *J* = 5.4 Hz, 3H), 2.53–2.47 (m, 1H), 2.43–2.37 (m, 1H), 2.35–2.27 (m, 1H), 2.18–2.10 (m, 1H).

^13^C NMR (126 MHz, CDCl_3_) δ 174.84, 165.56, 155.31, 152.98, 151.38, 151.20, 141.59, 139.42, 134.63, 130.36, 129.84, 126.92, 123.06, 122.53, 118.29, 118.12, 114.70, 114.52, 108.29, 107.27, 61.52, 61.50, 56.15, 53.09, 37.00, 30.31, 29.56.

#### 3.3.5. Synthesis of **17**

To a solution of compound **3** (1.0 equiv.) in DCM, *N*-(*tert*-Butoxycarbonyl)-*N*-[4-(dimethylazaniumylidene)-1,4-dihydropyridin-1-ylsulfonyl]azanide (4.0 equiv.) was added and the mixture was stirred at RT. Reaction progress was monitored by LC-MS. Then the reaction mixture was diluted with EtOAc, washed with H_2_O, 10% citric acid, 1M K_2_CO_3_, brine and dried over Na_2_SO_4_. The residue was purified using column flash chromatography (silica gel; EtOAc/hexanes). To the obtained compound, 4M HCl/EtOAc was added and the reaction mixture was stirred at RT overnight. Next the solvent was removed under reduced pressure and the residue was purified using preparative HPLC (MeCN/H_2_O) and lyophilized from dioxane to give the pure hydrochloride product **17** as a yellow solid with a yield of 21%.

ESI-MS for C_20_H_25_N_3_O_6_S (*m*/*z*): [M + H]^+^ 436, [M + Na]^+^ 458, [2M + H]^+^ 871, [2M + Na]^+^ 893, [M-H]^−^ 434.

^1^H NMR (500 MHz, CDCl_3_) δ 8.19 (s, 2H), 7.50–7.39 (m, 2H), 6.66 (s, 1H), 6.57 (s, 1H), 4.41–4.32 (m, 1H), 3.94 (m, 6H), 3.56 (s, 3H), 3.11 (s, 3H), 2.59–2.46 (m, 2H), 2.56–2.49 (s, 1H), 2.21 (s, 1H).

#### 3.3.6. Synthesis of **19**

To a solution of compound **3** (1.0 equiv.) and Et_3_N (3.0 equiv.) in DCM in an ice bath, 2-chloroethanesulfonyl chloride (1.2 equiv.) diluted with DCM was slowly added. Next, the ice bath was removed and the reaction mixture was stirred at RT. Reaction progress was monitored by LC-MS. Then the reaction mixture was diluted with EtOAc, washed with 1M K_2_CO_3_, brine and dried over Na_2_SO_4_. The residue was purified using column flash chromatography (silica gel; EtOAc/MeOH) and lyophilized from dioxane to give the pure product **19** as a yellow solid with a yield of 64%.

ESI-MS for C_22_H_26_N_2_O_6_S (*m*/*z*): [M + H]^+^ 447, [2M + H]^+^ 893, [2M + Na]^+^ 915, [M-H]^−^ 445.

^1^H NMR (500 MHz, (CD_3_)_2_SO) δ 8.26 (d, *J* = 7.5 Hz, 1H), 8.04 (s, 1H), 7.41 (s, 1H), 7.23 (d, *J* = 11.2 Hz, 1H), 6.71 (s, 1H), 6.64 (d, *J* = 11.5 Hz, 1H), 6.48 (dd, *J* = 16.4, 9.9 Hz, 1H), 5.71 (dd, *J* = 21.1, 13.1 Hz, 2H), 3.84–3.76 (m, 4H), 3.73 (s, 3H), 3.37 (s, 3H), 2.95 (s, 3H), 2.49 (s, 1H), 2.11–2.01 (m, 2H), 1.88–1.82 (m, 1H).

^13^C NMR (126 MHz, (CD_3_)_2_SO) δ 173.10, 155.58, 153.04, 150.65, 149.28, 141.12, 139.36, 137.66, 134.78, 128.80, 126.48, 125.64, 123.98, 108.88, 108.07, 61.19, 60.73, 56.26, 55.99, 38.70, 29.82.

#### 3.3.7. Synthesis of **20**

Compound **20** was obtained directly from compound **19**. To **19** in DCM, morpholine was added in excess. Reaction progress was monitored by LC-MS. Then the mixture was diluted with EtOAc, washed with H_2_O, brine and dried over Na_2_SO_4_. The residue was purified using column flash chromatography (silica gel; DCM/MeOH) and subsequently lyophilized from dioxane to give the pure product **20** as a yellow solid with a yield of 63%.

ESI-MS for C_26_H_35_N_3_O_7_S (*m*/*z*): [M + H]^+^ 534, [2M + H]^+^ 1067, [M-H]^−^ 532.

^1^H NMR (500 MHz, CDCl_3_) δ 7.72 (s, 1H), 7.43 (d, *J* = 11.2 Hz, 1H), 7.40–7.38 (m, 1H), 6.84–6.50 (m, 1H), 6.56 (d, *J* = 11.3 Hz, 1H), 6.53 (s, 1H), 4.48–4.35 (m, 1H), 3.93 (s, 3H), 3.90 (s, 3H), 3.63 (t, *J* = 4.5 Hz, 4H), 3.58 (s, 3H), 3.17–3.11 (m, 1H), 3.10 (d, *J* = 5.4 Hz, 3H), 3.07–2.99 (m, 1H), 2.83–2.76 (m, 1H), 2.75–2.69 (m, 1H), 2.51–2.33 (m, 7H), 1.99–1.91 (m, 1H).

^13^C NMR (126 MHz, CDCl_3_) δ 175.05, 155.45, 153.09, 150.89, 149.61, 141.54, 139.38, 134.35, 129.35, 129.06, 128.25, 126.38, 124.07, 108.00, 107.38, 61.37, 61.02, 56.09, 55.65, 53.37, 52.68, 51.08, 39.95, 30.38, 29.56.

### 3.4. Single Crystal X-ray Measurement

The single crystals of colchicine derivatives (**6**, **11**, **12**, **14**, **15**, **16**, **18** and **19**) were used for data collection on a four-circle KUMA KM4 diffractometer equipped with two-dimensional CCD area detector at room (295(1) K) and low temperature (100(1) K. The graphite monochromatized Mo-Kα radiation (λ = 0.71073 Å) and the ω-scan technique (Δω = 1°) were used for data collection. Lattice parameters were refined by the least-squares methods at all reflection positions. One image was monitored as a standard after every 40 images for a control of the crystal stability. Data collection and reduction along with absorption correction were performed using the CrysAlis software package [60]. The structures were solved by direct methods using SHELXT [61] giving positions of almost all non-hydrogen atoms. The structures were refined using SHELXL–2018 [62] with the anisotropic thermal displacement parameters. Hydrogen atoms were refined as rigid. Visualizations of the structures were made with the Diamond 3.0 program [63]. Details of the data collection parameters, crystallographic data and final agreement parameters are collected in Supplementary Table S1. The structures have been deposited with the Cambridge Crystallographic Data Center in the CIF format, no. CCDC 1980349–1980354 for **6**, **11**, **12**, CCDC 1980341–1980344 for **14** and **15**, CCDC 1980339–1980340 for **16** and CCDC 1980345–1980348 for **18** and **19** at 100 K and RT, respectively. Copies of this information can be obtained free of charge from The Director, CCDC, 12 Union Road, Cambridge, CB2 1EZ, UK (fax: +44 1223 336 033); email: deposit@ccdc.cam.ac.uk or www: http://www.ccdc.cam.ac.uk).

### 3.5. DFT Molecular Modeling

Molecular orbital calculations with full geometry optimization of colchicine derivatives (**6**, **11**, **12**, **14**, **15**, **16**, **18** and **19**) were performed with the Gaussian09 program package [52]. All calculations were carried out with the DFT level using the Becke3-Lee–Yang–Parr correlation functional (B3LYP) [53,54,55,56] with the 6–31+G basis set assuming the geometry resulting from the X-ray diffraction study as the starting structure. As convergence criteria, the threshold limits of 0.00025 and 0.0012 a.u. were applied for the maximum force and the displacement, respectively. The three-dimensional molecular electrostatic potential (3D MESP) maps are obtained on the basis of the DFT (B3LYP/6–31G) optimized. The calculated 3D MESP is mapped onto the total electron density isosurface (0.008 eÅ^-3^) for each molecule. The color code of MESP maps is in the range of −0.05 eÅ^−1^ (red) to +0.05 eÅ^−1^ (blue).

### 3.6. In Vitro Antiproliferative Activity

#### 3.6.1. Cell Lines and Culturing Conditions

Four human cancer cell lines and one murine normal cell line were used to evaluate antiproliferative activity of colchicine and its derivatives **1**–**21**: human lung adenocarcinoma (A549), human breast adenocarcinoma (MCF-7), human colon adenocarcinoma cell lines sensitive and resistant to doxorubicin (LoVo) and (LoVo/DX) respectively, and normal murine embryonic fibroblast cell line (BALB/3T3). The A549 cell line was purchased from the European Collection of Authenticated Cell Cultures (ECACC, Salisbury, UK). The MCF-7, LoVo and LoVo/DX cell lines was purchased from American Type Culture Collection (ATCC, Manassas, VA, USA). All the cell lines are maintained in the Institute of Immunology and Experimental Therapy (IIET), Wroclaw, Poland.

Human lung adenocarcinoma cell line was cultured in a mixture of OptiMEM and RPMI 1640 (1:1) medium (IIET, Wroclaw, Poland), supplemented with 5% fetal bovine serum HyClone (GE Healthcare, USA) and 2 mM L-glutamine (Sigma-Aldrich, Germany). Human breast adenocarcinoma cell line was cultured in a mixture of Eagle medium (IIET, Wroclaw, Poland), supplemented with 10% fetal bovine serum, 2 mM l-glutamine, 8 μg/mL insulin and 1% amino acids (Sigma-Aldrich, Germany). Human colon adenocarcinoma cell lines were cultured in a mixture of OptiMEM and RPMI 1640 (1:1) medium (IIET, Wroclaw, Poland), supplemented with 5% fetal bovine serum HyClone (GE Healthcare, USA), 2 mM l-glutamine, 1 mM sodium pyruvate (Sigma-Aldrich, Germany) and 10 μg/100 mL doxorubicin (Accord) for LoVo/DX. Murine embryonic fibroblast cells were cultured in Dulbecco medium (Gibco), supplemented with 10% fetal bovine serum (GE Healthcare, USA) and 2 mM L-glutamine (Sigma-Aldrich, Germany). All culture media contained antibiotics: 100 U/mL penicillin (Polfa-Tarchomin, Poland) and 0.1 mg/mL streptomycin (Sigma Aldrich, Germany). All cell lines were cultured during entire experiment in humid atmosphere at 37 °C and 5% CO_2_.

#### 3.6.2. Cell Viability Assays

Twenty-four hours before adding the tested compounds, all cell lines were seeded in 96-well plates (Sarstedt, Germany) in appropriate media with 0.5 × 10^4^ cells per well for A549 cell line, 0.75 × 10^4^ cells per well for MCF-7 cell line and 1.0 × 10^4^ cells per well for LoVo, LoVo/DX and BALB/3T3 cell lines. All cell lines were exposed to each tested agent at different concentrations in the range 100–0.001 μg/mL for 72 h. The cells were also exposed to the reference drug cisplatin (Teva Pharmaceuticals, Poland) and doxorubicin (Accord Healthcare Limited, UK). Additionally, all cell lines were exposed to DMSO (solvent used for tested compounds) (POCh, Poland) at concentrations corresponding to those present in dilutions of tested agents. After 72 h sulforhodamine B assay (SRB) was performed [64].

##### SRB

After 72 h of incubation with the tested compounds, the cells were fixed in situ by gently adding of 50 μL per well of cold 50% trichloroacetic acid TCA (POCh, Poland) and were incubated at room temperature for one hour. Then the wells were washed four times with water and air dried. Next, 50 μL of 0.1% solution of sulforhodamine B (Sigma-Aldrich, Germany) in 1% acetic acid (POCh, Poland) were added to each well and were incubated at room temperature for 0.5 h. After incubation time, unbound dye was removed by washing plates four times with 1% acetic acid, whereas the stain bound to cells was solubilized with 150 μL of 10 mM Tris base (Sigma-Aldrich, Germany). Absorbance of each solution was read from a Synergy H4 Hybrid Multi-Mode Microplate Reader (BioTek Instruments, USA) at the 540 nm wavelength.

Results are presented as mean IC_50_ (concentration of the tested compound that inhibits cell proliferation by 50%) ± standard deviation. IC_50_ values were calculated in Cheburator 0.4, Dmitry Nevozhay software (version 1.2.0 software by Dmitry Nevozhay, 2004–2014, http://www.cheburator.nevozhay.com, freely available for each experiment [65]. Compounds at each concentration were tested in triplicates in a single experiment and each experiment was repeated at least three times independently. Results are summarized in Table 2.

### 3.7. Molecular Docking Studies

#### 3.7.1. Ligand Preparation

The ligand structures were prepared using Ligprep from the Schrödinger suite [66]. Conformations and tautomeric states were assigned to the ligands by following the ligand preparation protocol implemented in Schrödinger suite with default settings. LigPrep generates variants of the same ligand with different tautomeric, stereochemical, and ionization properties.

#### 3.7.2. Tubulin Model

The tubulin crystal structures available in the PDB are those for bovine protein. The bovine tubulin structure of tubulin (PDB ID: 1SA0) [67] was used as a template to construct the homology model of human αβ-tubulin isotypes (βI (UniProtKb: P07437), which is the most abundant isotype in most tumors using the Molecular Operating Environment (MOE) software package [68]. The sequence corresponding to the gene TUBA1A (UniProt ID: Q71U36) was chosen as a reference sequence for human tubulin, whereas gene TUBB associated to I isoform (UniProt ID: P07437) was chosen for human tubulin. Homology modeling was performed using MOE by setting the number of generated models to 10 and by selecting the final model based on MOE’s generalized Born/volume integral (GB/VI) scoring function. The models used in the simulations performed for the present study were developed earlier and described in our previous publications [69]. These earlier investigations can also be considered to be validations of the computational model employed here since they were directly compared to the corresponding experimental data.

#### 3.7.3. Molecular Dynamics Simulations

The missing hydrogens for heavy atoms were added using the tLEAP module of AMBER 14 with the AMBER14SB force field [70]. The protonation states of all ionizable residues were determined at pH = 7 using the MOE program [68]. Each protein model was solvated in a 12 Å box of TIP3P water. In order to bring the salt concentration to the physiological value of 0.15 M, 93 Na^+^ ions and 57 Cl^−^ ions were added. Minimization of the structure was carried out in two steps, using the steepest descent and conjugate gradient methods successively. At first, minimization was made in 2 ps on solvent atoms only, by restraining the protein-ligand complex. Next, minimization was run without the restraint in 10 ps. After minimization, the molecular dynamics (MD) simulations were carried out in three steps: heating, density equilibration, and production. At first, each solvated system was heated to 298 K for 50 ps, with weak restraints on all backbone atoms. Next, density equilibration was carried out for 50 ps of constant pressure equilibration at 298 K, with weak restraints. Finally, MD production runs were performed for all systems for 70 ns. The root-mean-square deviation (RMSD) of both the entire tubulin structure and the colchicine binding site were found to reach a plateau after 40 ns. Clustering analysis of the last 30 ns of the generated MD trajectory was carried out using the Amber’s CPPTRAJ program [71] to identify representative conformations of the tubulin dimer. Clustering was made via the hierarchical agglomerative approach using the RMSD of atoms in the colchicine binding site as a metric. An RMSD cutoff of 1.0 Å was set to differentiate the clusters. On the basis of the clustering analysis, three representative structures of the tubulin dimer were found. The docking was performed on all the three representative structures and the one with the highest docking score was selected, which was the largest cluster (about 70% of the simulation) conformation of the tubulin structure. During the modeling, the cofactors including GTP, GDP, colchicine, and the magnesium ion located at the interface between α–and β–monomers were kept as part of the environment and included in the refinement step.

#### 3.7.4. Docking Simulations

We used the AutoDock Vina [72] program to predict the binding pose of the ligands under flexible ligand and rigid receptor conditions. Dockbox package was used to facilitate preparing docking inputs and post-processing of the docking results [73]. Docking simulations performed with a cubic box (size 30.0 Å) were centered at the center of binding pockets and the docking was run separately on tubulin structure. Every generated pose was energy-minimized using Amber14 by keeping the protein fixed and was re-scored using the MOE’s GBVI/WSA dG scoring function [68]. No constraints were applied in the docking studies. For each compound/protein-structure pair, the pose with the best score was identified and used as an initial configuration for molecular mechanics Gibbs–Boltzmann surface area MM/GBSA computations.

#### 3.7.5. Binding Energy and Pairwise Per-Residue Free Energy Decomposition Calculations Using MM/GBSA Method

The MM/GBSA technique is used to calculate the free energy associated with the binding of double modified colchicine amides and sulfonamides [74]. This method combines molecular mechanics with continuum solvation models. We performed MM-GBSA integrated in Amber. The binding free energy is estimated as:

(1) $$\Delta G_{bind}={\bar{G}}_{complex}-\left[{\bar{G}}_{protein}+{\bar{G}}_{ligand}\right]$$

where G is the average free energy of the complex, protein, and ligand, are calculated according to the equation:

(2) $$\bar{G}={\bar{E}}_{M+}{\bar{G}}_{solvation}-T\bar{S}$$

where EMM are determined with the SANDER program and represent the internal energy (bond, angle, and dihedral), van der Waals and electrostatic interactions (see Equation (3)). TS is the entropy contribution estimated using normal mode (nmode) analysis.

(3) $${\bar{E}}_{M\;}={\bar{E}}_{\mathrm{int}\;}+{\bar{E}}_{\mathrm{elec}\;}+{\bar{E}}_{\mathrm{vdW}\;}$$

The solvation free energy can be calculated as the sum of polar and nonpolar contributions. The polar parts are obtained by using the generalized-born (GB) model—resulting in the MM/GBSA method, whereas the nonpolar terms are estimated from a linear relation (Equation (4)) to the solvent accessible surface area (SASA).

(4) $${\bar{G}}_{non-polar}=\gamma \;SASA+b$$

In the present study, a 2 ns-duration MD trajectory was run in TIP3P water using Amber14, for every top pose generated at the end of the docking step. It is worth noting that to assess the performance of MM/GBSA methodology [75], we evaluated the prediction accuracy of this method by various simulation protocols including 1 ns MD production calculations using PDBbind data set. Too long an MD simulation could be prejudicious for the overall success of the MM/GBSA method. According to this study and the common practice to calculate binding energies using MM/GBSA, we have decided to run MD production simulation for 2 ns. The MM/GBSA calculations were performed on a subset of 200 frames collected at regular time intervals from the trajectory. For PB calculations, an ionic strength of 0.0 nM (istrng = 0.0) and a solvent probe radius of 1.6 Å (prbrad = 1.6) were used. For GB calculations, the igb parameter was set to 5 that corresponds to a modified GB model equivalent to model II in reference. Pairwise free energy decomposition analysis was performed using the MM/GBSA decomposition process by the MM/GBSA program in AMBER 14 to compute the interaction between the inhibitors and each residue. For each of the tested compounds, the active residues estimated from MM/GBSA decomposition process and the best GB score out of the trajectories associated with the representative structures of the tubulin dimer were collected and are reported in Table 3 and Figure 4.

### 3.8. Calculation clogP 

We used the Molinspiration online database (http://www.molinspiration.com, free of charge) to predict the *clogP* values for all synthesized compounds and collected them in Table 3 [76].

## 4. Conclusions

In an attempt to discover novel potent inhibitors of microtubule dynamics, eighteen novel double modified colchicine analogs comprising methylamine substituent at position C10 of ring C and amide or sulfonamide substituents at position C7 of ring B were successfully synthesized, purified and their structures were confirmed by spectroscopic analyses as well as X-ray measurements. Four human cancer cell lines (A549, MCF-7, LoVo, LoVo/DX) were used to evaluate the anticancer potency of all synthesized compounds.

Compared with unmodified colchicine, the majority of its studied derivatives exhibited excellent potency against A549, MCF-7, and LoVo cell lines. The antiproliferative activity of colchicine derivatives was in the nanomolar range and they also were characterized by lower IC_50_ values also than doxorubicin and cisplatin, commonly used as antitumor agents in cancer chemotherapy. The preliminary SAR revealed that the type of substituent at position C7 in ring B and the presence of –NHCH_3_ group at position C10 in ring C of colchicine **1** were of cardinal importance to the compounds’ cytotoxicity.

The calculated values of the selectivity index (SI) clearly show that it is possible to design more selective compounds than colchicine **1**. The most active double-modified colchicine derivatives were the compounds that included chloroacetamide and dichloroacetamide (**9**, **10**) and 2–chlorobenzamide (**13**) moiety (Table 2). However, in vitro tests showed that they did not have high SI values (except towards the LoVo cells) and *in vivo* testing should be carried out to determine their therapeutic potential. But of all tested compounds, 4,4,4-trifluorobutyric amide of 10-*N*-methylaminocolchicine **11** was the most prominent. Compound **11** showed high activity towards A549, MCF7 and LoVo cell lines, with antiproliferative IC_50_ values ranging between 10.4 and 14.6 nM as well as SI values higher than 5 for these three cell lines. The most selective towards MCF-7 cell line was compound **17** (SI was almost 10) and towards LoVo cell line the most selective were compounds **7**, **9**, **10**, and **13** (SI were over 6). So we conclude that the appropriate modification of colchicine molecule and synthesis of its analogs might overcome the toxicity, which is a major challenge in designing a potential colchicine-based drug candidate.

Compound **11** was subsequently identified as the that with potentially the best therapeutic index and therefore the most promising candidate for further development, as it showed strong in vitro anticancer activities with high SI against three human cancer cell lines. However, further in vivo studies should be conducted for the successful development of this compound.

Molecular docking study showed the ability of the obtained colchicine derivatives to bind into the active sites of βI-tubulin isotype with the well-defined binding modes. It is worthy to mention that 10-*N*-methylaminocolchicine based compounds serve as useful templates for the further development of the anticancer and antimitotic agents. Further evaluation should help to find more detailed structure-activity relationships of microtubule-targeting drugs and CBS inhibitors, which can help in rational drug design in future.

## Figures and Tables

**Table 1:** Selected torsion angles (°) of colchicine derivatives **6**, **11**, **12**, **14**, **15**, **16**, **18** and **19** obtained by X-ray analysis and DFT computation for a comparison.

		6	11	12	14	15	16	18	19
**C1**–**C16**–**C13**–**C12**	100 K 295 K DFT value	53.1(4) 55.2(4) 53.1	55.5(6) 55.5(6) 52.3	53.7(3) 55.0(3) 53.4	54.0(5) 54.4(4) 53.5	53.6(4) 54.7(4) 52.9	54.1(3) 55.0(3) 53.3	56.1(4) 56.1(4) 54.5	55.3(3) 55.9(4) 56.0
**C17**–**O1**–**C1**–**C2**	100 K 295 K DFT value	−79.0(4) −86.5(4) −73.0	−86.4(6) −86.4(6) −70.7	−89.3(3) −86.4(4) −80.3	−90.6(5) −87.4(5) −80.0	−87.3(4) −87.8(4) −72.9	−86.8(4) −86.4(4) −79.4	−108.6(3) −106.8(3) −74.8	66.2(3) 65.6(4) 59.2
**C18**–**O2**–**C2**–**C3**	100 K 295 K DFT value	108.0(3) 106.9(4) 70.1	105.2(6) 105.2(6) 68.8	96.1(2) 98.7(3) 82.1	95.5(4) 98.0(4) 82.3	99.7(4) 101.4(4) 71.1	94.5(3) 98.7(3) 81.1	−77.5(4) −74.0(4) −59.6	−102.7(3) −100.6(4) −77.2
**C19**–**O3**–**C3**–**C4**	100 K 295 K DFT value	−14.1(5) −8.1(7) −4.0	−10.5(9) −10.5(10) −3.7	8.6(4) 6.9(6) −2.0	14.4(6) 11.4(7) −1.9	−6.0(6) −2.3(6)−3.8	7.4(6) 6.9(6) −1.9	1.2(5) 0.2(5) 0.7	7.7(4) 6.8(5) 3.1
**C20**–**N1**–**C10**–**C11**	100 K 295 K DFT value	7.7(5) 6.8(6) −0.9	8.8(8) 8.7(9) −0.9	4.6(3) 7.5(4) −1.4	4.4(6) 5.0(6) −1.0	5.0(5) 5.2(5) −1.2	6.6(4) 7.5(4) −1.3	−0.2(5) −1.7(5) 0.2	4.7(4) 4.0(7) −1.6

**Table 2:** Antiproliferative activity (IC_50_) of colchicine (**1**) and its derivatives (**2**–**21**) compared with antiproliferative activity of standard anticancer drugs doxorubicin and cisplatin and the calculated values of the resistance index (RI) of tested compounds.

Compound	A549	MCF-7	LoVo	LoVo/DX		BALB/3T3
	IC_50_ [nM]	IC_50_ [nM]	IC_50_ [nM]	IC_50_ [nM]	RI	IC_50_ [nM]
**1**	115.3 ± 23.6	22.6 ± 1.3	17.5 ± 2.5	1646.6 ± 314.0	93.9	115.3 ± 36.8
**2**	10.8 ± 1.3	8.6 ± 1.3	4.3 ± 1.3	271.3 ± 99.9	63.0	10.8 ± 1.3
**3**	16.9 ± 2.8	19.7 ± 1.7	14.0 ± 1.7	129.2 ± 11.8	9.2	19.7 ± 7.0
**4**	14.6 ± 2.4	14.6 ± 1.5	9.7 ± 1.5	271.8 ± 104.4	28.0	19.4 ± 4.1
**5**	14.1 ± 2.4	14.1 ± 1.4	9.6 ± 0.5	194.8 ± 51.9	20.2	16.4 ± 3.5
**6**	13.6 ± 1.4	15.9 ± 6.6	6.8 ± 3.9	102.3 ± 20.7	15.0	13.6 ± 2.3
**7**	613.8 ± 194.4	464.8 ± 186.7	62.4 ± 16.9	2435.7 ± 923.4	39.0	545.9 ± 104.4
**8**	11.7 ± 1.4	18.8 ± 9.9	7.0 ± 1.4	171.2 ± 41.3	24.3	28.1 ± 10.8
**9**	11.6 ± 2.8	9.2 ± 1.4	1.8 ± 0.4	62.4 ± 6.7	35.2	11.6 ± 2.3
**10**	8.6 ± 1.3	8.6 ± 1.2	1.5 ± 0.5	38.5 ± 21.6	25.8	10.7 ± 1.3
**11**	14.6 ± 2.1	12.7 ± 0.4	10.4 ± 1.3	289.6 ± 165.2	27.8	81.3 ± 20.4
**12**	13.0 ± 1.3	13.0 ± 1.3	8.5 ± 0.4	99.9 ± 10.0	11.8	13.0 ± 3.3
**13**	6.3 ± 3.2	9.2 ± 0.8	0.7 ± 0.1	9.6 ± 3.3	14.0	6.2 ± 1.6
**14**	10.7 ± 0.6	12.6 ± 1.3	8.6 ± 0.8	102.5 ± 24.9	12.0	12.6 ± 2.1
**15**	36.8 ± 12.1	13.0 ± 2.2	10.8 ± 1.3	832.1 ± 292.7	76.8	43.3 ± 29.7
**16**	17.3 ± 3.7	12.8 ± 0.9	10.8 ± 1.3	946.9 ± 260.5	87.4	52.0 ± 29.3
**17**	56.6 ± 14.7	10.3 ± 3.9	21.1 ± 17.5	6466.0 ± 264.2	306.0	100.1 ± 24.1
**18**	10.6 ± 0.6	15.5 ± 1.7	9.3 ± 1.1	540.2 ± 107.2	57.8	14.2 ± 13.2
**19**	11.4 ± 1.7	13.0 ± 4.3	8.4 ± 0.7	306.7 ± 144.9	36.7	8.3 ± 3.8
**20**	800.3 ± 130.0	150.0 ± 25.3	268.4 ± 94.0	44385.7 ± 23852.0	165.4	991.1 ± 280.5
**21**	85.5 ± 5.4	134.3 ± 41.5	73.5 ± 15.7	6122.0 ± 825.1	83.3	87.6 ± 13.5
Doxorubicin	141.7 ± 46.0	204.2 ± 47.8	99.4 ± 41.0	8732.0 ± 2540.7	87.9	149.0 ±126.8
Cisplatin	5741.0 ± 968.0	7139.8 ± 1218.7	7076.3 ± 1596.2	8336.5 ± 1119.2	1.2	5665.1 ± 31.8

The IC_50_ value is defined as the concentration of a compound at which 50% growth inhibition is observed. The IC_50_ values shown are mean ± SD. Human lung adenocarcinoma (A549), human breast adenocarcinoma (MCF-7), human colon adenocarcinoma cell line (LoVo) and doxorubicin-resistant subline (LoVo/DX), normal murine embryonic fibroblast cell line (BALB/3T3). The RI (Resistance Index) indicates how many times more chemoresistant is a resistant subline relative to its parental cell line. The RI was calculated for each compound using the formula: RI = (IC_50_ for LoVo/DX cell line)/(IC_50_ for LoVo cell line). When RI is 0–2, the cells are sensitive to the compound tested, RI in the range 2–10 means that the cells shows moderate sensitivity to the drug tested, RI above 10 indicates strong drug resistance.

**Table 3:** Computational predictions of interactions between tested compounds (**1**–**21**) and homology modeled tubulin βI. 3D representation and 2D layout of colchicine derivatives–tubulin protein complex, binding energy (BE), calculated octanol/water partition coefficient (clog*P*) and active residues are tabulated.

Compound	3D Representation of the Interactions	2D Representation of the Interactions	Binding Energy [kcal/mol]	clog*P*	Active Residues
**1**	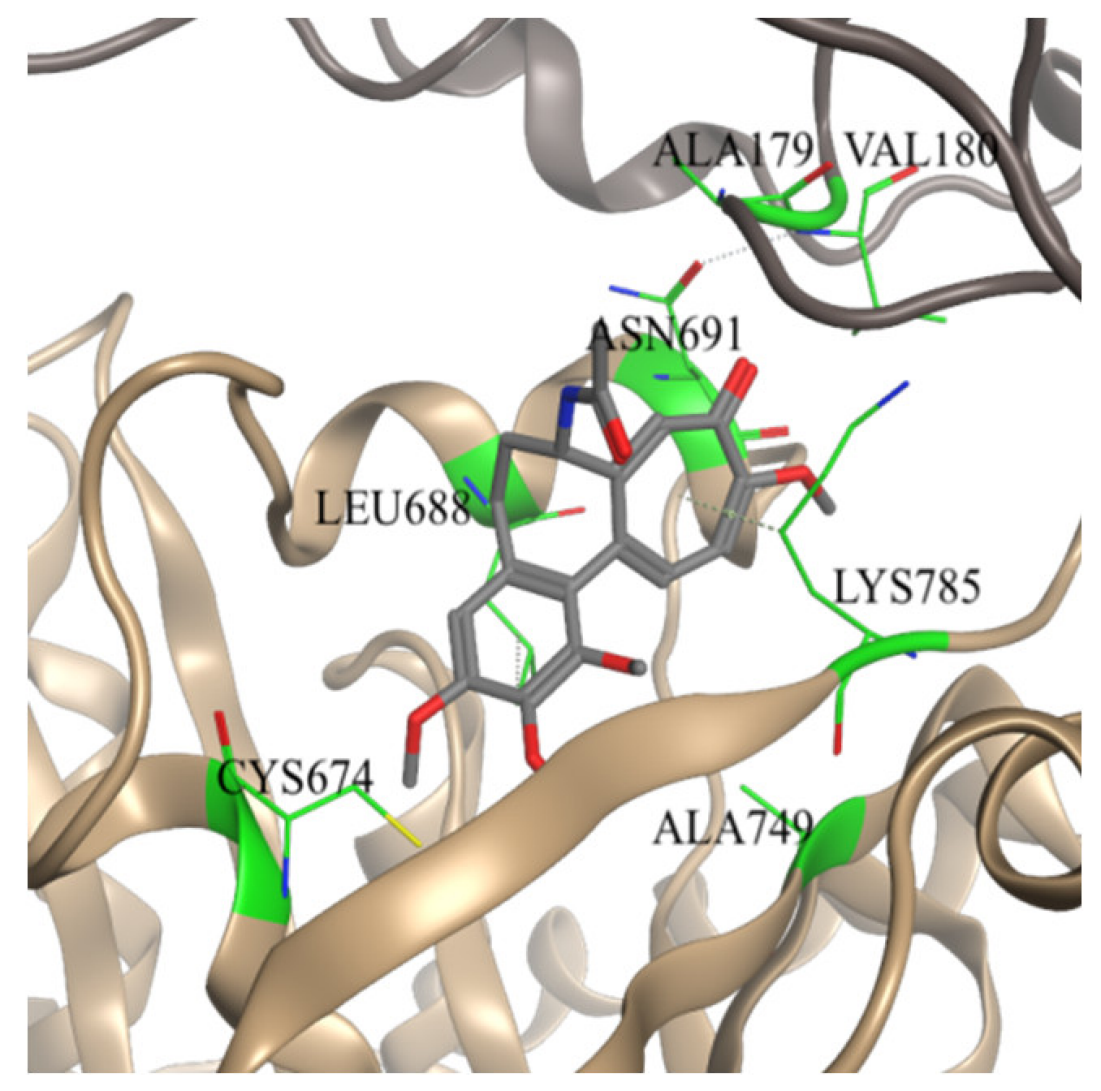	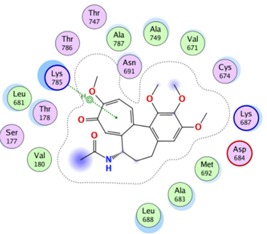	−41.0	1.1	Ala179 Val180 **Cys674** **Leu688** Asn691 Ala749 **Lys785**
**2**	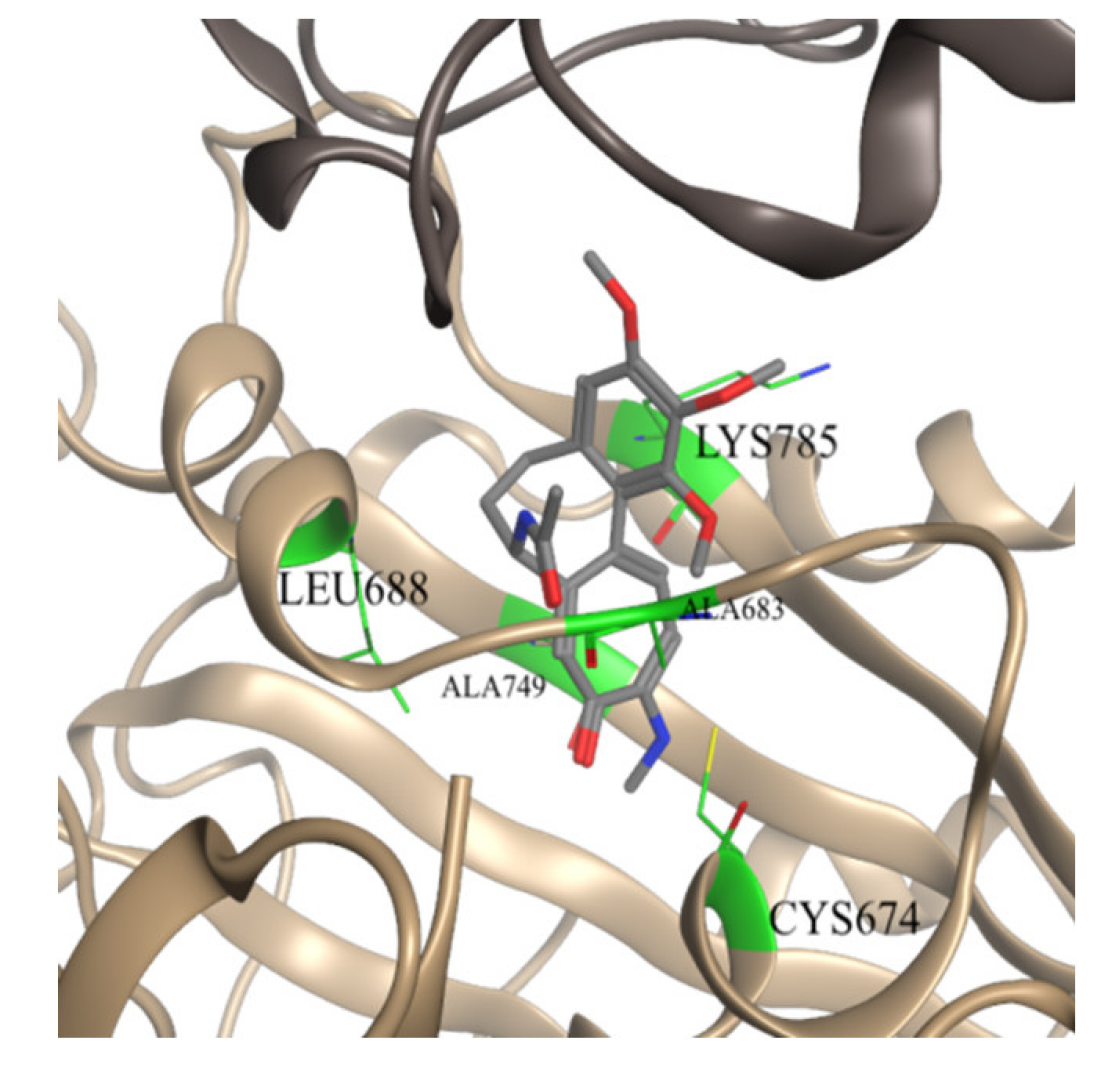	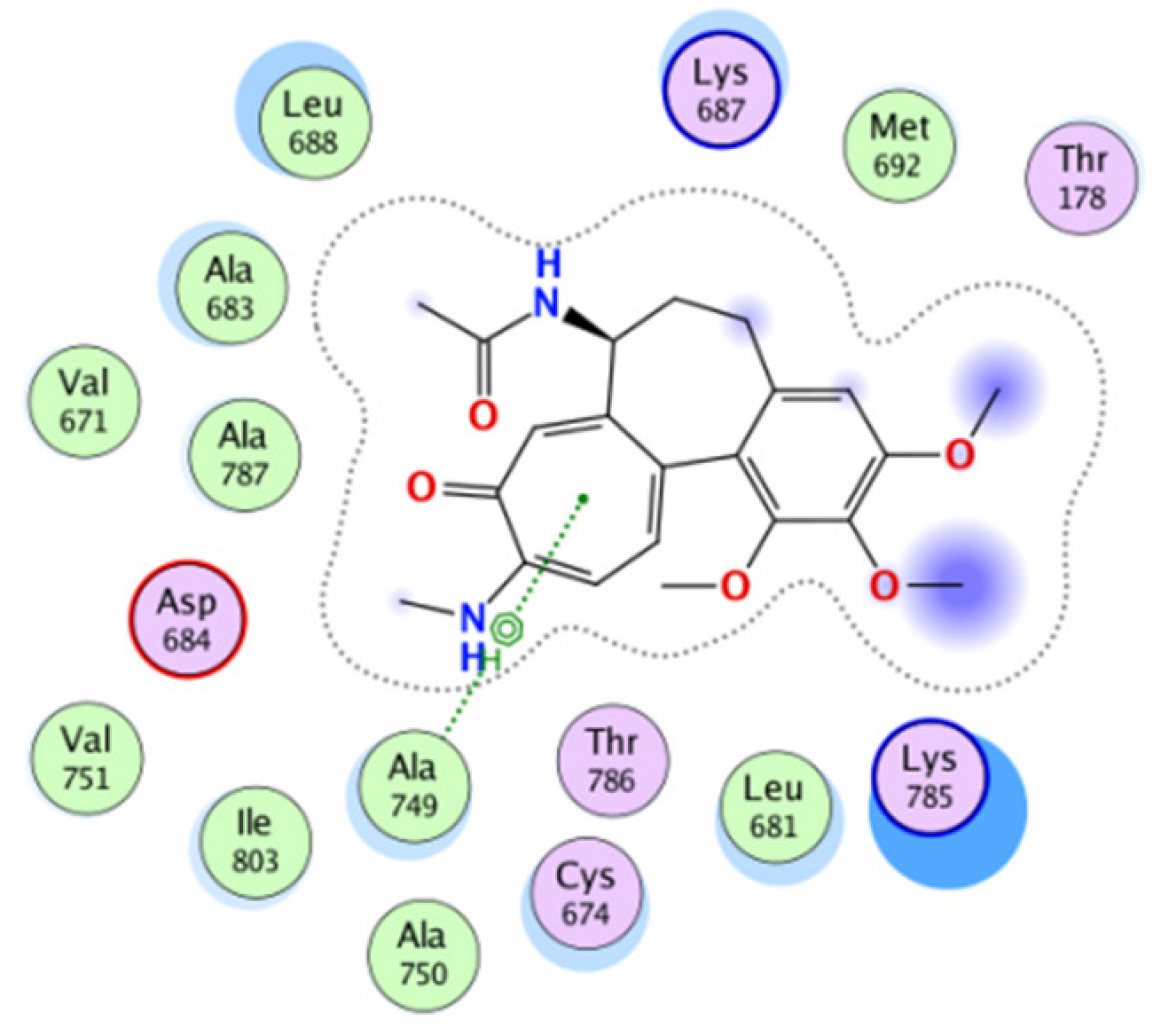	−39.3	1.6	Cys674 Ala683 **Leu688** **Lys785**
**3**	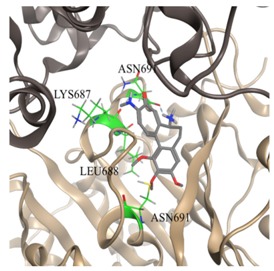	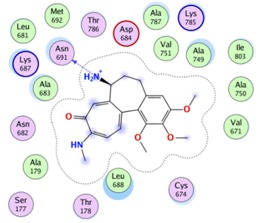	−4.0	0.9	Cys674 Lys687 **Leu688** **Asn691**
**4**	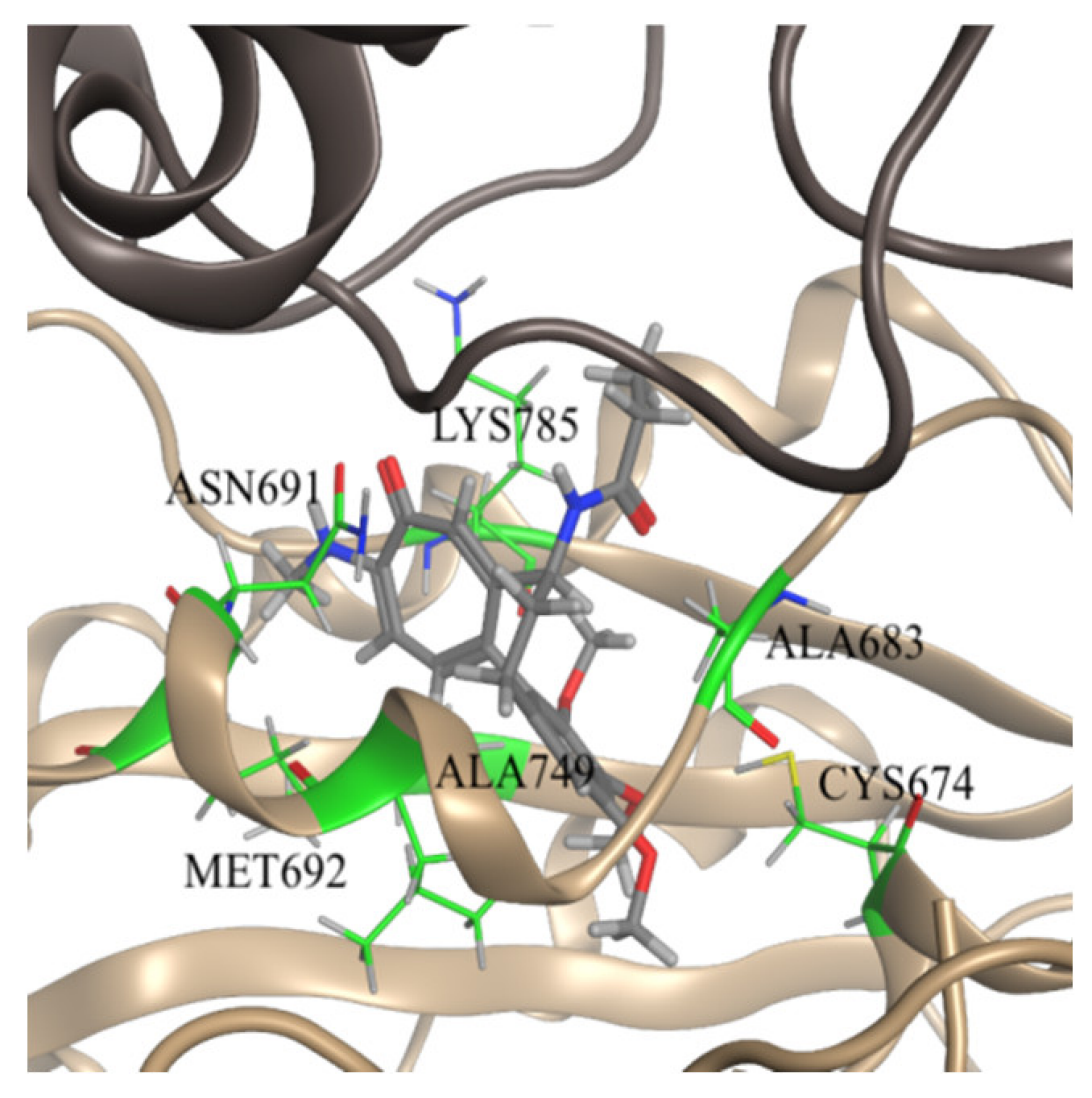	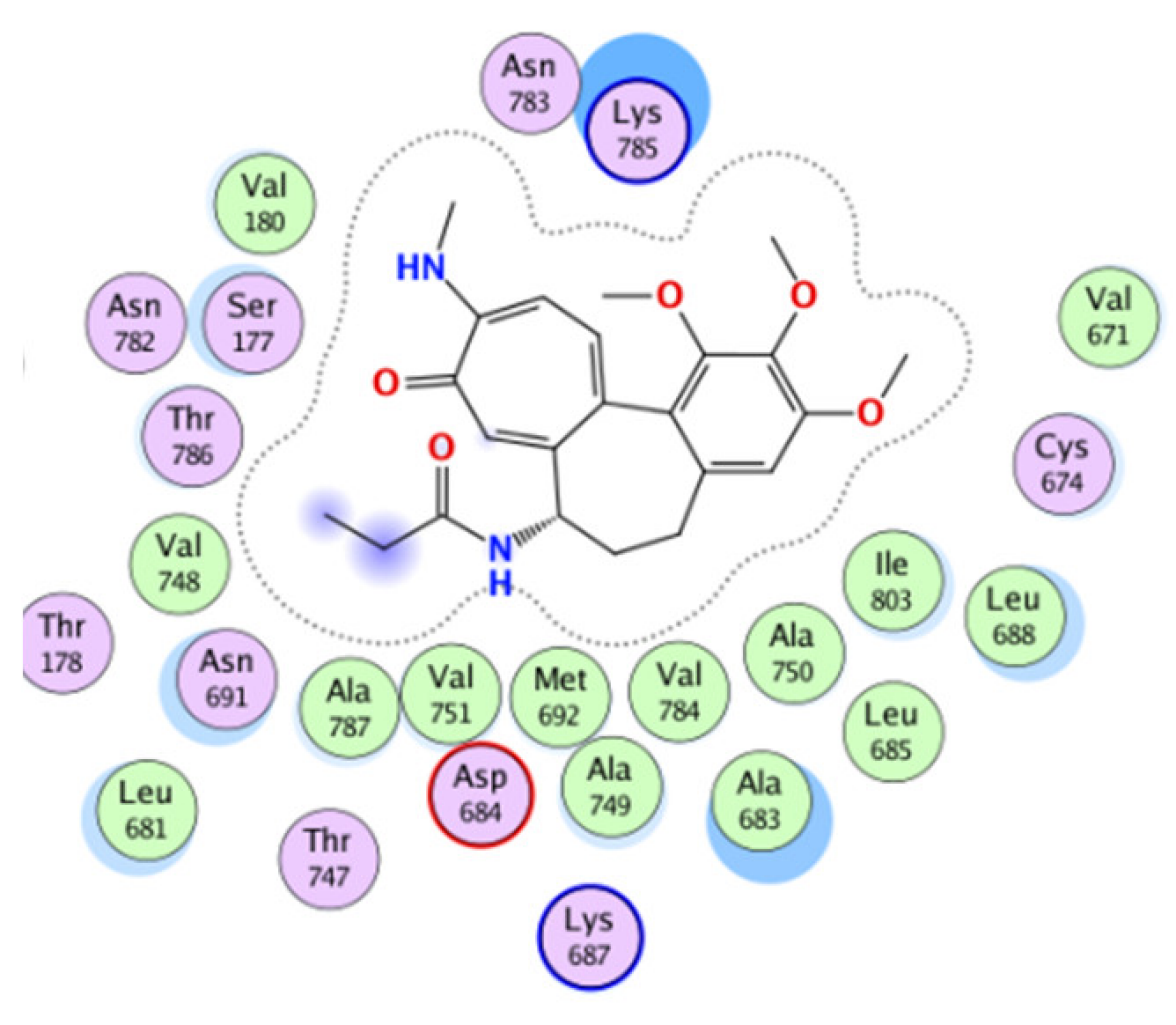	−43.4	1.9	Cys674 Ala683 **Leu688** Asn691 Met692 Ala749 **Lys785**
**5**	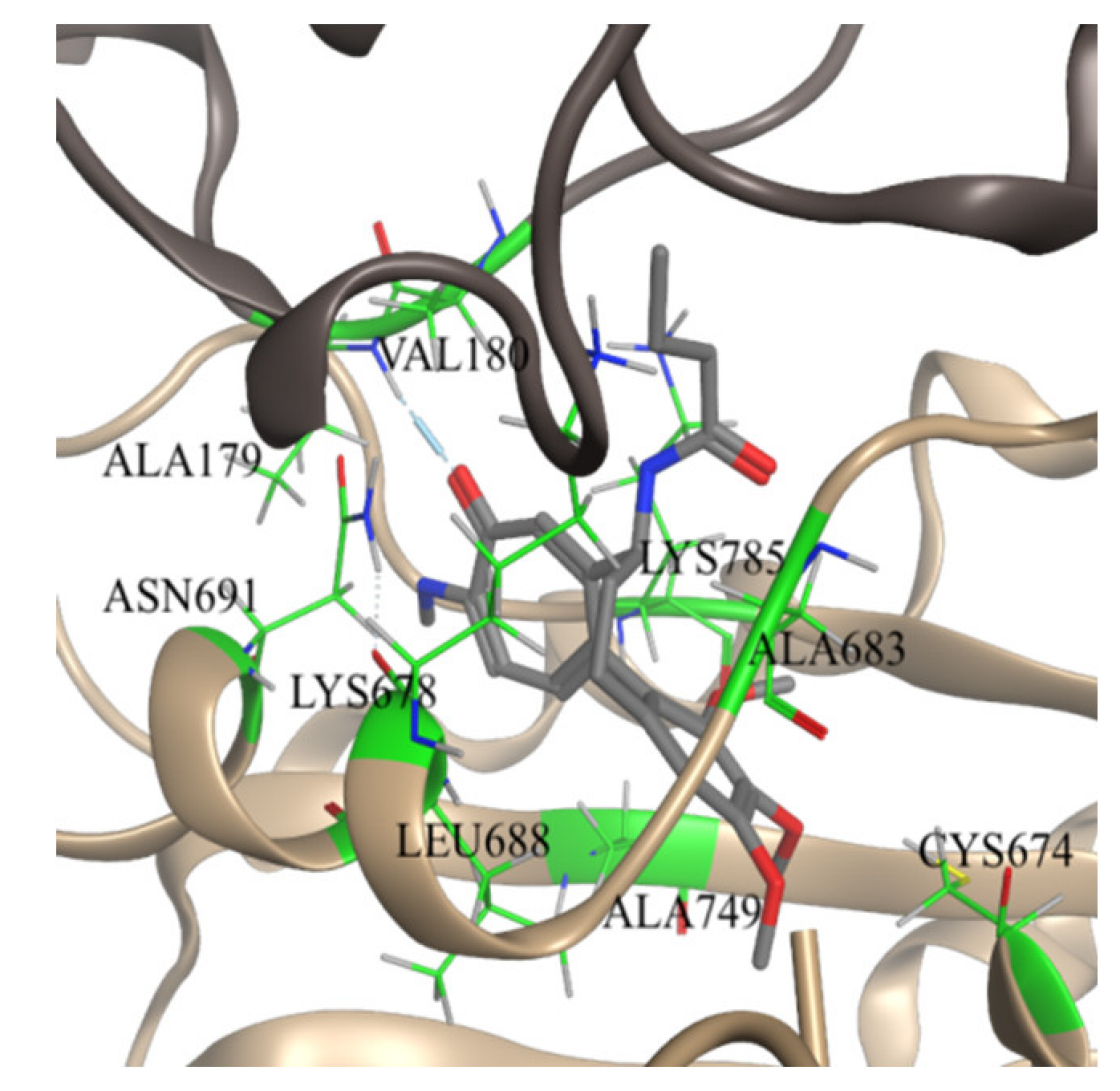	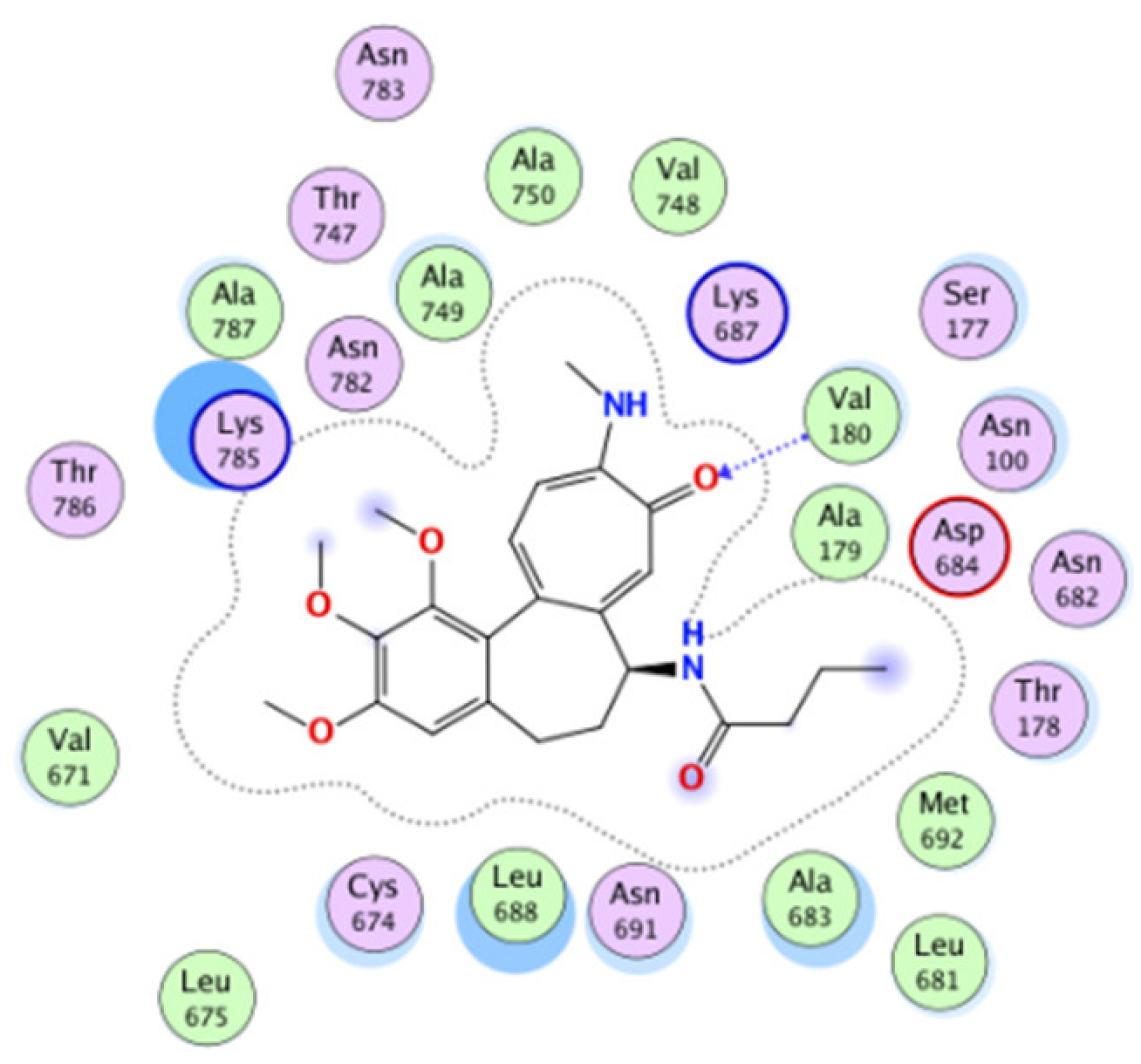	−43.0	2.5	Ala179 Val180 **Cys674** **Leu688** Asn691 Ala749 **Lys785**
**6**	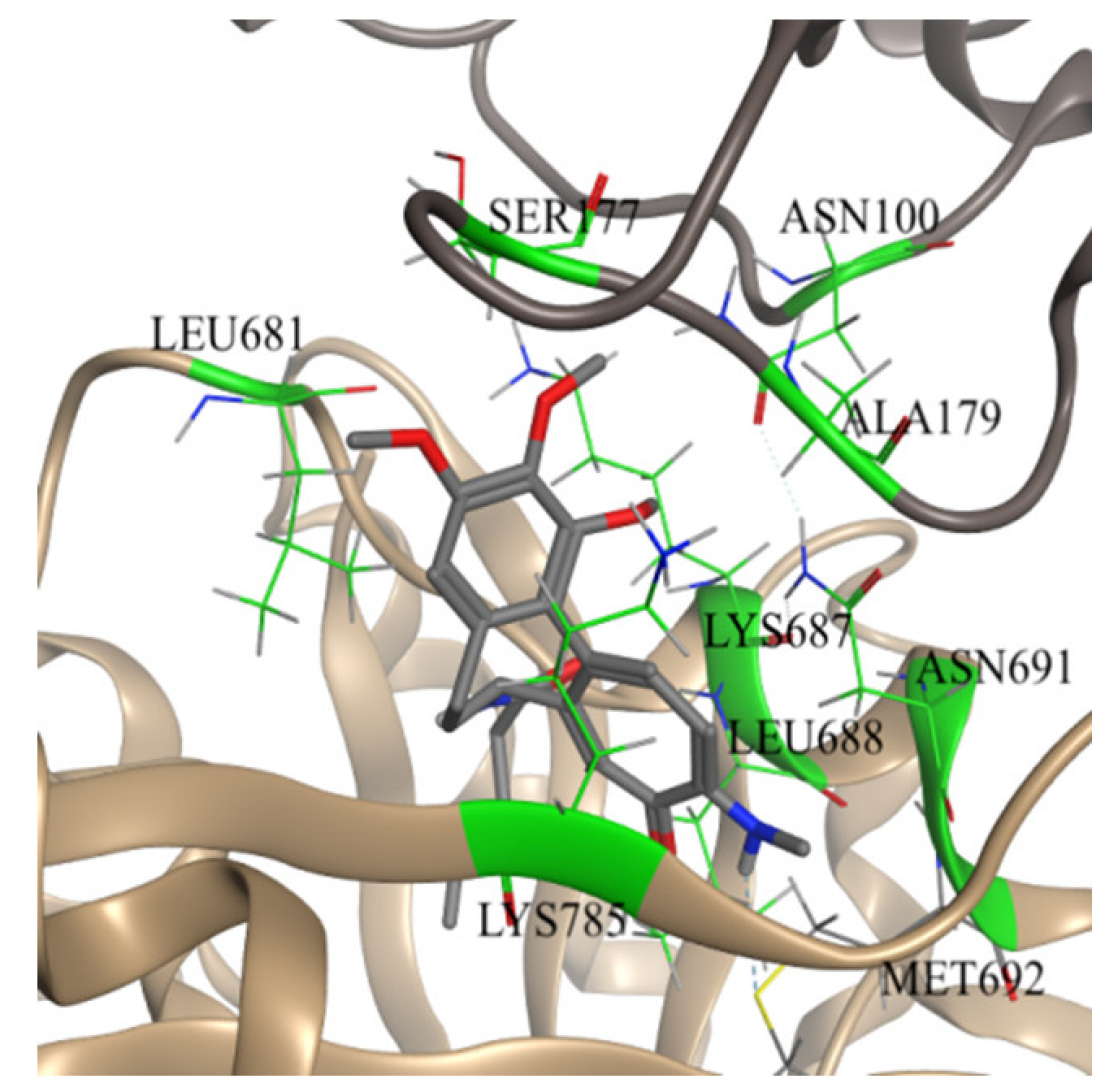	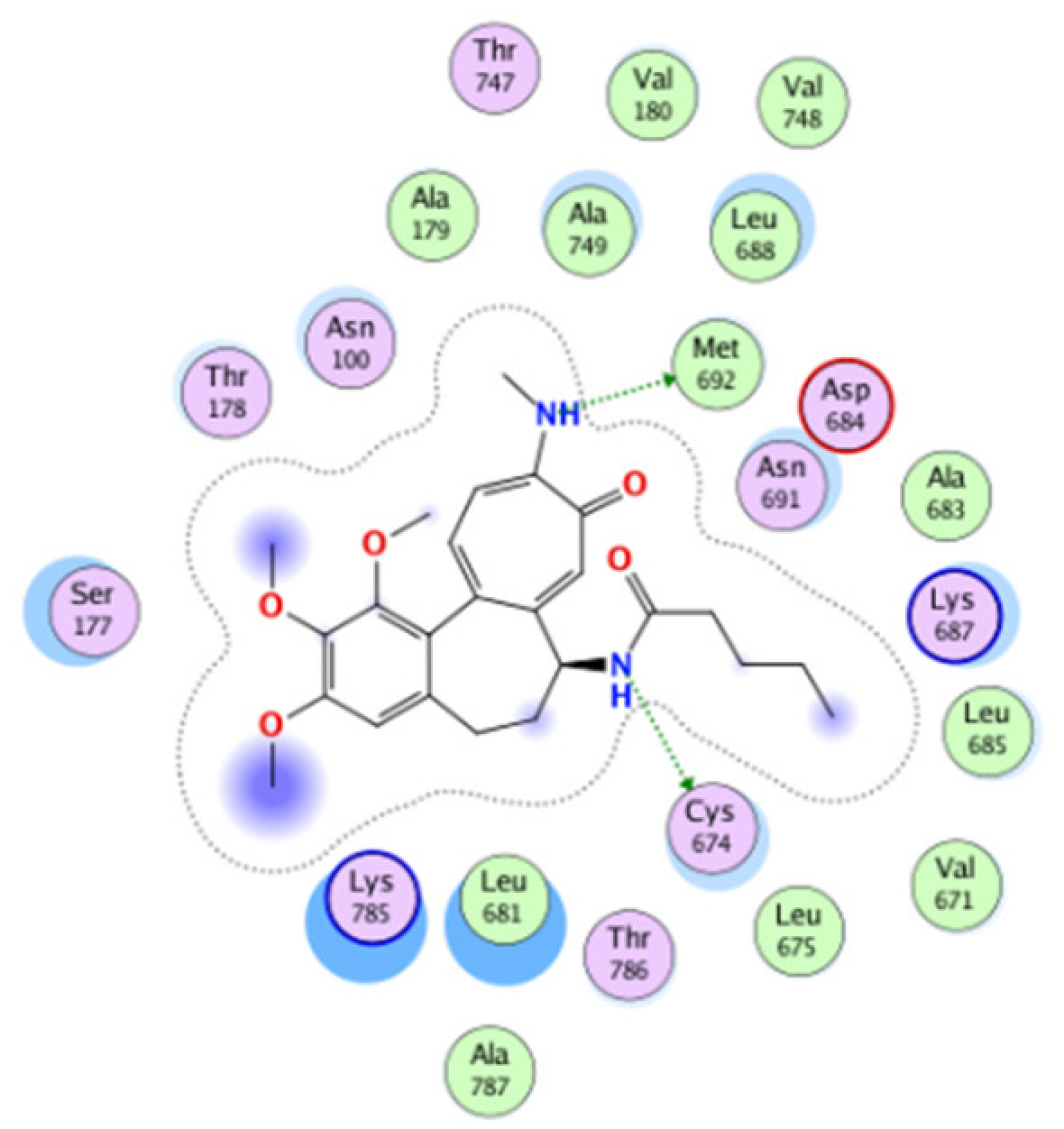	−37.2	3.0	Asn100 Ser177 Ala179 Leu681 **Lys687** Leu688 Asn691 **Lys785**
**7**	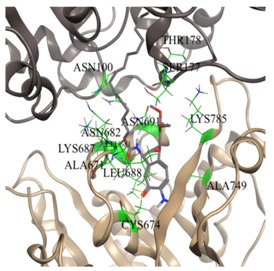	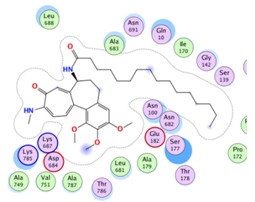	−53.7	8.7	Gln10 Asn100 Ser177 Thr178 Cys674 Leu681 Asn682 Ala683 **Lys687** **Leu688** Asn691 Ala749 **Lys785**
**8**	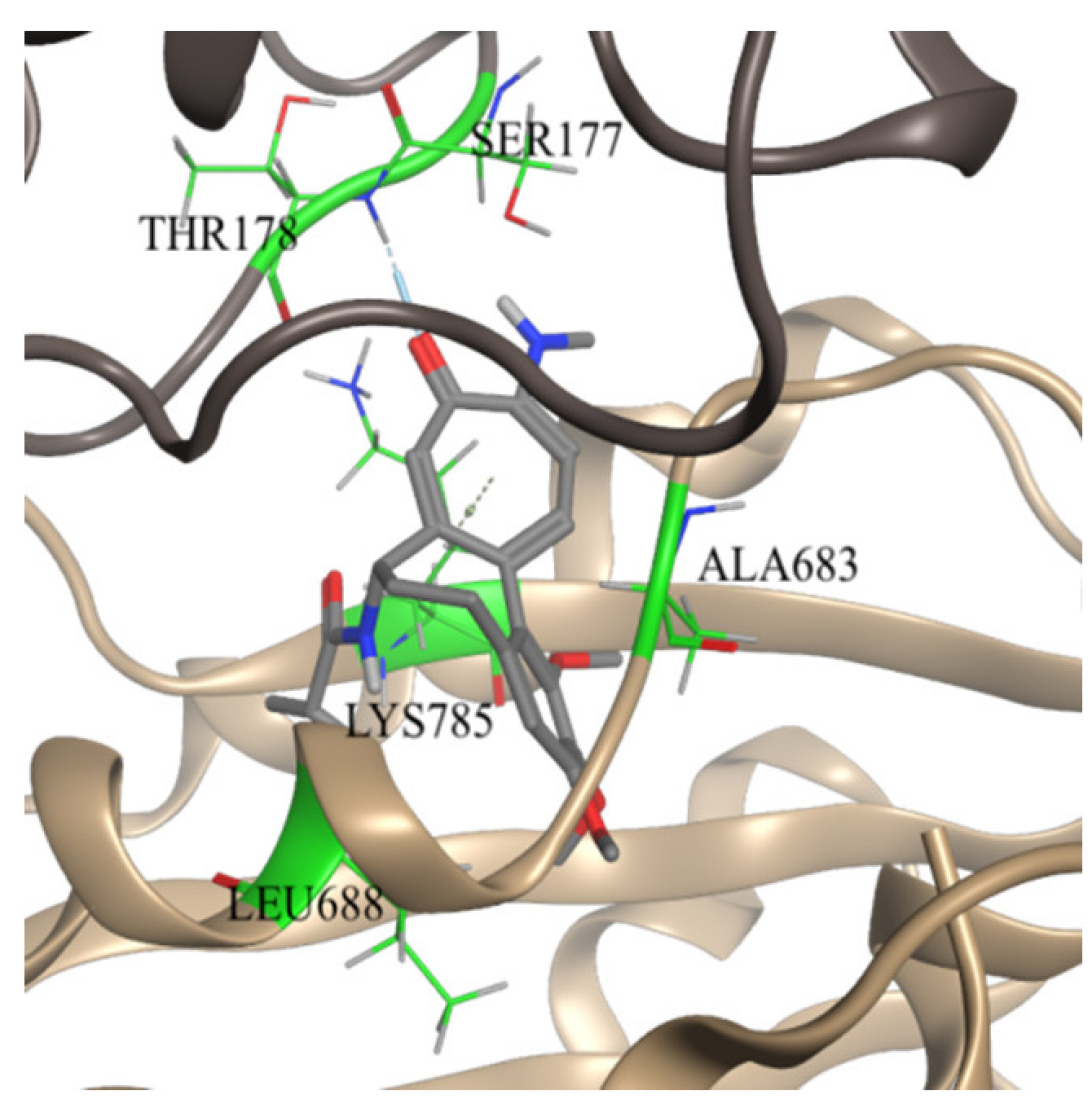	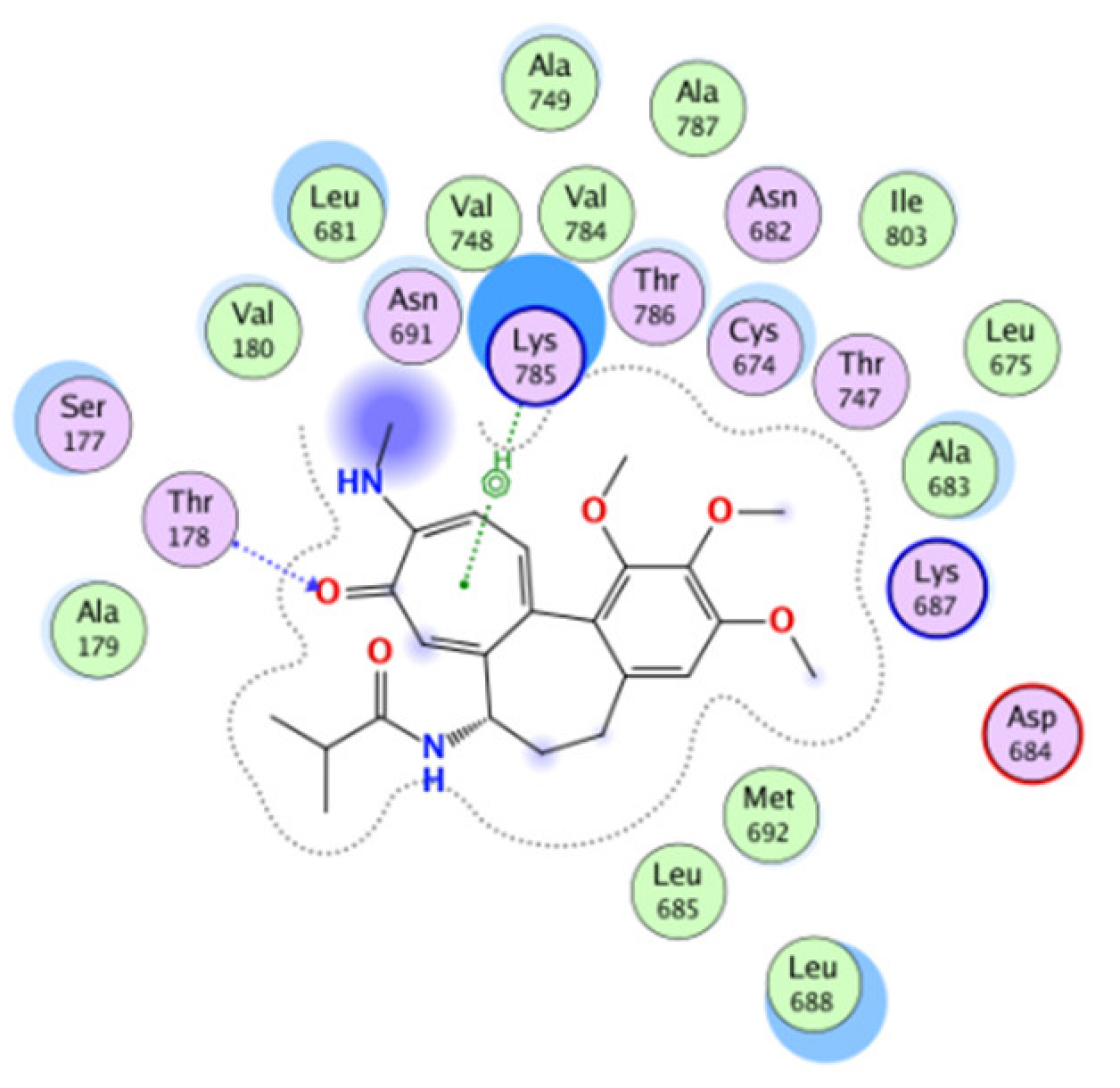	−32.3	2.7	Ser177 Leu681 Ala683 **Leu688** **Lys785**
**9**	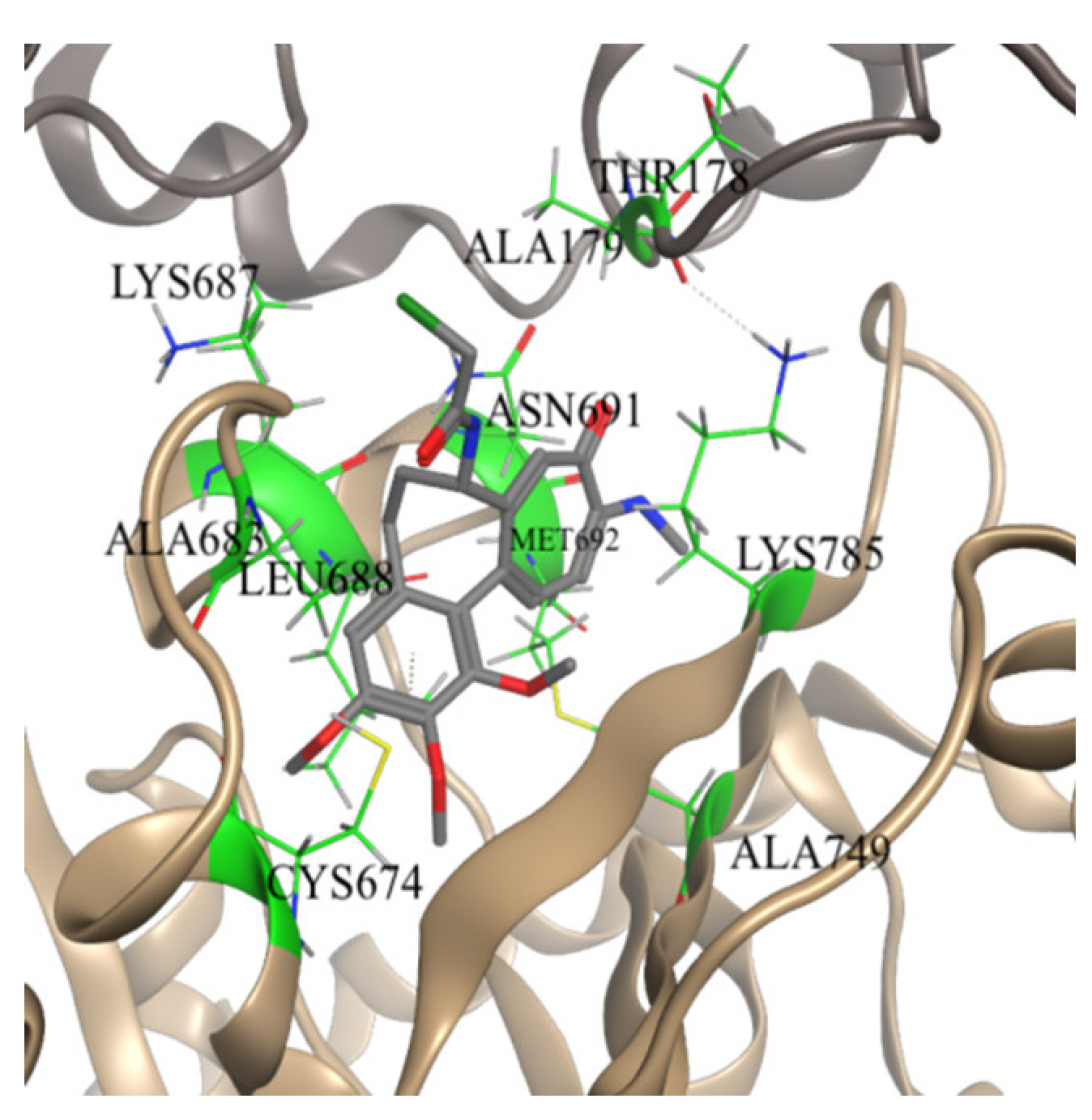	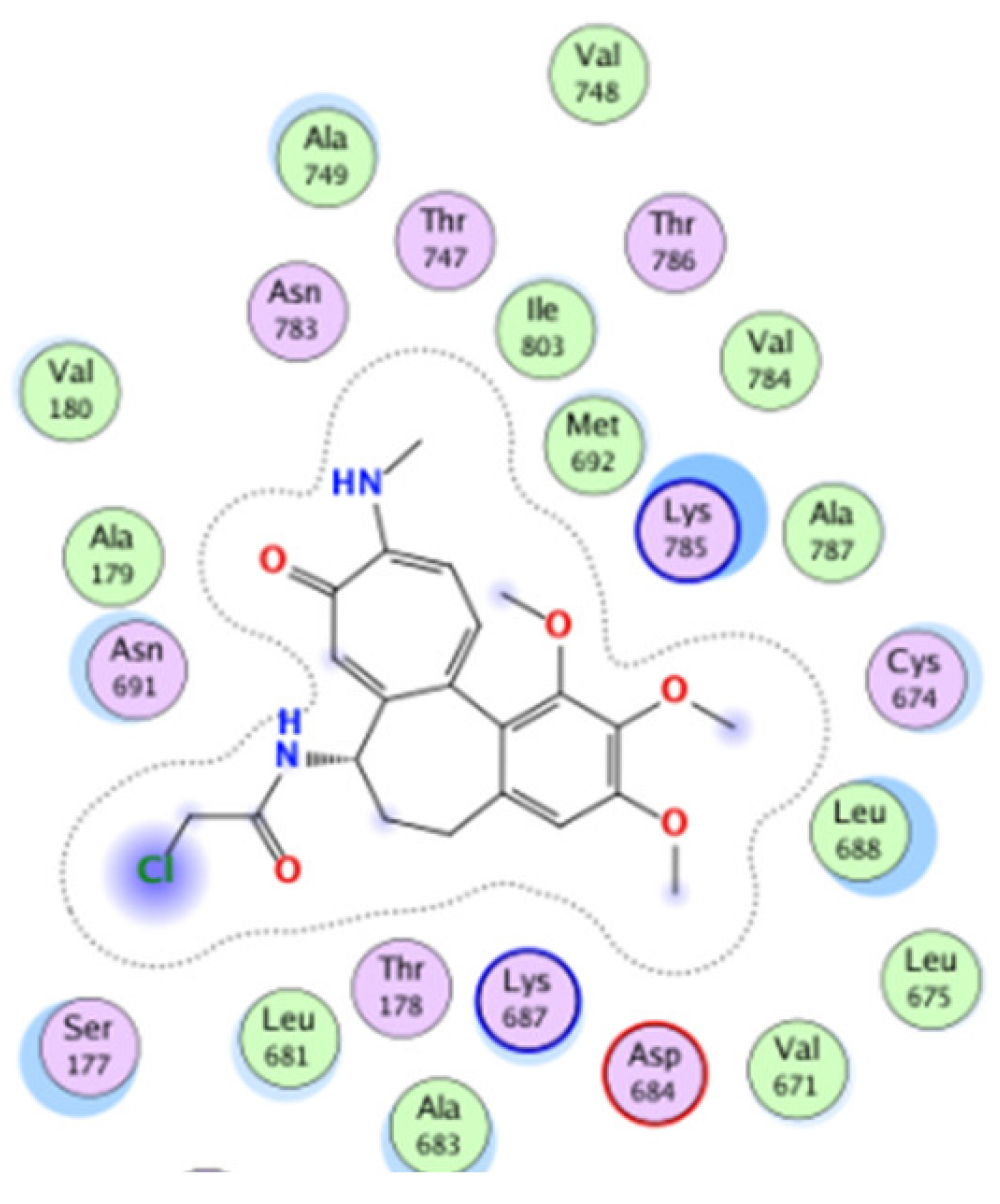	−44.0	2.2	Ala179 Cys674 Ala683 Lys687 **Leu688** Asn691 Met693 Ala749 **Lys785**
**10**	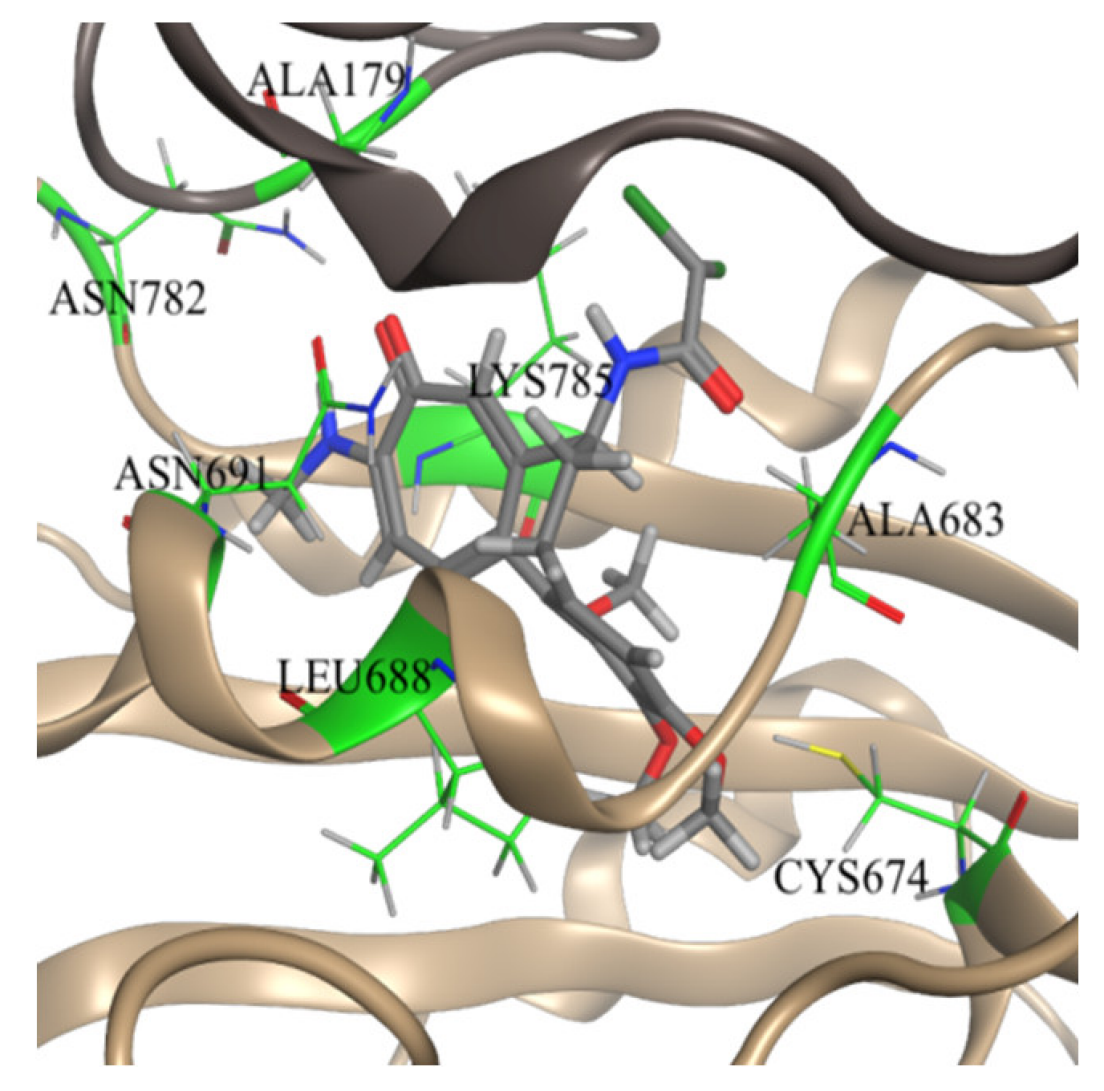	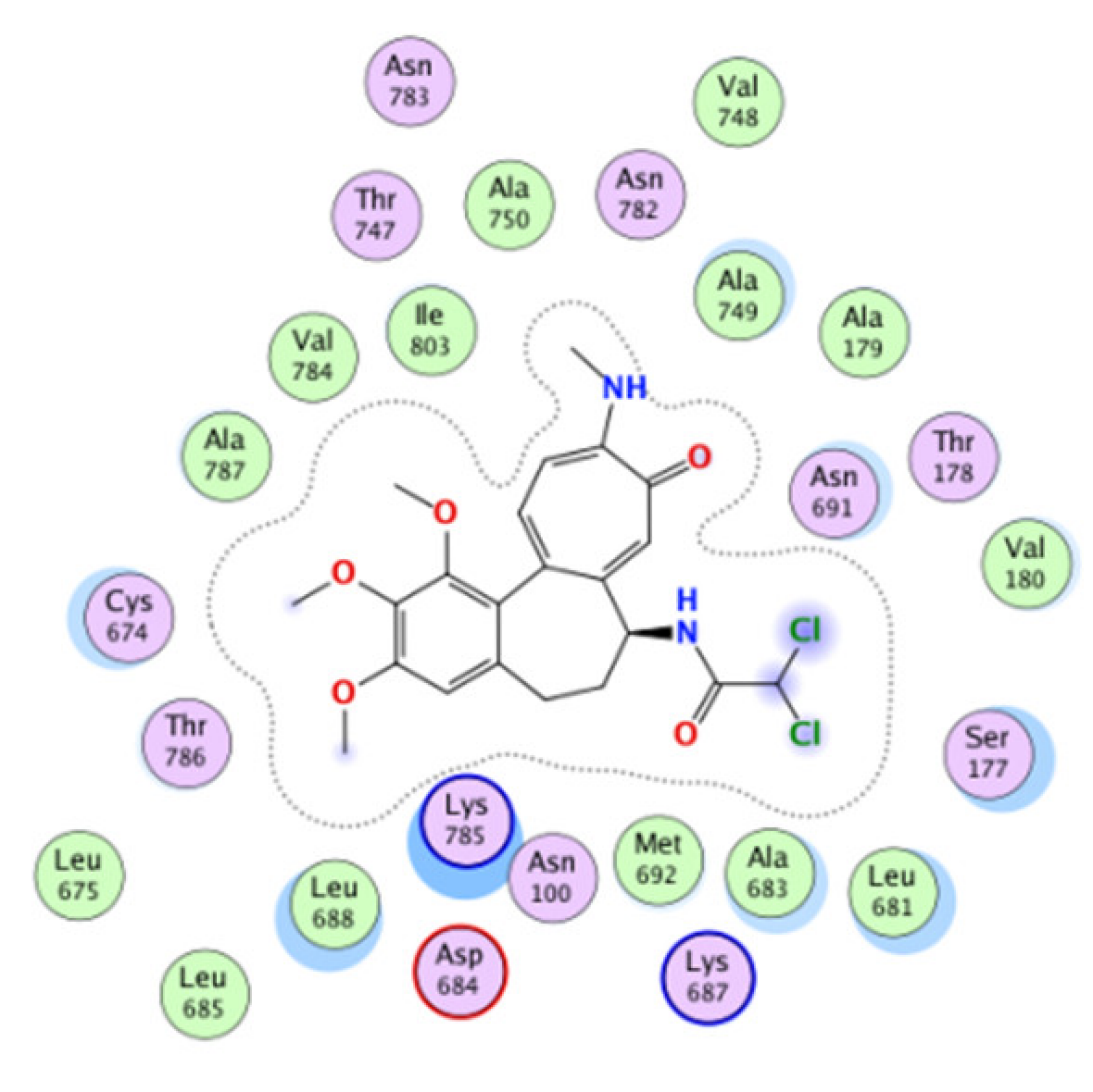	−41.8	2.7	Ala179 Cys674 Ala683 **Leu688** Asn691 Asn782 **Lys785**
**11**	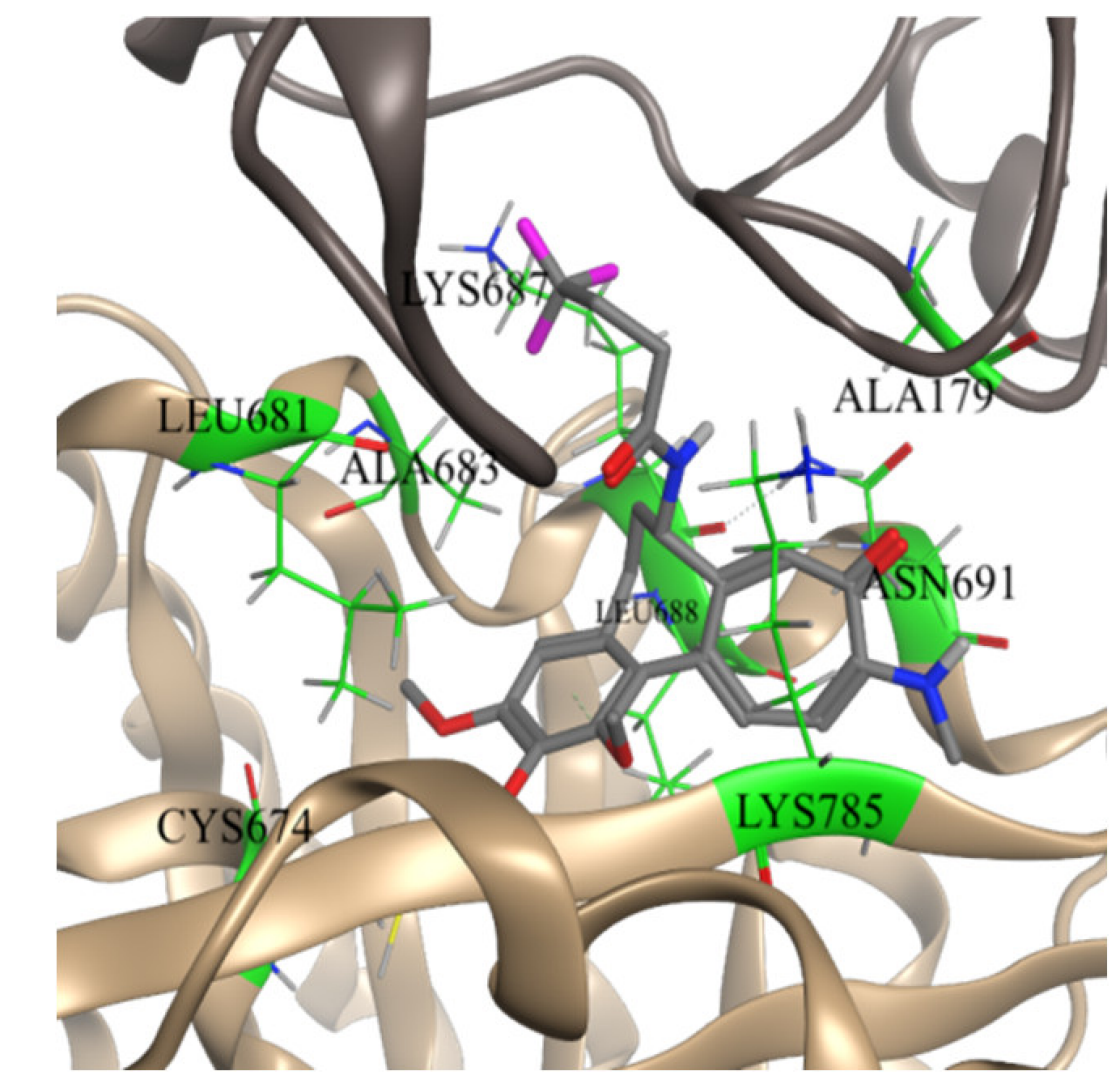	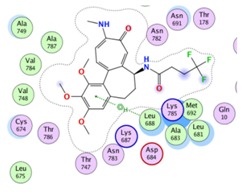	−34.7	3.0	Ala179 Cys674 Leu681 Ala683 Lys687 **Leu688** Asn691 **Lys785**
**12**	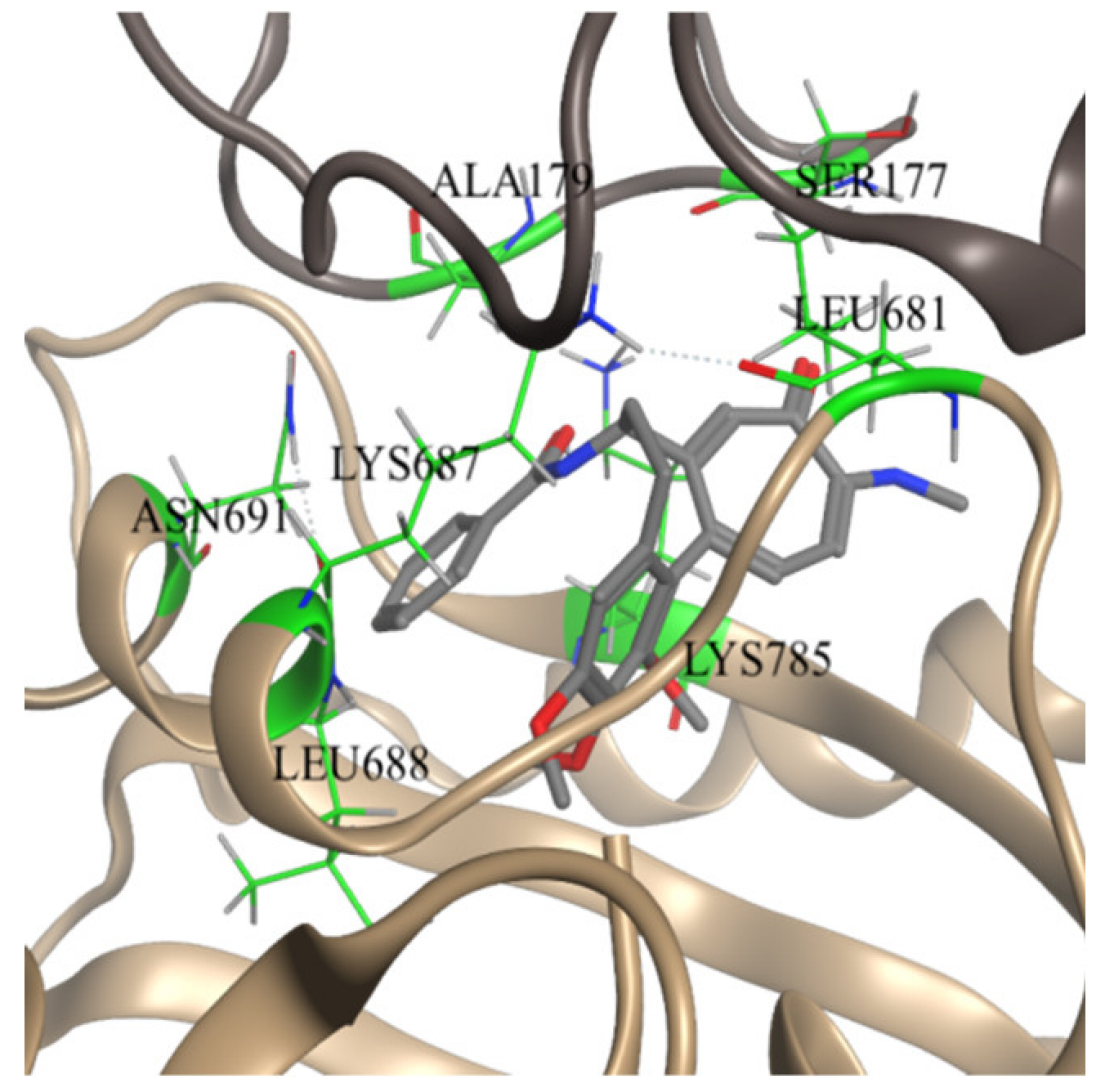	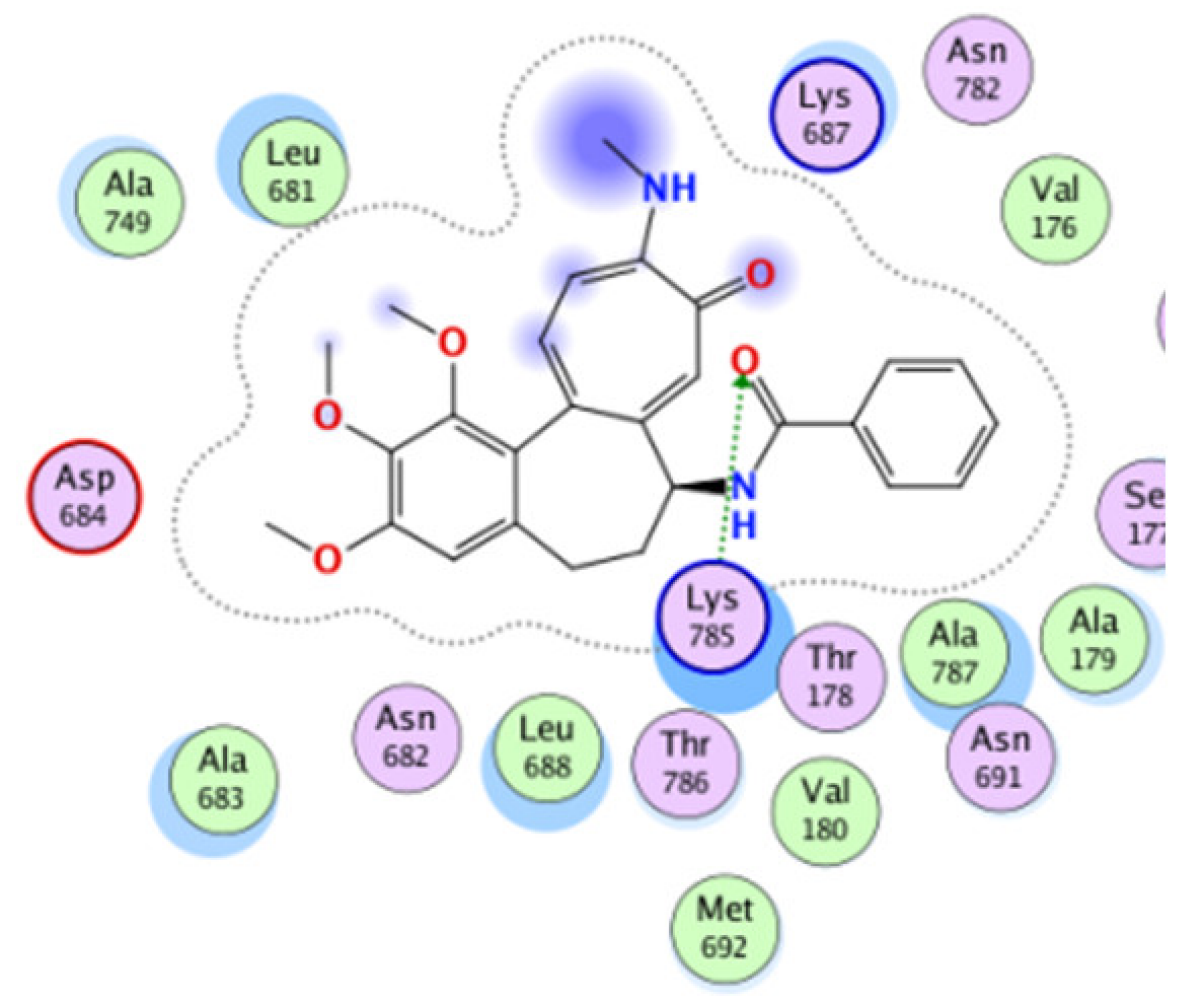	−35.2	3.3	Ser177 Ala179 Leu681 Lys687 **Leu688** Asn691 **Lys785**
**13**	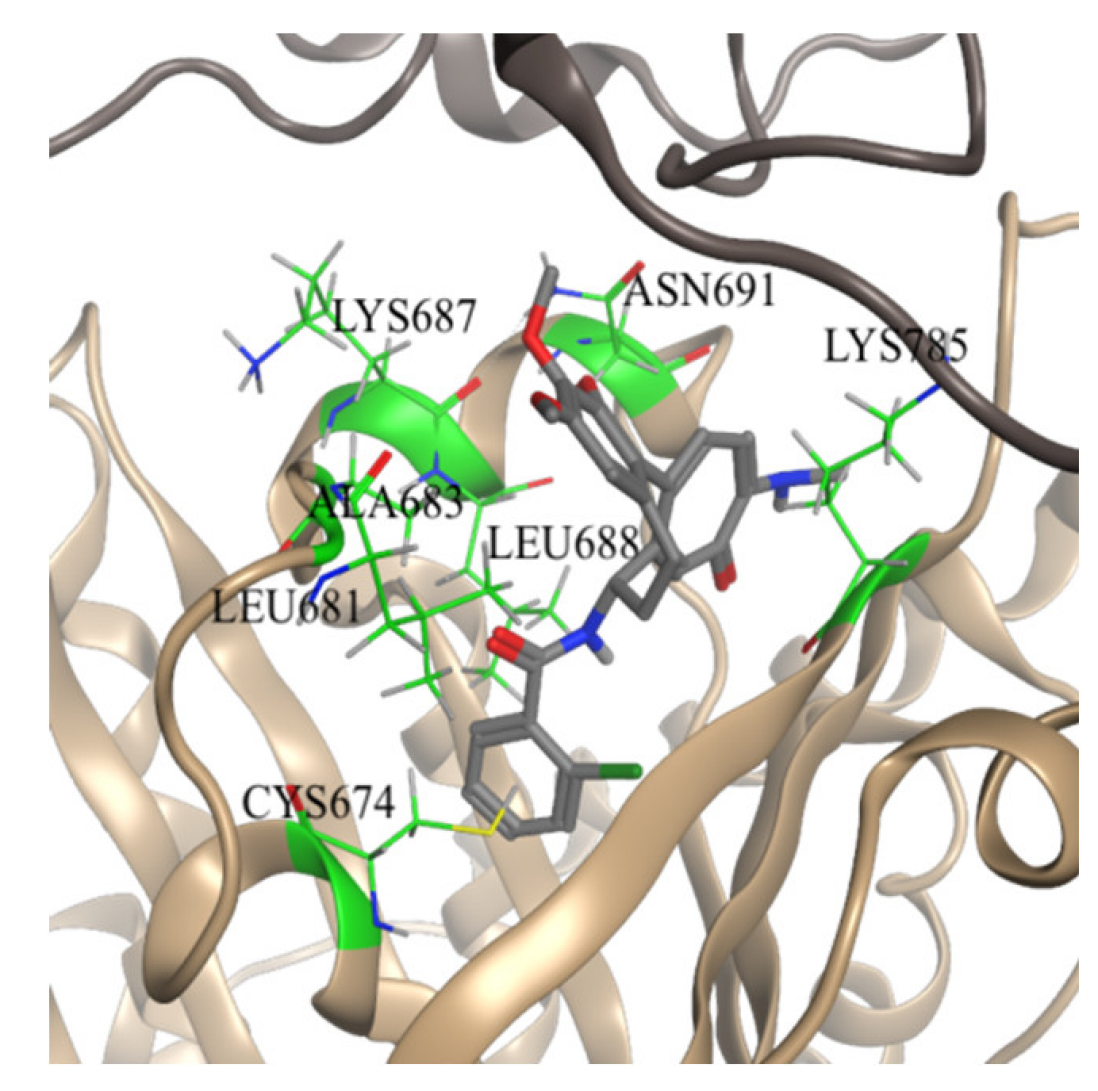	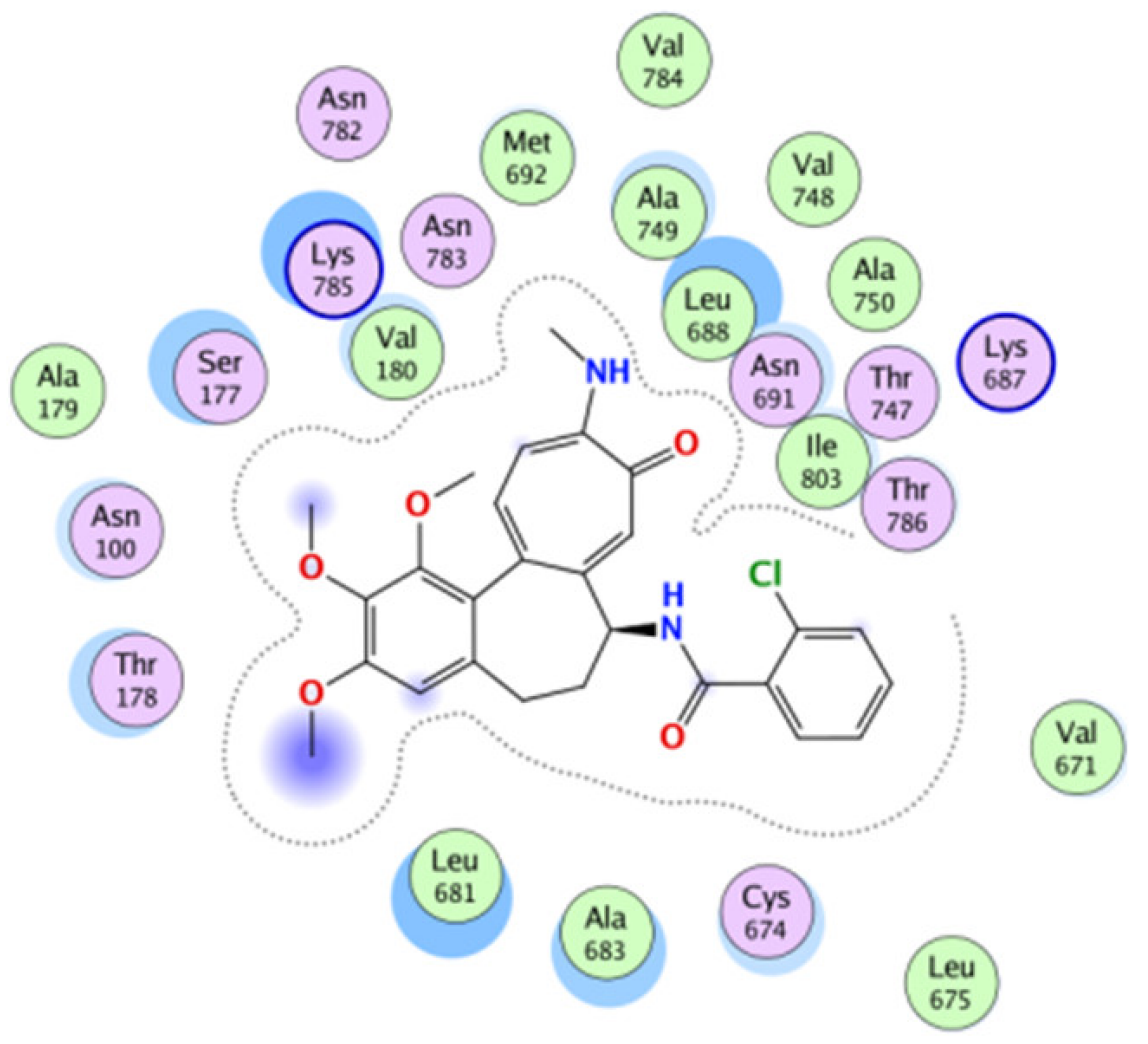	−40.1	3.9	Cys674 Leu681 Ala683 **Lys687** **Leu688** Asn691 **Lys785**
**14**	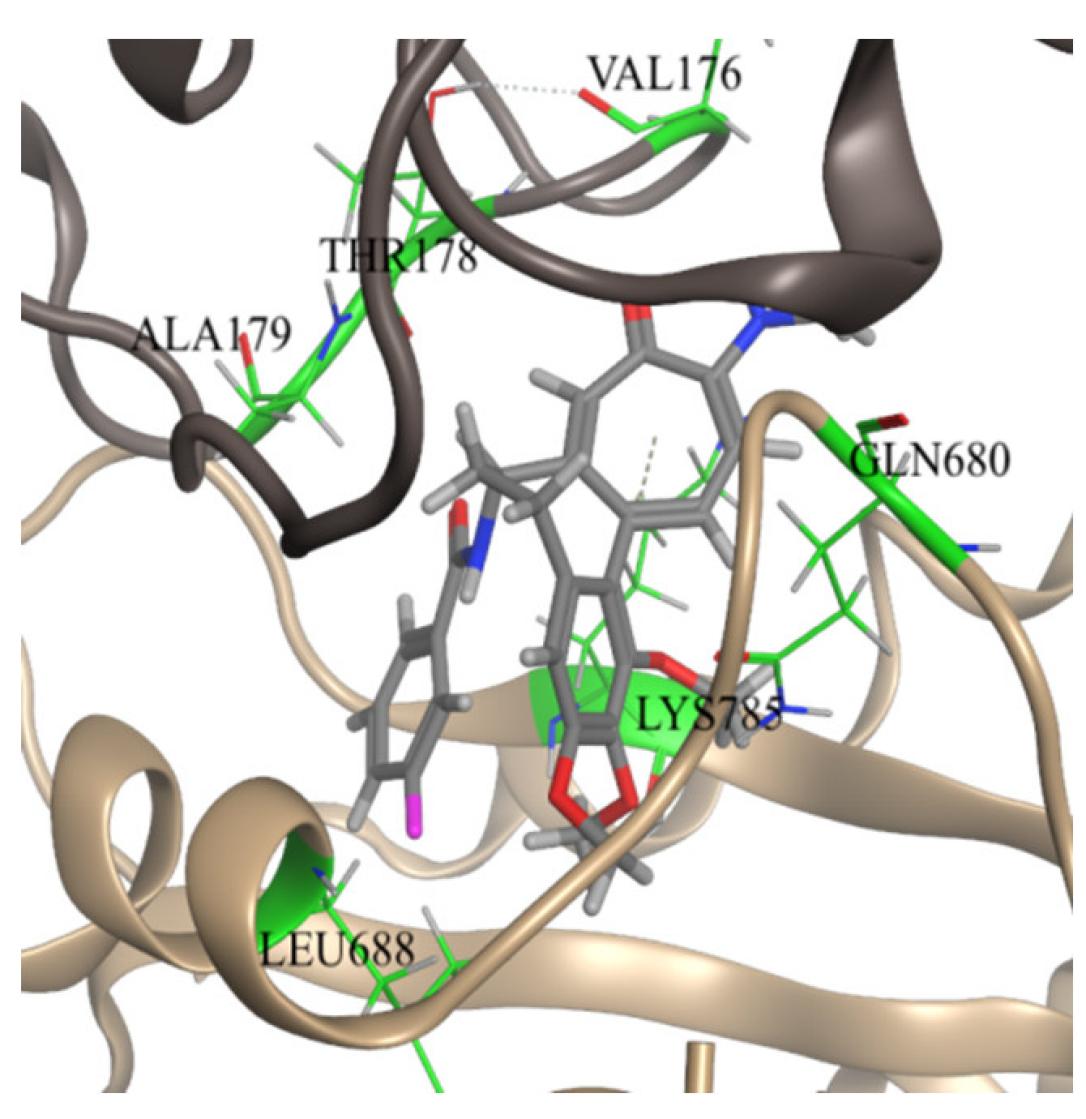	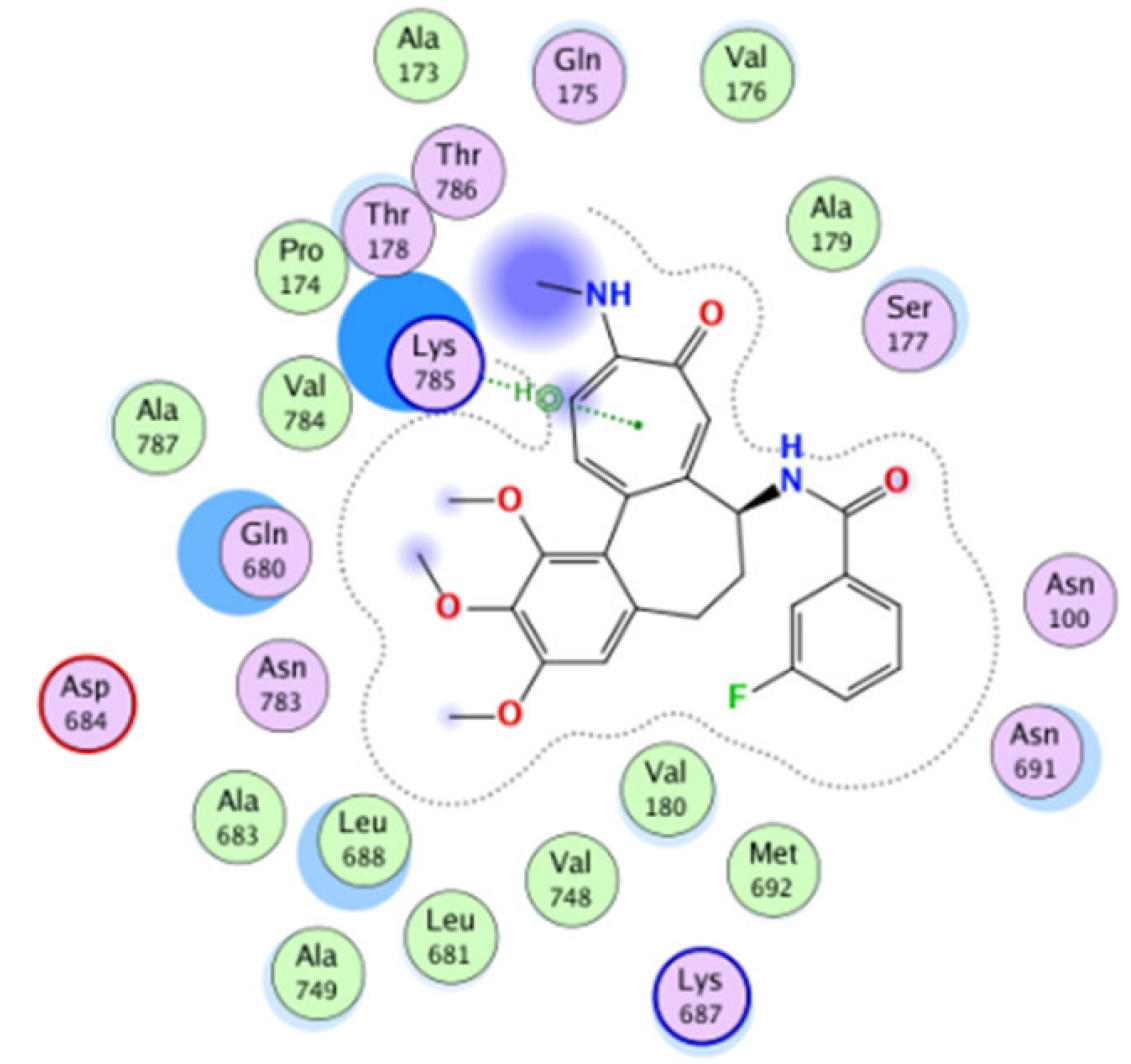	−34.3	3.4	Val176 Ser177 Thr178 Ala179 **Gln680** Leu688 **Lys785**
**15**	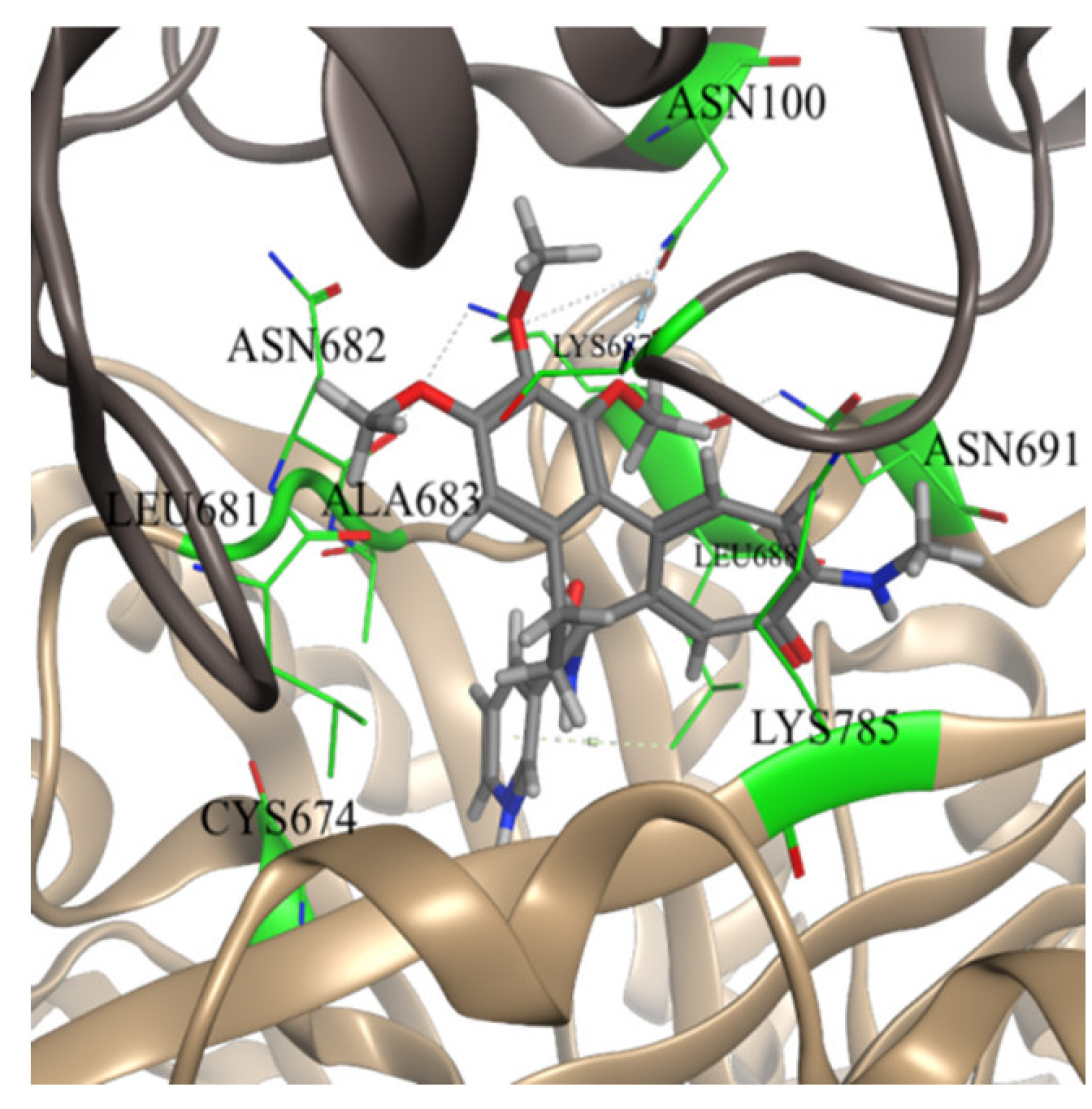	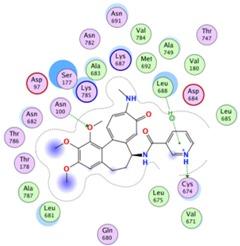	−43.9	2.0	**Asn100** **Ser177** Cys674 **Leu681** Asn682 Ala683 **Lys687** **Leu688** Asn691 **Lys785**
**16**	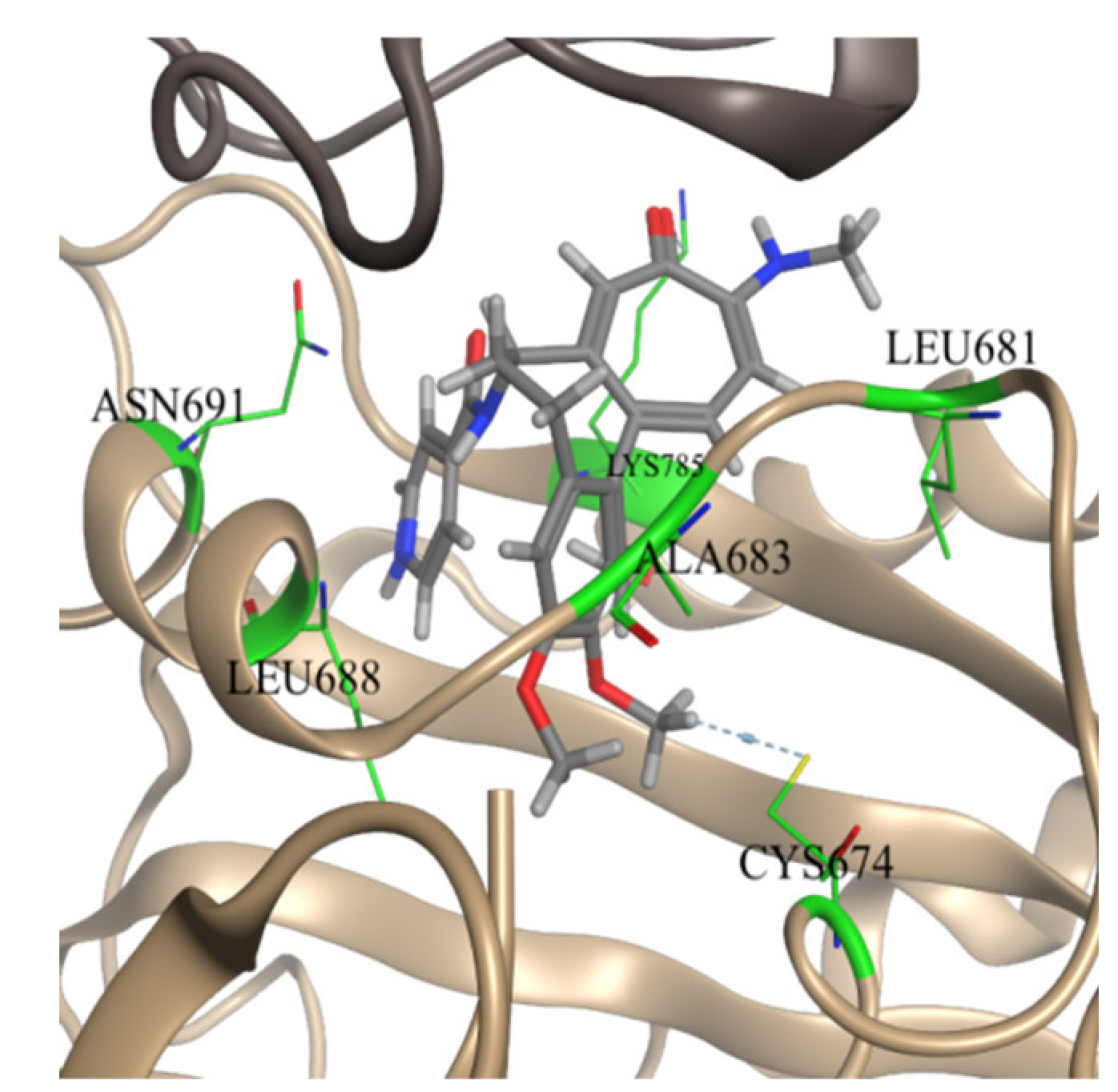	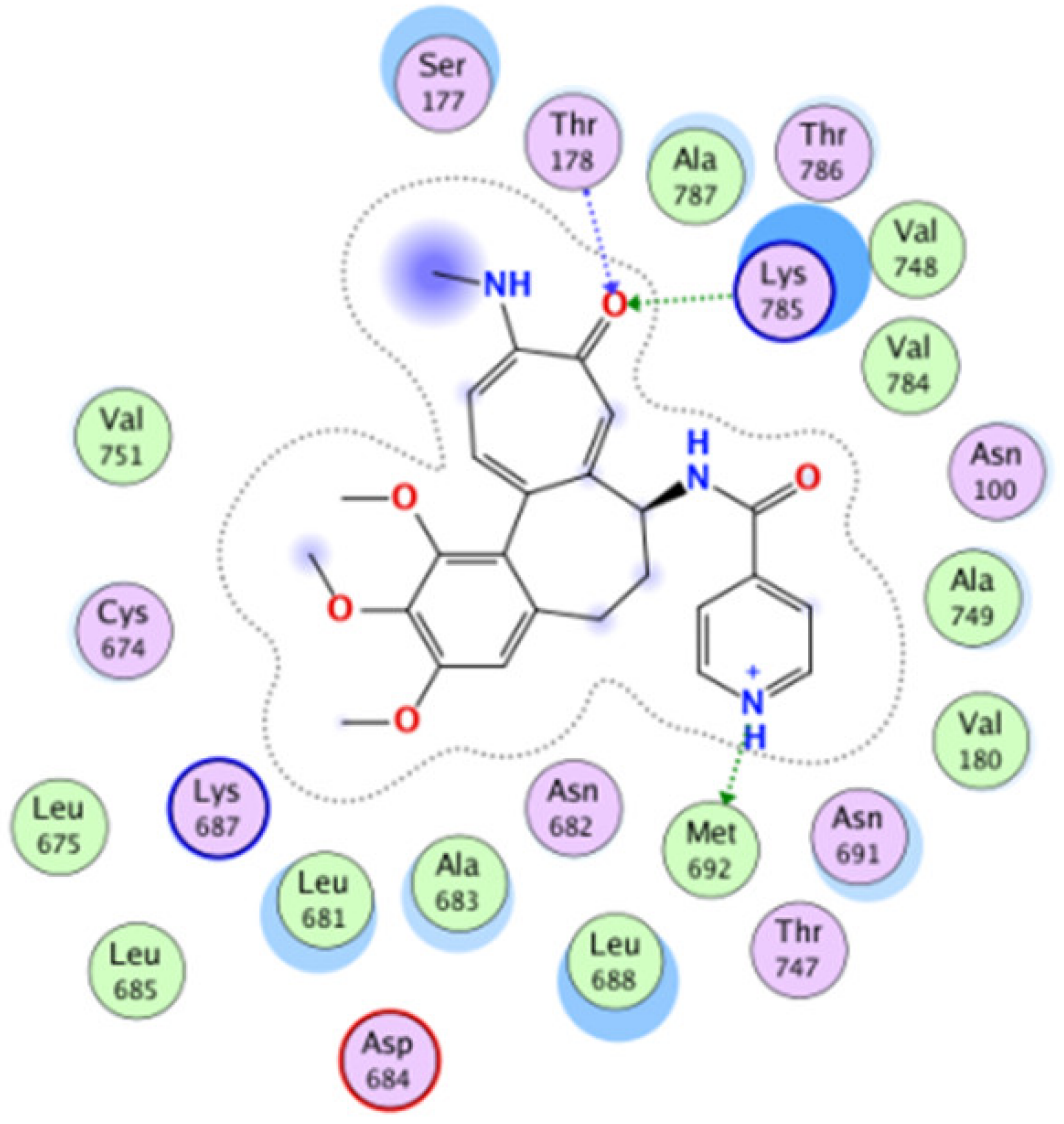	−38.3	2.0	Ser177 Thr178 Cys674 Leu681 Ala683 **Leu688** Asn691 **Lys785**
**17**	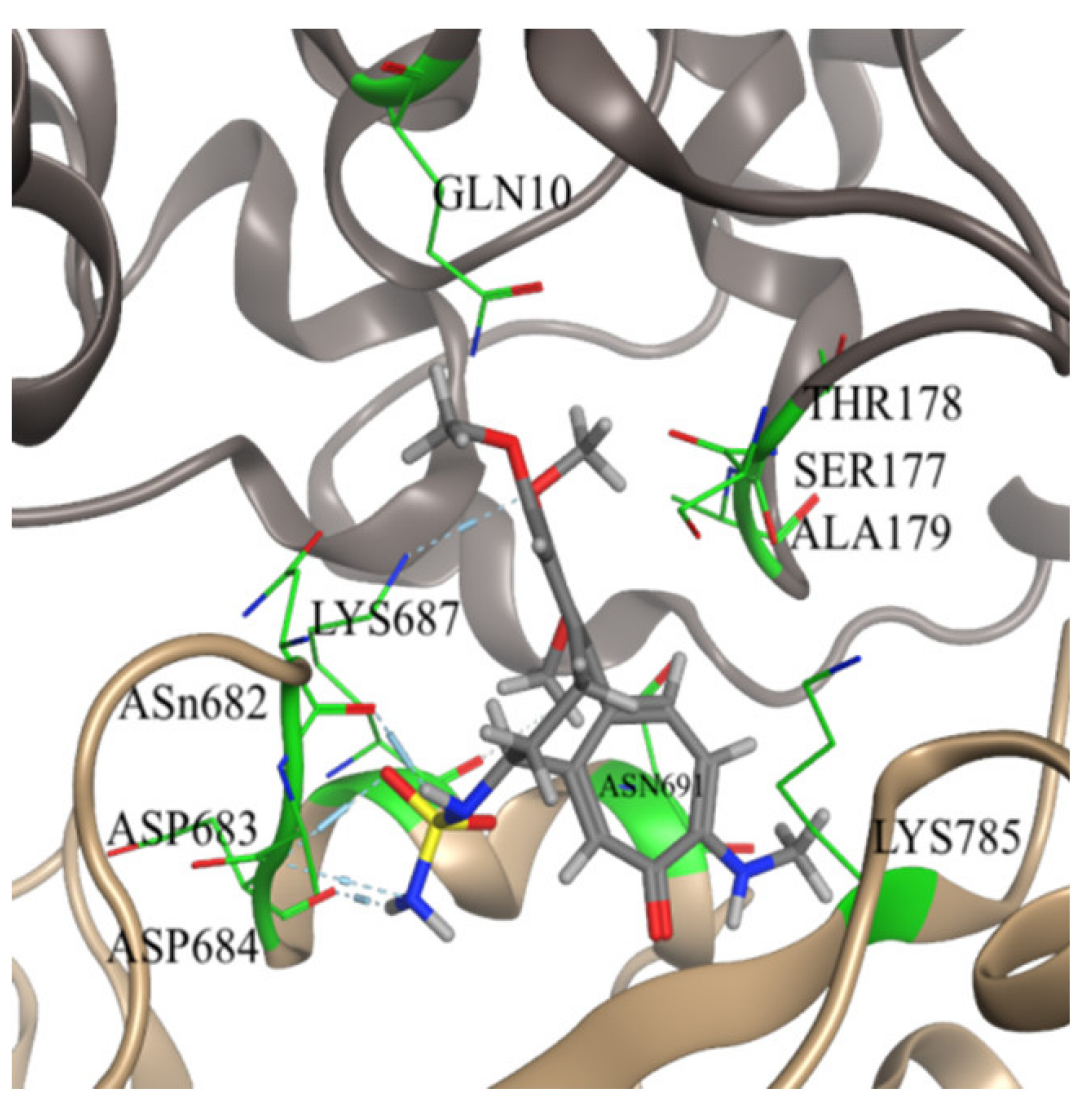	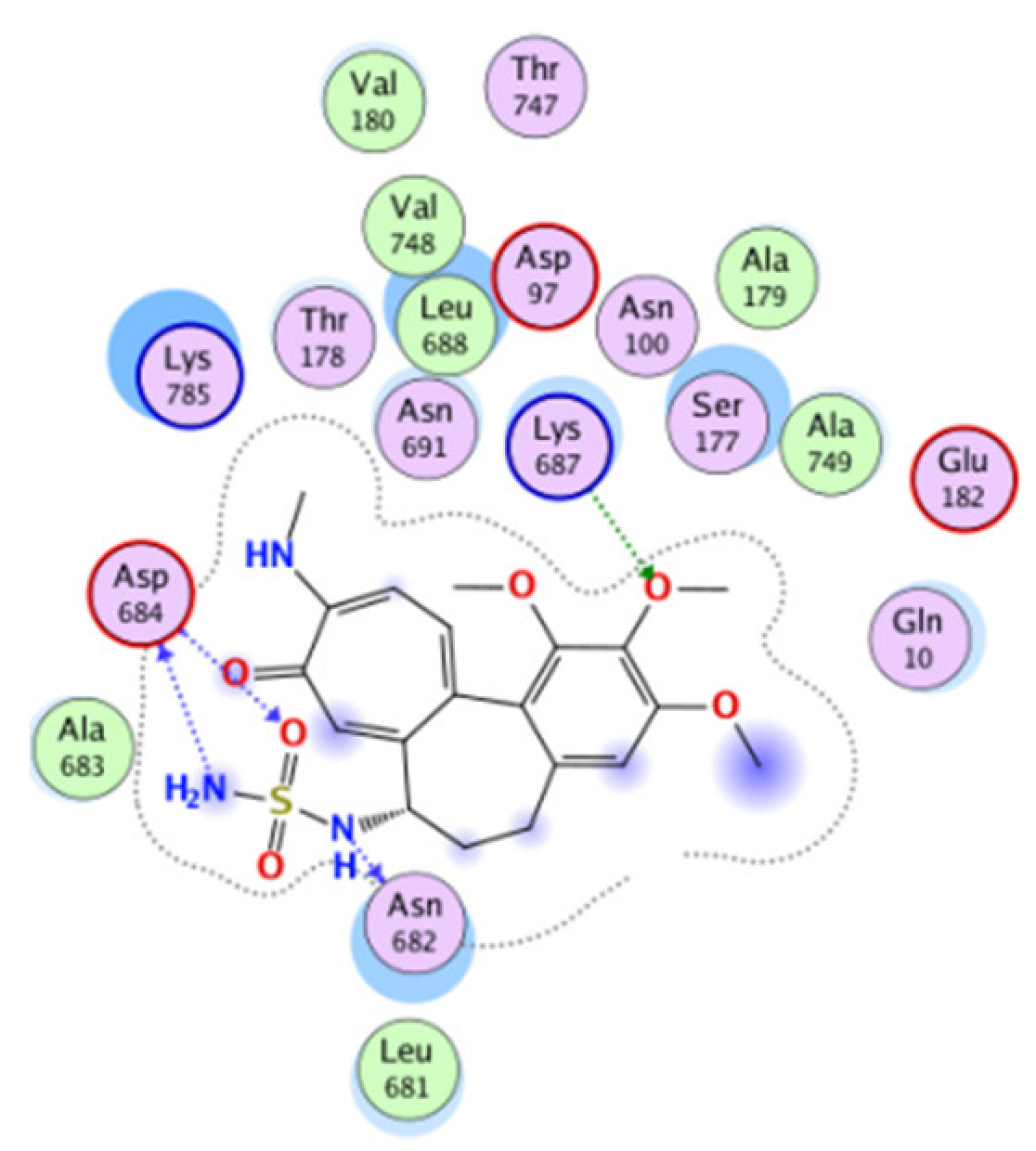	−34.8	1.9	**Gln10** **Ser177** Thr178 Ala179 Asn682 Ala683 **Asp684** **Lys687** Asn691 Lys785
**18**	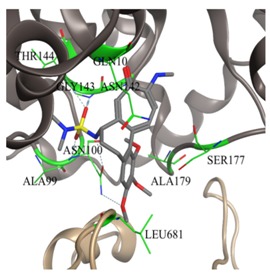	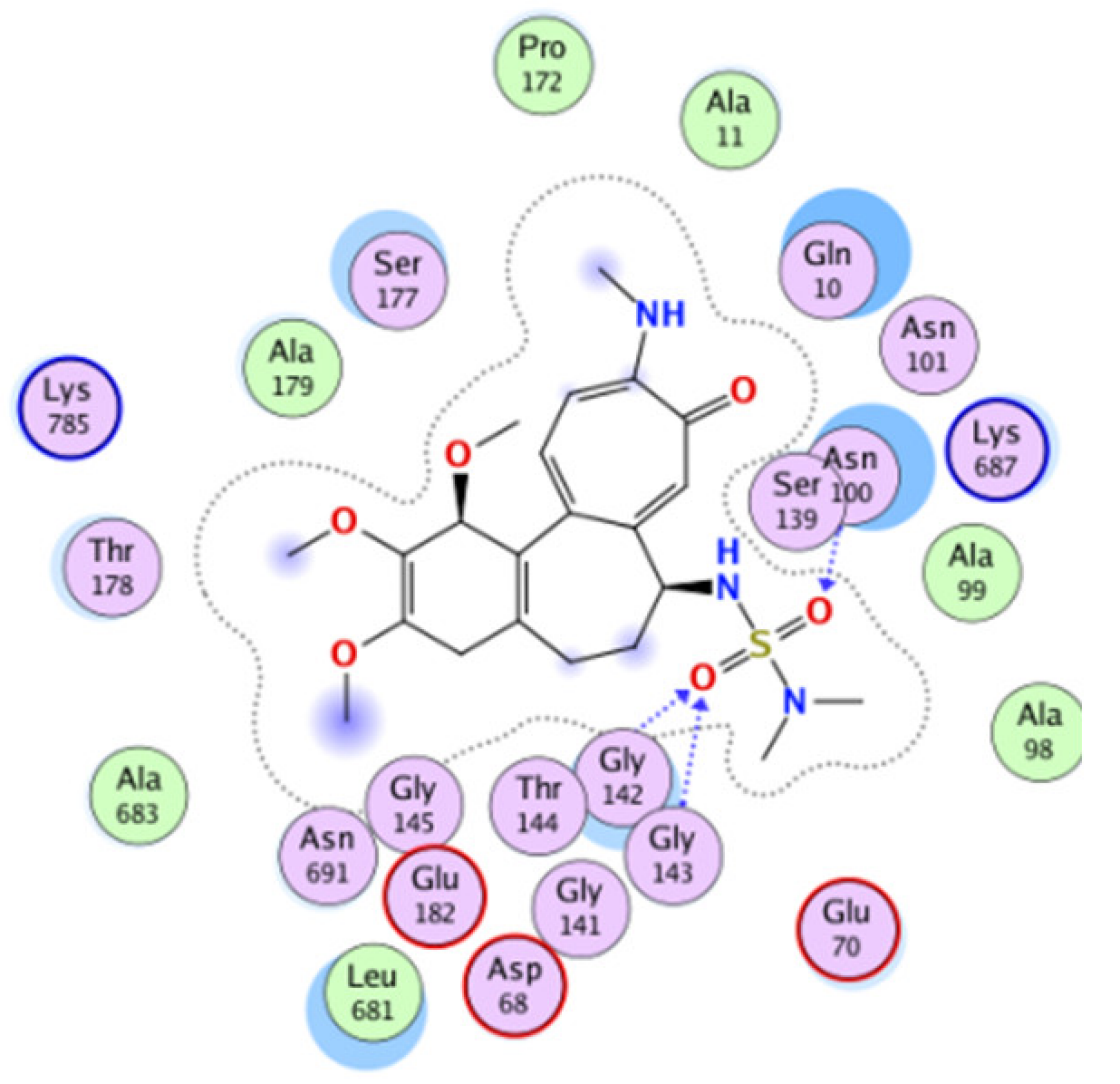	−54.6	2.5	**Gln10** Ala99 Asn100 **Gly142** **Gly143** **Thr144** **Ser177** Thr178 Ala179 Leu681
**19**	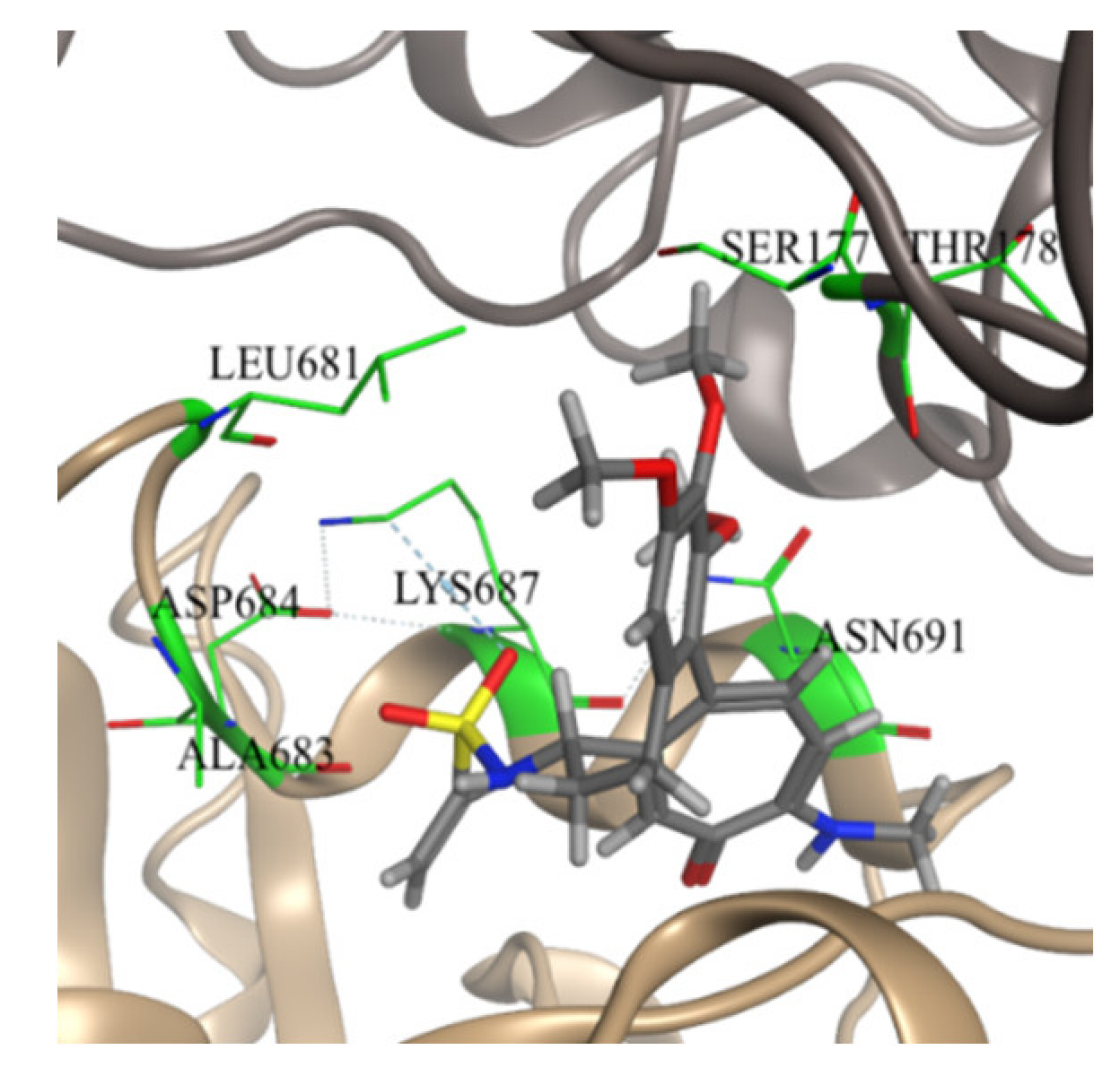	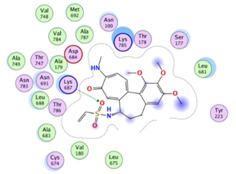	−23.4	2..8	**Ser177** Thr178 **Leu681** Ala683 Asp684 **Lys687** Asn691 **Lys785**
**20**	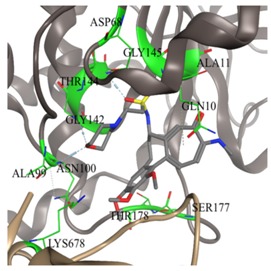	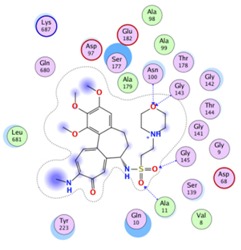	−59.6	2.4	Gly9 Gln10 Ala11 **Asp68** Ala99 **Asn100** Gly142 Gly143 Thr144 **Gly145** Ser177 Thr178 **Lys687**
**21**	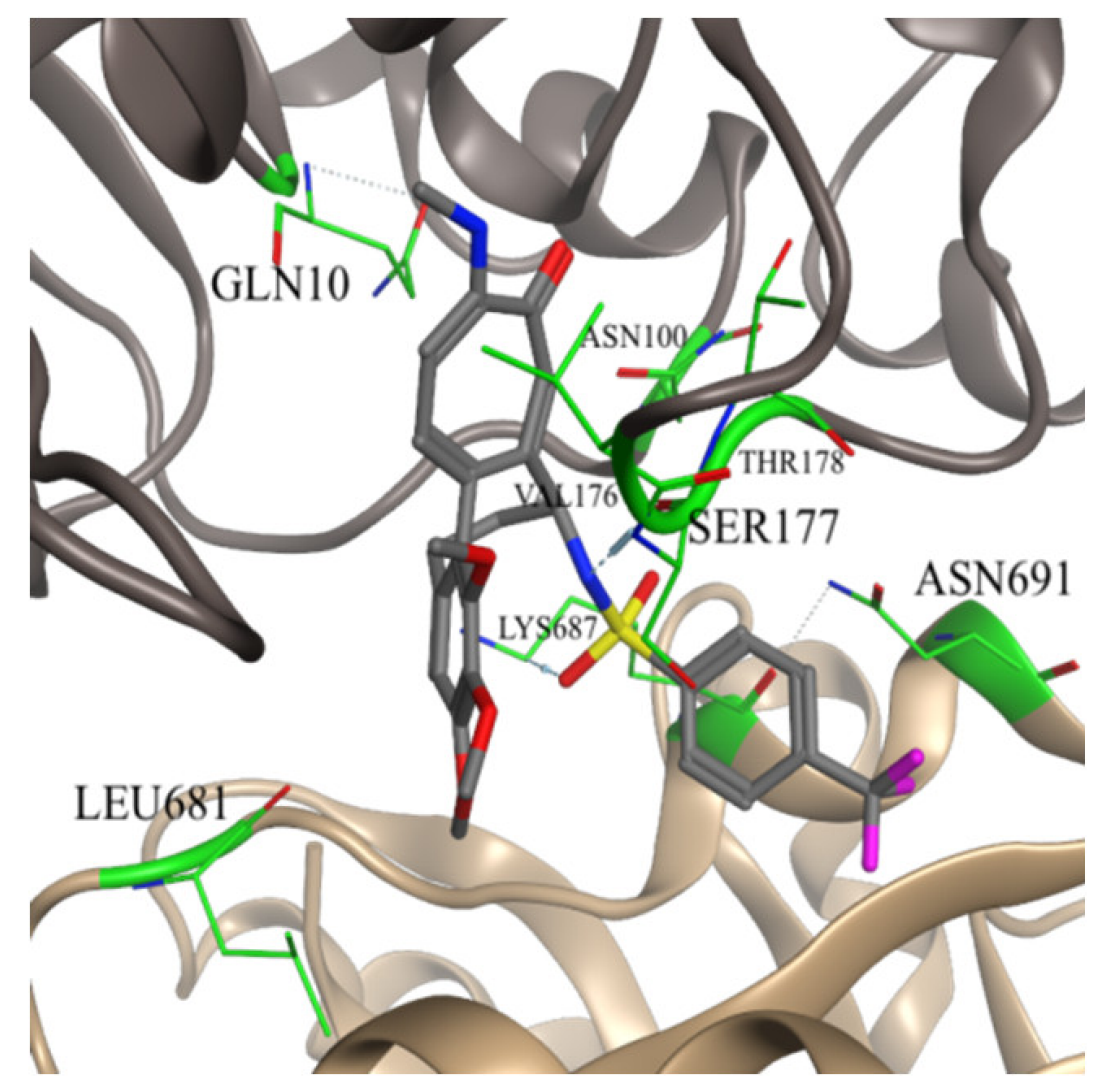	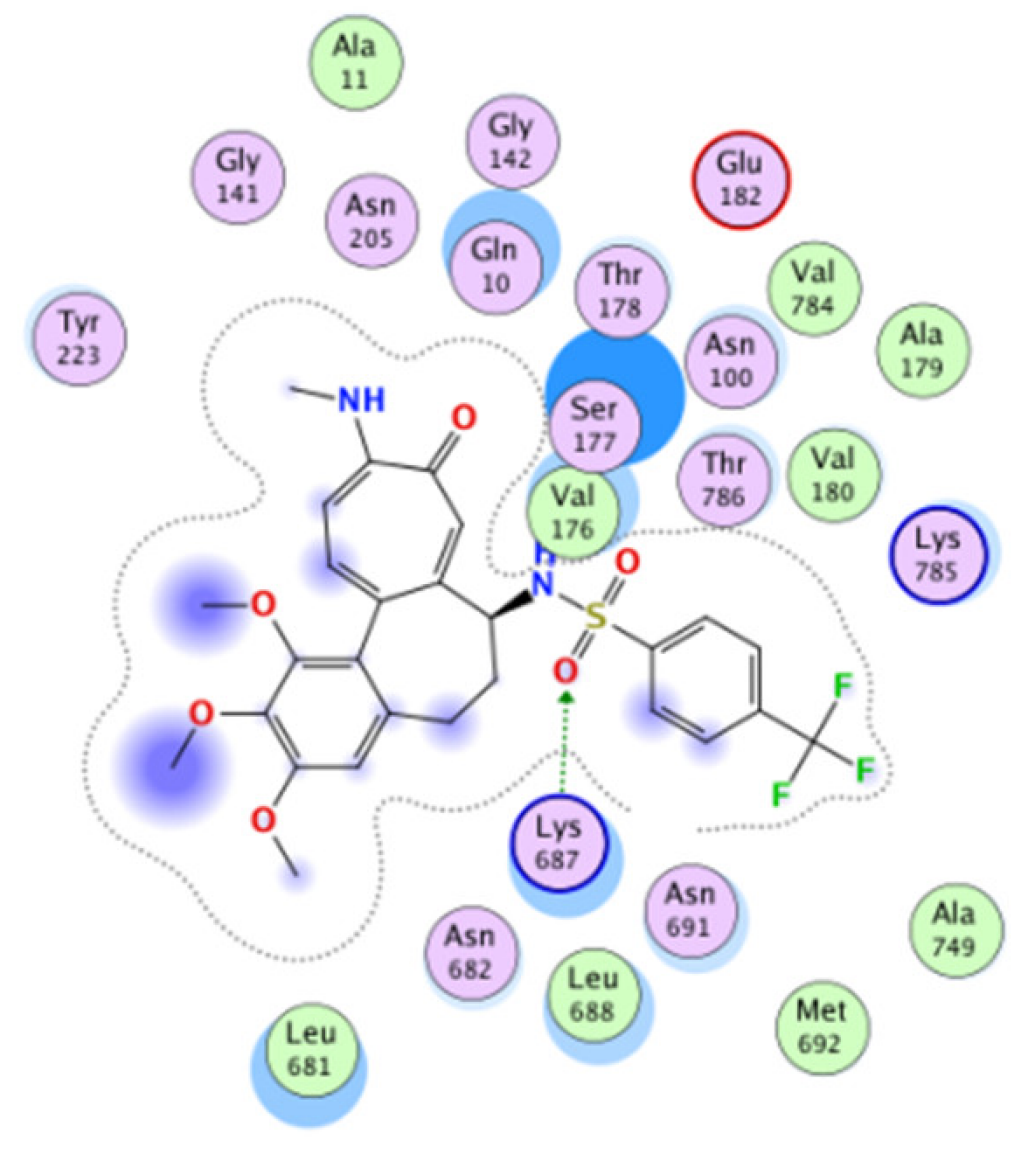	−29.8	4.9	Ala179 Val180 **Cys674** **Leu688** Asn691 Ala749 **Lys785**
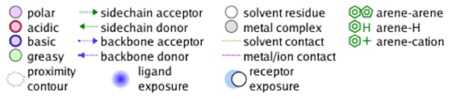					

## Supplementary Materials

The following are available online, Table S1: Details of the data collection parameters, crystallographic data and the final agreement parameters. (for compounds **6**, **11**, **12**, **14**, **15**, **16**, **18** and **19**); Table S2: Optimized parameters for colchicine derivatives (**6**, **11**, **12**, **14**, **15**, **16**, **18** and **19**), Figure S1: The LC-MS chromatogram and mass spectra of **2**; Figure S2: The ^1^H NMR spectrum of **2** in CDCl_3_; Figure S3: The ^13^C NMR spectrum of **2** in CDCl_3_; Figure S4: The LC-MS chromatogram and mass spectra of **3**; Figure S5: The ^1^H NMR spectrum of **3** in CDCl_3_; Figure S6: The ^13^C NMR spectrum of **3** in CDCl_3_; Figure S7: The LC-MS chromatogram and mass spectra of **4**; Figure S8: The ^1^H NMR spectrum of **4** in CDCl_3_, Figure S9: The ^13^C NMR spectrum of **4** in CDCl_3_, Figure S10: The LC-MS chromatogram and mass spectra of **5**; Figure S11: The ^1^H NMR spectrum of **5** in CDCl_3_, Figure S12: The ^13^C NMR spectrum of **5** in CDCl_3_; Figure S13: The LC-MS chromatogram and mass spectra of **6**; Figure S14: The ^1^H NMR spectrum of **6** in CDCl_3_; Figure S15: The ^13^C NMR spectrum of **6** in CDCl_3_; Figure S16: The LC-MS chromatogram and mass spectra of **7**; Figure S17: The ^1^H NMR spectrum of **7** in CDCl_3_; Figure S18: The ^13^C NMR spectrum of **7** in CDCl_3_; Figure S19: The LC-MS chromatogram and mass spectra of **8**; Figure S20: The ^1^H NMR spectrum of **8** in CDCl_3_; Figure S21:. The ^13^C NMR spectrum of **8** in CDCl_3_; Figure S22: The LC-MS chromatogram and mass spectra of **9**; Figure S23: The ^1^H NMR spectrum of **9** in CDCl_3_; Figure S24: The ^13^C NMR spectrum of **9** in CDCl_3_; Figure S25: The LC-MS chromatogram and mass spectra of **10**; Figure S26: The ^1^H NMR spectrum of **10** in CDCl_3_; Figure S27: The ^13^C NMR spectrum of **10** in CDCl_3_; Figure S28. The LC-MS chromatogram and mass spectra of **11**; Figure S29. The ^1^H NMR spectrum of **11** in CDCl_3_; Figure S30: The ^13^C NMR spectrum of **11** in CDCl_3_; Figure S31. The LC-MS chromatogram and mass spectra of **12**; Figure S32: The ^1^H NMR spectrum of **12** in CDCl_3_; Figure S33: The ^13^C NMR spectrum of **12** in CDCl_3_; Figure S34: The LC-MS chromatogram and mass spectra of **13**; Figure S35: The ^1^H NMR spectrum of **13** in CD_2_Cl_2_; Figure S36: The ^13^C NMR spectrum of **13** in CD_2_Cl_2_. Figure S37: The LC-MS chromatogram and mass spectra of **14**; Figure S38: The ^1^H NMR spectrum of **14** in CDCl_3;_ Figure S39: The ^13^C NMR spectrum of **14** in CDCl_3_; Figure S40: The LC-MS chromatogram and mass spectra of **15**; Figure S41: The ^1^H NMR spectrum of **15** in CDCl_3_; Figure S42: The ^13^C NMR spectrum of **15** in CDCl_3;_ Figure S43: The LC-MS chromatogram and mass spectra of **16**; Figure S44: The ^1^H NMR spectrum of **16** in CDCl_3_. Figure S45: The ^13^C NMR spectrum of **16** in CDCl_3_; Figure S46: The LC-MS chromatogram and mass spectra of **17**; Figure S47: The ^1^H NMR spectrum of **17** in CDCl_3_; Figure S48: The LC-MS chromatogram and mass spectra of **18**;Figure S49: The ^1^H NMR spectrum of **18** in CDCl_3_; Figure S50: The ^13^C NMR spectrum of **18** in CDCl_3_; Figure S51: The LC-MS chromatogram and mass spectra of **19**; Figure S52: The ^1^H NMR spectrum of **19** in (CD_3_)_2_SO; Figure S53: The ^13^C NMR spectrum of **19** in (CD_3_)_2_SO; Figure S54: The LC-MS chromatogram and mass spectra of **20**; Figure S55**:** The ^1^H NMR spectrum of **20** in CDCl_3_. Figure S56: The ^13^C NMR spectrum of **20** in CDCl_3_; Figure S57: The LC-MS chromatogram and mass spectra of **21**; Figure S58: The ^1^H NMR spectrum of **21** in CDCl_3_. Figure S59: The ^13^C NMR spectrum of **21** in CDCl_3_; Figure S60: Molecular structure of colchicine derivatives (**6**, **11**, **12**, **14**, **15**, **16**, **18** and **19**) at 295 K and 100 K.
