# Supplementary material for: Synthesis, Antiproliferative Activity and Molecular Docking Studies of Novel Doubly Modified Colchicine Amides and Sulfonamides as Anticancer Agents
Source: Molecules. 2020 Apr 14;25(8):1789. doi: 10.3390/molecules25081789 (PMC7221574; doi:10.3390/molecules25081789)
Supplement: Supplementary file 1 [file molecules-25-01789-s001.pdf]

Synthesis, Antiproliferative Activity and Molecular Docking Studies of Novel Doubly Modified Colchicine Amides and Sulfonamides as Anticancer Agents

Julia Krzywik [1,2], Witold Mozga [2], Maral Aminpour [3], Jan Janczak [4], Ewa Maj [5], Joanna Wietrzyk [5], Jack A. Tuszyński[3,6] and Adam Huczyński [1,*]

[1] Department of Medical Chemistry, Faculty of Chemistry, Adam Mickiewicz University, Uniwersytetu Poznańskiego 8, 61-614 Poznań, Poland; julia.krzywik@amu.edu.pl (J.K.)

[2] TriMen Chemicals, Piłsudskiego 141, 92-318 Łódź, Poland; mozga@trimen.pl (W.M.), julia@trimen.pl (J.K.)

[3] Department of Oncology, University of Alberta, Edmonton, T6G 1Z2, Canada; aminpour@ualberta.ca (M.A.), jack.tuszynski@gmail.com (J.A.T.)

[4] Institute of Low Temperature and Structure Research, Polish Academy of Sciences, PO Box 1410, 50-950 Wrocław, Poland; j.janczak@intibs.pl (J.J.)

[5] Hirszfeld Institute of Immunology and Experimental Therapy, Polish Academy of Sciences, Rudolfa Weigla 12, 53-114 Wrocław, Poland; ewa.maj@hirszfeld.pl (E.M.), joanna.wietrzyk@hirszfeld.pl (J.W.)

[6] DIMEAS, Politecnico di Torino, Corso Duca degli Abruzzi, 24, Torino, 10129, Italy

Supplementary material

Table S1. Details of the data collection parameters, crystallographic data and the final agreement parameters. (for compounds **6**, **11**, **12** ,**14**, **15**, **16**, **18** and **19**).
Table S2. Optimized parameters for colchicine derivatives (**6**, **11**, **12** ,**14**, **15**, **16**, **18** and **19**).
Figure S1. The LC-MS chromatogram and mass spectra of **2**.
Figure S2. The ^{1}H NMR spectrum of **2** in $CDCl_3$.
Figure S3. The ^{13}C NMR spectrum of **2** in $CDCl_3$.
Figure S4. The LC-MS chromatogram and mass spectra of **3**.
Figure S5. The ^{1}H NMR spectrum of **3** in $CDCl_3$.
Figure S6. The ^{13}C NMR spectrum of **3** in $CDCl_3$.
Figure S7. The LC-MS chromatogram and mass spectra of **4**.
Figure S8. The ^{1}H NMR spectrum of **4** in $CDCl_3$.
Figure S9. The ^{13}C NMR spectrum of **4** in $CDCl_3$.
Figure S10. The LC-MS chromatogram and mass spectra of **5**.
Figure S11. The ^{1}H NMR spectrum of **5** in $CDCl_3$.
Figure S12. The ^{13}C NMR spectrum of **5** in $CDCl_3$.
Figure S13. The LC-MS chromatogram and mass spectra of **6**.
Figure S14. The ^{1}H NMR spectrum of **6** in $CDCl_3$.
Figure S15. The ^{13}C NMR spectrum of **6** in $CDCl_3$.

Figure S16. The LC-MS chromatogram and mass spectra of **7**.
Figure S17. The ^{1}H NMR spectrum of **7** in $CDCl_3$.
Figure S18. The ^{13}C NMR spectrum of **7** in $CDCl_3$.
Figure S19. The LC-MS chromatogram and mass spectra of **8**.
Figure S20. The ^{1}H NMR spectrum of **8** in $CDCl_3$.
Figure S21. The ^{13}C NMR spectrum of **8** in $CDCl_3$.
Figure S22. The LC-MS chromatogram and mass spectra of **9**.
Figure S23. The ^{1}H NMR spectrum of **9** in $CDCl_3$.
Figure S24. The ^{13}C NMR spectrum of **9** in $CDCl_3$.
Figure S25. The LC-MS chromatogram and mass spectra of **10**.
Figure S26. The ^{1}H NMR spectrum of **10** in $CDCl_3$.
Figure S27. The ^{13}C NMR spectrum of **10** in $CDCl_3$.
Figure S28. The LC-MS chromatogram and mass spectra of **11**.
Figure S29. The ^{1}H NMR spectrum of **11** in $CDCl_3$.
Figure S30. The ^{13}C NMR spectrum of **11** in $CDCl_3$.
Figure S31. The LC-MS chromatogram and mass spectra of **12**.
Figure S32. The ^{1}H NMR spectrum of **12** in $CDCl_3$.
Figure S33. The ^{13}C NMR spectrum of **12** in $CDCl_3$.
Figure S34. The LC-MS chromatogram and mass spectra of **13**.
Figure S35. The ^{1}H NMR spectrum of **13** in CD_2Cl_2.
Figure S36. The ^{13}C NMR spectrum of **13** in CD_2Cl_2.
Figure S37. The LC-MS chromatogram and mass spectra of **14**.
Figure S38. The ^{1}H NMR spectrum of **14** in $CDCl_3$.
Figure S39. The ^{13}C NMR spectrum of **14** in $CDCl_3$.
Figure S40. The LC-MS chromatogram and mass spectra of **15**.
Figure S41. The ^{1}H NMR spectrum of **15** in $CDCl_3$.
Figure S42. The ^{13}C NMR spectrum of **15** in $CDCl_3$.
Figure S43. The LC-MS chromatogram and mass spectra of **16**.
Figure S44. The ^{1}H NMR spectrum of **16** in $CDCl_3$.
Figure S45. The ^{13}C NMR spectrum of **16** in $CDCl_3$.
Figure S46. The LC-MS chromatogram and mass spectra of **17**.
Figure S47. The ^{1}H NMR spectrum of **17** in $CDCl_3$.
Figure S48. The LC-MS chromatogram and mass spectra of **18**.
Figure S49. The ^{1}H NMR spectrum of **18** in $CDCl_3$.
Figure S50. The ^{13}C NMR spectrum of **18** in $CDCl_3$.
Figure S51. The LC-MS chromatogram and mass spectra of **19**.
Figure S52. The ^{1}H NMR spectrum of **19** in $(CD_3)_2SO$.
Figure S53. The ^{13}C NMR spectrum of **19** in $(CD_3)_2SO$.
Figure S54. The LC-MS chromatogram and mass spectra of **20**.
Figure S55. The ^{1}H NMR spectrum of **20** in $CDCl_3$.
Figure S56. The ^{13}C NMR spectrum of **20** in $CDCl_3$.
Figure S57. The LC-MS chromatogram and mass spectra of **21**.

Figure S58. The ^{1}H NMR spectrum of **21** in $CDCl_3$.
Figure S59. The ^{13}C NMR spectrum of **21** in $CDCl_3$.
Figure S60. Molecular structure of colchicine derivatives (**6**, **11**, **12** ,**14**, **15**, **16**, **18** and **19**) at 295 K and 100 K.

Table S1. Details of the data collection parameters, crystallographic data and the final agreement parameters (for compounds **6**, **11**, **12** ,**14**, **15**, **16**, **18** and **19**).

Crystal size(mm)	**6** $C_{25}H_{32}N_2O_5$ 0.32 × 0.27 × 0.21		**11** $C_{24}H_{27}F_3N_2O_5$ 0.33 × 0.27 × 0.22	
Mol. weight	440.52	440.52	480.47	480.47
Temp. [K]	100(1)	295(1)	100(1)	295(1)
Crystal system	trigonal	trigonal	trigonal	trigonal
Space group	$P3_221$	$P3_221$	$P3_221$	$P3_221$
a, [Å]	9.7178(4)	9.9846(3)	9.8538(4)	9.9997(4)
b, [Å]	9.7178(4)	9.9846(3)	9.8538(4)	9.9997(4)
c, [Å]	42.2684(14)	41.8491(11)	41.929(2)	41.834(2)
α, [°]	90.0	90.0	90.0	90.0
β, [°]	90.0	90.0	90.0	90.0
γ, [°]	120.0	120.0	120.0	120.0
V, [Å^3]	3456.9(3)	3613.1(3)	3525.8(3)	3622.7(3)
Z	6	6	6	6
λ, (Mo*Kα*) [Å]	0.71073	0.71073	0.71073	0.71073
μ [mm^{-1}]	0.088	0.085	0.110	0.107
D_{calc} [g·cm^{-3}]	1.270	1.215	1.358	1.321
F(000)	1416	1416	1512	1512
T_{min}/T_{max}	0.9628/1.0	0.9628/1.0	0.9697/1.0	0.9697/1.0
θ range, [°]	2.42÷27.99	2.41÷28.00	2.58÷27.99	2.58÷27.99
Refls collected,	36324	65309	36030	56405
unique and	5550	5814	5669	5830
observed, $I>2\sigma(I)$	4640	4253	5063	4192
R_{int}	0.0637	0.0520	0.0713	0.0843
R[$F^2>2\sigma(F^2)$]	0.0534	0.0605	0.0631	0.0758
wR(F^2) all refls	0.1221	0.1474	0.1397	0.1979
Goodness-of-fit,S	1.014	1.037	1.008	1.056
$\Delta\rho_{max};\Delta\rho_{min}$[eÅ^{-3}]	0.31; -0.34	0.23, −0.17	0.26, −0.32	0.24, -0.31
Flack parameter	0.1(6)	0.7(6)	0.0(4)	-0.1(6)

Crystal size(mm)	**12** $C_{27}H_{28}N_2O_5$ 0.31 × 0.29 × 0.23		**14** $C_{27}H_{27}FN_2O_5$ 0.28 × 0.22 × 0.15	
Mol. weight	460.51	460.51	478.50	478.50
Temp. [K]	100(1)	295(1)	100(1)	295(1)
Crystal system	trigonal	trigonal	trigonal	trigonal
Space group	$P3_221$	$P3_221$	$P3_221$	$P3_221$
a, [Å]	9.9014(3)	9.9641(3)	9.9118(3)	10.0282(3)
b, [Å]	9.9014(3)	9.9641(3)	9.9118(3)	10.0282(3)
c, [Å]	41.7786(16)	41.691(2)	41.722(2)	41.793(2)
α, [°]	90.0	90.0	90.0	90.0
β, [°]	90.0	90.0	90.0	90.0
γ, [°]	120.0	120.0	120.0	120.0
V, [Å^3]	3547.1(3)	3584.7(2)	3549.8(2)	3639.8(2)
Z	6	6	6	6
λ, (Mo*Kα*) [Å]	0.71073	0.71073	0.71073	0.71073
μ [mm^{-1}]	0.090	0.090	0.098	0.096
D_{calc} [g·cm^{-3}]	1.293	1.283	1.343	1.310
F(000)	1464	1464	1512	1512
T_{min}/T_{max}	0.9522/1.0	0.9872/1.0	0.9618/1.0	0.9622/1.0
θ range, [°]	2.57÷28.00	2.36÷27.99	2.56÷27.50	2.40÷28.00
Refls collected,	47745	67834	44528	64873
unique and	5705	5784	5426	5846
observed, $I>2\sigma(I)$	5149	4510	4852	5087
R_{int}	0.0463	0.0469	0.0416	0.0350
R[$F^2>2\sigma(F^2)$]	0.0401	0.0490	0.0590	0.0598
wR(F^2) all refls	0.0938	0.1145	0.1579	0.1592
Goodness-of-fit,S	1.001	1.002	1.059	1.002
$\Delta\rho_{max};\Delta\rho_{min}$[eÅ^{-3}]	0.26, −0.29	0.16, -0.15	0.90, -061	0.85, -0.34
Flack parameter	0.3(4)	0.3(5)	0.3(8)	-0.4(4)

Crystal size(mm)	**15** $C_{26}H_{27}N_3O_5$ 0.29 × 0.26 × 0.15		**16** $C_{26}H_{27}N_3O_5$ 0.32 × 0.25 × 0.19	
Mol. weight	461.50	461.50	461.50	461.50
Temp. [K]	100(1)	295(1)	100(1)	295(1)
Crystal system	trigonal	trigonal	trigonal	trigonal
Space group	$P3_221$	$P3_221$	$P3_221$	$P3_221$
a, [Å]	9.8951(3)	9.9988(3)	9.8283(3)	9.9641(3)
b, [Å]	9.8951(3)	9.9988(3)	9.8283(3)	9.9641(3)
c, [Å]	41.6172(11)	41.776(2	41.596(2)	41.691(2)
α, [°]	90.0	90.0	90.0	90.0
β, [°]	90.0	90.0	90.0	90.0
γ, [°]	120.0	120.0	120.0	120.0
V, [Å^3]	3528.9(2)	3617.1(2)	3479.7(3)	3584.7(2)
Z	6	6	6	6
λ, (Mo*Kα*) [Å]	0.71073	0.71073	0.71073	0.71073
μ [mm^{-1}]	0.091	0.089	0.093	0.090
D_{calc} [g·cm^{-3}]	1.303	1.271	1.321	1.283
F(000)	1464	1464	1464	1464
T_{min}/T_{max}	0.9594/1.0	0.9594/1.0	0.9872/1.0	0.9872/1.0
θ range, [°]	2.67÷28.00	2.67÷28.00	2.39÷28.00	2.36÷27.99
Refls collected,	66157	61740	95268	67834
unique and	5677	5529	5596	5784
observed, $I>2\sigma(I)$	5301	4110	4906	4510
R_{int}	0.0700	0.0550	0.0590	0.0469
R[$F^2>2\sigma(F^2)$]	0.0587	0.0560	0.0485	0.0490
wR(F^2) all refls	0.1377	0.1218	0.1149	0.1145
Goodness-of-fit, S	1.002	1.061	1.000	1.002
$\Delta\rho_{max};\Delta\rho_{min}$[eÅ^{-3}]	0.29, -0.28	0.15, -0.18	0.42, -0.37	0.16, -0.15
Flack parameter	0.0(4)	-0.7(4)	-0.2(3)	0.3(5)

Crystal size(mm)	**18** $C_{22}H_{29}N_3O_6S$ 0.32 × 0.25 × 0.19		**19** $C_{22}H_{26}N_2O_6S$ 0.29 × 0.21 × 0.16	
Mol. weight	463.54	463.54	446.51	446.51
Temp. [K]	100(1)	295(1)	100(1)	295(1)
Crystal system	orthorhombic	orthorhombic	monoclinic	monoclinic
Space group	$P2_12_12_1$	$P2_12_12_1$	$P2_1$	$P2_1$
a, [Å]	7.4663(3)	7.5770(2)	8.9687(3)	9.1044(5)
b, [Å]	11.8854(5)	12.0086(3)	8.7611(3)	8.7682(4)
c, [Å]	25.5743(9)	25.8218(6)	14.2839(5)	14.3323(7)
α, [°]	90.0	90.0	90.0	90.0
β, [°]	90.0	90.0	99.874(3)	100.30(1)
γ, [°]	90.0	90.0	90.0	90.0
V, [Å^3]	2269.46(1)	2349.5(1)	1105.74(7)	1125.7(1)
Z	4	4	2	2
λ, (Mo*Kα*) [Å]	0.71073	0.71073	0.71073	0.71073
μ [mm^{-1}]	0.186	0.180	0.187	0.184
D_{calc} [g·cm^{-3}]	1.357	1.310	1.341	1.317
F(000)	984	984	472	472
T_{min}/T_{max}	0.9778/1.0	0.9782/1.0	0.9700/1.0	0.9705/1.0
θ range, [°]	2.84÷26.99	2.80÷27.00	2.50÷26.99	2.47÷26.99
Refls collected,	30386	72807	19325	17917
unique and	4954	5135	4794	4877
observed, $I>2\sigma(I)$	3993	3866	4345	3769
R_{int}	0.0728	0.0735	0.0409	0.0377
R[$F^2>2\sigma(F^2)$]	0.0443	0.0471	0.0347	0.0434
wR(F^2) all refls	0.0890	0.0947	0.0762	0.0957
Goodness-of-fit, S	1.001	1.001	1.000	1.000
$\Delta\rho_{max};\Delta\rho_{min}$[eÅ^{-3}]	0.23, -0.35	0.15, -0.23	0.18, -0.28	0.16, -0.32
Flack parameter	-0.07(5)	-0.02(3)	0.04(4)	-0.03(4)

Table S2. Optimized parameters for colchicine derivatives (**6**, **11**, **12** ,**14**, **15**, **16**, **18** and **19**).

(a) 6

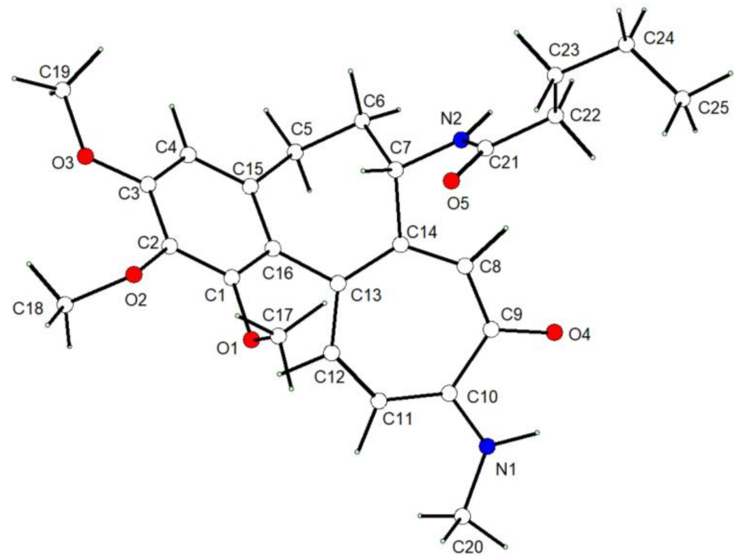

C1–O1	1.397	C2–O2	1.395	C3–O3	1.392
O1–C17	1.479	O2–C18	1.472	O3–C19	1.456
C10–N1	1.358	N1–C20	1.453	C9–O4	1.285
C7–N2	1.463	N2–C21	1.373	C21–O5	1.256
C21–C22	1.520	C22–C23	1.543	C23–C24	1.543
C24–C25	1.540	C1–C2	1.404	C2–C3	1.407
C3–C4	1.399	C4–C15	1.403	C15–C16	1.415
C15–C5	1.516	C5–C6	1.550	C6–C7	1.549
C7–C14	1.546	C14–C8	1.385	C8–C9	1.429
C9–C10	1.483	C10–C11	1.495	C11–C12	1.406
C12–C13	1.396	C13–C14	1.433		
C1–O1–C17	116.31	C2–O2–C18	117.13	C3–O3–C19	118.94
C1–C16–C13	121.26	C16–C13–C14	119.03	C16–C15–C5	119.17
C4–C15–C5	120.35	C15–C5–C6	111.89	C5–C6–C7	112.40
C6–C7–C14	111.60	C7–C14–C13	115.32	C6–C7–N2	109.35
C7–N2–C21	122.21	N2–C21–O2	121.68	N2–C21–C22	116.28
C21–C22–C23	112.24	C22–C23–C24	113.73	C23–C24–C25	114.34
C10–N1–C20	125.95	C11–C10–N1	121.94	C9–C10–N1	111.70
C8–C9–O4	119.75	O4–C9–C10	116.38	C8–C9–C10	123.86
C9–C10–C11	126.36	C10–C11–C12	130.53	C11–C12–C13	132.26

C1–C16–C13–C12	53.14	C17–O1–C1–C2	-72.99
C18–O2–C2–C3	70.11	C19–O3–C3–C4	-4.01
O1–C1–C2–O2	6.07	O2–C2–C3–O3	-4.85
C11–C10–N1–C20	-0.89	N1–C10–C9–O4	1.07
C1–C16–C13–C14	-127.47	C3–C4–C15–C5	-177.12
C4–C15–C5–C6	107.24	C15–C5–C6–C7	42.24
C5–C6–C7–N2	175.23	C5–C6–C7–C14	47.32
C6–C7–C14–C13	-77.84	C7–C14–C13–C16	2.74
C14–C13–C16–C15	53.70	C14–C7–N2–C21	-87.08
C7–N2–C21–O5	-0.98	N2–C21–C22–C23	134.11
C17–O1–C1–C16	108.72		

(b) 11

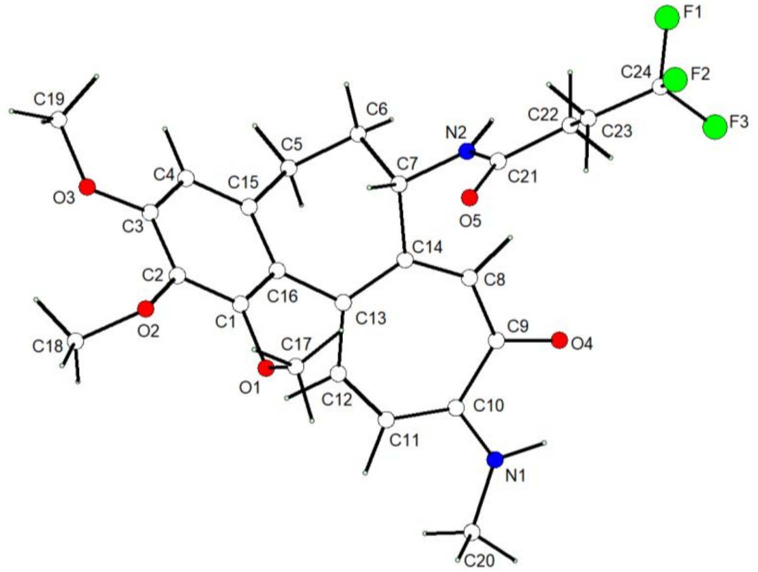

C1–O1	1.397	C2–O2	1.395	C3–O3	1.392
O1–C17	1.479	O2–C18	1.472	O3–C19	1.456
C10–N1	1.357	N1–C20	1.453	C9–O4	1.284
C7–N2	1.465	N2–C21	1.368	C21–O5	1.255
C21–C22	1.525	C22–C23	1.535	C23–C24	1.501
C24–F1	1.408	C24–F2	1.400	C24–F3	1.405
C1–C2	1.404	C2–C3	1.407	C3–C4	1.399
C4–C15	1.403	C15–C16	1.415	C1–C16	1.415
C15–C5	1.516	C6–C6	1.550	C6–C7	1.548
C7–C14	1.546	C14–C13	1.433	C14–C8	1.385
C8–C9	1.429	C9–C10	1.483	C10–C11	1.395
C11–C12	1.406	C12–C13	1.396	C13–C16	1.501
C1–O1–C17	116.65	C2–O2–C18	117.23	C3–O3–C19	118.98
C1–C16–C13	121.29	O1–C1–C2	119.31	O2–C2–C3	121.85
O3–C3–C4	124.15	C4–C15–C5	120.20	C15–C5–C6	112.03
C5–C6–C7	112.23	C6–C7–C14	111.72	C7–C14–C13	115.34
C14–C13–C16	119.11	C14–C8–C9	134.35	C8–C9–C10	123.86
C8–C9–O4	119.71	O4–C9–C10	116.43	C9–C10–N1	111.69
C10–N1–C20	125.99	N1–C10–C11	122.00	C10–C11–C12	130.53
C11–C12–C13	132.34	C12–C13–C14	124.59	C12–C13–C16	116.30
C23–C24–F1	112.68	C23–C24–F2	112.00	C23–C24–F3	112.94

C1–C16–C13–C12	52.32	C17–O1–C1–C2	-70.70
C18–O2–C2–C3	68.81	C19–O3–C3–C4	-3.68
O1–C1–C2–O2	5.82	O2–C2–C3–O3	-4.33
C11–C10–N1–C20	-0.91	N1–C10–C9–O4	1.07
C1–C16–C13–C14	-128.43	C3–C4–C15–C5	-177.65
C4–C15–C5–C6	107.54	C15–C5–C6–C7	42.31
C5–C6–C7–N2	174.97	C5–C6–C7–C14	47.21
C6–C7–C14–C13	-78.18	C7–C14–C13–C16	3.28
C14–C13–C16–C15	53.15	C14–C7–N2–C21	-86.58
C7–N2–C21–O5	-1.57	N2–C21–C22–C23	161.04
C21–C22–C23–C24	177.11	C22–C23–C24–F1	59.90
C22–C23–C24–F2	179.64	C22–C23–C24–F3	-60.14
C17–O1–C1–C16	111.35		

(c) 12

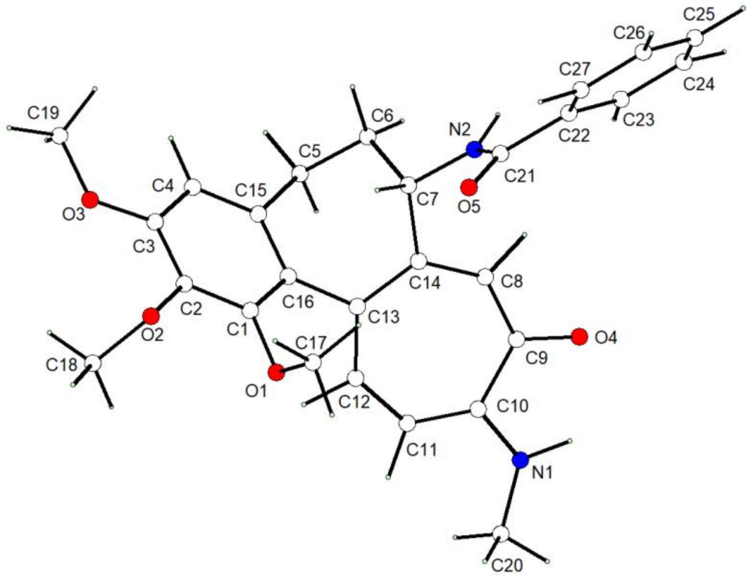

C1–O1	1.375	C2–O2	1.373	C3–O3	1.365
O1–C17	1.439	O2–C18	1.432	O3–C19	1.422
C10–N1	1.350	N1–C20	1.445	C9–O4	1.254
C7–N2	1.455	N2–C21	1.371	C21–O5	1.231
C1–C2	1.402	C2–C3	1.408	C3–C4	1.398
C4–C15	1.400	C15–C16	1.410	C1–C16	1.413
C15–C5	1.514	C5–C6	1.545	C6–C7	1.544
C7–C14	1.543	C14–C8	1.381	C8–C9	1.436
C9–C10	1.490	C10–C11	1.392	C11–C12	1.406
C12–C13	1.391	C13–C14	1.433	C13–C16	1.500
C1–O1–C17	114.71	C2–O2–C18	115.11	C3–O3–C19	118.49
C1–C16–C13	121.34	O1–C1–C2	118.52	O2–C2–C3	120.67
O3–C3–C4	124.77	C4–C15–C5	120.21	C15–C5–C6	111.96
C5–C6–C7	112.66	C6–C7–C14	111.53	C7–C14–C13	115.24
C14–C13–C16	119.19	C14–C8–C9	134.67	C8–C9–C10	123.06
C8–C9–O4	120.20	O4–C9–C10	116.74	C9–C10–N1	111.41
C10–N1–C20	126.26	N1–C10–C11	122.10	C10–C11–C12	130.64
C11–C12–C13	132.47	C12–C13–C14	124.45	C12–C13–C16	116.35
C1–C16–C13–C12		53.39	C17–O1–C1–C2	-80.30	
C18–O2–C2–C3		82.09	C19–O3–C3–C4	-2.00	
O1–C1–C2–O2		4.82	O2–C2–C3–O3	-4.20	
C11–C10–N1–C20		-1.38	N1–C10–C9–O4	1.78	
C1–C16–C13–C14		-127.62	C3–C4–C15–C5	-177.11	
C4–C15–C5–C6		107.46	C15–C5–C6–C7	42.75	
C5–C6–C7–N2		174.80	C5–C6–C7–C14	47.76	
C6–C7–C14–C13		-78.83	C7–C14–C13–C16	3.34	
C14–C13–C16–C15		53.58	C14–C7–N2–C21	-91.67	
C7–N2–C21–O5		0.17	N2–C21–C22–C23	-28.25	
O5–C21–C22–C23		152.51	C21–C22–C23–C24	-179.24	

(d) 14

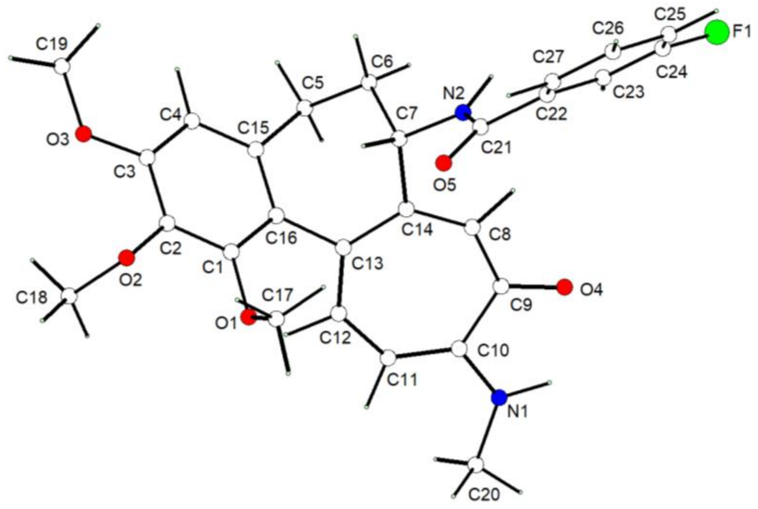

C1–O1	1.375	C2–O2	1.373	C3–O3	1.365
O1–C17	1.439	O2–C18	1.433	O3–C19	1.422
C10–N1	1.350	N1–C20	1.445	C9–O4	1.253
C7–N2	1.456	N2–C21	1.369	C21–O5	1.230
C1–C2	1.402	C2–C3	1.408	C3–C4	1.399
C4–C15	1.400	C15–C16	1.410	C1–C16	1.413
C15–C5	1.514	C5–C6	1.545	C6–C7	1.544
C7–C14	1.543	C14–C13	1.432	C13–C16	1.500
C14–C8	1.381	C8–C9	1.436	C9–C10	1.490
C10–C11	1.392	C11–C12	1.405	C12–C13	1.391
C21–C22	1.507	C22–C23	1.403	C23–C24	1.389
C24–C25	1.389	C25–C26	1.398	C26–C27	1.395
C27–C22	1.402	C24–F1	1.360		
C1–O1–C17	114.78	C2–O2–C18	115.13	C3–O3–C19	118.48
C1–C16–C13	121.31	O1–C1–C2	118.57	O2–C2–C3	120.66
O3–C3–C4	124.74	C4–C15–C5	120.23	C15–C5–C6	112.00
C5–C6–C7	112.75	C6–C7–C14	111.58	C7–C14–C13	115.20
C14–C13–C16	119.25	C14–C8–C9	134.66	C8–C9–C10	123.09
C8–C9–O4	120.17	O4–C9–C10	116.74	C9–C10–N1	111.41
C10–N1–C20	126.27	N1–C10–C11	122.12	C10–C11–C12	130.63
C11–C12–C13	132.52	C12–C13–C14	124.44	C12–C13–C16	116.30

C1–C16–C13–C12	53.54	C17–O1–C1–C2	-80.03
C18–O2–C2–C3	82.28	C19–O3–C3–C4	-1.89
O1–C1–C2–O2	4.96	O2–C2–C3–O3	-4.23
C11–C10–N1–C20	-0.96	N1–C10–C9–O4	1.45
C1–C16–C13–C14	-127.54	C3–C4–C15–C5	-177.30
C4–C15–C5–C6	107.81	C15–C5–C6–C7	42.48
C5–C6–C7–N2	174.95	C5–C6–C7–C14	46.97
C6–C7–C14–C13	-77.56	C7–C14–C13–C16	2.92
C14–C13–C16–C15	53.84	C14–C7–N2–C21	-91.42
C7–N2–C21–O5	1.16	N2–C21–C22–C23	-27.73
O5–C21–C22–C23	153.15	C21–C22–C23–C24	-179.60
C22–C23–C24–C25	-0.18	C22–C23–C24–F1	179.42

(e) 15

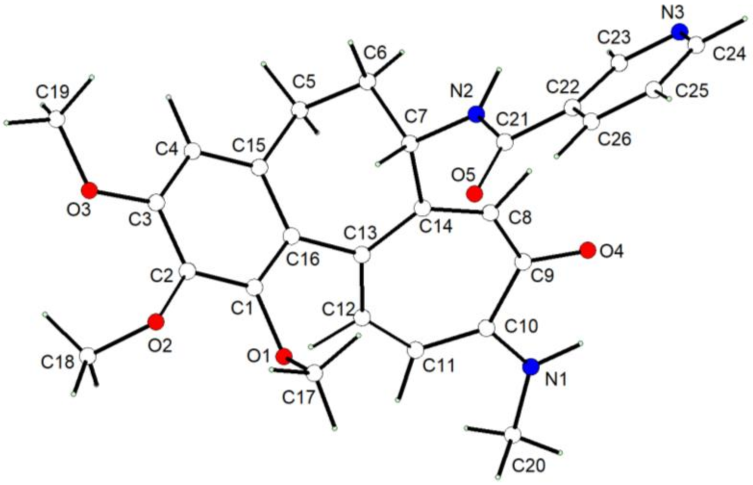

C1–O1	1.397	C2–O2	1.395	C3–O3	1.392
O1–C17	1.479	O2–C18	1.472	O3–C19	1.456
C10–N1	1.358	N1–C20	1.453	C9–O4	1.284
C7–N2	1.464	N2–C21	1.370	C21–O5	1.258
C1–C2	1.403	C2–C3	1.407	C3–C4	1.399
C4–C15	1.403	C15–C16	1.415	C1–C16	1.415
C15–C5	1.516	C5–C6	1.551	C6–C7	1.548
C7–C14	1.546	C14–C13	1.433	C13–C16	1.501
C14–C8	1.385	C8–C9	1.430	C9–C10	1.484
C10–C11	1.395	C11–C12	1.406	C12–C13	1.396
C21–C22	1.498	C22–C23	1.405	C23–N3	1.351
N3–C24	1.353	C24–C25	1.401	C25–C26	1.398
C1–O1–C17	116.35	C2–O2–C18	116.99	C3–O3–C19	118.97
C1–C16–C13	121.31	O1–C1–C2	119.16	O2–C2–C3	121.68
O3–C3–C4	124.23	C4–C15–C5	120.28	C15–C5–C6	111.91
C5–C6–C7	112.11	C6–C7–C14	111.65	C7–C14–C13	115.19
C14–C13–C16	118.99	C14–C8–C9	134.22	C8–C9–C10	123.84
C8–C9–O4	119.77	O4–C9–C10	116.38	C9–C10–N1	111.67
C10–N1–C20	125.97	N1–C10–C11	121.94	C10–C11–C12	130.52
C11–C12–C13	132.24	C12–C13–C14	124.65	C12–C13–C16	116.35
C22–C23–N3	123.35	C23–N3–C24	117.95	N3–C24–C25	122.77
C24–C25–C26	118.78	C26–C22–C23	117.84	C21–C22–C26	118.05

C1–C16–C13–C12	52.88	C17–O1–C1–C2	-72.91
C18–O2–C2–C3	71.05	C19–O3–C3–C4	-3.78
O1–C1–C2–O2	6.00	O2–C2–C3–O3	-4.89
C11–C10–N1–C20	-1.19	N1–C10–C9–O4	1.51
C1–C16–C13–C14	-127.78	C3–C4–C15–C5	-177.09
C4–C15–C5–C6	107.16	C15–C5–C6–C7	42.40
C5–C6–C7–N2	175.15	C5–C6–C7–C14	47.37
C6–C7–C14–C13	-78.49	C7–C14–C13–C16	3.35
C14–C13–C16–C15	53.35	C14–C7–N2–C21	-87.31
C7–N2–C21–O5	-0.45	N2–C21–C22–C23	-17.97
C21–C22–C23–N3	179.53	C22–C23–N3–C24	0.46
C7–C14–C8–C9	-179.77	C13–C16–C15–C5	-4.22

(f) 16

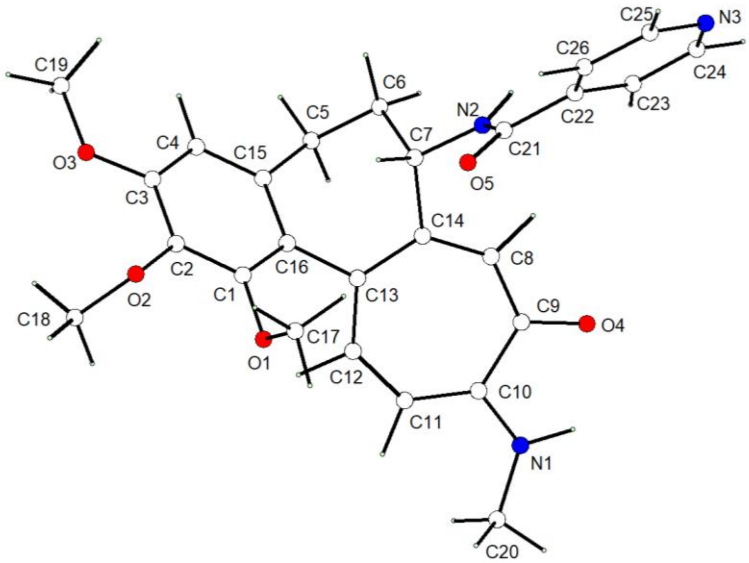

C1–O1	1.374	C2–O2	1.373	C3–O3	1.365
O1–C17	1.439	O2–C18	1.433	O3–C19	1.422
C10–N1	1.350	N1–C20	1.445	C9–O4	1.253
C7–N2	1.456	N2–C21	1.367	C21–O5	1.229
C1–C2	1.402	C2–C3	1.408	C3–C4	1.399
C4–C15	1.400	C15–C16	1.410	C1–C16	1.413
C15–C5	1.514	C5–C6	1.545	C6–C7	1.544
C7–C14	1.543	C14–C13	1.432	C13–C16	1.500
C14–C8	1.381	C8–C9	1.436	C9–C10	1.490
C10–C11	1.392	C11–C12	1.405	C12–C13	1.392
C21–C22	1.508	C22–C23	1.400	C23–C24	1.398
C24–N3	1.338	N3–C25	1.341	C25–C26	1.395
C1–O1–C17	114.85	C2–O2–C18	115.18	C3–O3–C19	118.53
C1–C16–C13	119.97	O1–C1–C2	118.62	O2–C2–C3	120.73
O3–C3–C4	124.77	C4–C15–C5	120.19	C15–C5–C6	111.98
C5–C6–C7	112.56	C6–C7–C14	111.62	C7–C14–C13	115.21
C14–C13–C16	119.22	C14–C8–C9	134.69	C8–C9–C10	123.05
C8–C9–O4	120.16	O4–C9–C10	116.78	C9–C10–N1	111.42
C10–N1–C20	126.28	N1–C10–C11	122.11	C10–C11–C12	130.66
C11–C12–C13	132.51	C12–C13–C14	124.39	C12–C13–C16	116.39
C6–C7–N2	109.36	C7–N2–C21	122.20	N2–C21–O5	122.79
N2–C21–C22	116.03	C21–C22–C23	123.81	C22–C23–C24	118.75
C23–C24–N3	123.75	C24–N3–C25	117.06	N3–C25–C26	123.77

C1–C16–C13–C12	53.27	C17–O1–C1–C2	-79.35
C18–O2–C2–C3	81.14	C19–O3–C3–C4	-1.89
O1–C1–C2–O2	4.87	O2–C2–C3–O3	-4.06
C11–C10–N1–C20	-1.33	N1–C10–C9–O4	1.72
C1–C16–C13–C14	-127.76	C3–C4–C15–C5	-177.24
C4–C15–C5–C6	107.45	C15–C5–C6–C7	42.67
C5–C6–C7–N2	174.84	C5–C6–C7–C14	46.87
C6–C7–C14–C13	-77.86	C7–C14–C13–C16	3.27
C14–C13–C16–C15	53.47	C14–C7–N2–C21	-91.33
C7–N2–C21–O5	-0.05	N2–C21–C22–C23	-29.97
C21–C22–C23–C24	-179.12	C22–C23–C24–N3	-0.47
C23–C24–N3–C25	0.93	C24–N3–C25–C26	-0.21

(g) 18

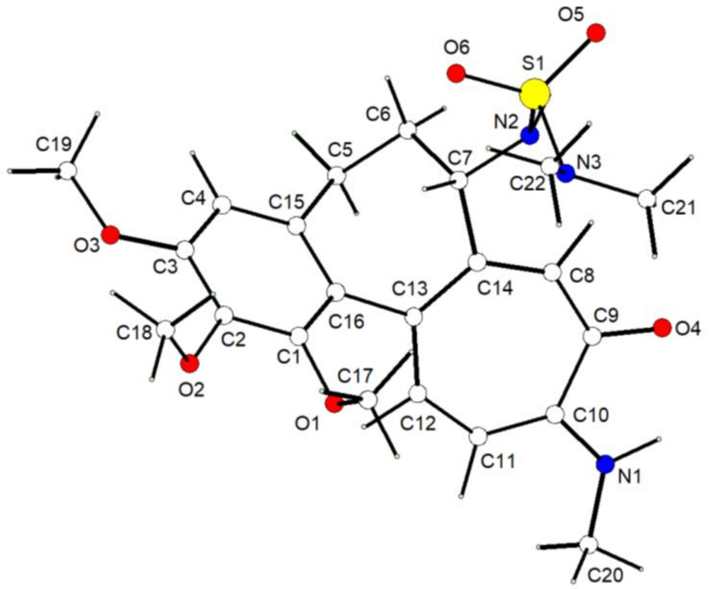

C1–O1	1.375	C2–O2	1.371	C3–O3	1.367
O1–C17	1.430	O2–C18	1.434	O3–C19	1.423
C10–N1	1.350	N1–C20	1.445	C9–O4	1.253
C7–N2	1.472	N2–S1	1.676	S1–O5	1.469
S1–O6	1.467	S1–N3	1.695	N3–C21	1.472
N3–C22	1.473	C1–C2	1.406	C2–C3	1.410
C3–C4	1.398	C4–C15	1.398	C15–C16	1.409
C1–C16	1.413	C15–C5	1.512	C5–C6	1.543
C6–C7	1.547	C7–C14	1.541	C14–C13	1.434
C13–C16	1.501	C14–C8	1.382	C8–C9	1.437
C9–C10	1.490	C10–C11	1.392	C11–C12	1.405
C12–C13	1.392				
C1–O1–C17	116.30	C2–O2–C18	117.91	C3–O3–C19	118.61
C1–C16–C13	119.02	O1–C1–C2	119.33	O2–C2–C3	122.68
O3–C3–C4	124.14	C4–C15–C5	120.47	C15–C5–C6	112.02
C5–C6–C7	113.28	C6–C7–C14	111.84	C7–C14–C13	115.36
C14–C13–C16	119.53	C14–C8–C9	134.69	C8–C9–C10	123.24
C8–C9–O4	120.10	O4–C9–C10	116.66	C9–C10–N1	111.44
C10–N1–C20	126.27	N1–C10–C11	122.13	C10–C11–C12	130.59
C11–C12–C13	132.65	C12–C13–C14	124.47	C12–C13–C16	115.98
C6–C7–N2	109.70	C7–N2–S1	119.57	N2–S1–O5	104.55
N2–S1–O6	112.09	N2–S1–N3	100.10	S1–N3–C21	114.05
S1–N3–C22	113.93	C21–N3–C22	113.26		

C1–C16–C13–C12	54.48	C17–O1–C1–C2	-74.76
C18–O2–C2–C3	-59.62	C19–O3–C3–C4	0.66
O1–C1–C2–O2	-0.48	O2–C2–C3–O3	1.63
C11–C10–N1–C20	0.17	N1–C10–C9–O4	-0.06
C1–C16–C13–C14	-126.94	C3–C4–C15–C5	-178.79
C4–C15–C5–C6	108.80	C15–C5–C6–C7	42.15
C5–C6–C7–N2	173.82	C5–C6–C7–C14	46.87
C6–C7–C14–C13	-75.71	C7–C14–C13–C16	1.04
C14–C13–C16–C15	54.68	C14–C7–N2–S1	130.07
C7–N2–S1–O5	-160.19	C7–N2–S1–O6	-28.79
C7–N2–S1–N3	82.85	N2–S1–N3–C21	68.32
N2–S1–N3–C22	-159.52		

(h) 19

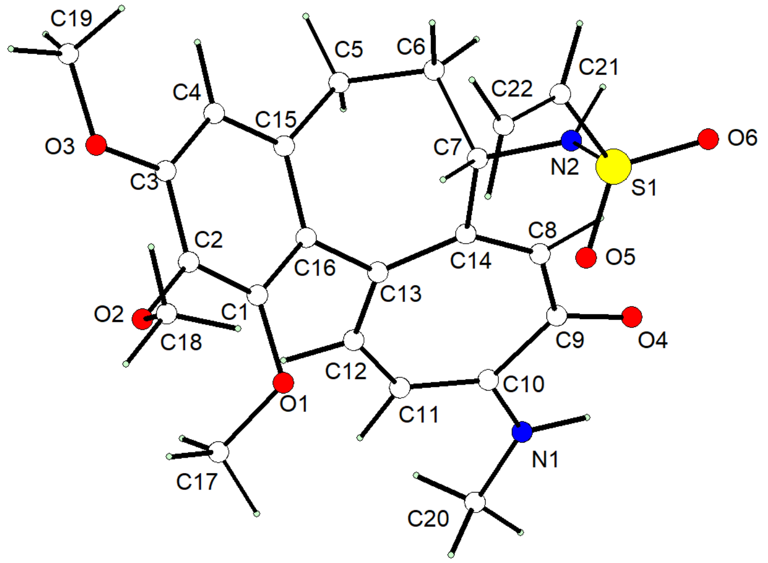

C1–O1	1.371	C2–O2	1.376	C3–O3	1.366
O1–C17	1.434	O2–C18	1.434	O3–C19	1.422
C10–N1	1.350	N1–C20	1.445	C9–O4	1.251
C7–N2	1.464	N2–S1	1.669	S1–O5	1.463
S1–O6	1.466	C1–C2	1.405	C2–C3	1.409
C3–C4	1.398	C4–C15	1.400	C15–C16	1.408
C1–C16	1.416	C15–C5	1.512	C5–C6	1.544
C6–C7	1.548	C7–C14	1.544	C14–C13	1.434
C13–C16	1.501	C14–C8	1.380	C8–C9	1.438
C9–C10	1.491	C10–C11	1.391	C11–C12	1.406
C12–C13	1.390				
C1–O1–C17	118.62	C2–O2–C18	115.06	C3–O3–C19	118.45
C1–C16–C13	119.10	O1–C1–C2	120.82	O2–C2–C3	120.34
O3–C3–C4	124.65	C4–C15–C5	120.35	C15–C5–C6	111.79
C5–C6–C7	113.43	C6–C7–C14	111.45	C7–C14–C13	115.27
C14–C13–C16	119.50	C14–C8–C9	134.49	C8–C9–C10	123.26
C8–C9–O4	120.15	O4–C9–C10	116.59	C9–C10–N1	111.38
C10–N1–C20	126.29	N1–C10–C11	122.17	C10–C11–C12	130.47
C11–C12–C13	132.81	C12–C13–C14	124.33	C12–C13–C16	116.15
C6–C7–N2	108.85	C7–N2–S1	122.96	N2–S1–O5	107.60
N2–S1–O6	104.90	N2–S1–C21	105.83	S1–C21–C22	121.75

C1–C16–C13–C12	55.98	C17–O1–C1–C2	59.22
C18–O2–C2–C3	-77.18	C19–O3–C3–C4	3.10
O1–C1–C2–O2	-4.56	O2–C2–C3–O3	0.04
C11–C10–N1–C20	-1.61	N1–C10–C9–O4	1.44
C1–C16–C13–C14	-125.41	C3–C4–C15–C5	-177.15
C4–C15–C5–C6	108.09	C15–C5–C6–C7	42.68
C5–C6–C7–N2	173.52	C5–C6–C7–C14	46.61
C6–C7–C14–C13	-75.46	C7–C14–C13–C16	0.65
C14–C13–C16–C15	55.49	C14–C7–N2–S1	108.99
C7–N2–S1–O5	46.52	C7–N2–S1–O6	179.05
C7–N2–S1–C21	-68.32	N2–S1–C21–C22	112.70
O5–S1–C21–C22	-2.12	O6–S1–C21–C22	-135.94

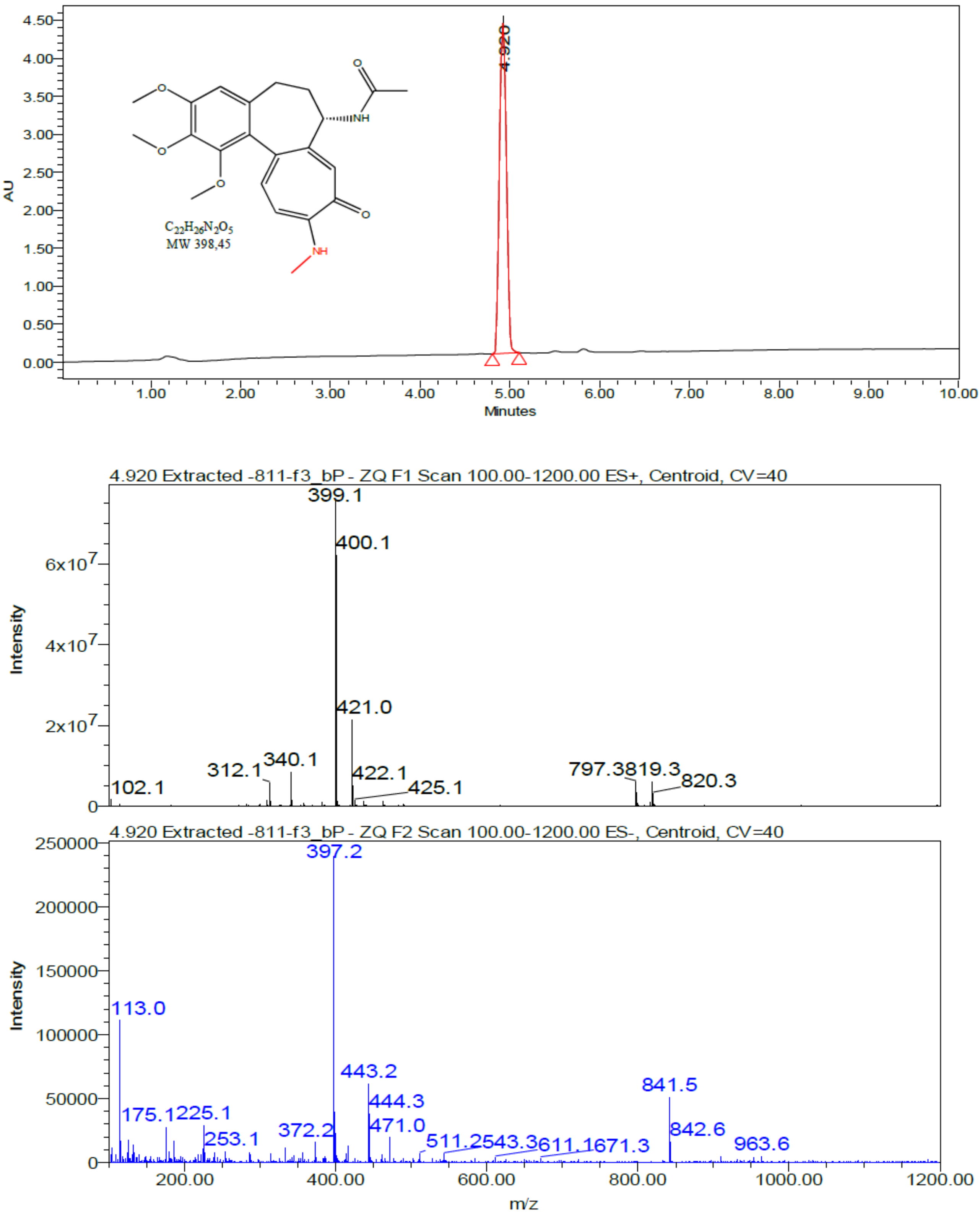

Figure S1. The LC-MS chromatogram and mass spectra of **2**.

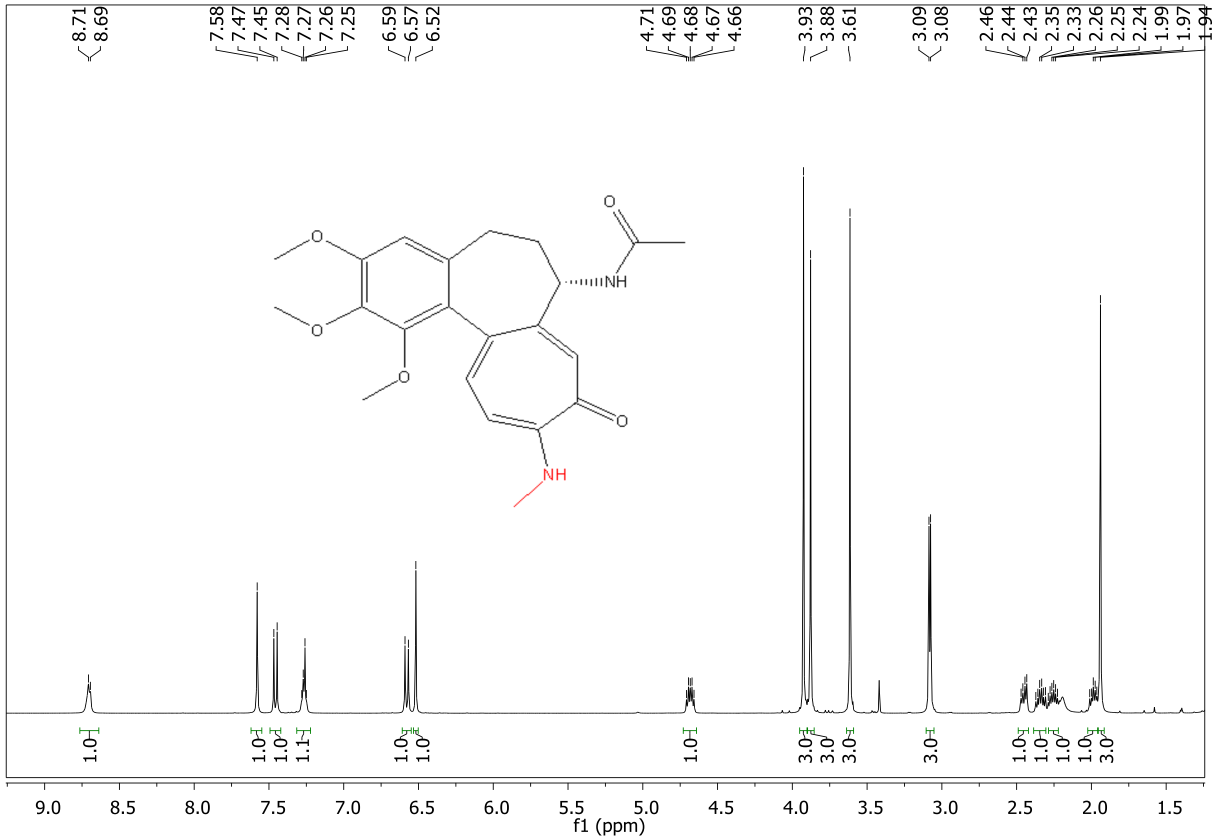

Figure S2. The ^{1}H NMR spectrum of **2** in $CDCl_3$.

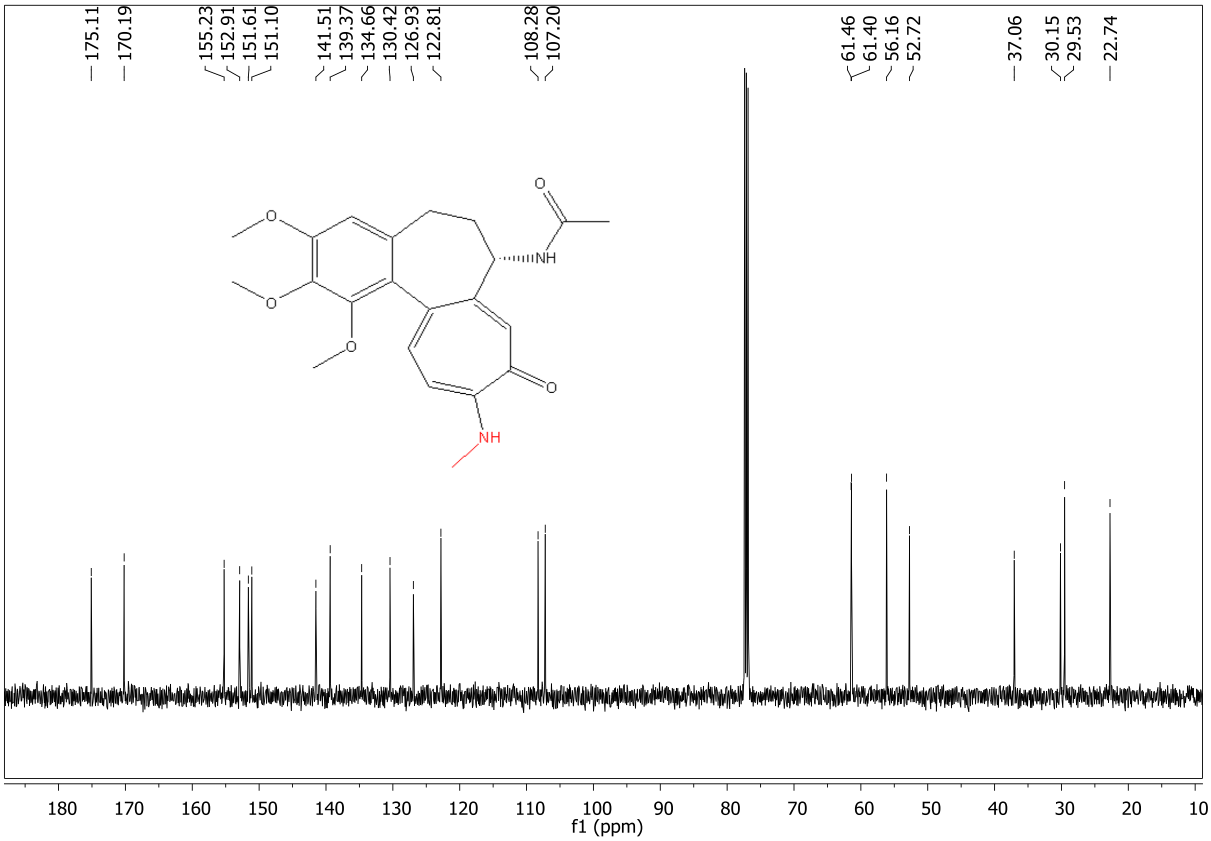

Figure S3. The ^{13}C NMR spectrum of **2** in $CDCl_3$.

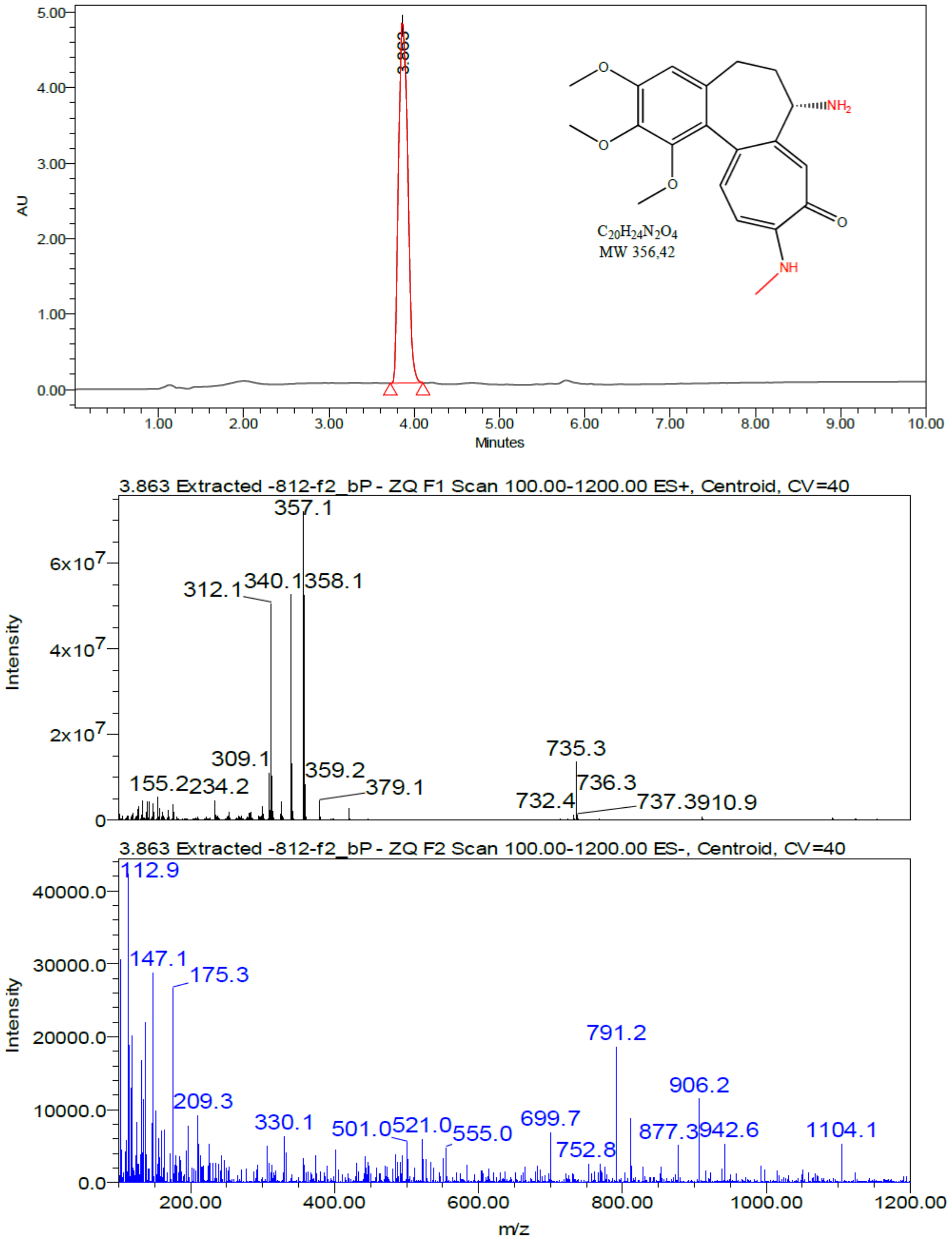

Figure S4. The LC-MS chromatogram and mass spectra of **3**.

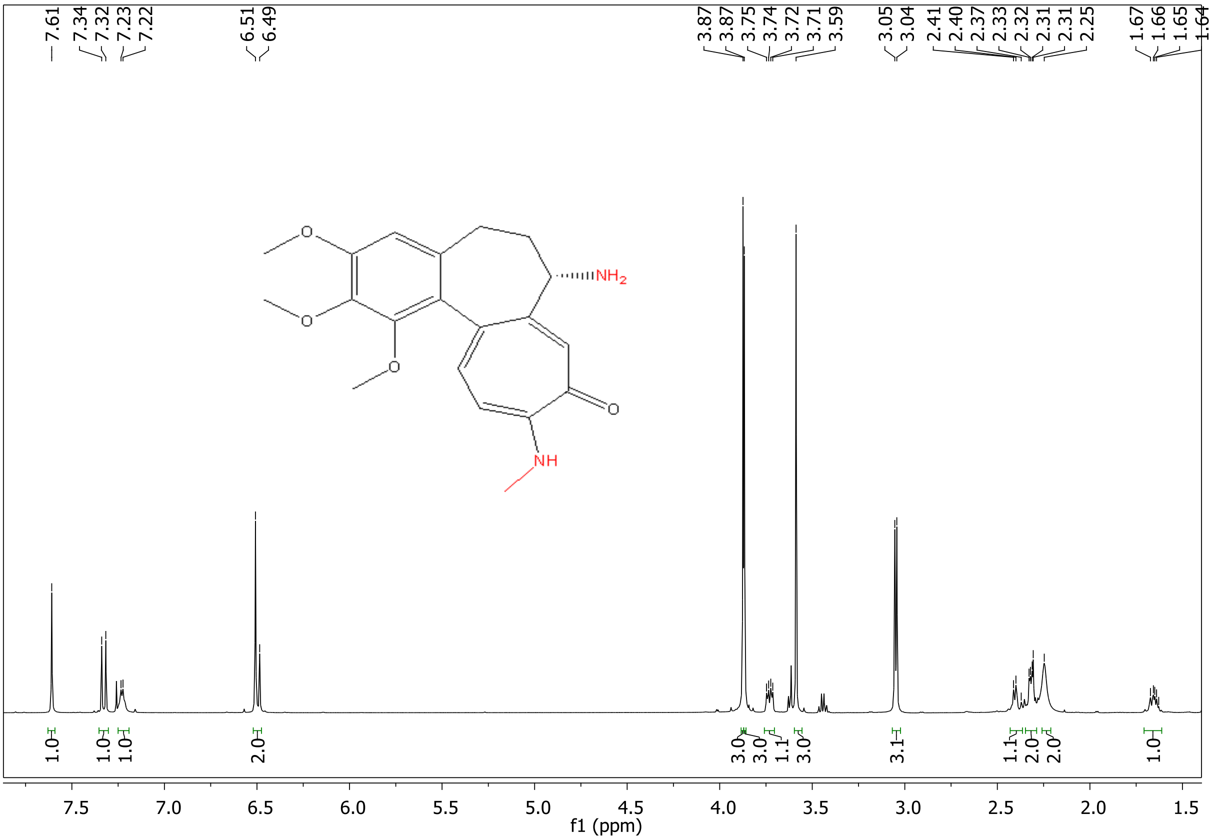

Figure S5. The ^{1}H NMR spectrum of **3** in $CDCl_3$.

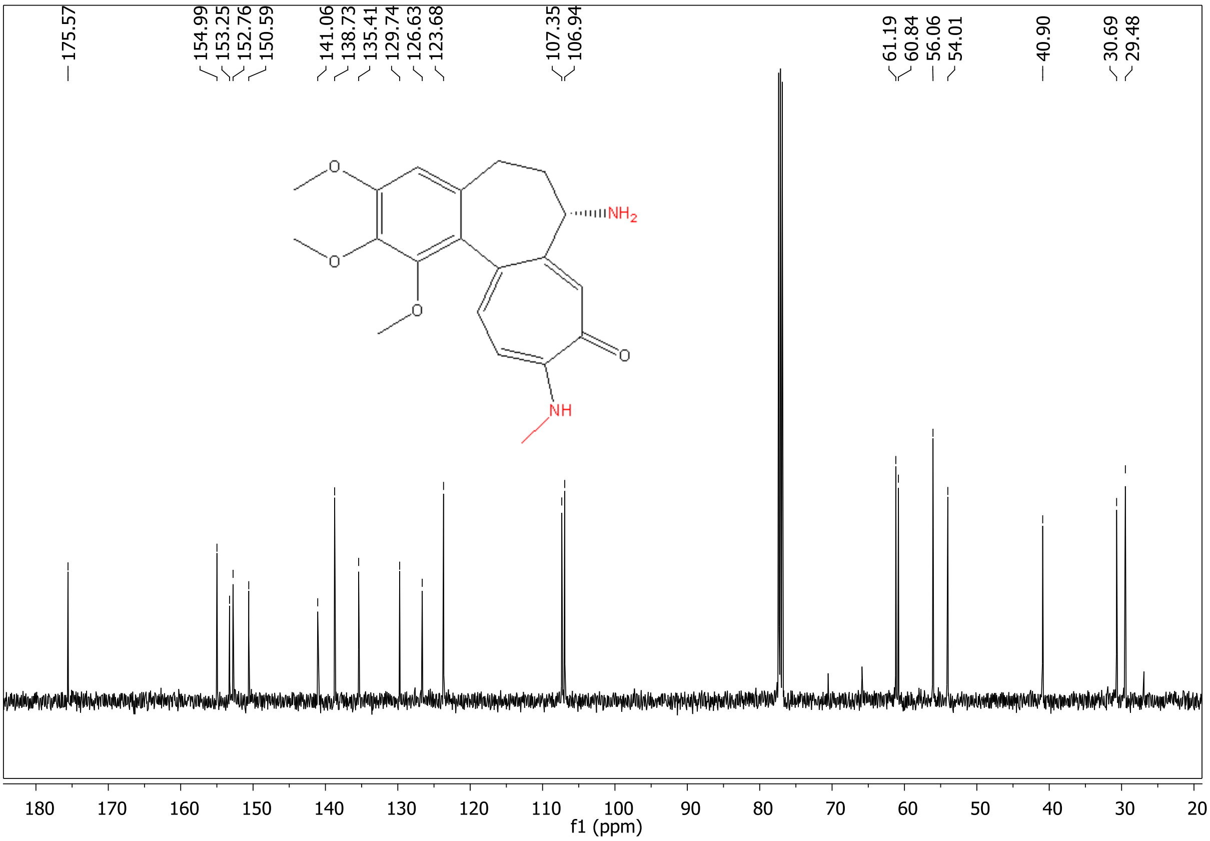

Figure S6. The ^{13}C NMR spectrum of **3** in $CDCl_3$.

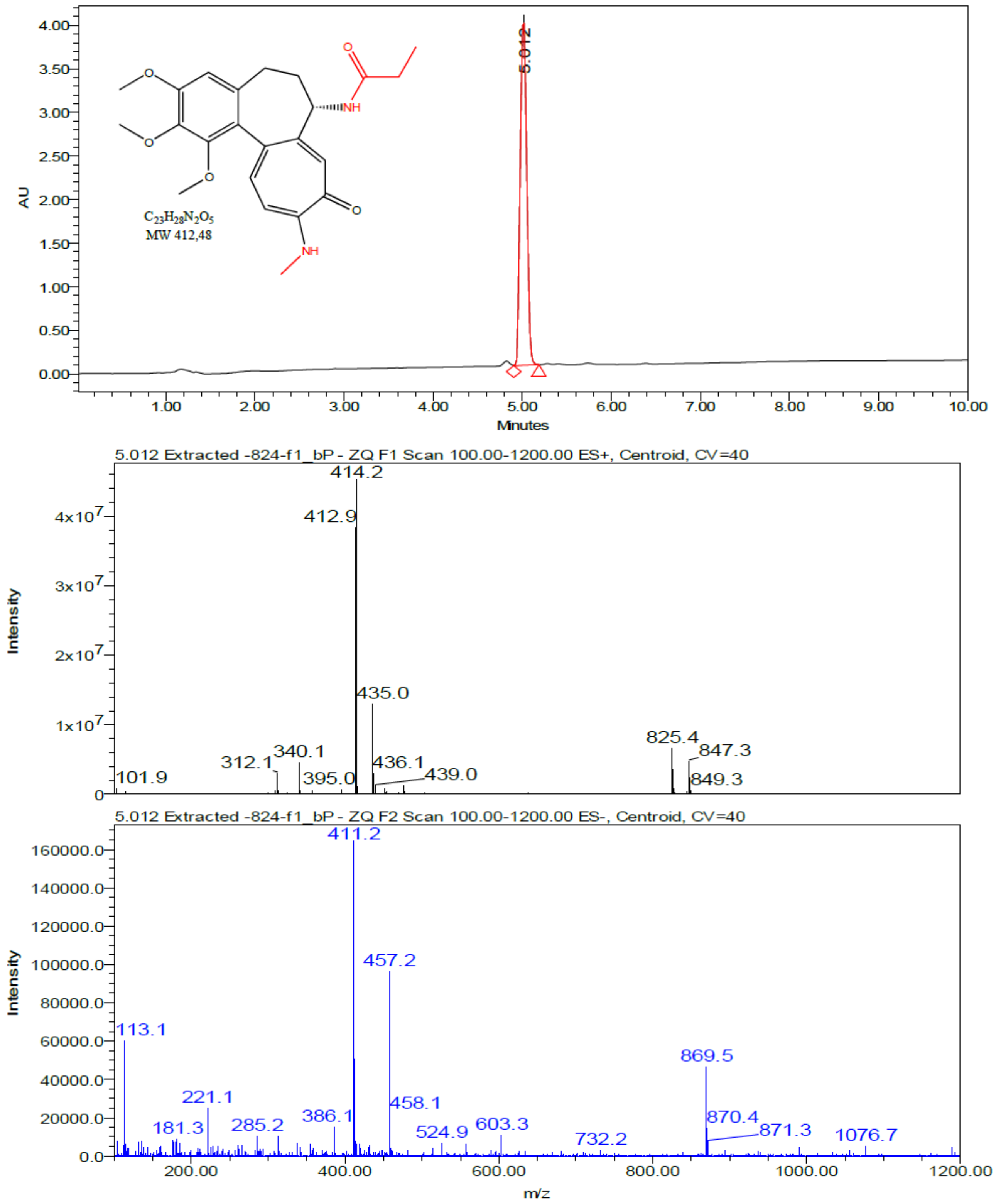

Figure S7. The LC-MS chromatogram and mass spectra of **4**.

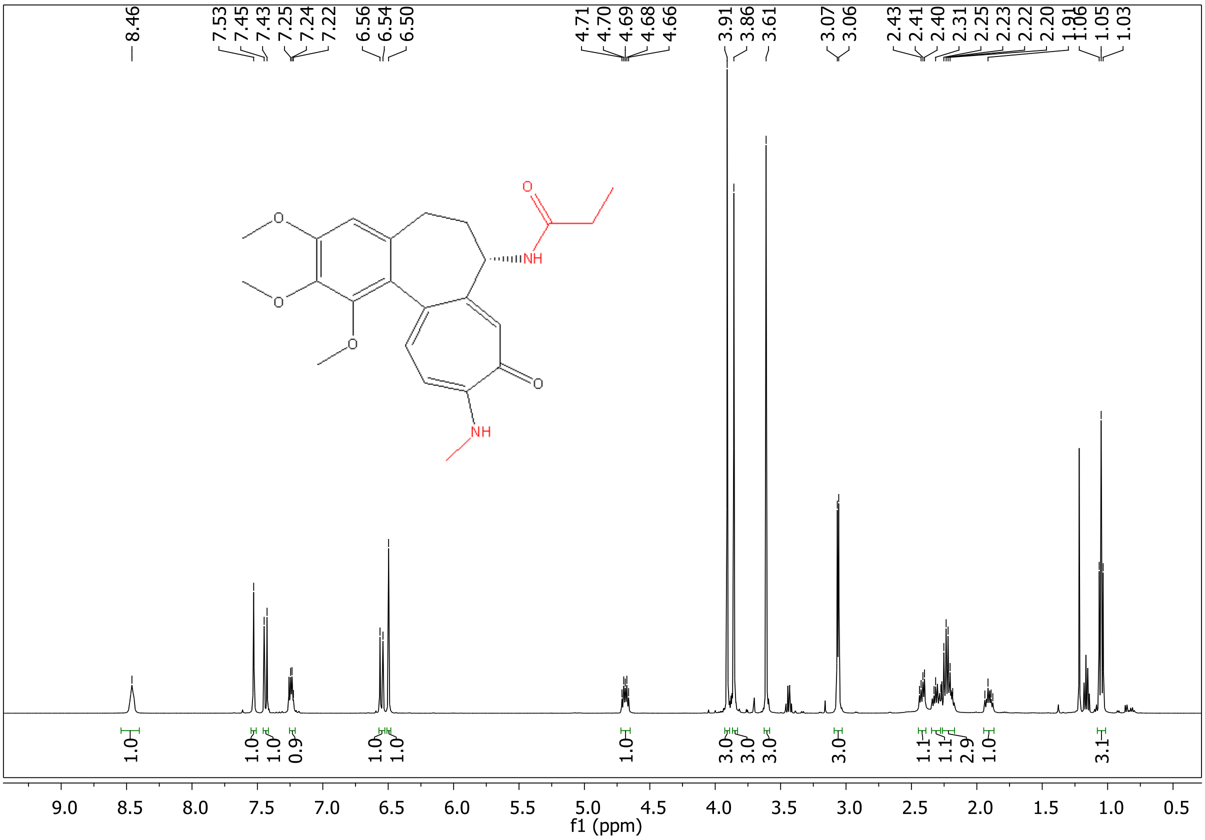

Figure S8. The ^{1}H NMR spectrum of **4** in $CDCl_3$.

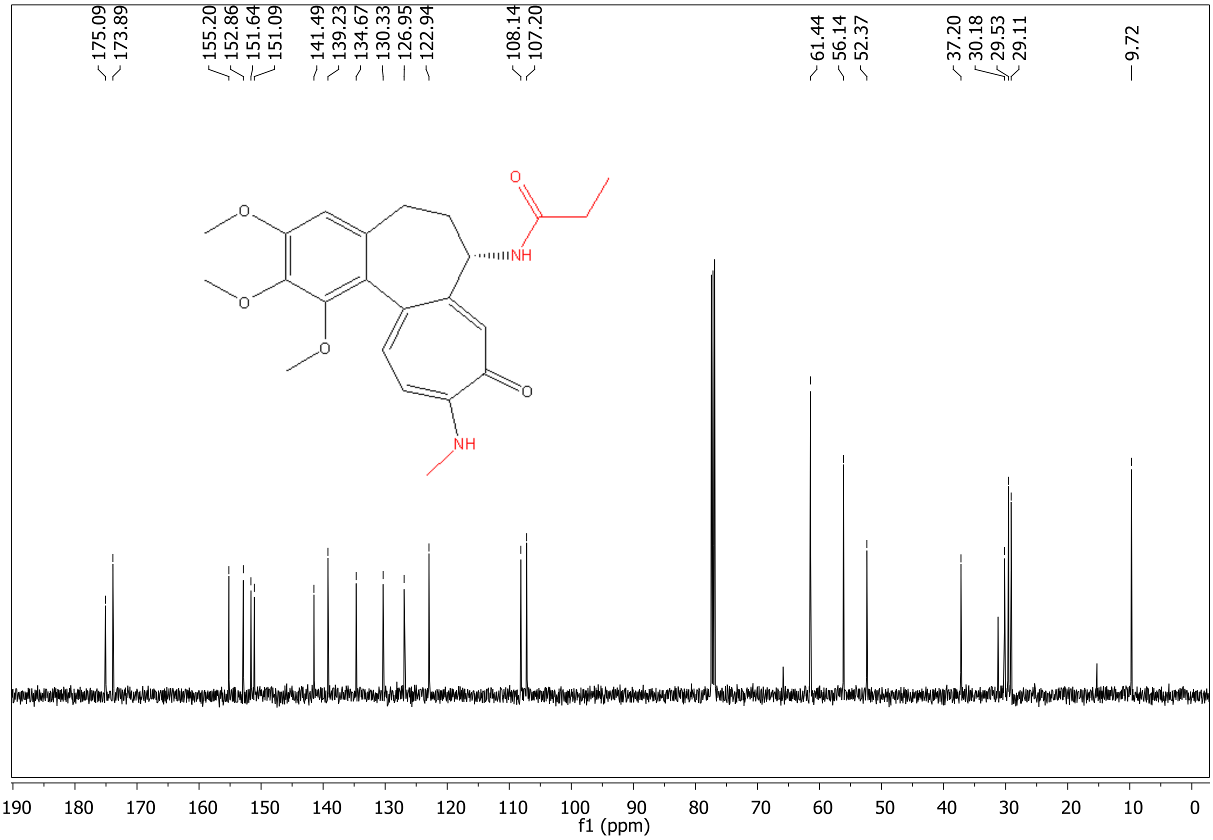

Figure S9. The ^{13}C NMR spectrum of **4** in $CDCl_3$.

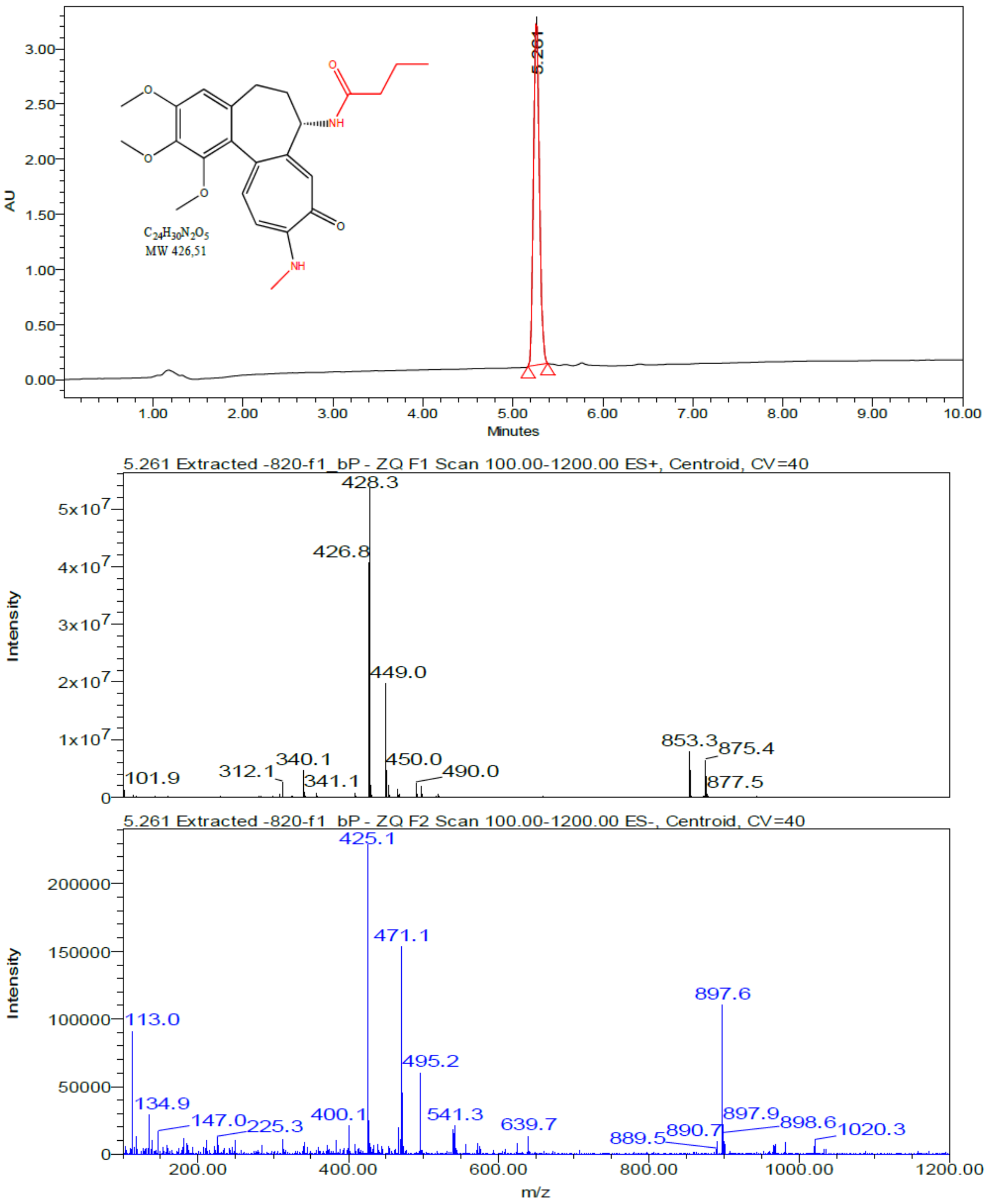

Figure S10. The LC-MS chromatogram and mass spectra of **5**.

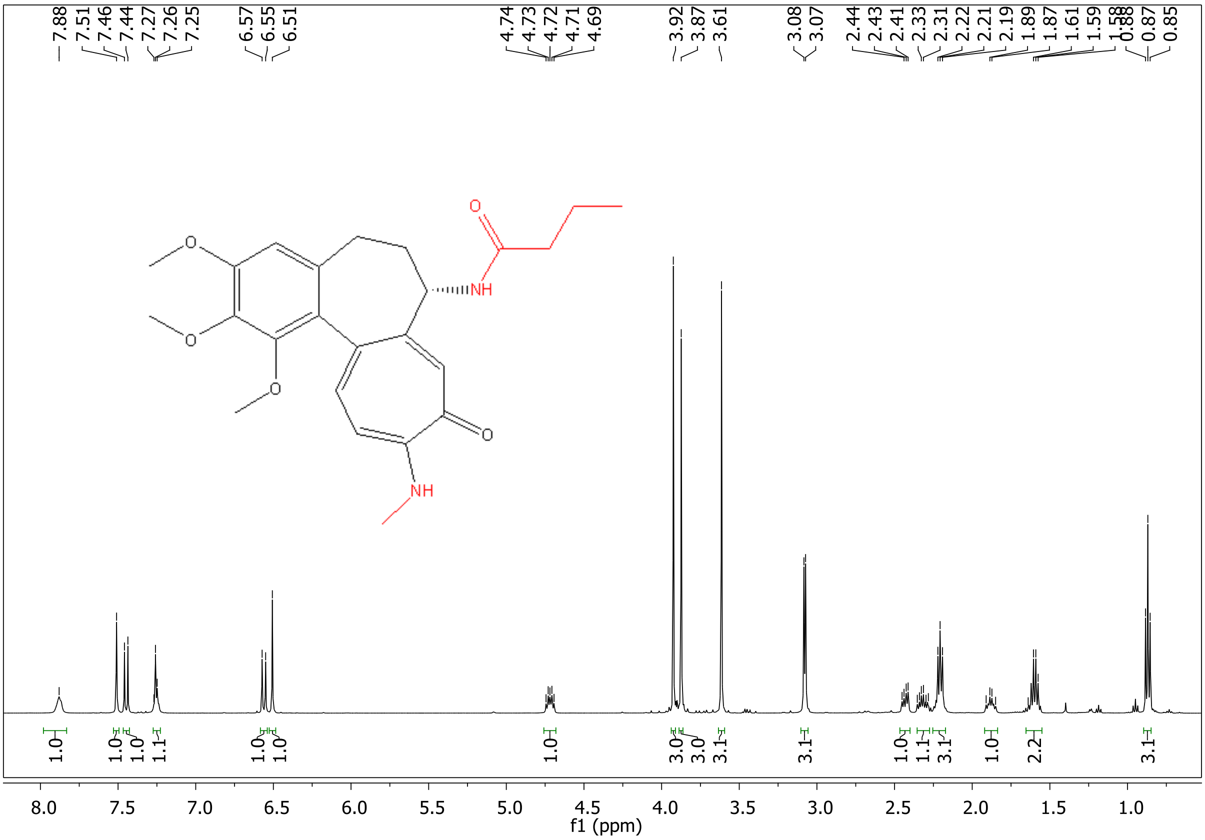

Figure S11. The ^{1}H NMR spectrum of **5** in $CDCl_3$.

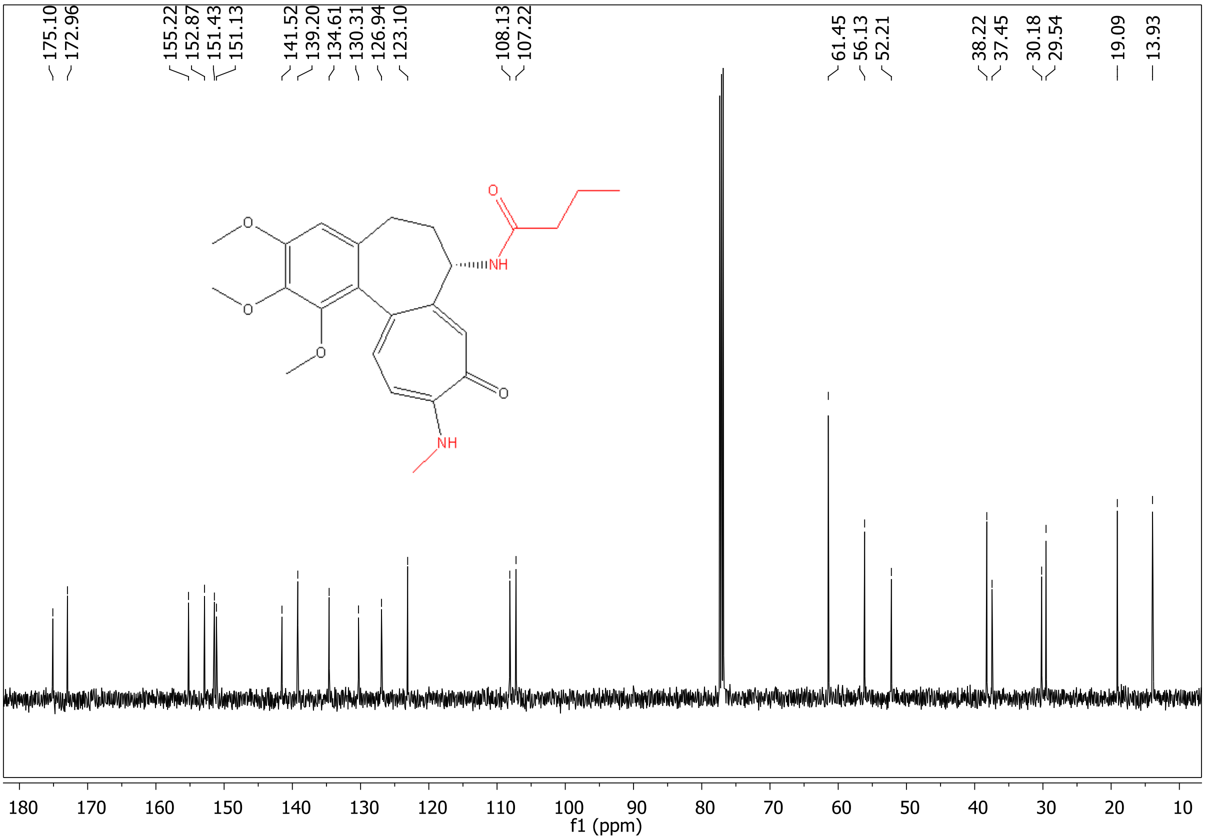

Figure S12. The ^{13}C NMR spectrum of **5** in $CDCl_3$.

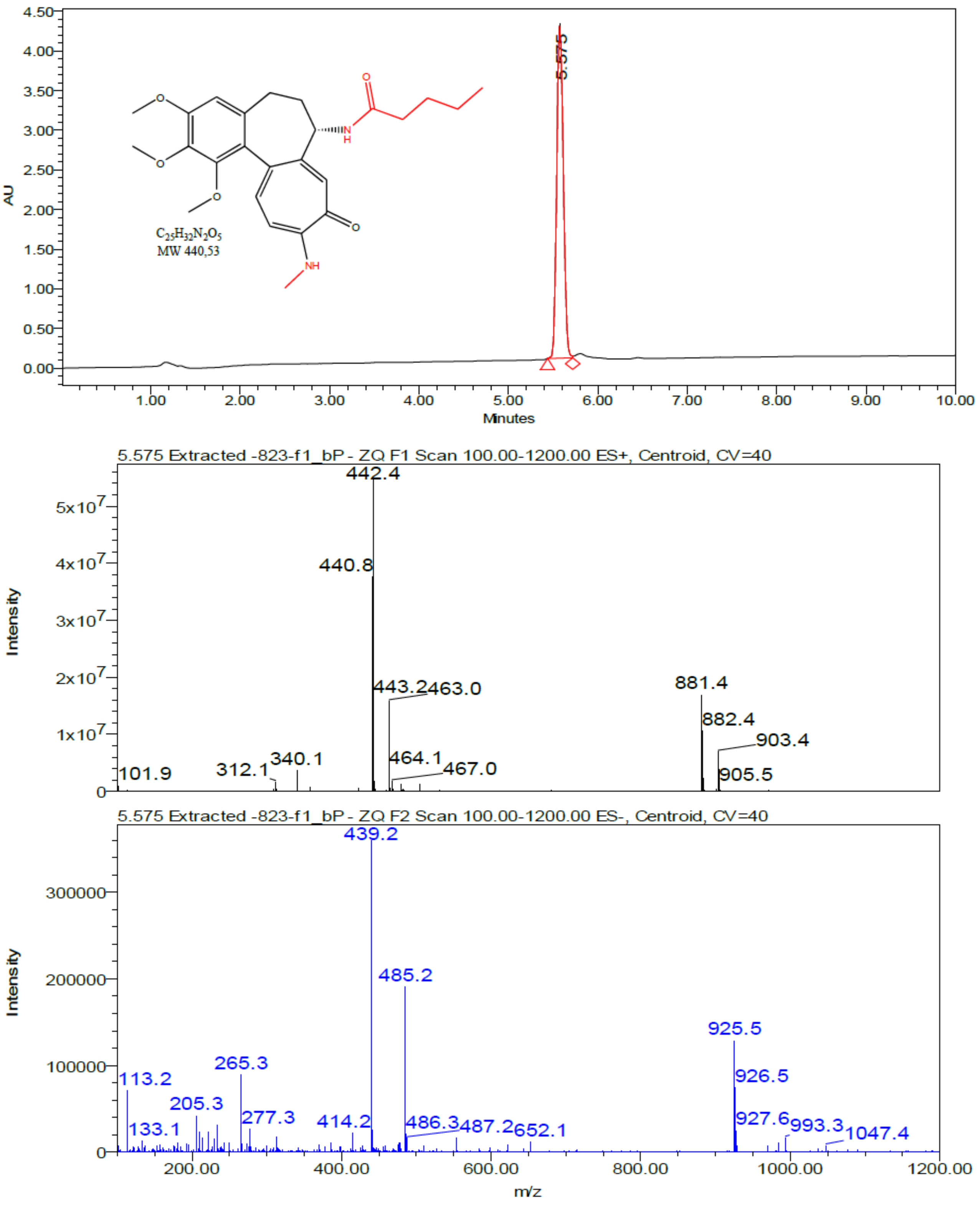

Figure S13. The LC-MS chromatogram and mass spectra of **6**.

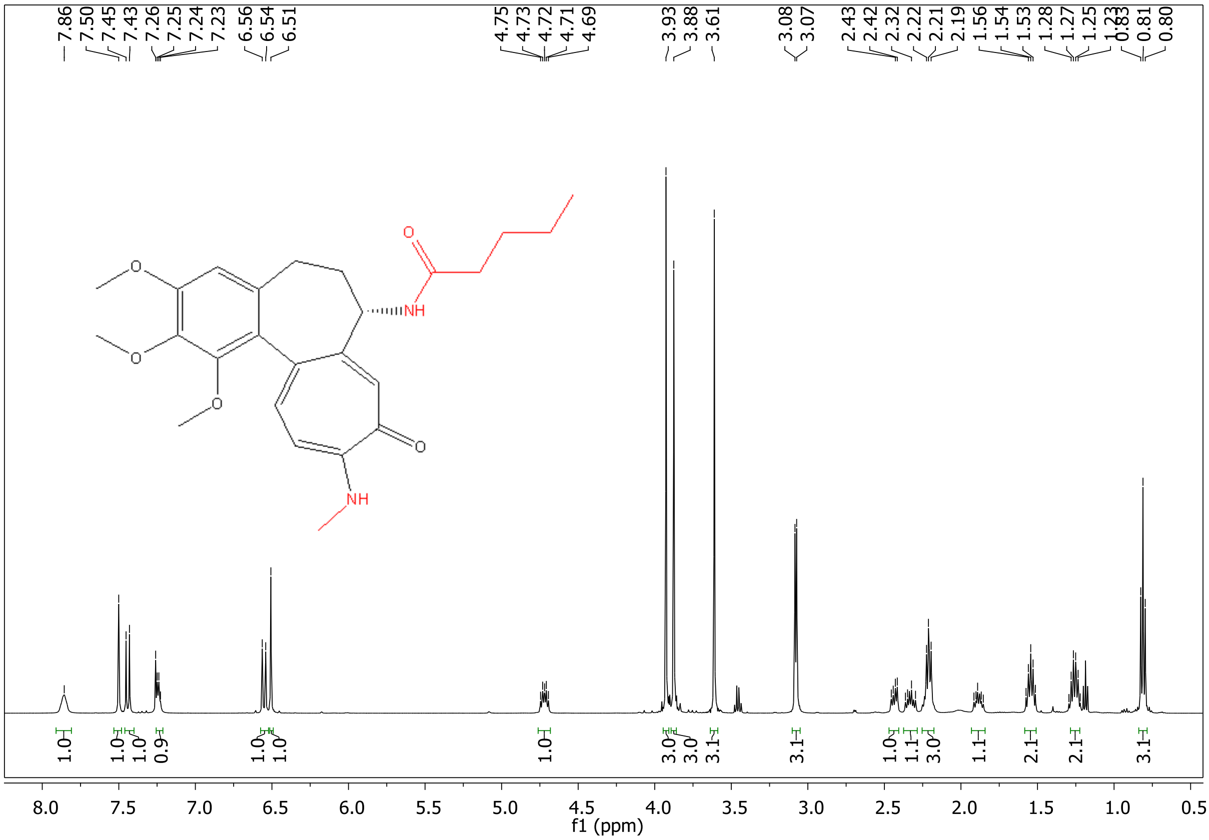

Figure S14. The ^{1}H NMR spectrum of **6** in $CDCl_3$.

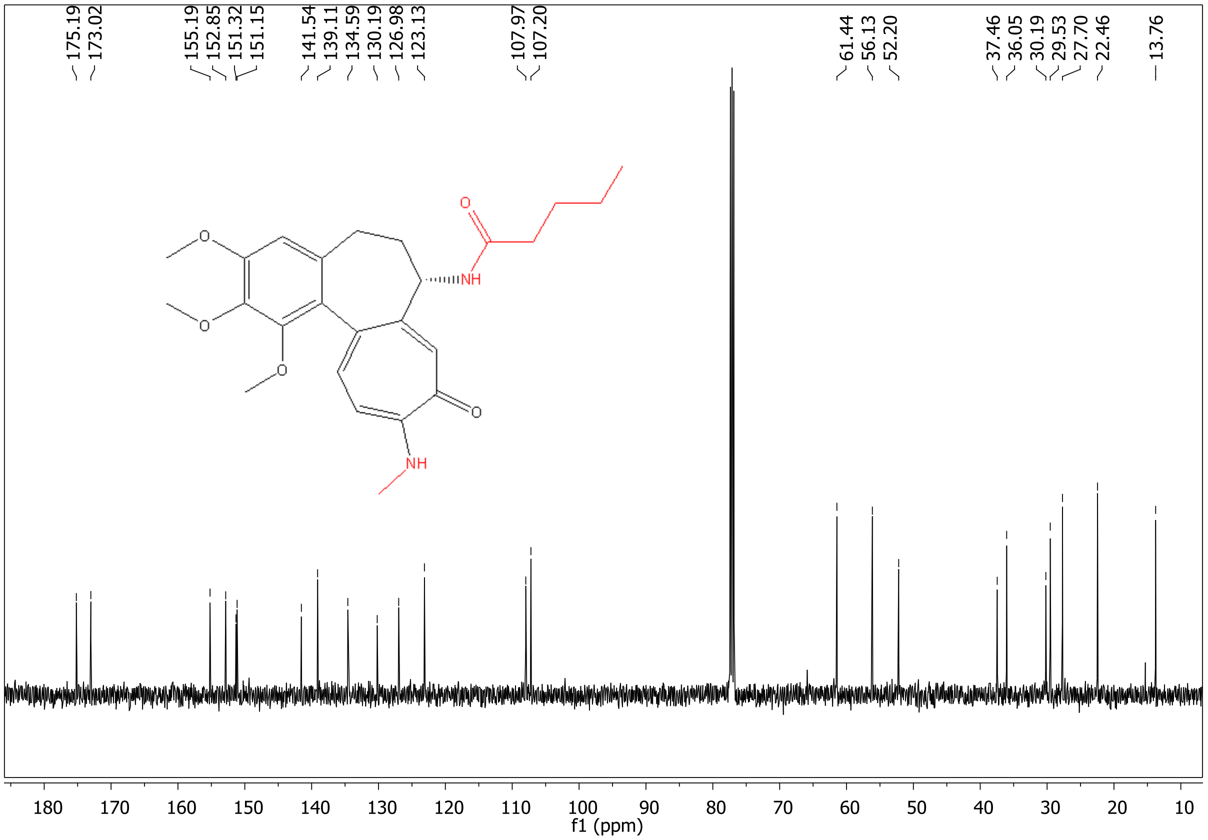

Figure S15. The ^{13}C NMR spectrum of **6** in $CDCl_3$.

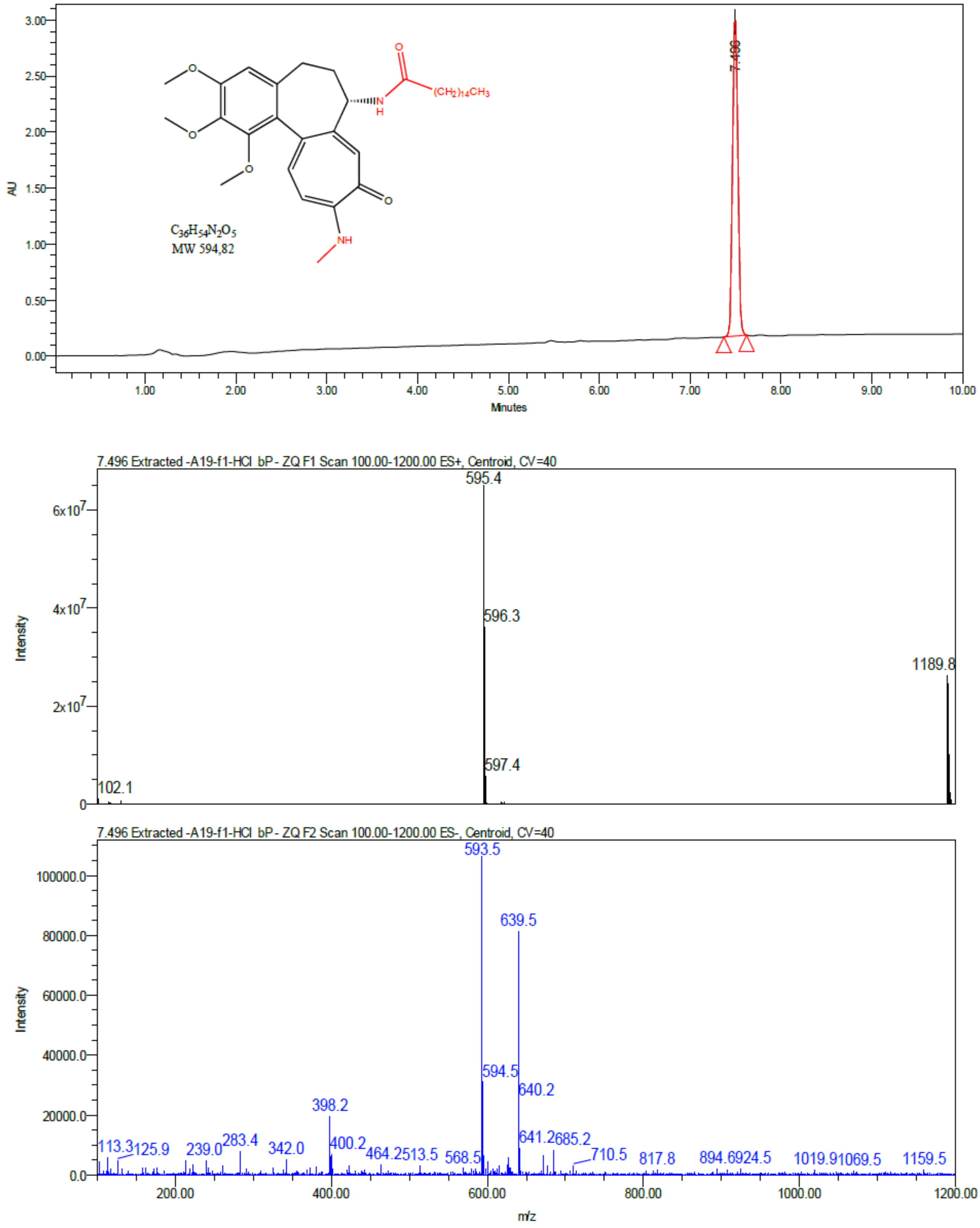

Figure S16. The LC-MS chromatogram and mass spectra of **7**.

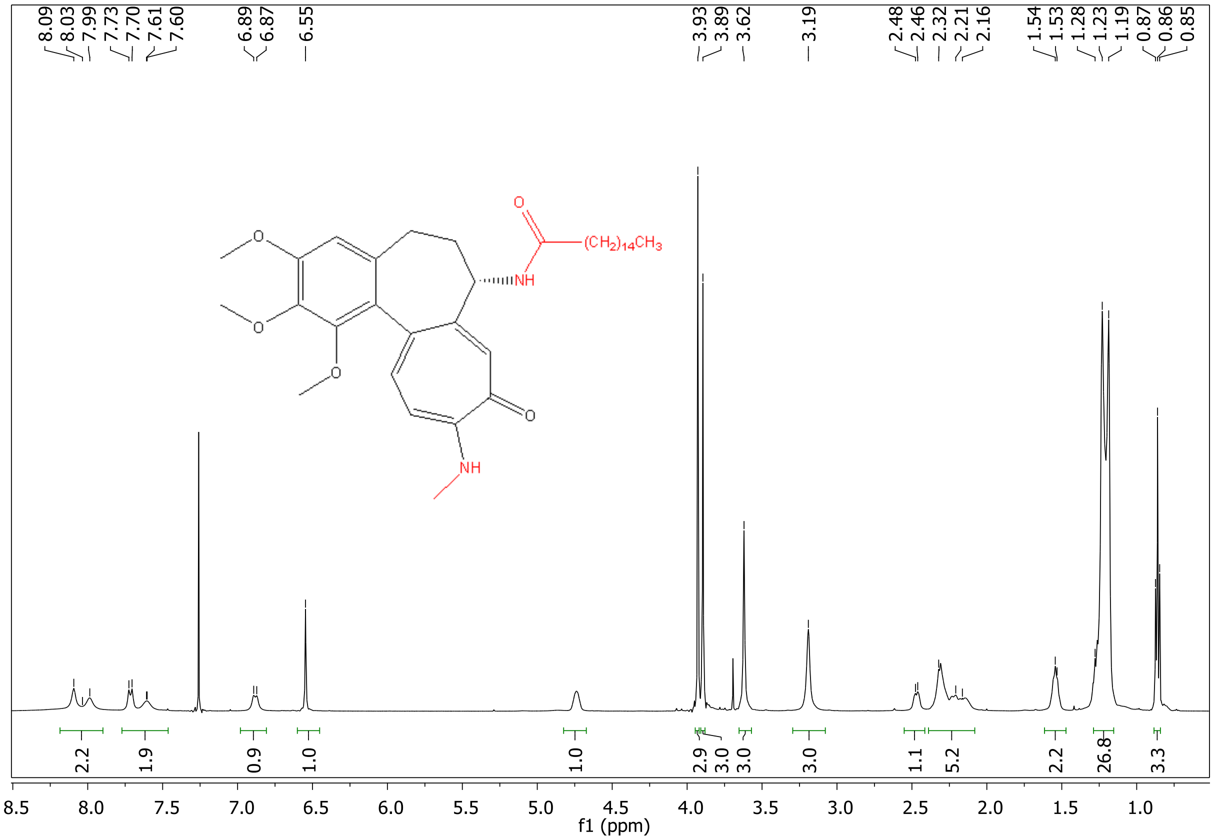

Figure S17. The ^{1}H NMR spectrum of **7** in $CDCl_3$.

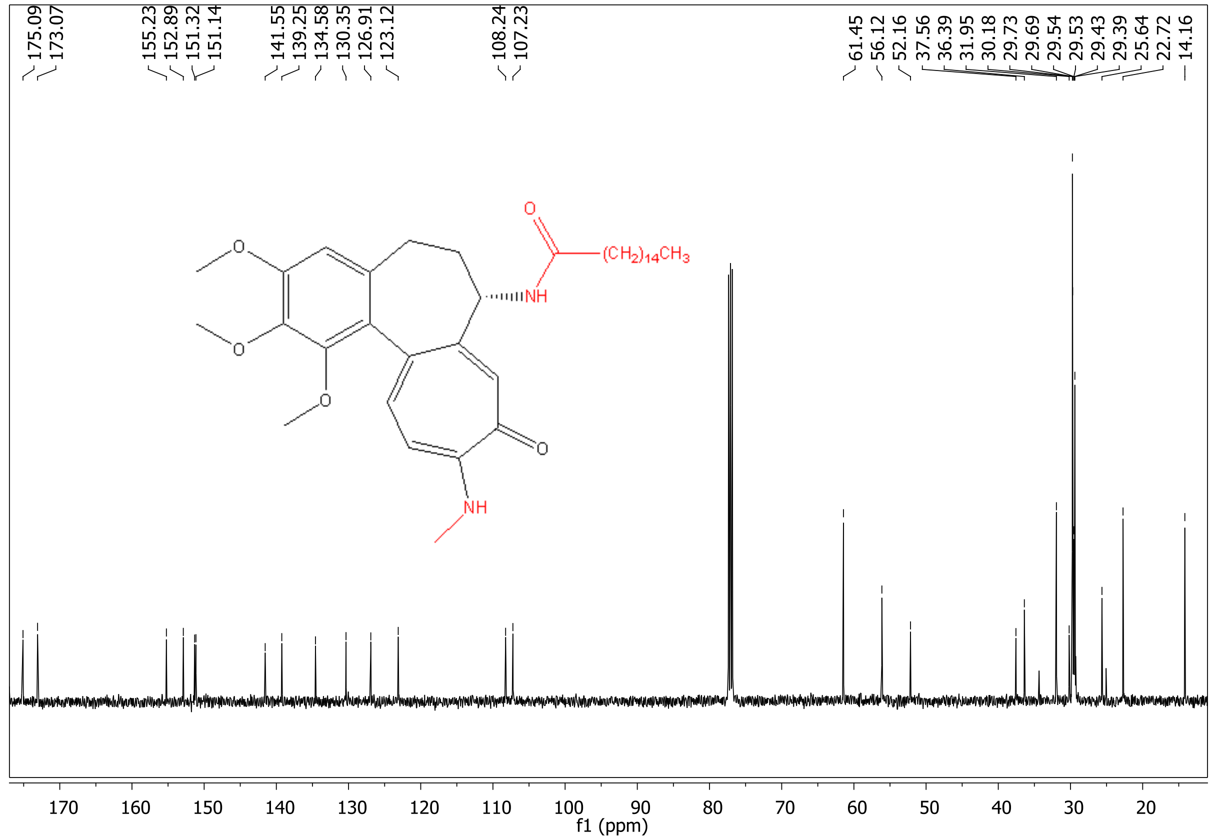

Figure S18. The ^{13}C NMR spectrum of **7** in $CDCl_3$.

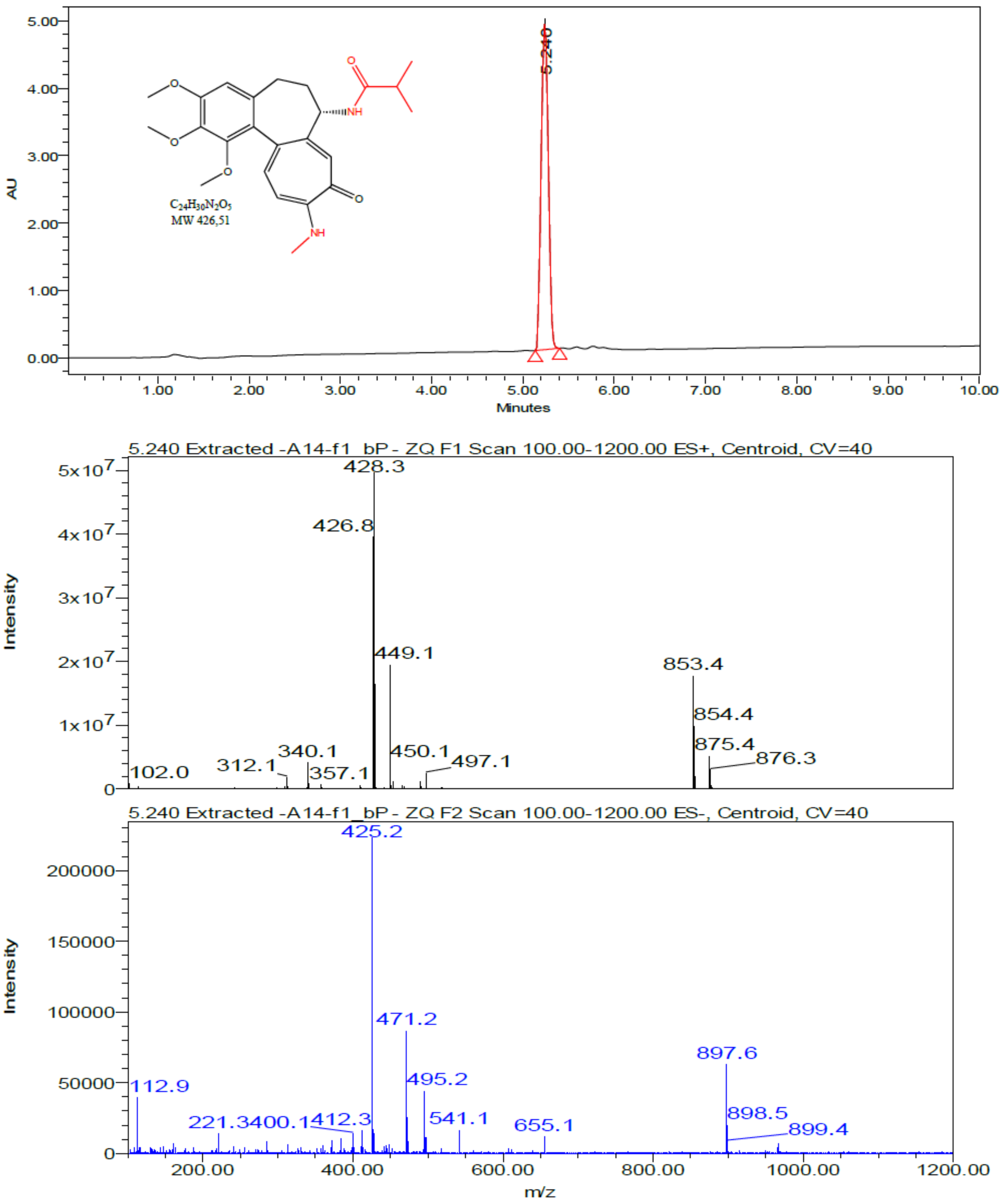

Figure S19. The LC-MS chromatogram and mass spectra of **8**.

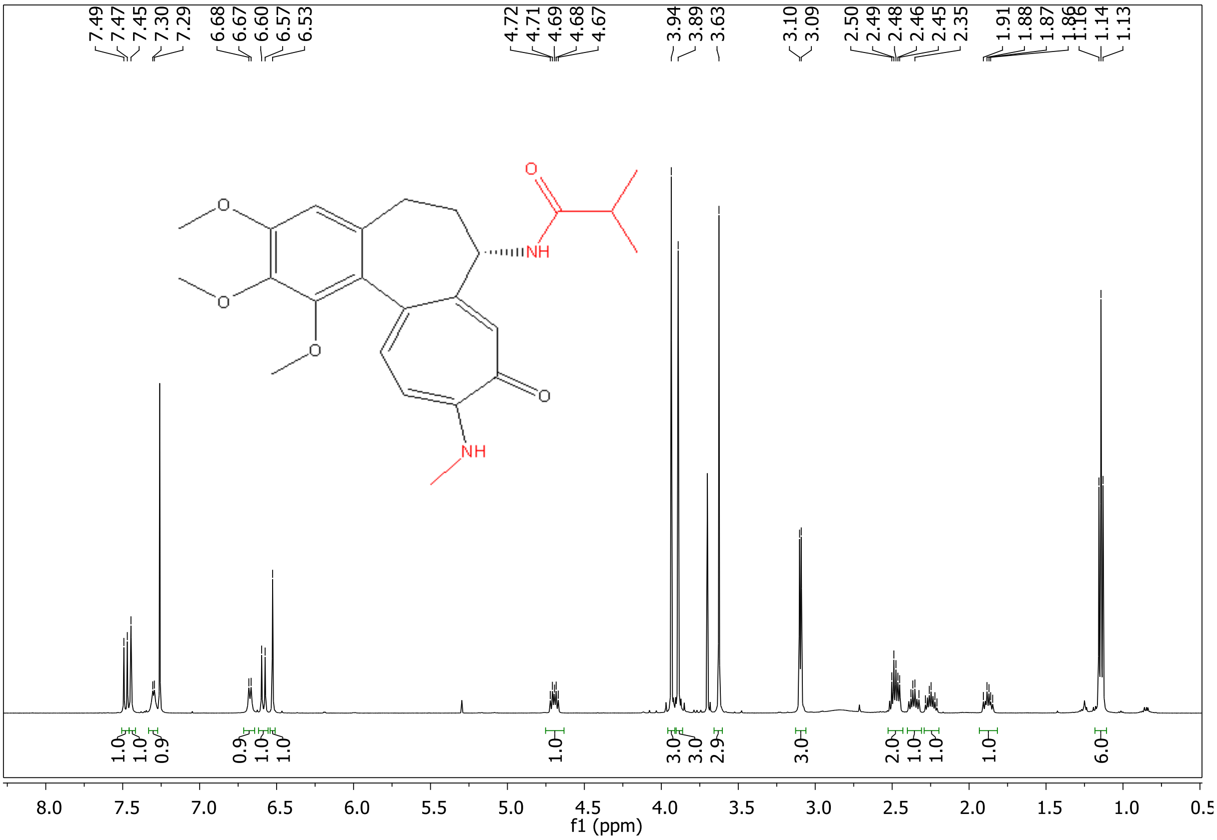

Figure S20. The ^{1}H NMR spectrum of **8** in $CDCl_3$.

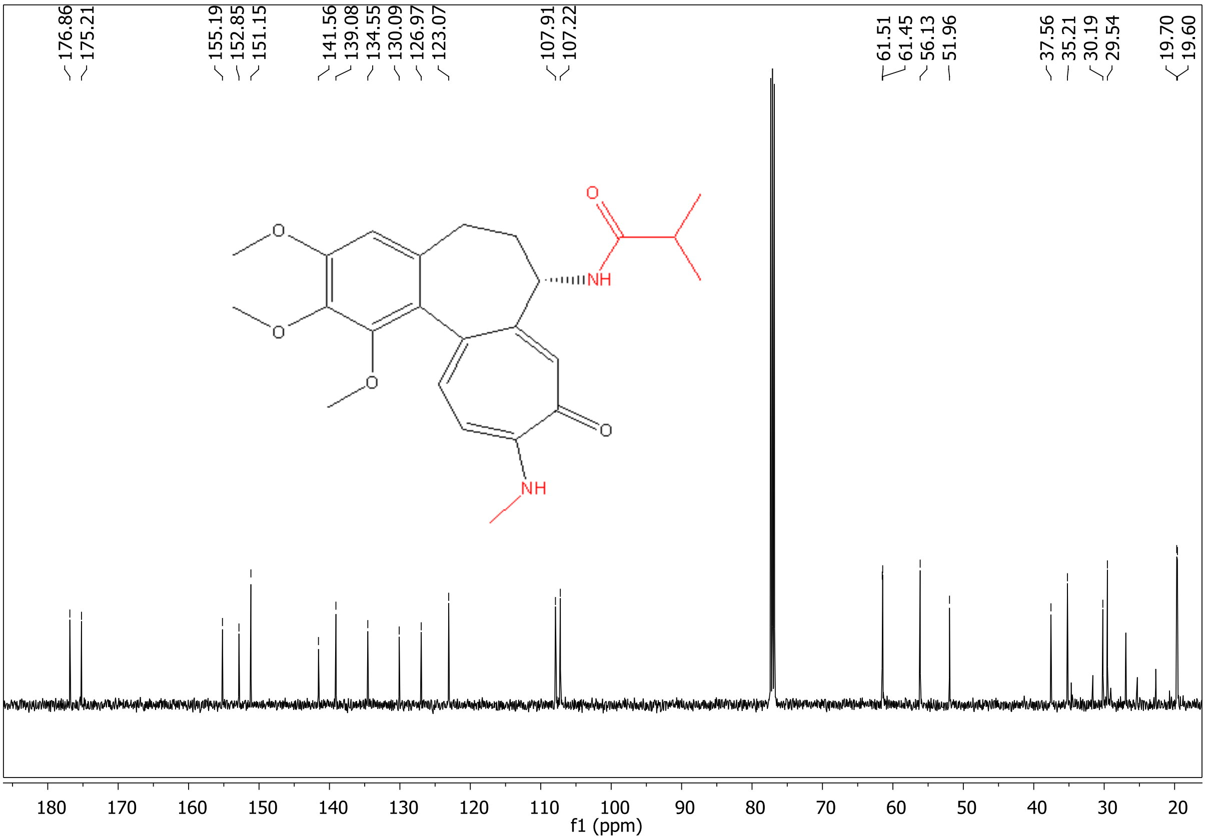

Figure S21. The ^{13}C NMR spectrum of **8** in $CDCl_3$.

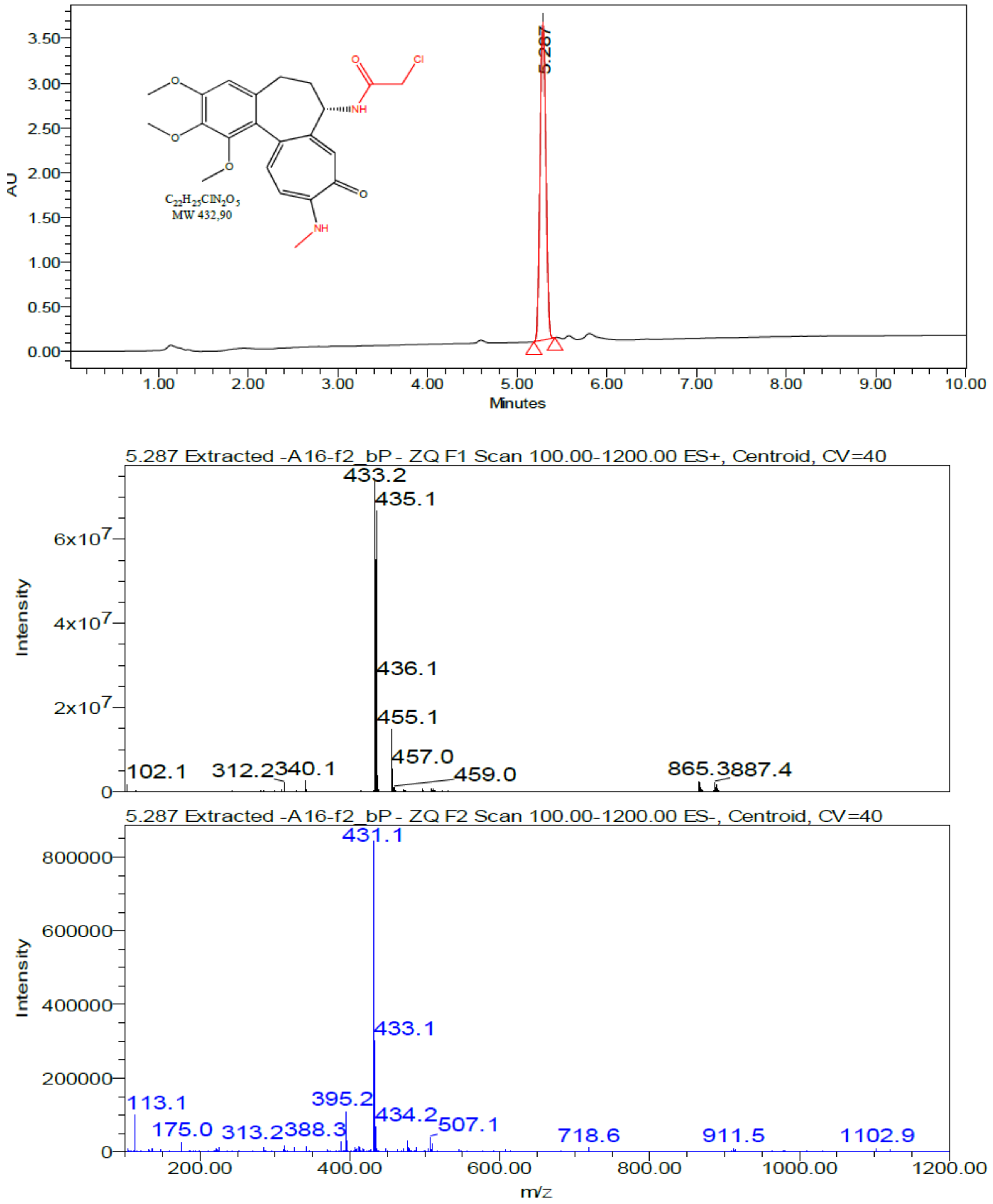

Figure S22. The LC-MS chromatogram and mass spectra of **9**.

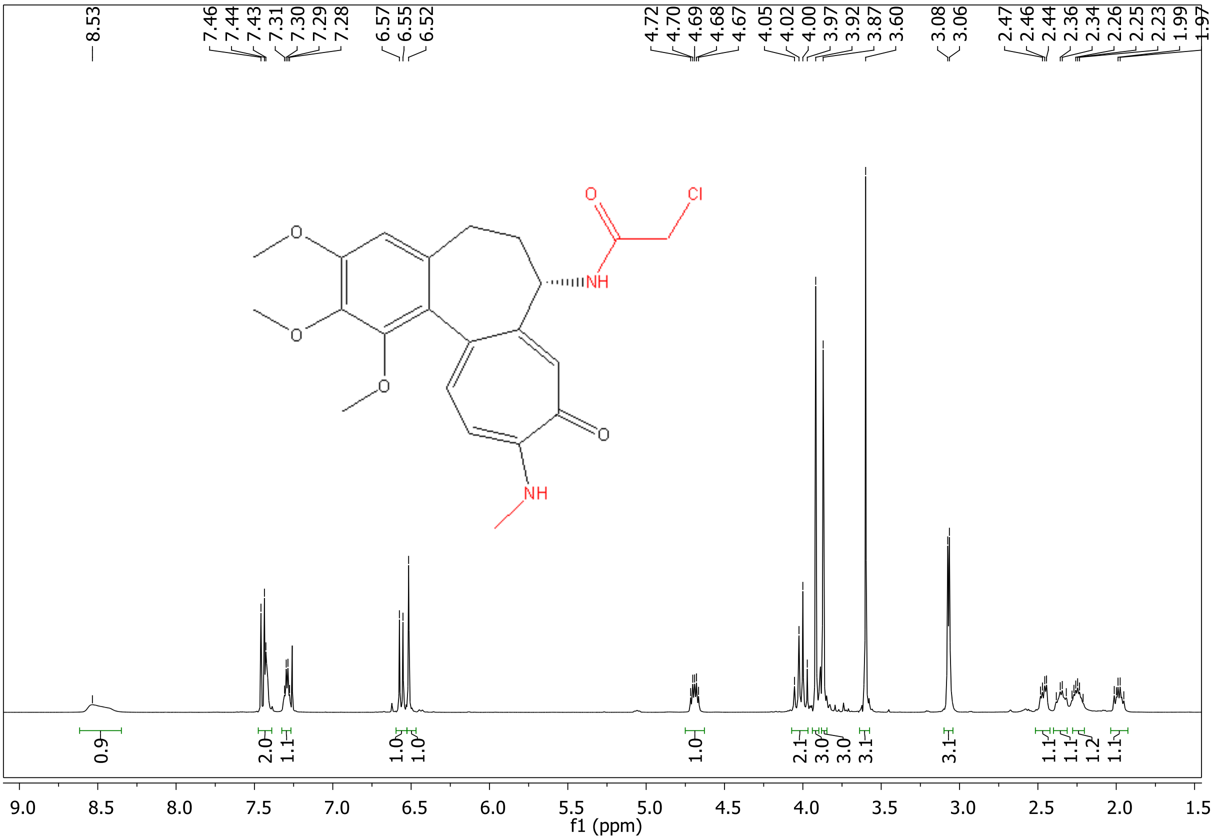

Figure S23. The ^{1}H NMR spectrum of **9** in $CDCl_3$.

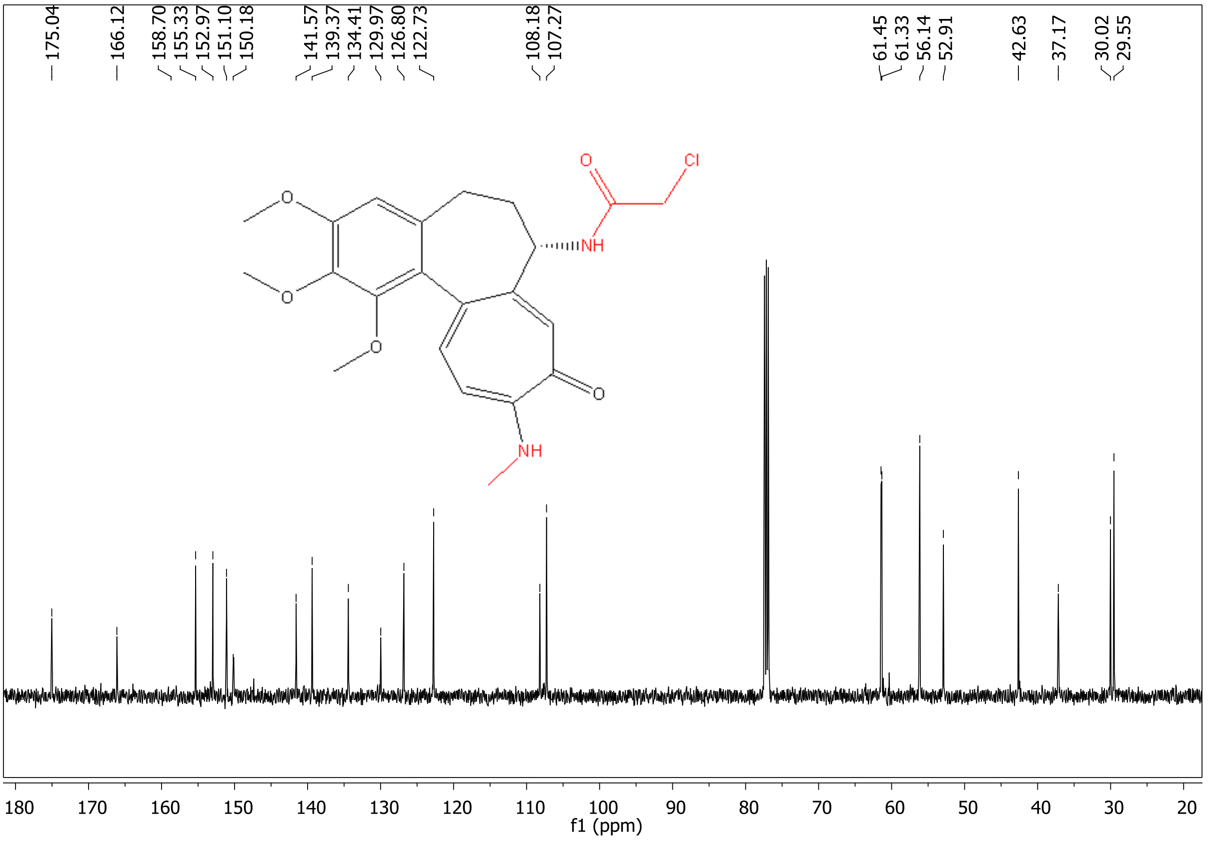

Figure S24. The ^{13}C NMR spectrum of **9** in $CDCl_3$.

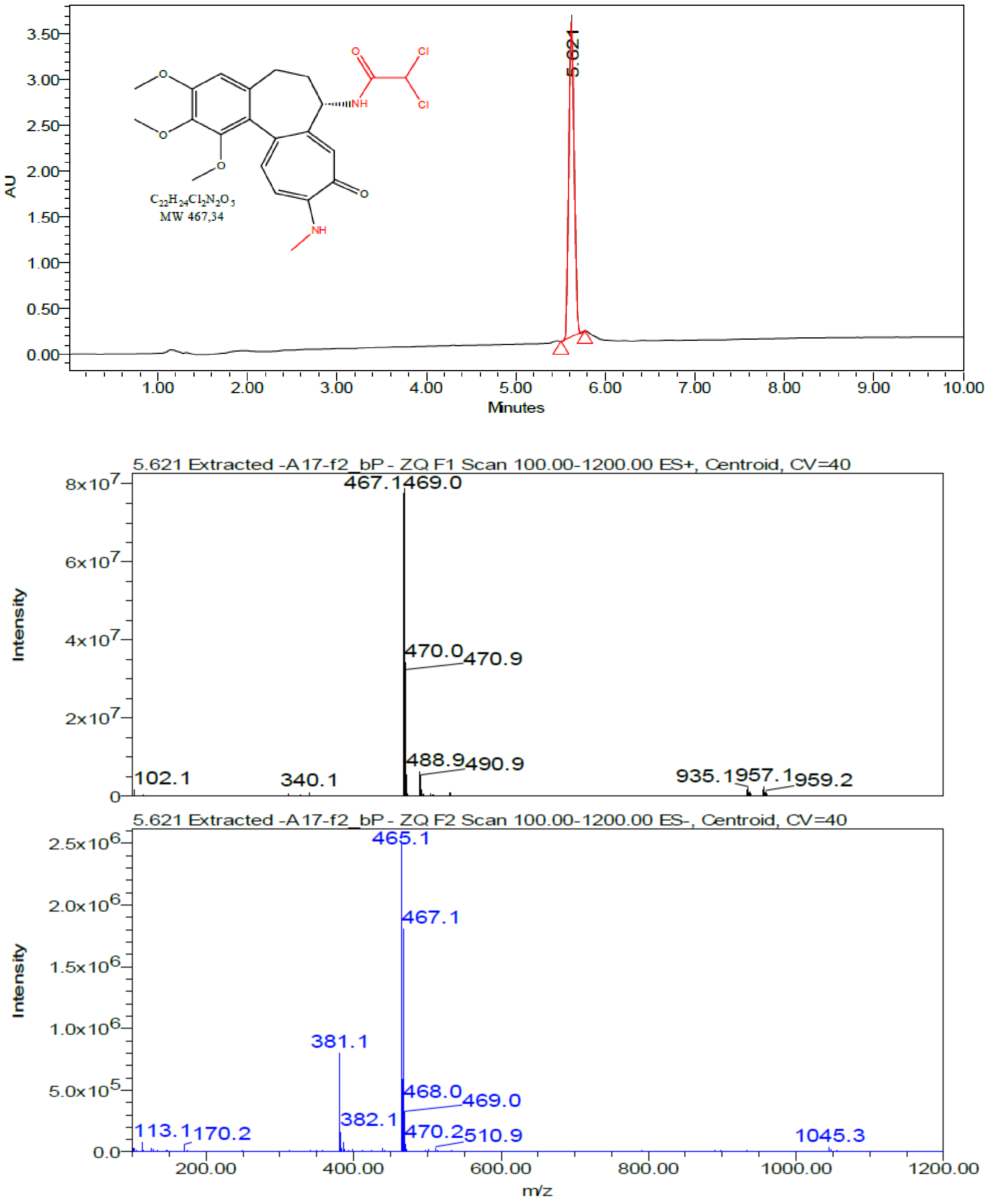

Figure S25. The LC-MS chromatogram and mass spectra of **10**.

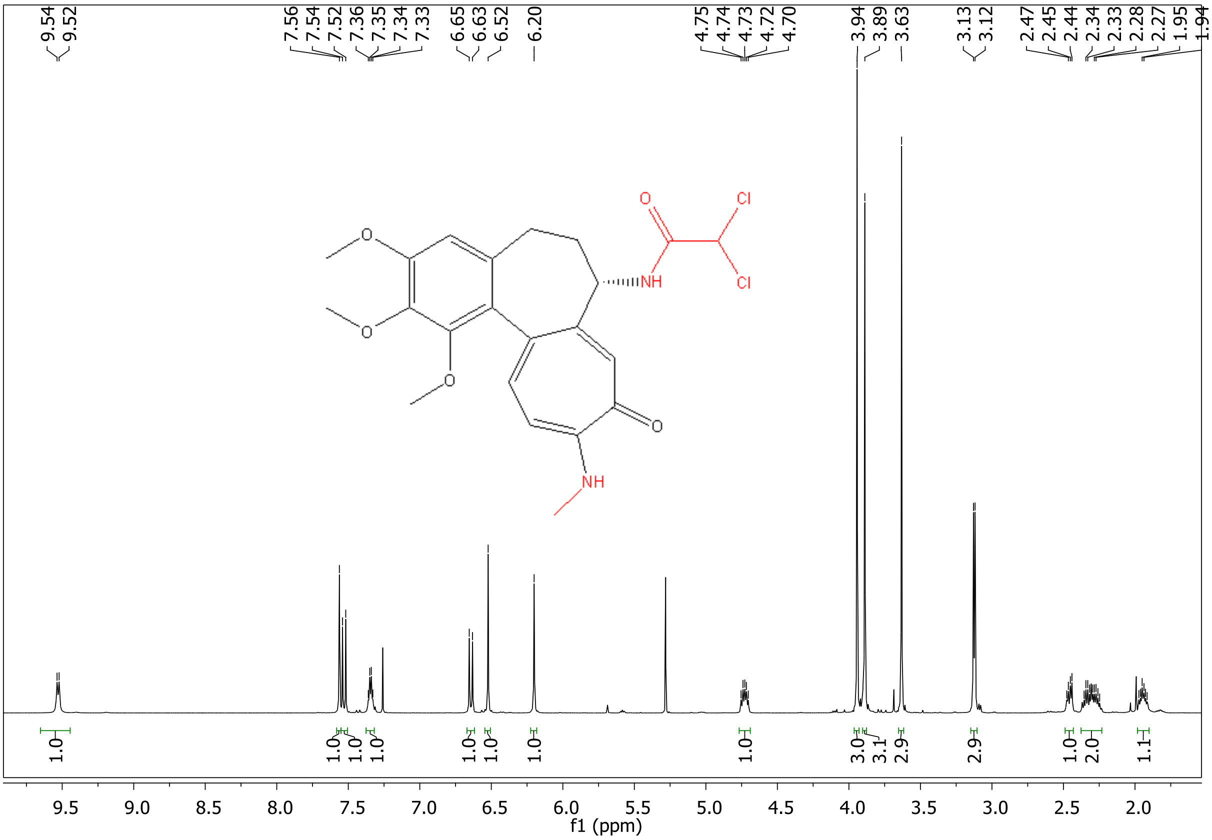

Figure S26. The ^{1}H NMR spectrum of **10** in $CDCl_3$.

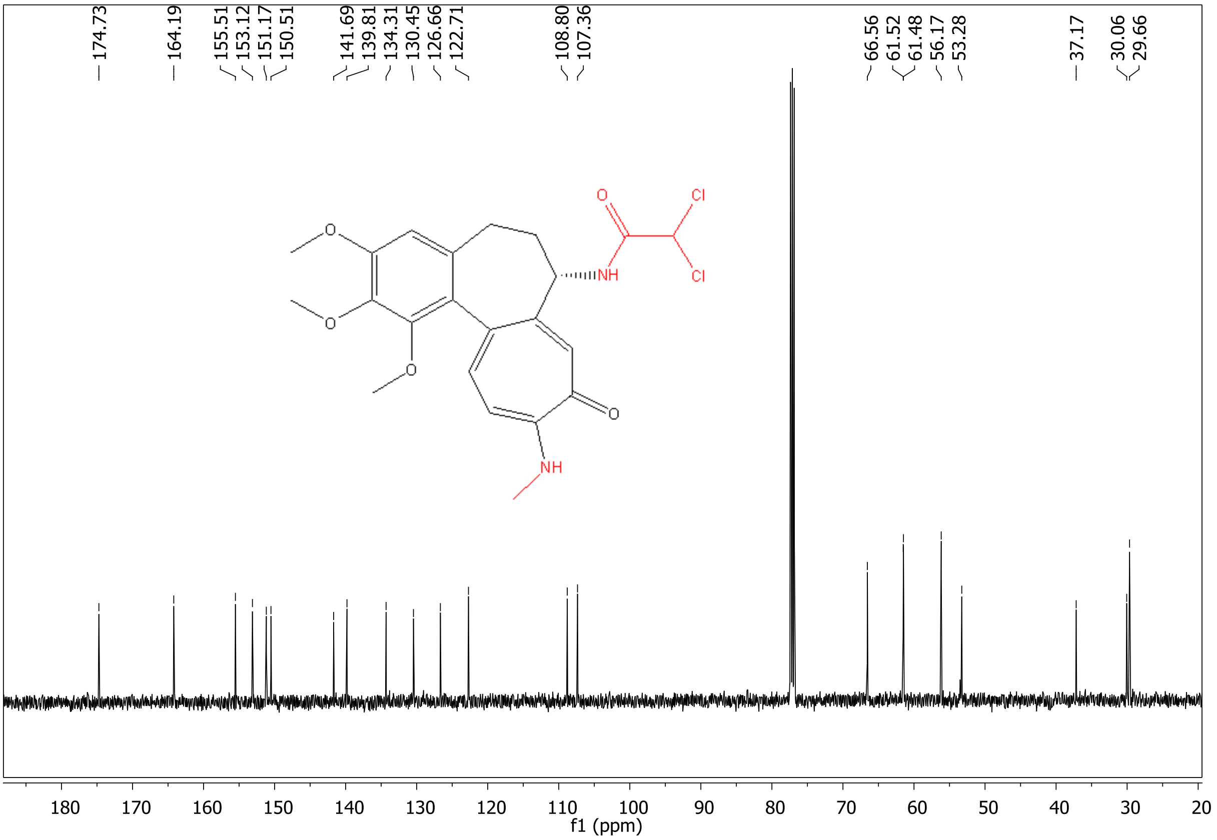

Figure S27. The ^{13}C NMR spectrum of **10** in $CDCl_3$.

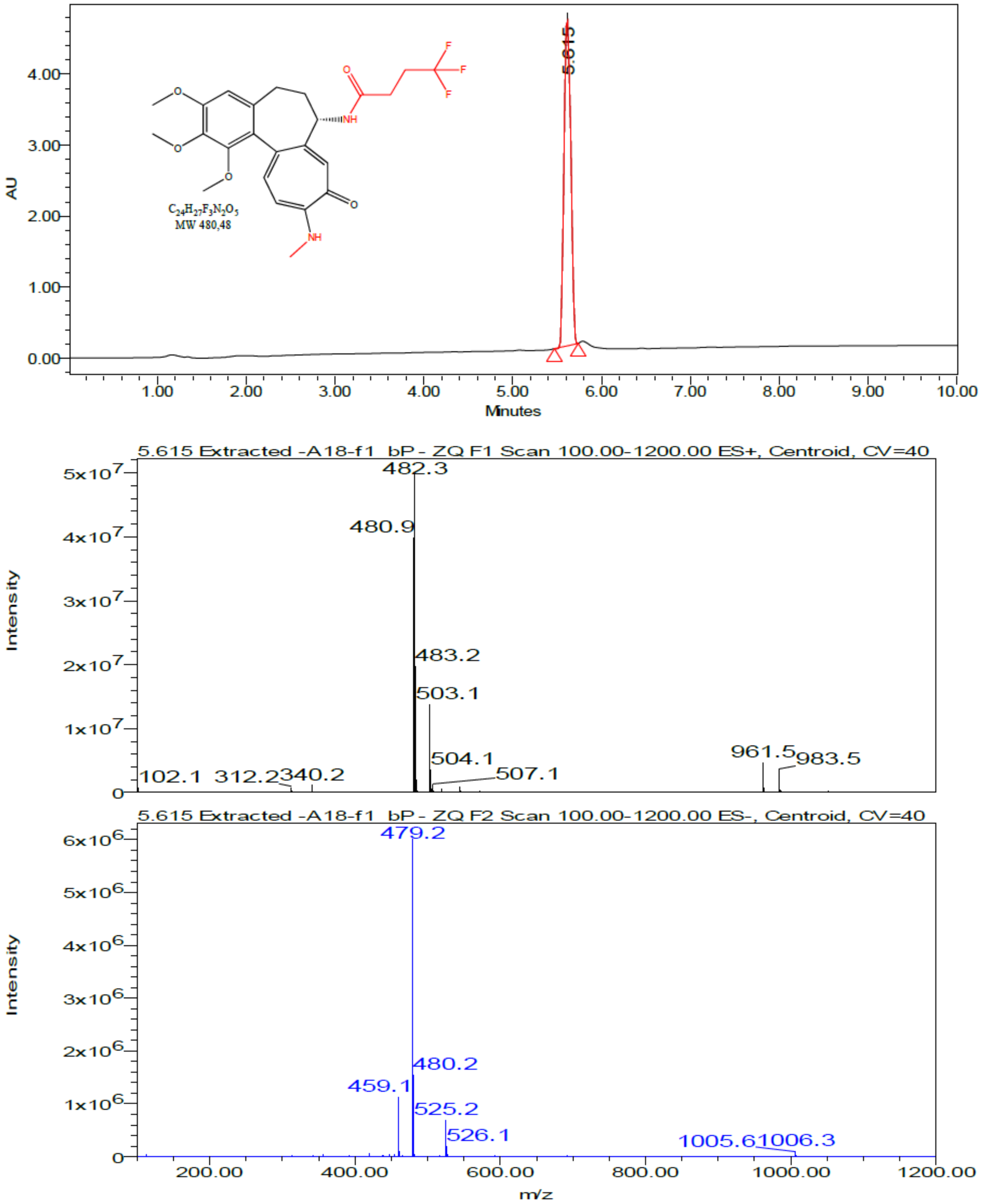

Figure S28. The LC-MS chromatogram and mass spectra of **11**.

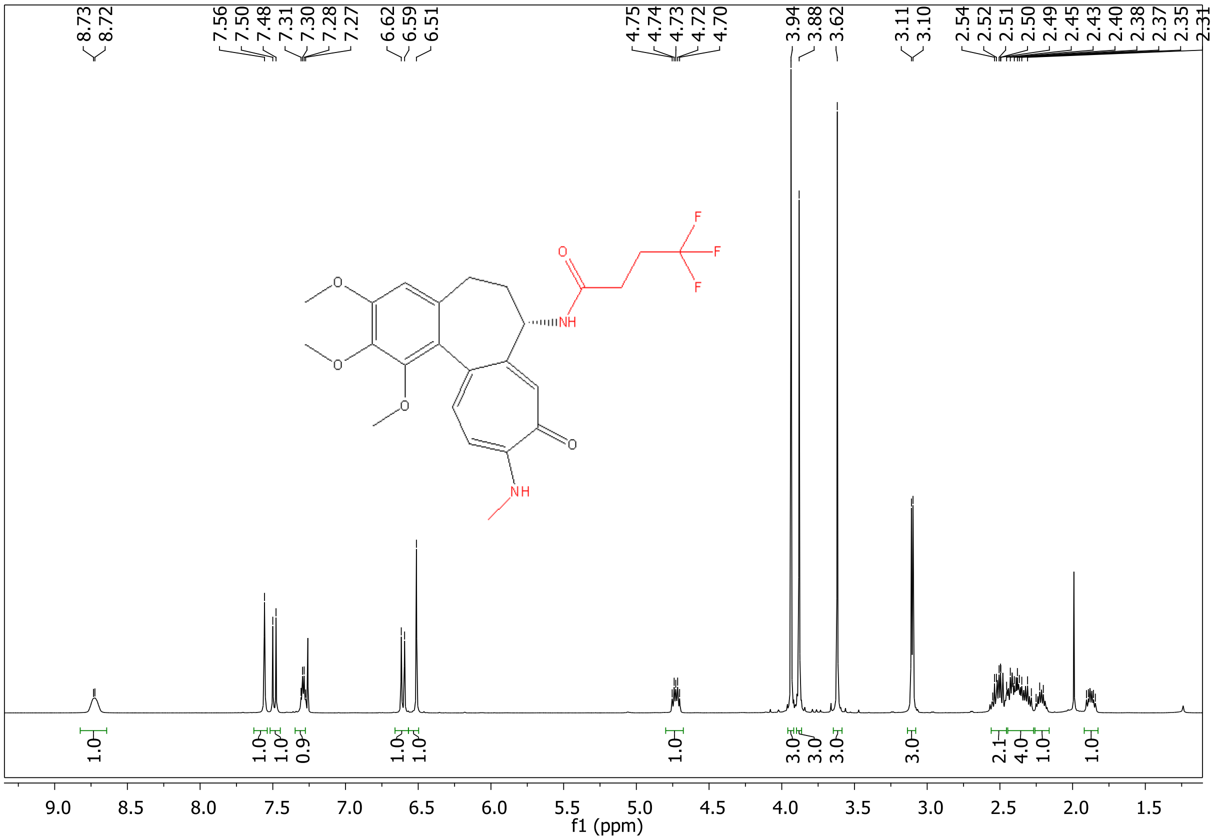

Figure S29. The ^{1}H NMR spectrum of **11** in $CDCl_3$.

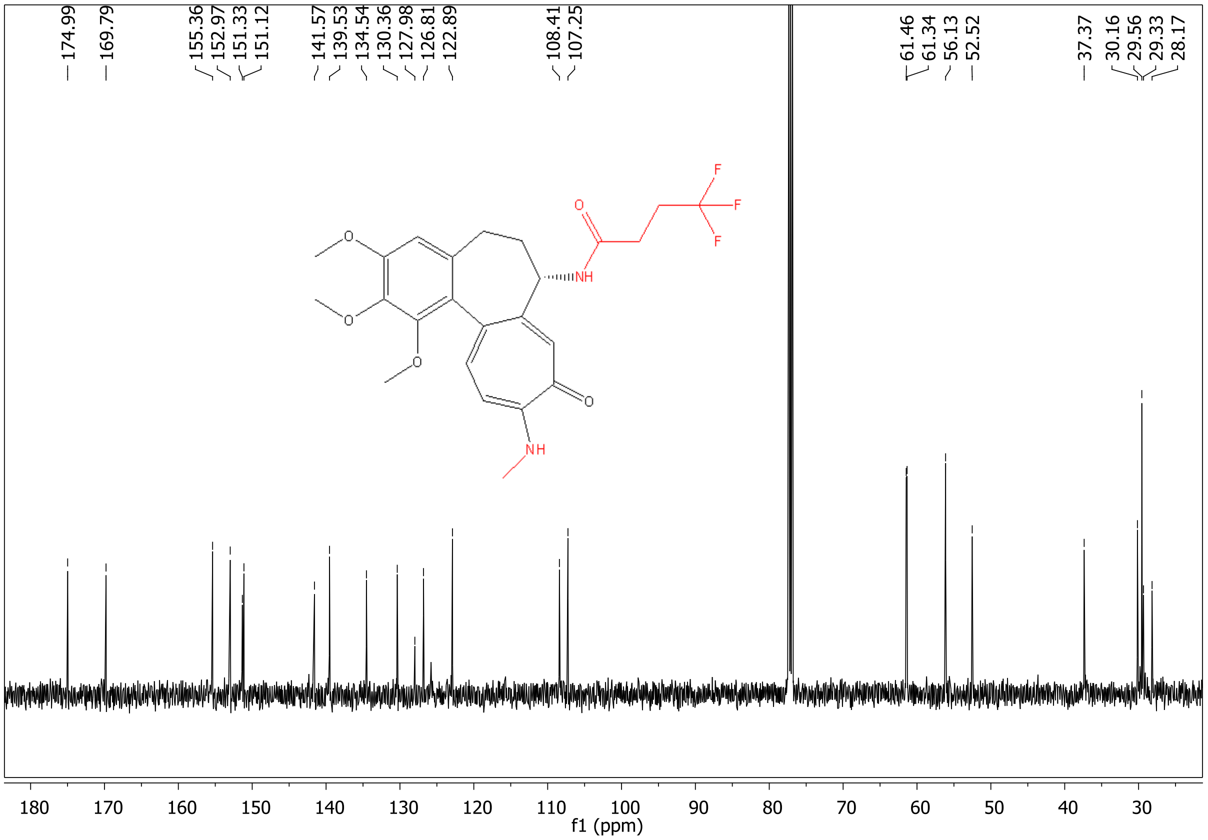

Figure S30. The ^{13}C NMR spectrum of **11** in $CDCl_3$.

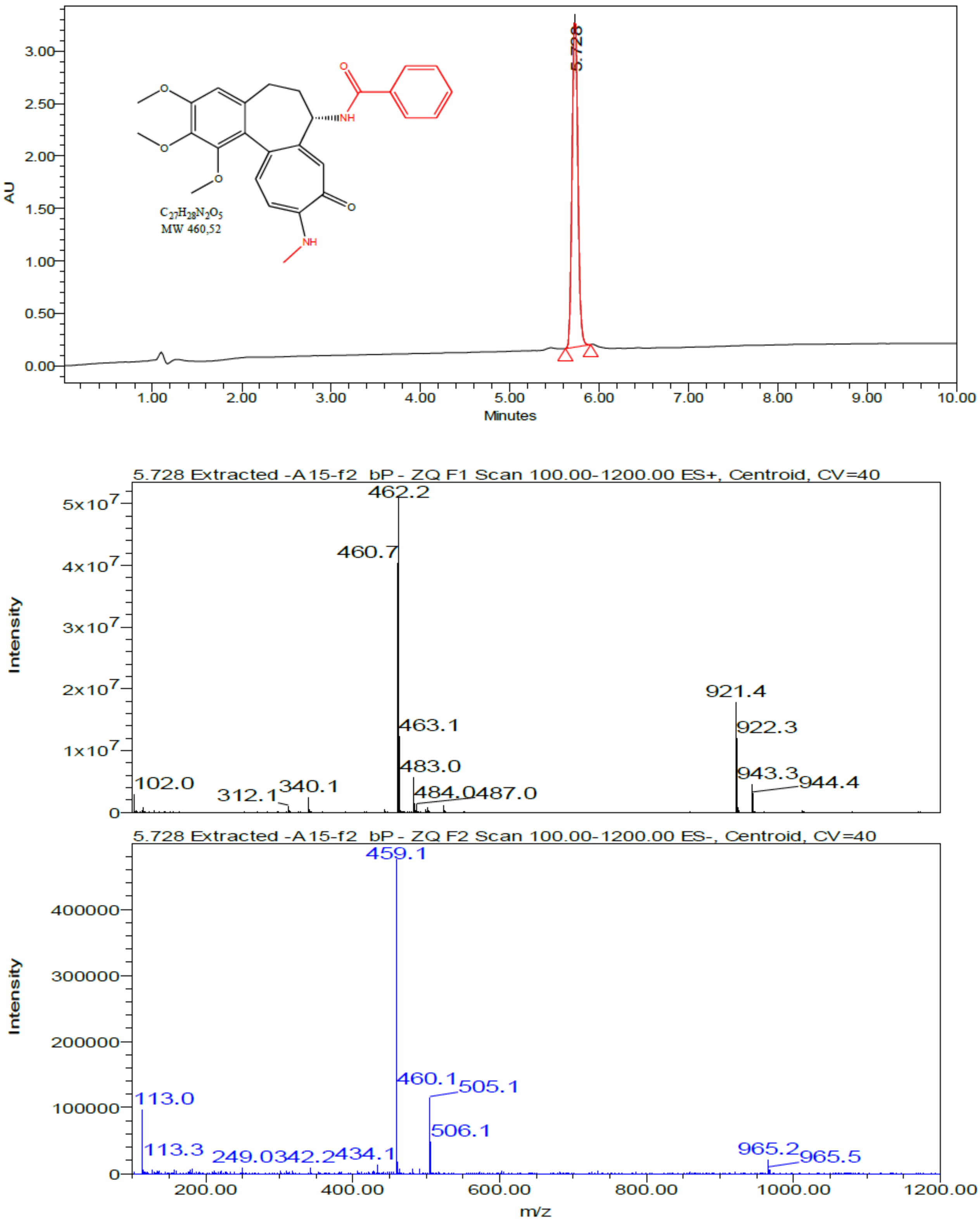

Figure S31. The LC-MS chromatogram and mass spectra of **12**.

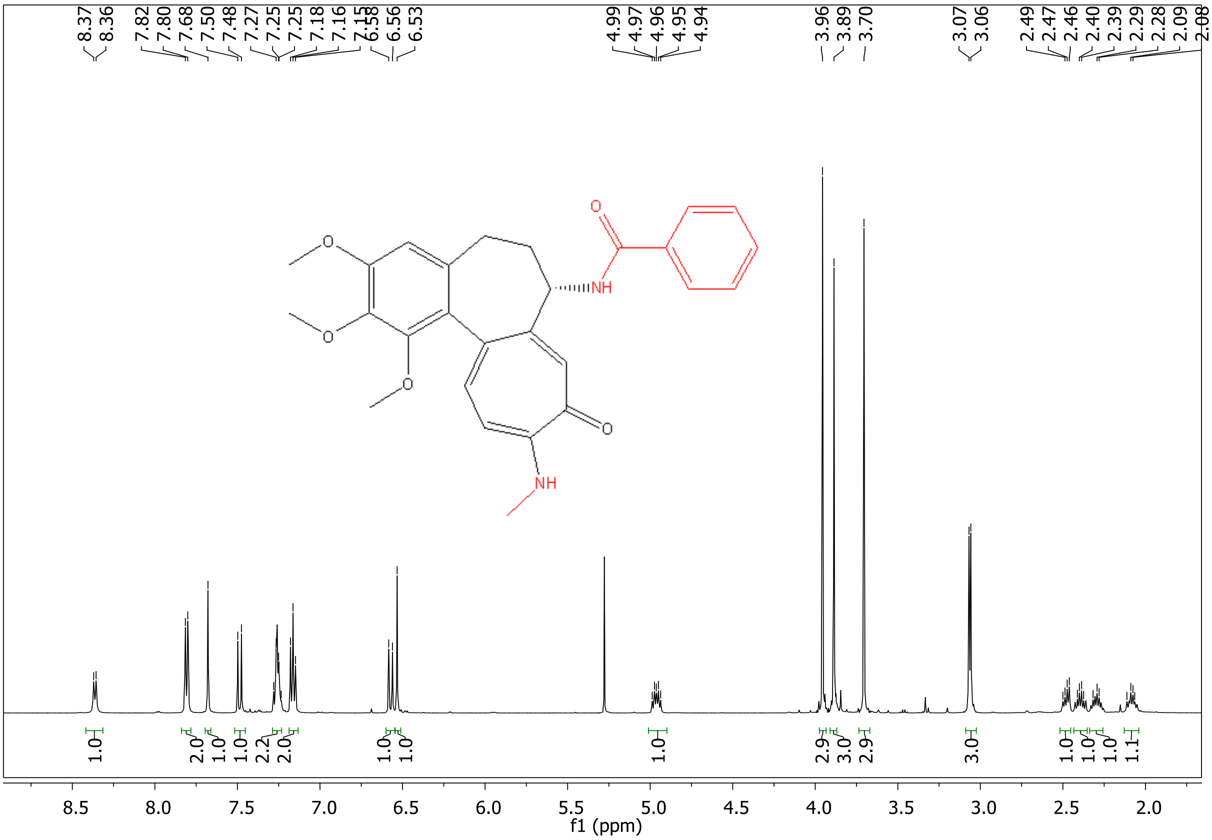

Figure S32. The ^{1}H NMR spectrum of **12** in $CDCl_3$.

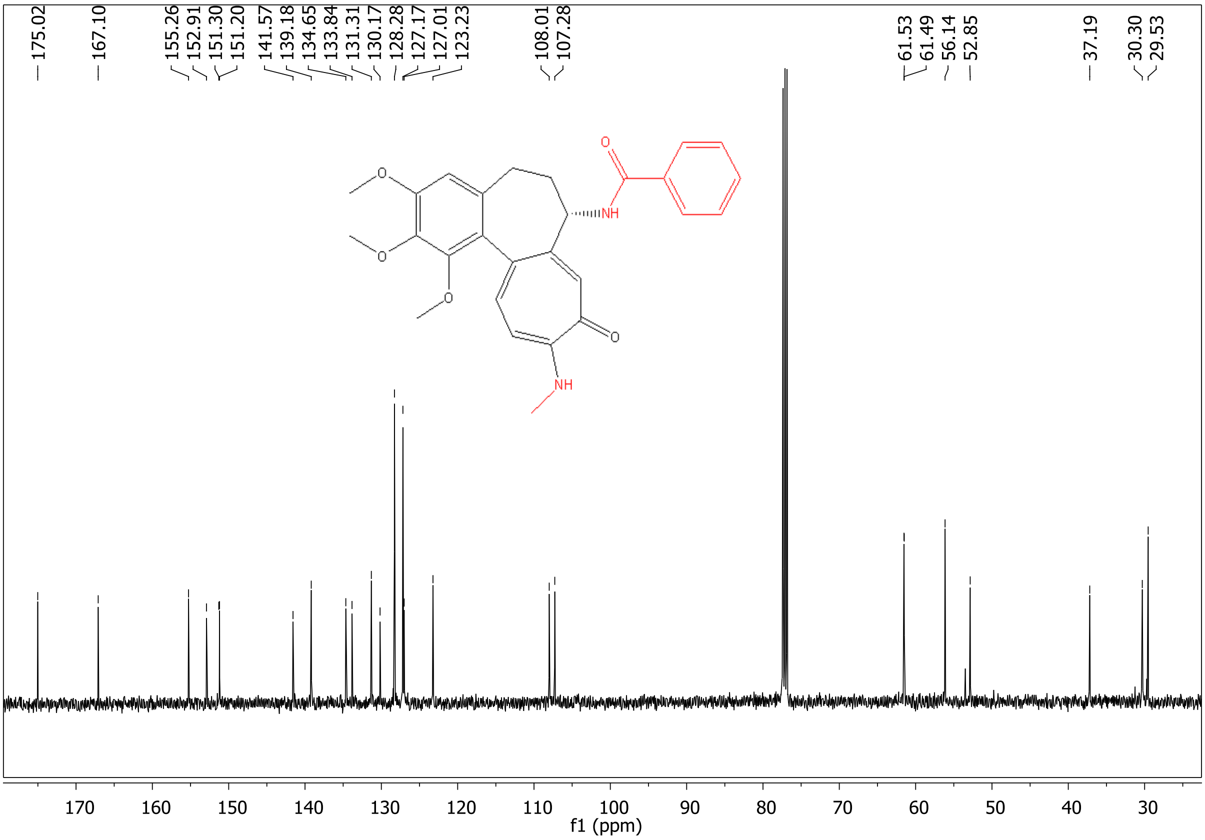

Figure S33. The ^{13}C NMR spectrum of **12** in $CDCl_3$.

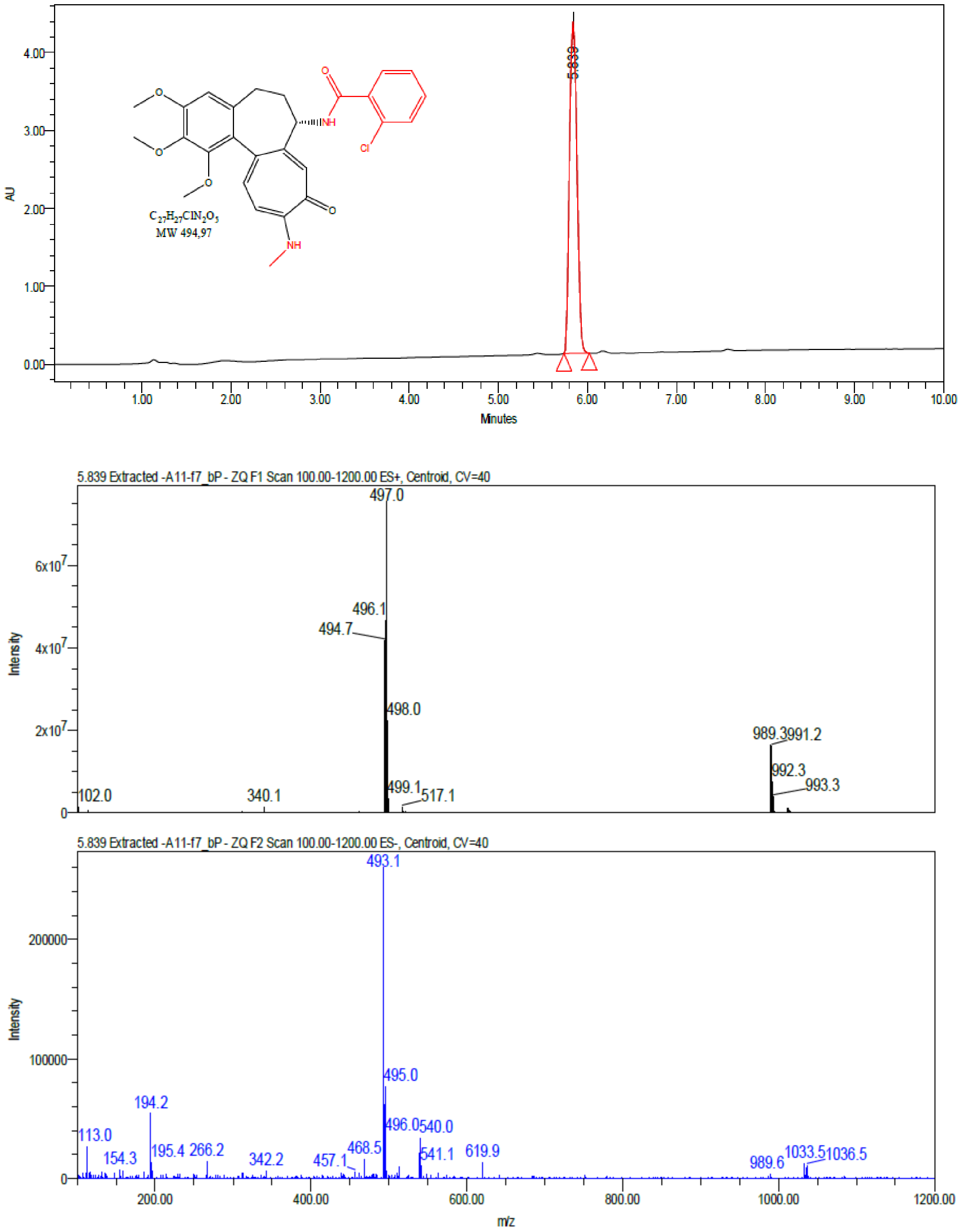

Figure S34. The LC-MS chromatogram and mass spectra of **13**.

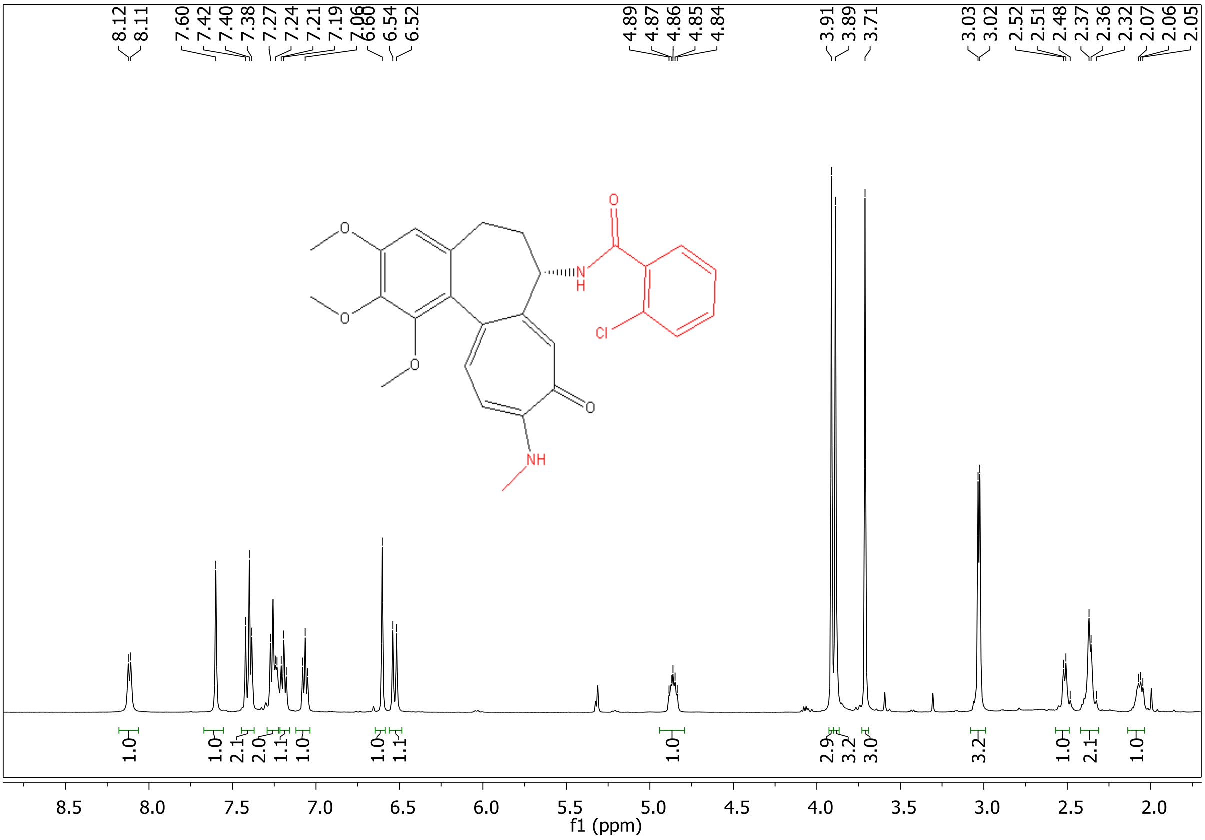

Figure S35. The ^{1}H NMR spectrum of **13** in CD_2Cl_2.

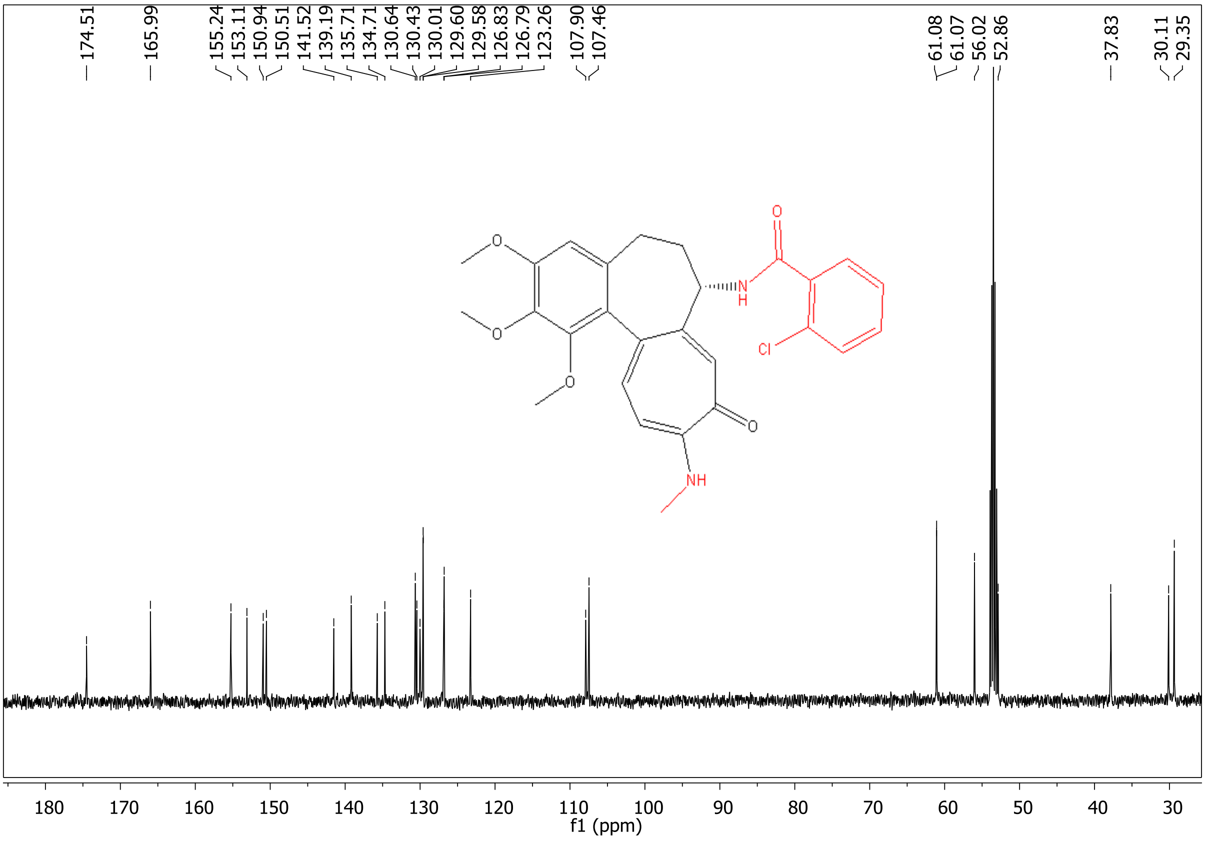

Figure S36. The ^{13}C NMR spectrum of **13** in CD_2Cl_2.

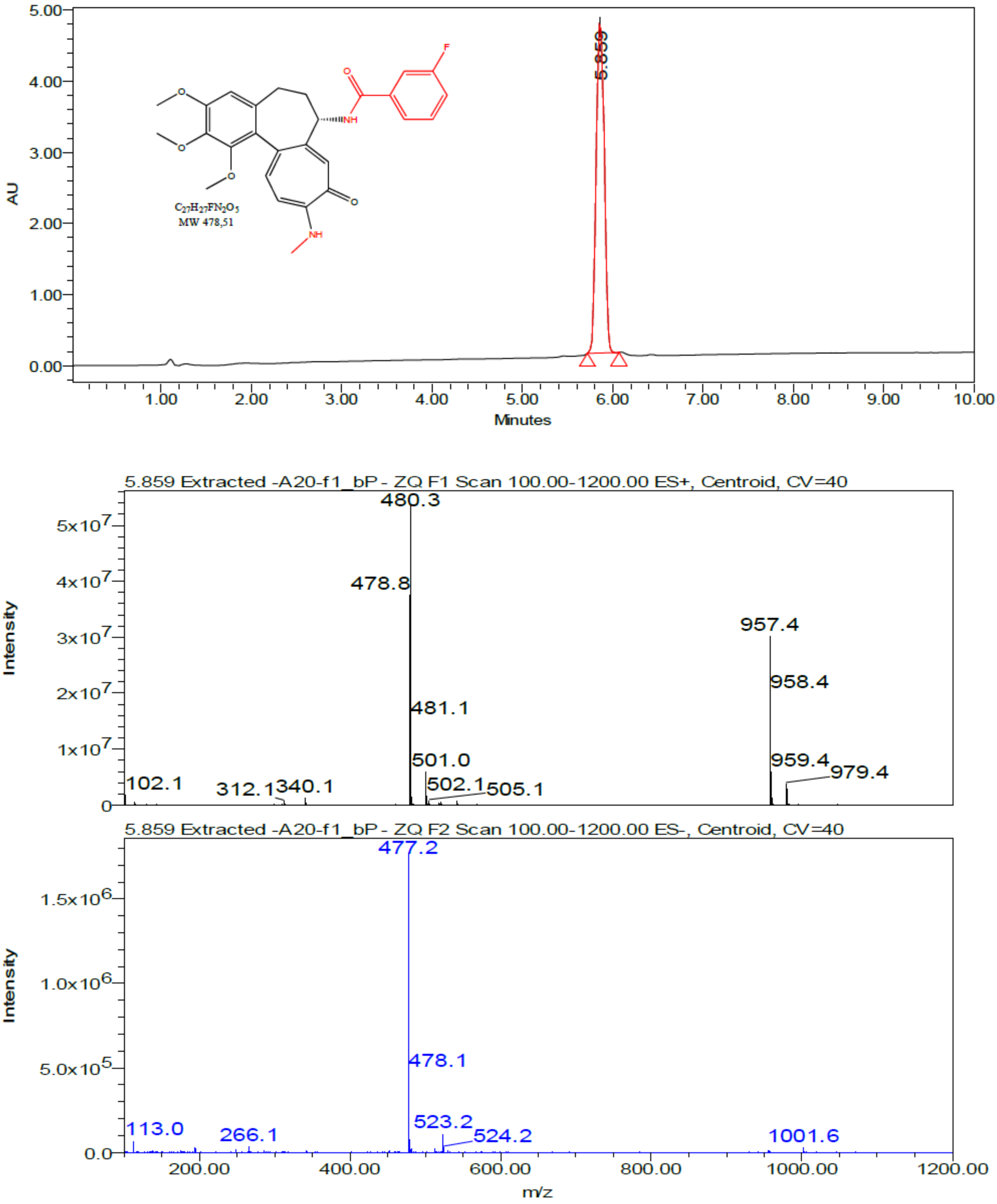

Figure S37. The LC-MS chromatogram and mass spectra of **14**.

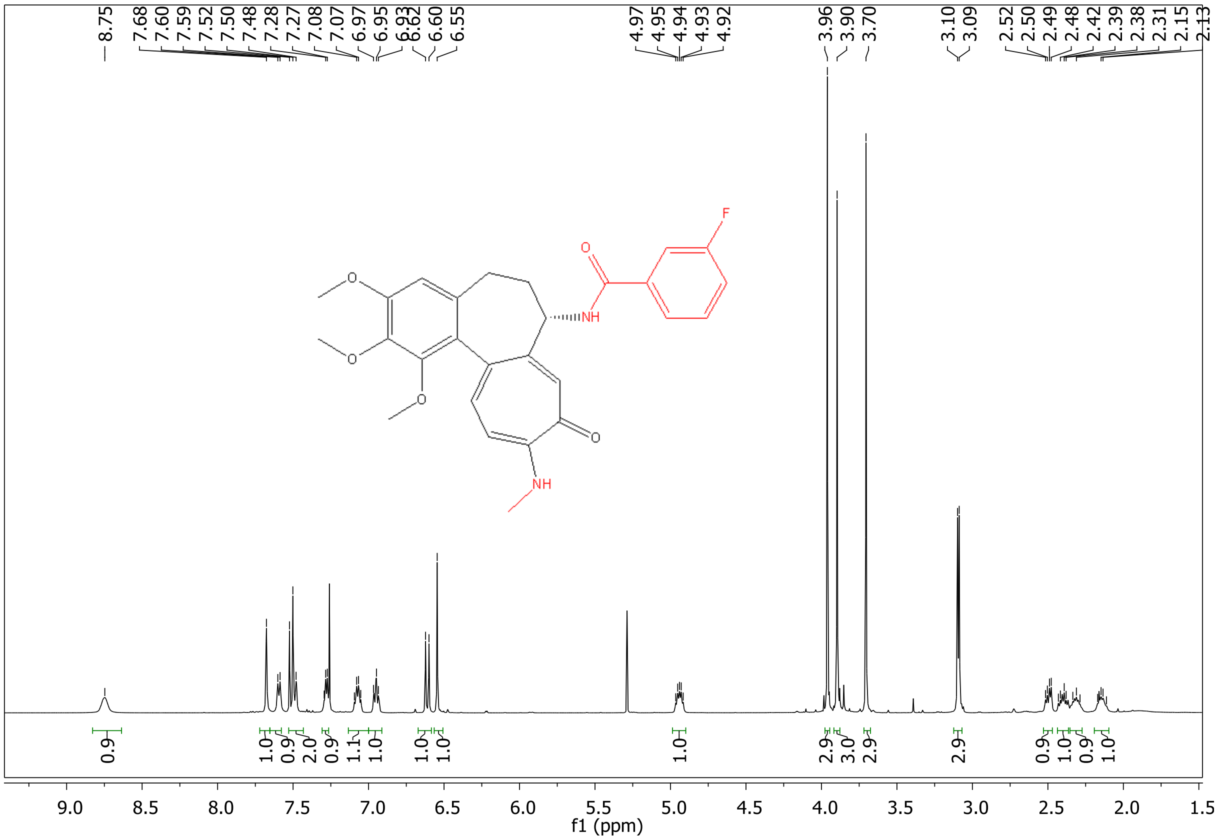

Figure S38. The ^{1}H NMR spectrum of **14** in $CDCl_3$.

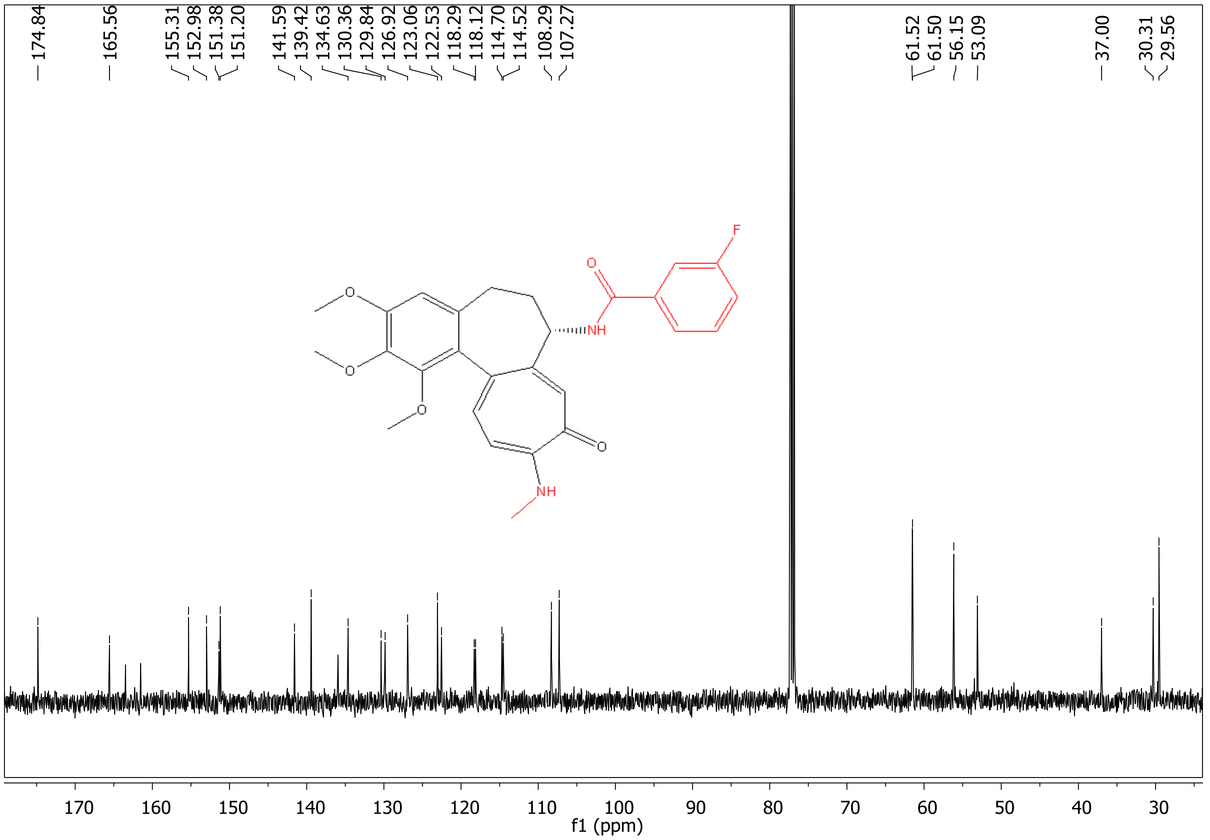

Figure S39. The ^{13}C NMR spectrum of **14** in $CDCl_3$.

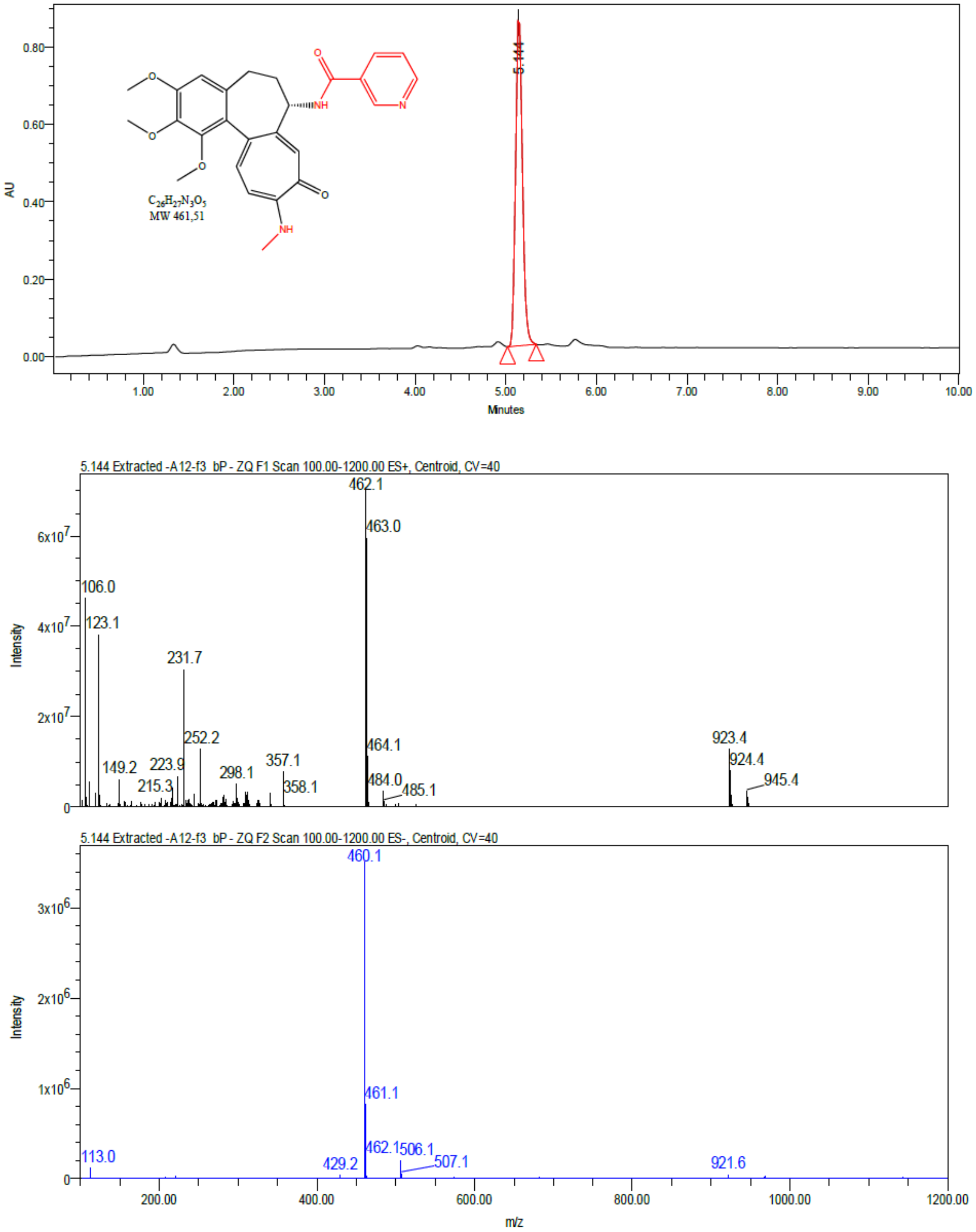

Figure S40. The LC-MS chromatogram and mass spectra of **15**.

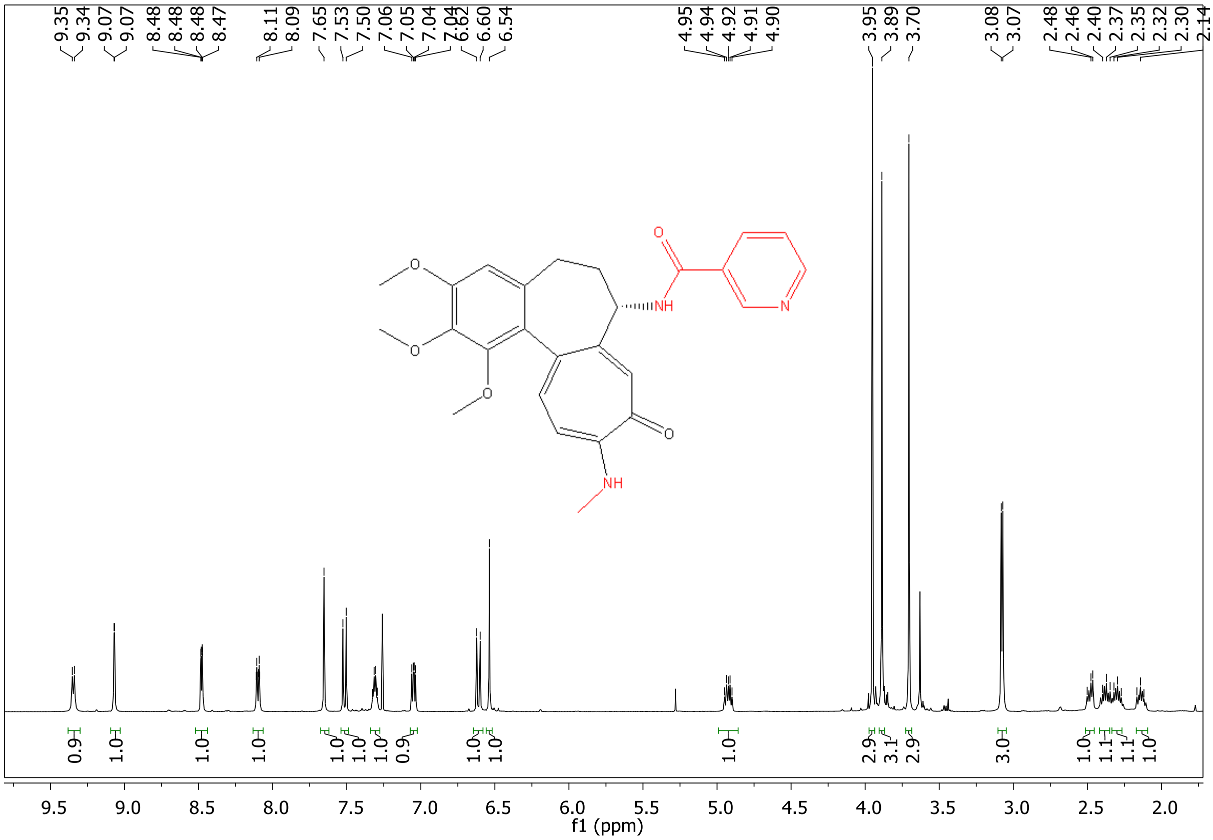

Figure S41. The ^{1}H NMR spectrum of **15** in $CDCl_3$.

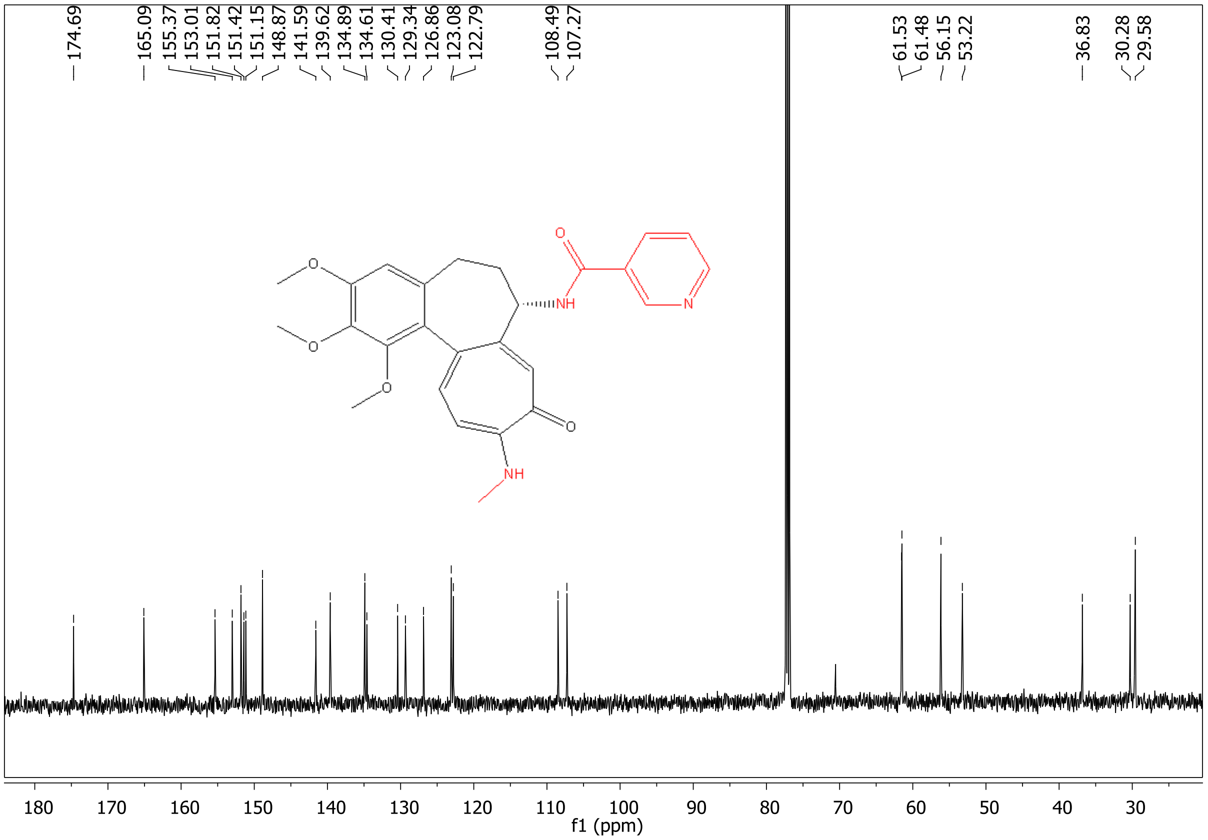

Figure S42. The ^{13}C NMR spectrum of **15** in $CDCl_3$.

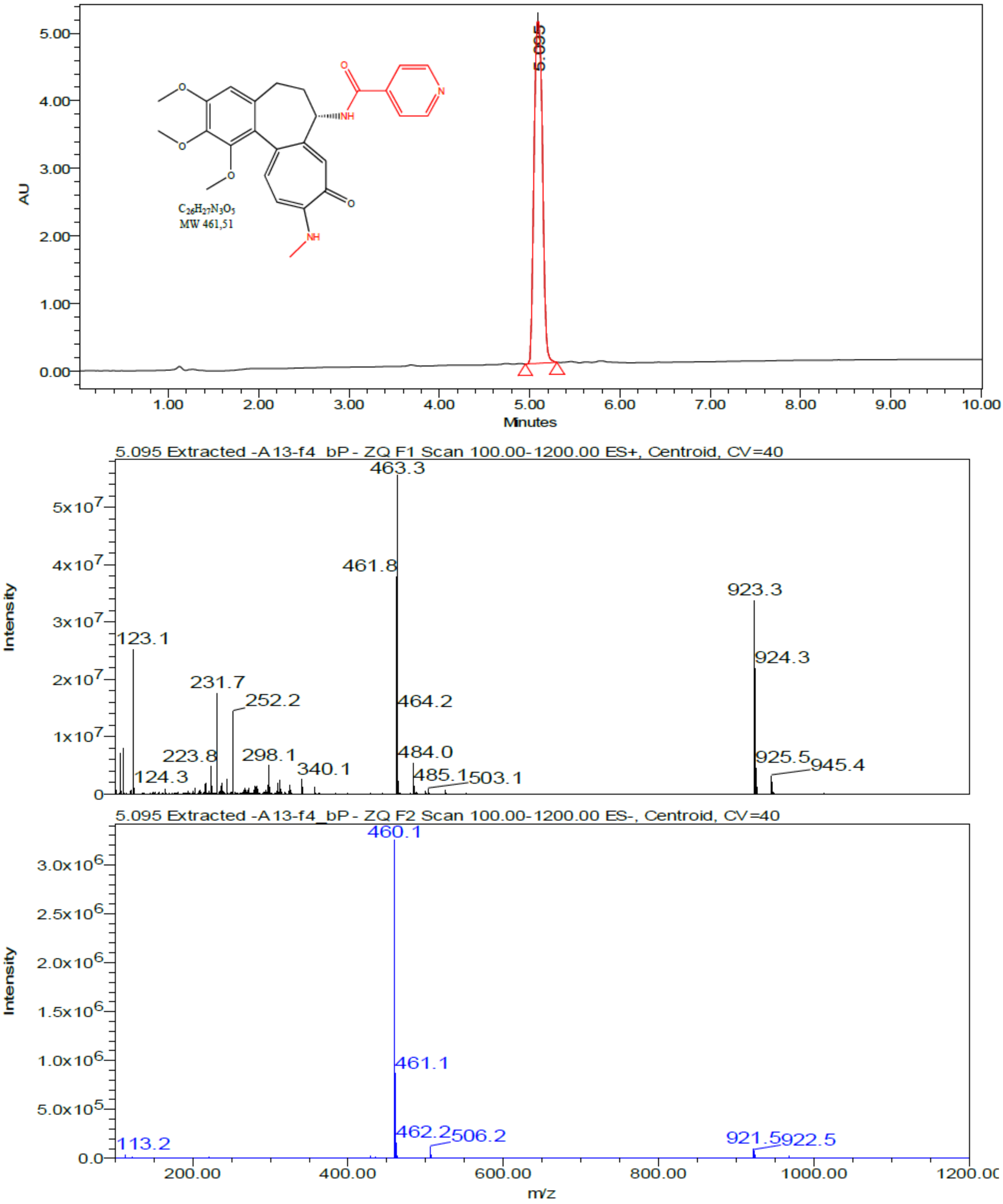

Figure S43. The LC-MS chromatogram and mass spectra of **16**.

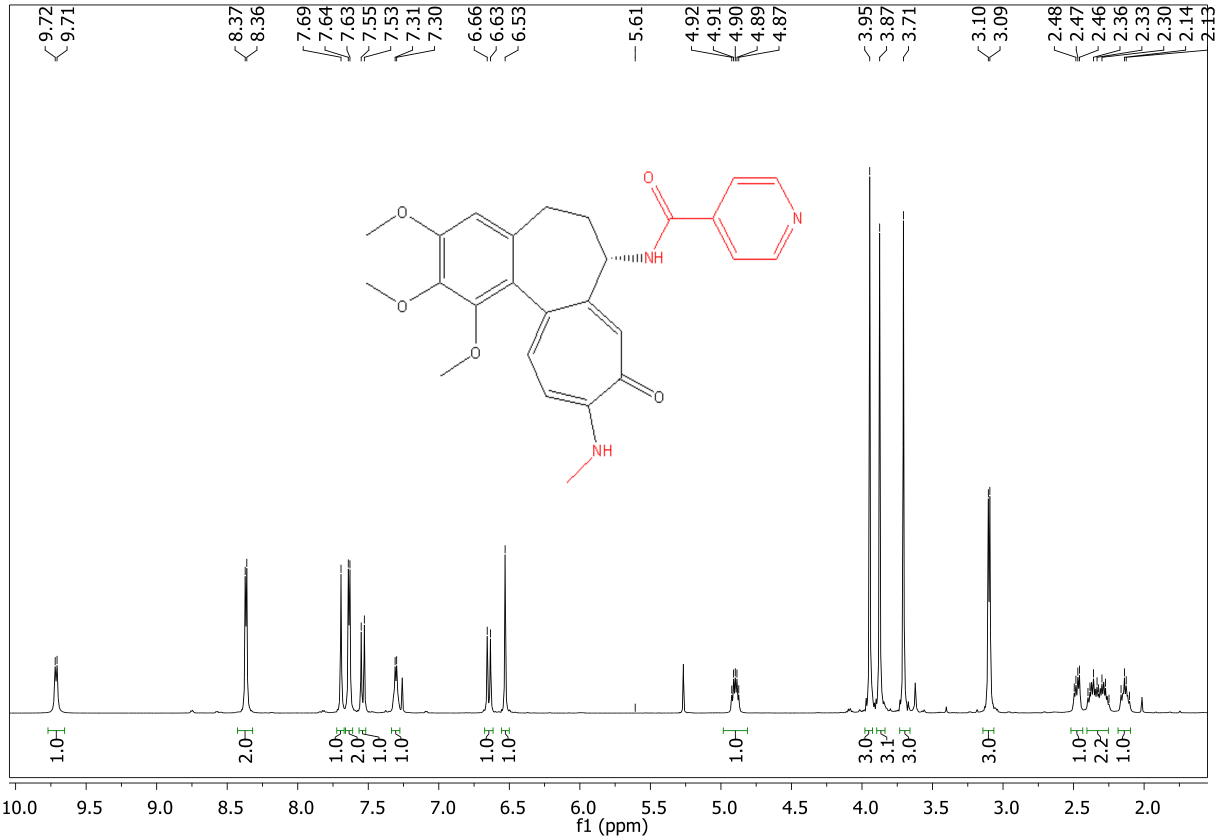

Figure S44. The ^{1}H NMR spectrum of **16** in $CDCl_3$.

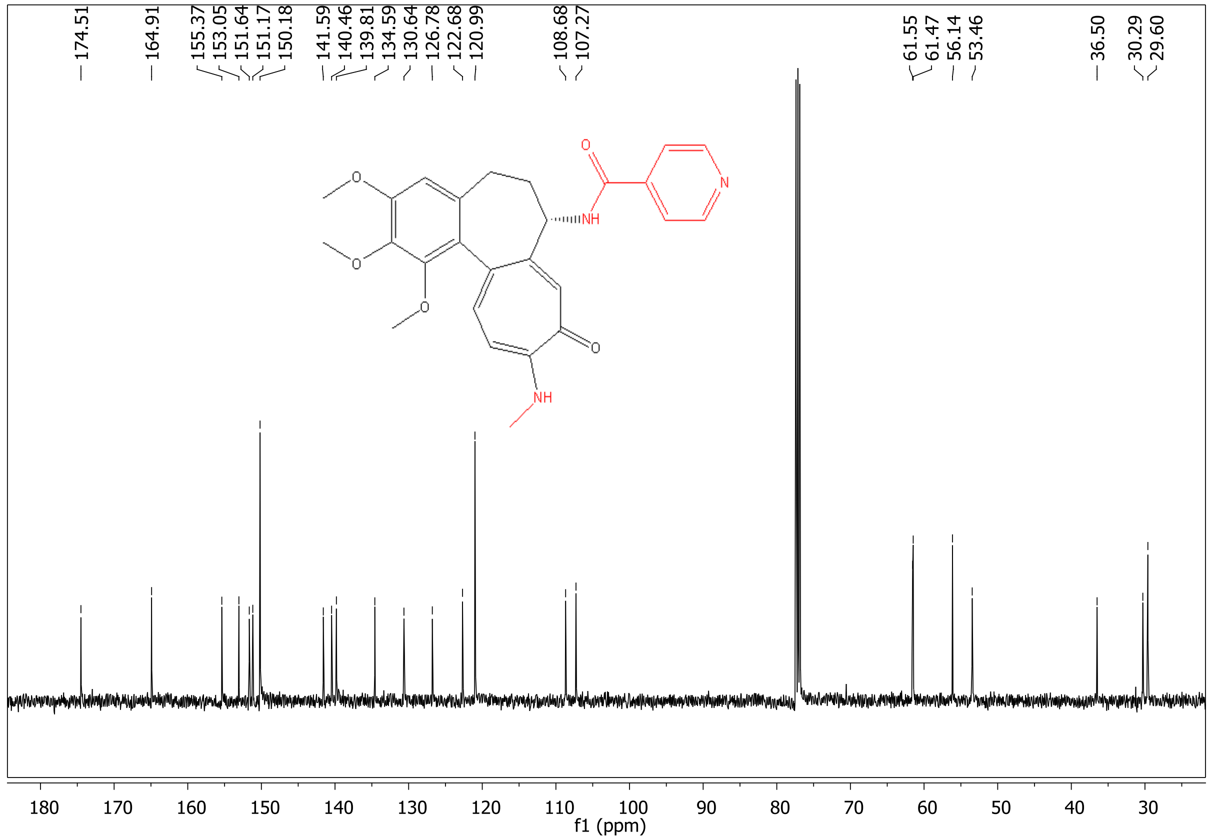

Figure S45. The ^{13}C NMR spectrum of **16** in $CDCl_3$.

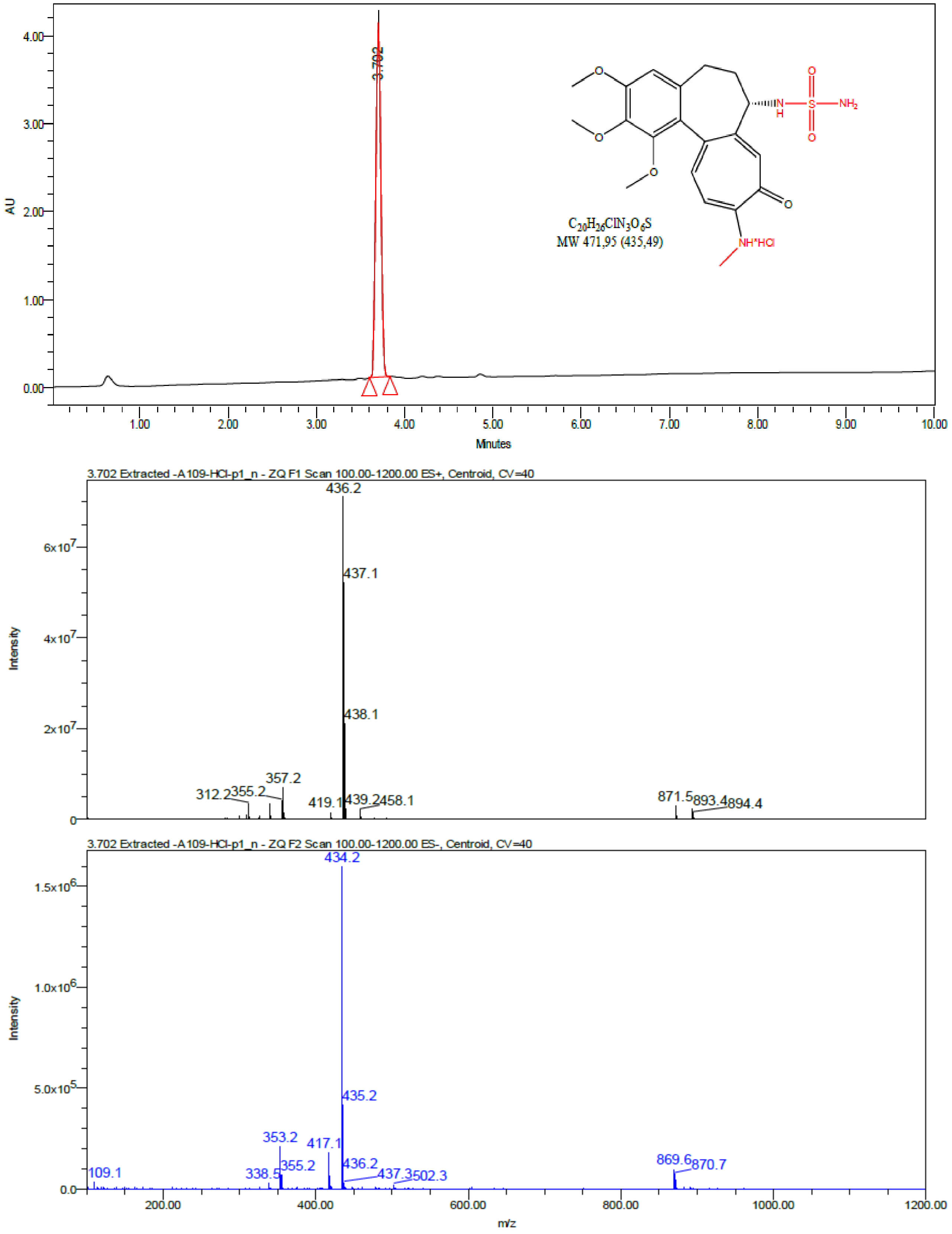

Figure S46. The LC-MS chromatogram and mass spectra of **17**.

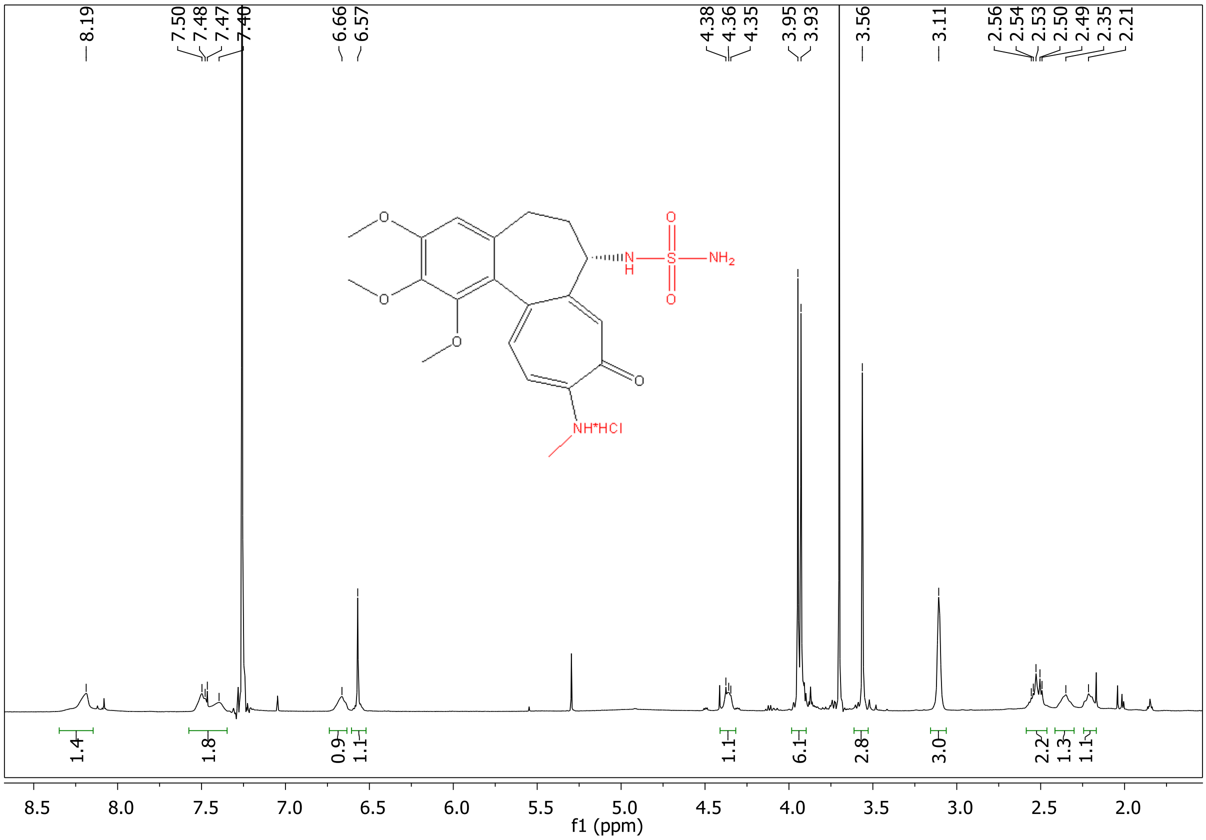

Figure S47. The ^{1}H NMR spectrum of **17** in $CDCl_3$.

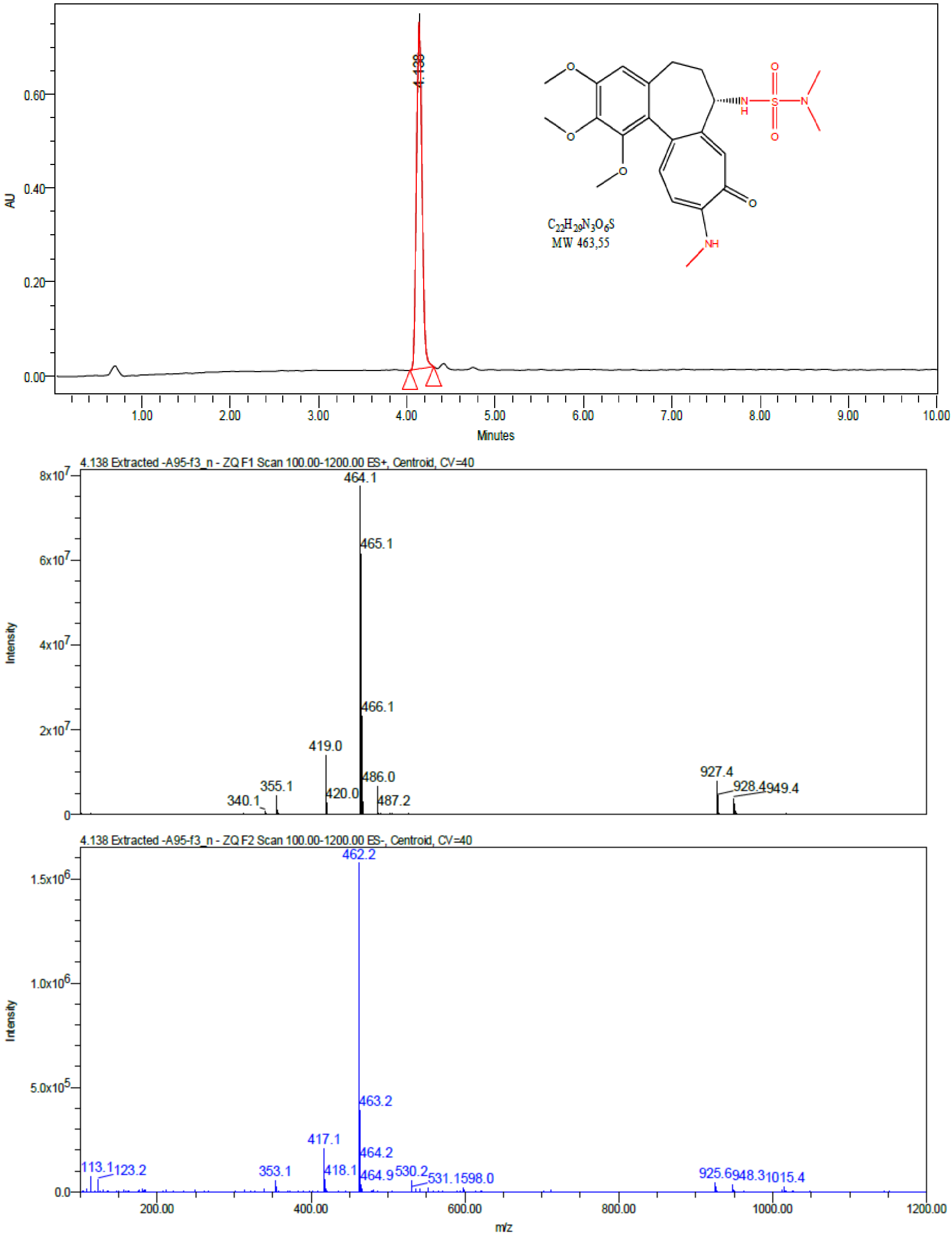

Figure S48. The LC-MS chromatogram and mass spectra of **18**.

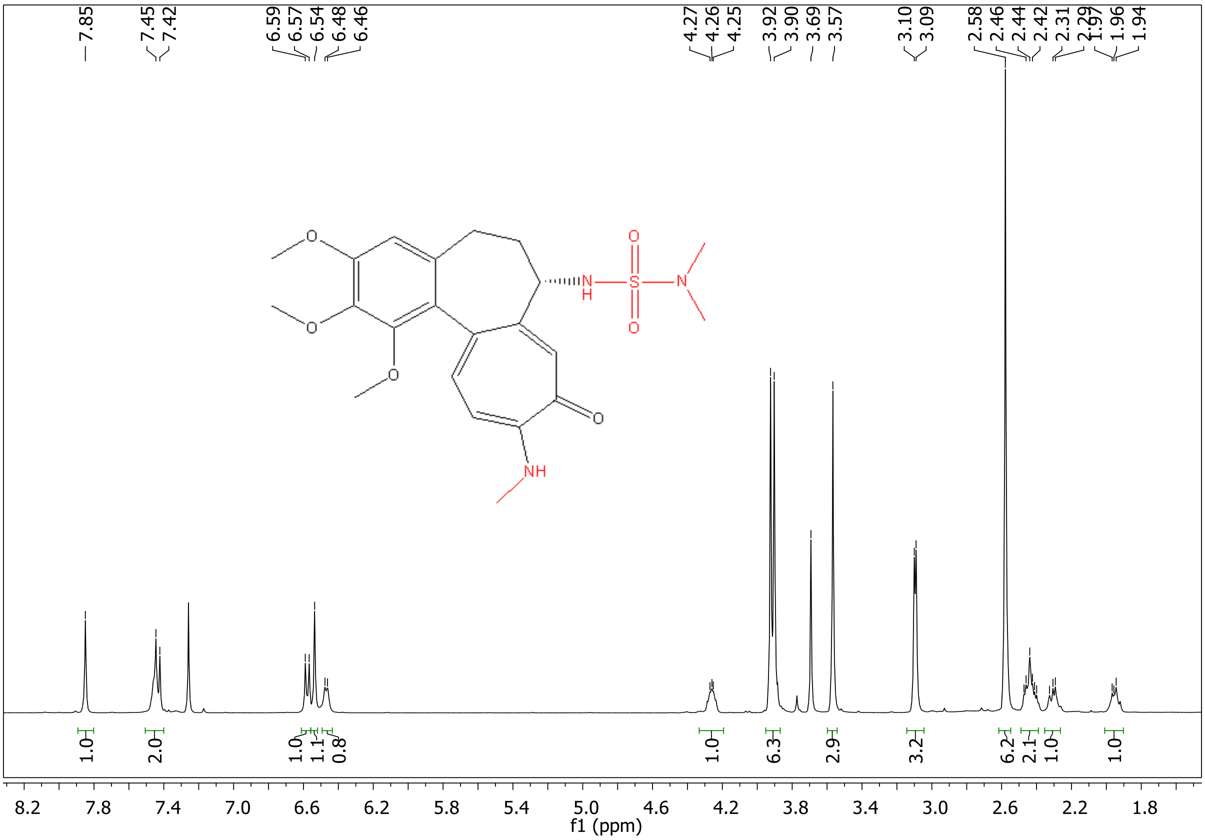

Figure S49. The ^{1}H NMR spectrum of **18** in $CDCl_3$.

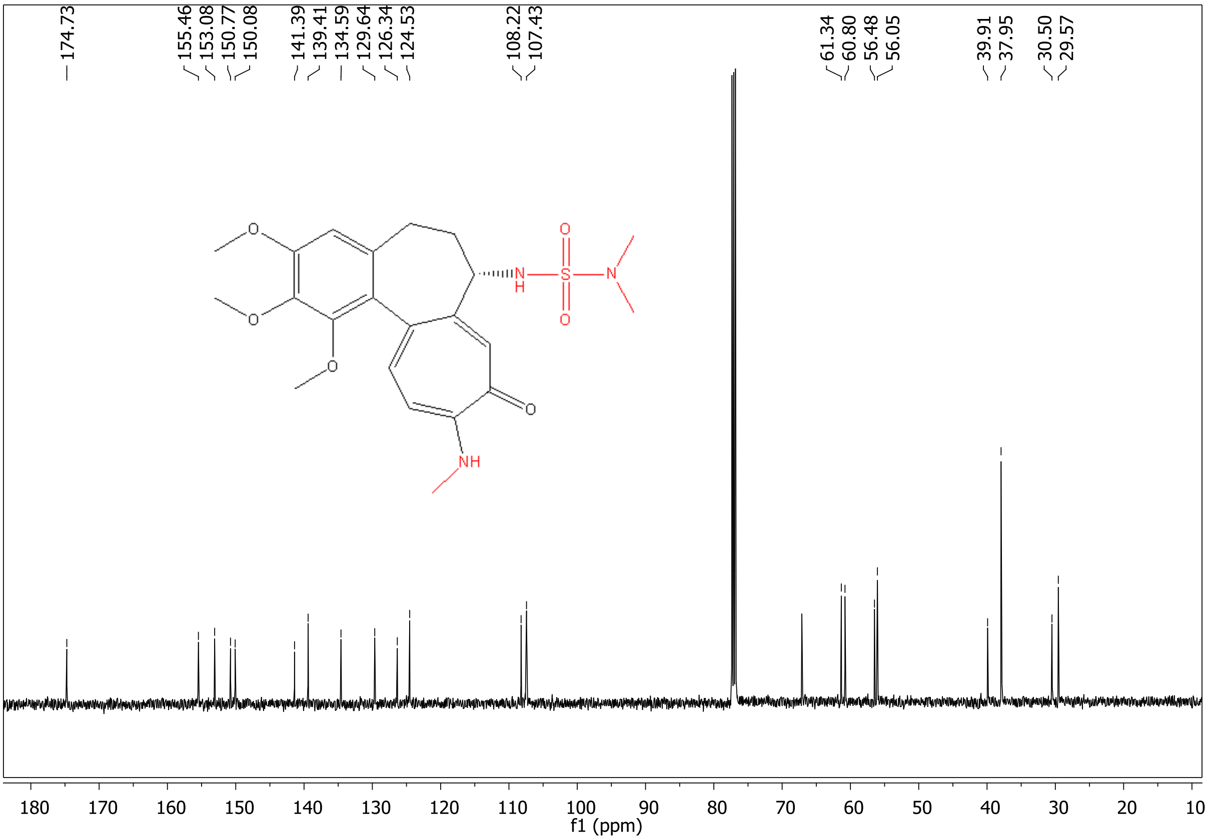

Figure S50. The ^{13}C NMR spectrum of **18** in $CDCl_3$.

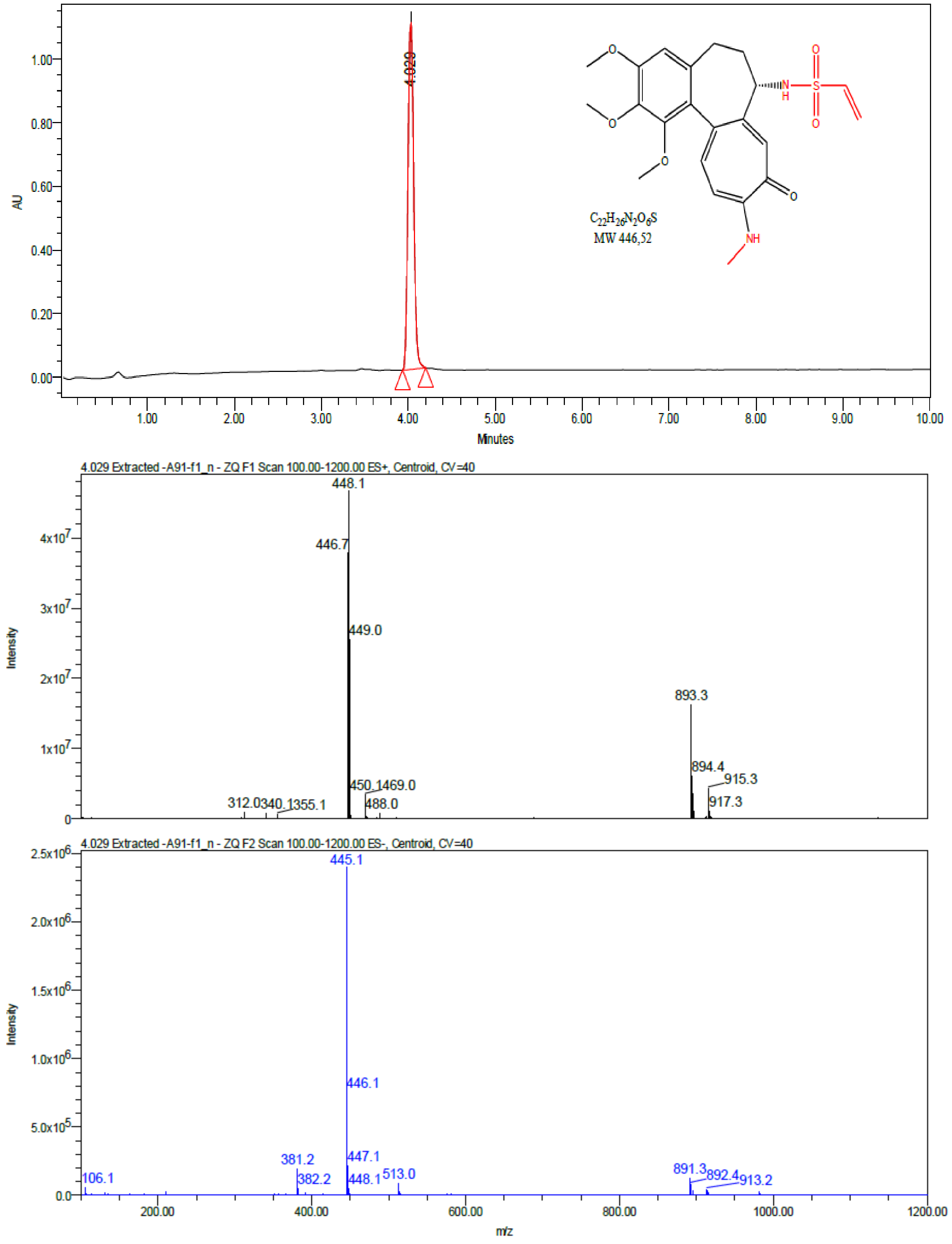

Figure S51. The LC-MS chromatogram and mass spectra of **19**.

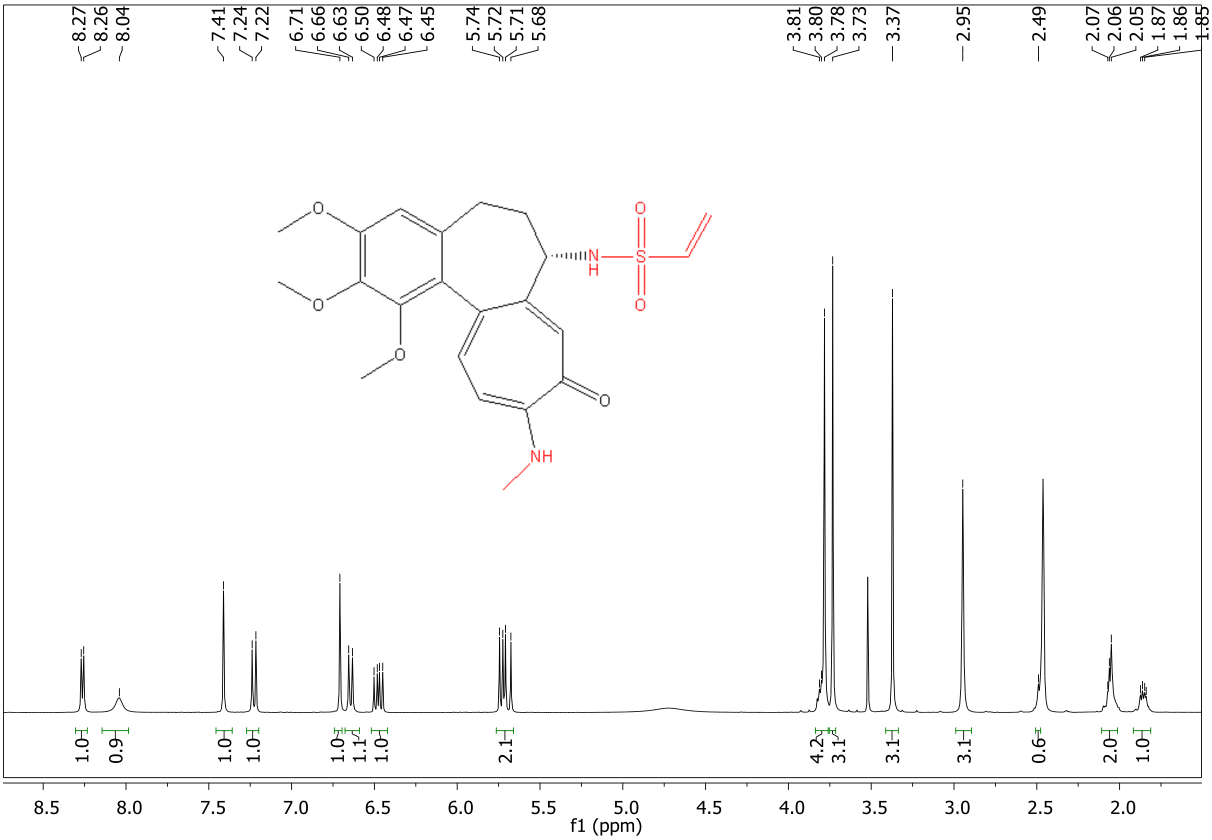

Figure S52. The ^{1}H NMR spectrum of **19** in $(CD_3)_2SO$.

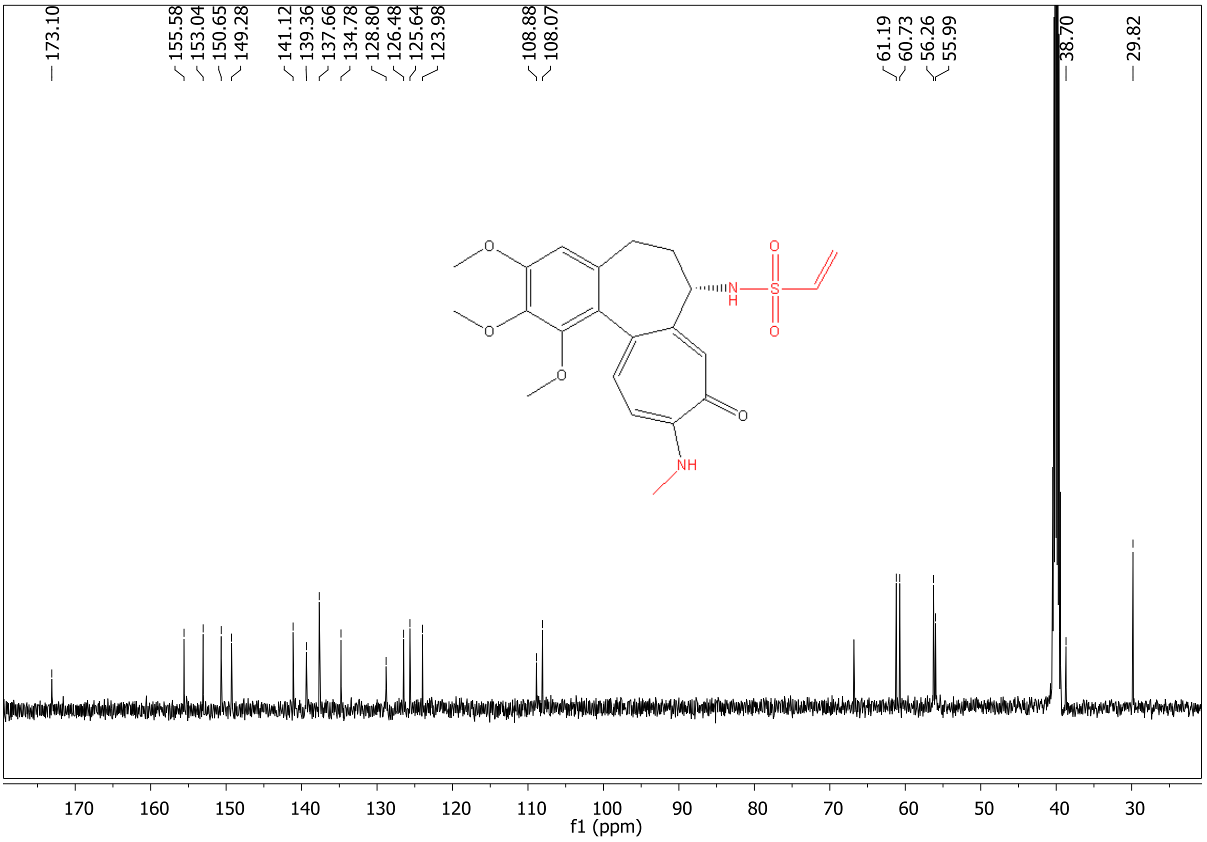

Figure S53. The ^{13}C NMR spectrum of **19** in $(CD_3)_2SO$.

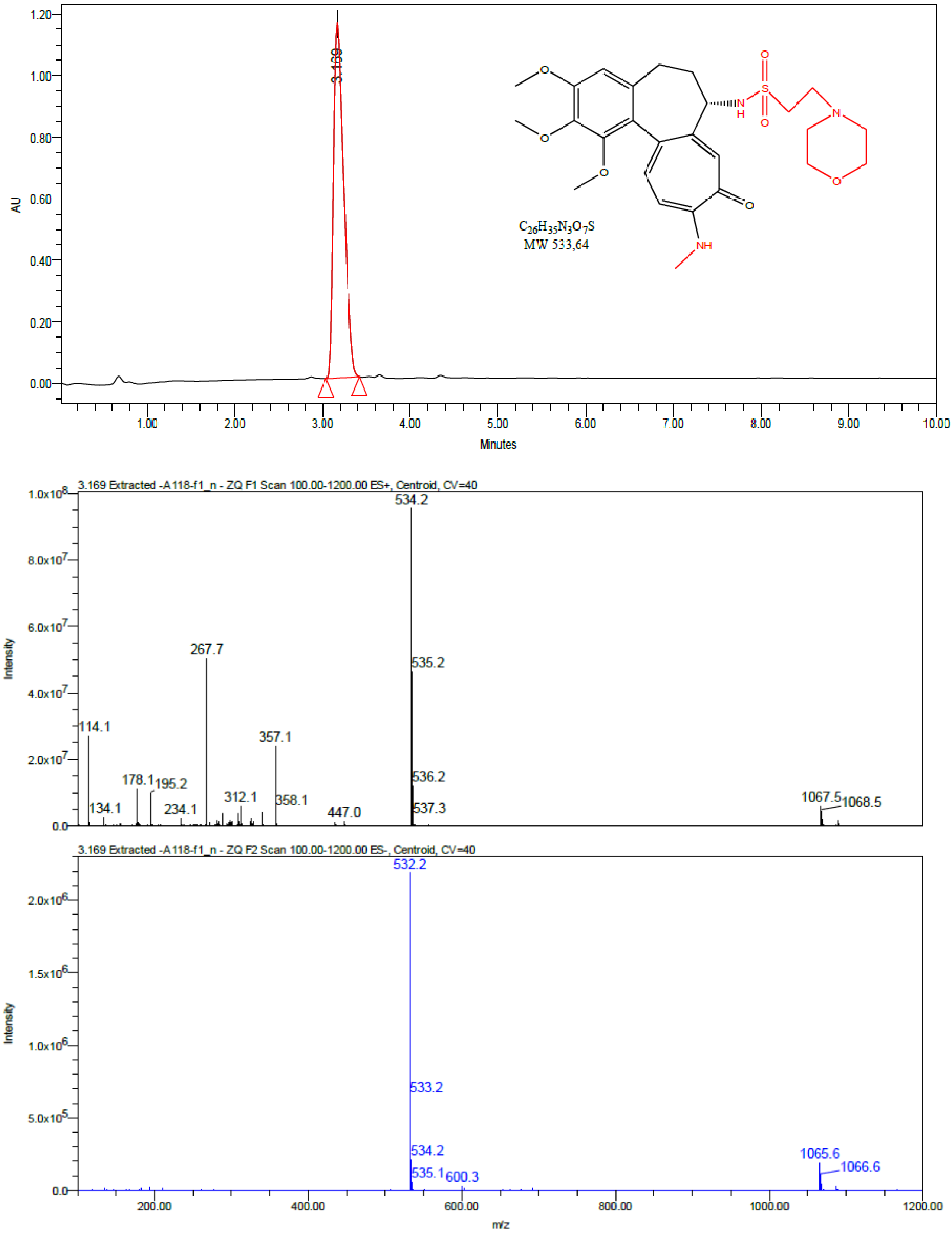

Figure S54. The LC-MS chromatogram and mass spectra of **20**.

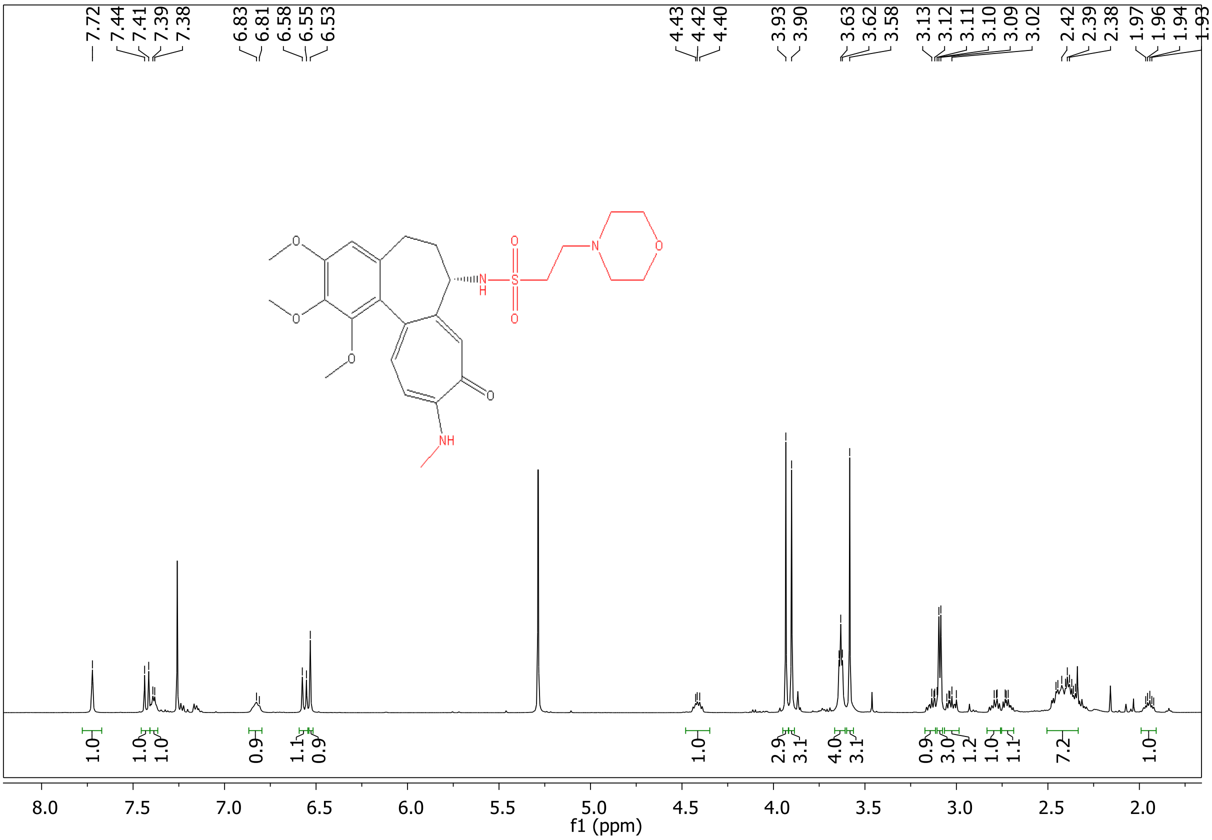

Figure S55. The ^{1}H NMR spectrum of **20** in $CDCl_3$.

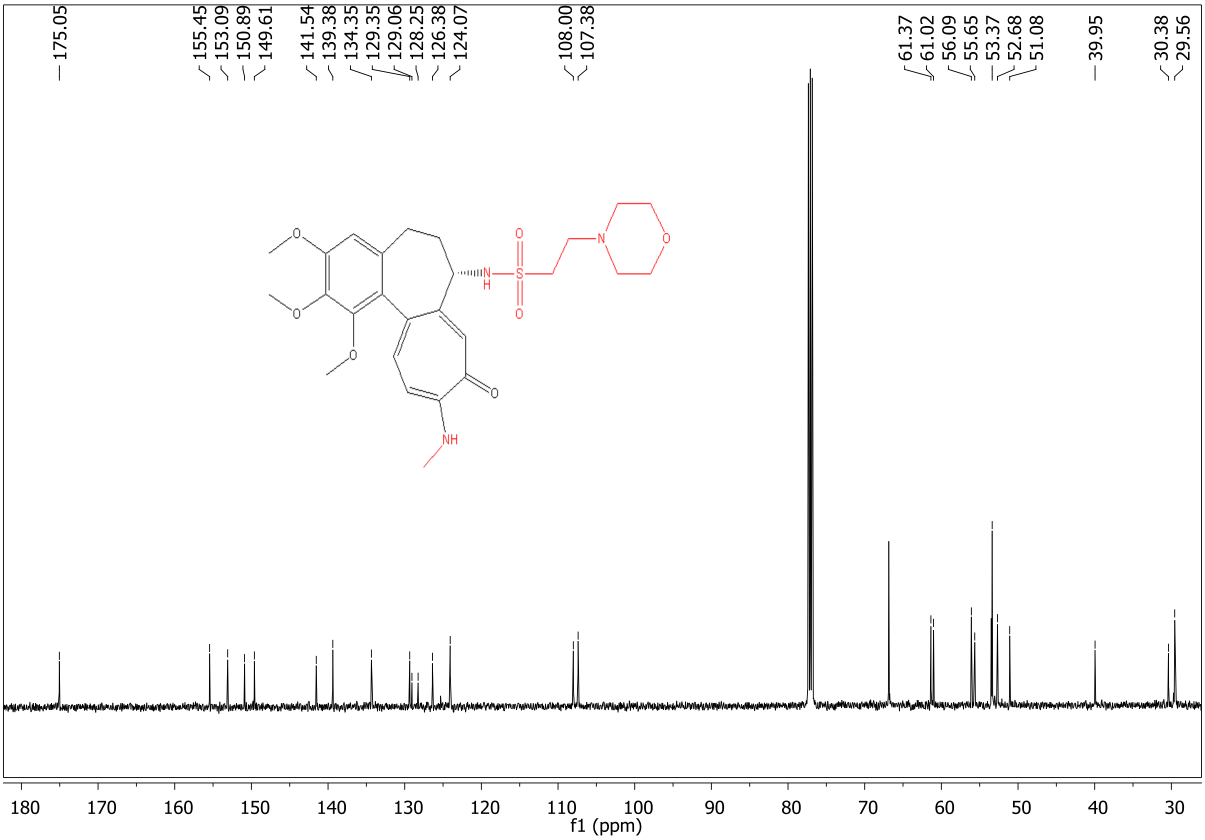

Figure S56. The ^{13}C NMR spectrum of **20** in $CDCl_3$.

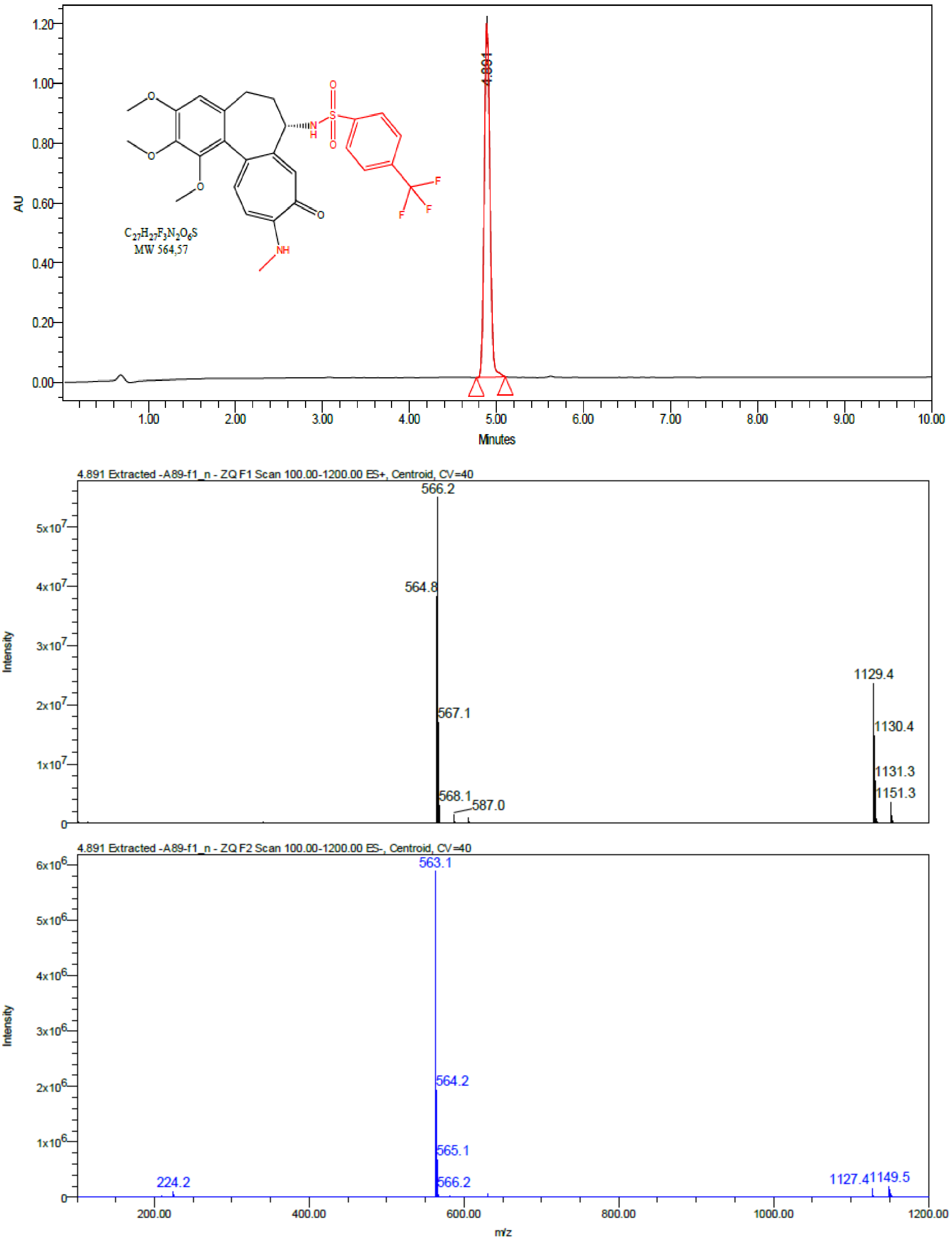

Figure S57. The LC-MS chromatogram and mass spectra of **21**.

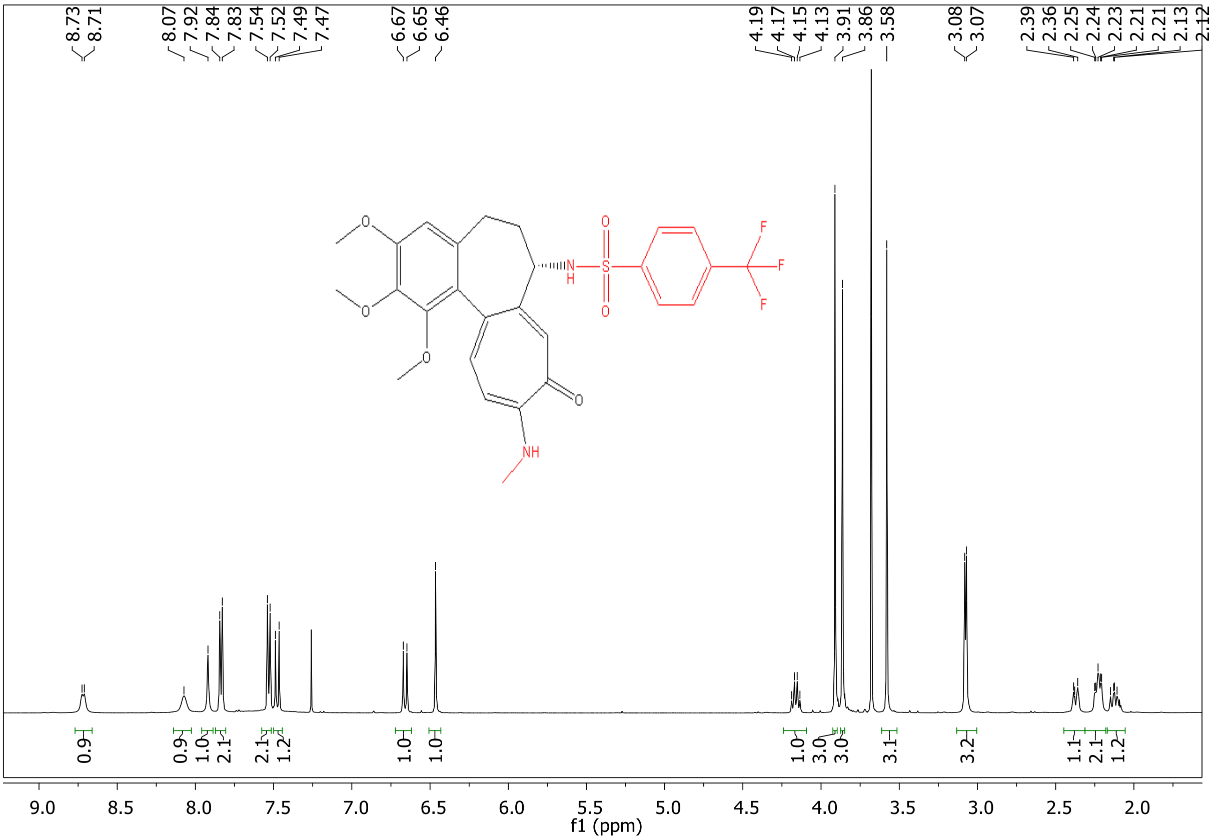

Figure S58. The ^{1}H NMR spectrum of **21** in $CDCl_3$.

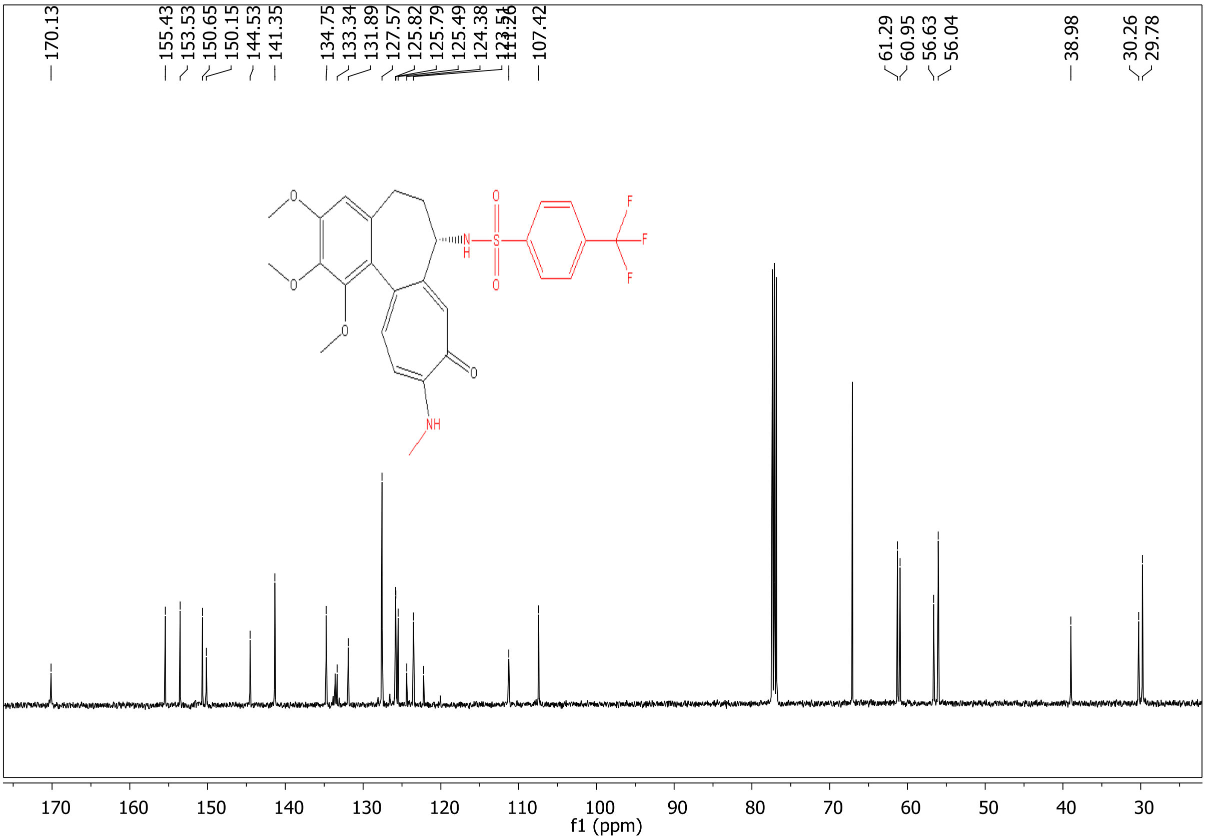

Figure S59. The ^{13}C NMR spectrum of **21** in $CDCl_3$.

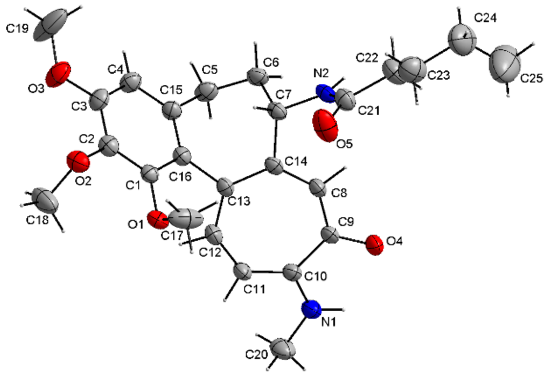

6 (RT)

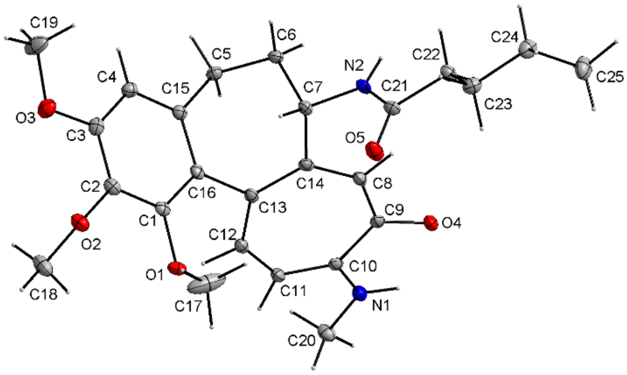

6 (100 K)

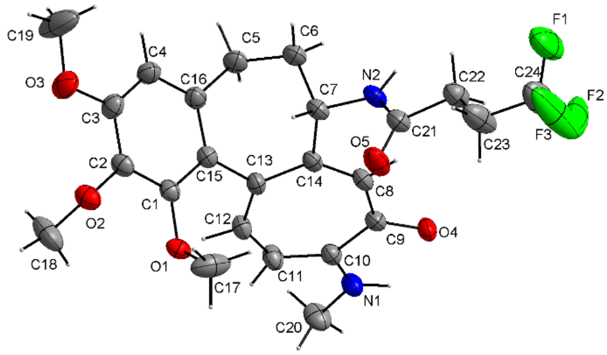

11 (RT)

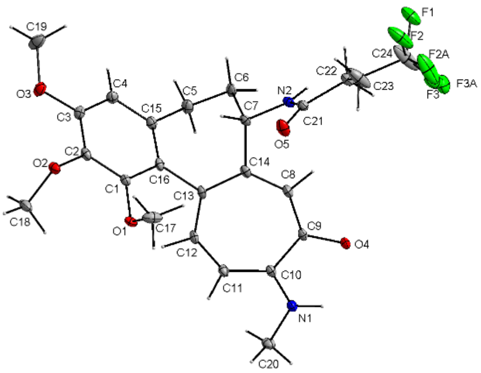

11 (100 K)

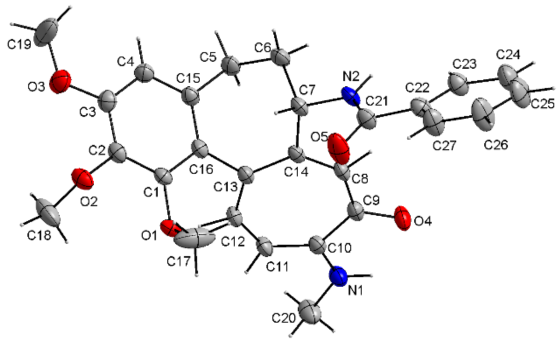

12 (RT)

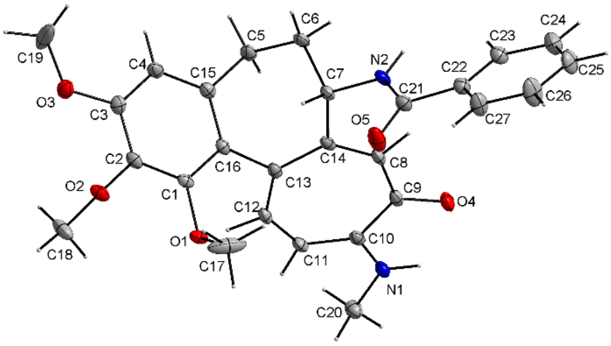

12 (100 K)

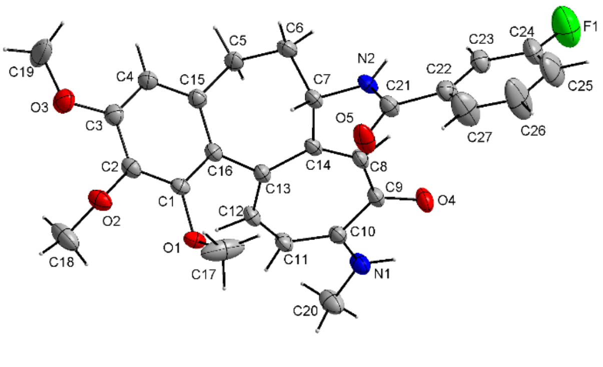

14 (RT)

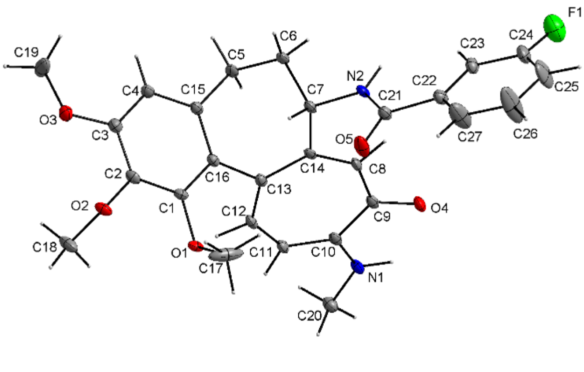

14 (100 K)

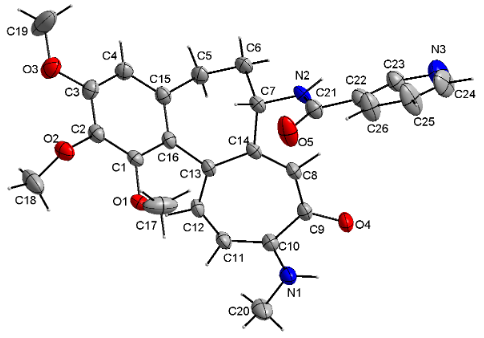

15 (RT)

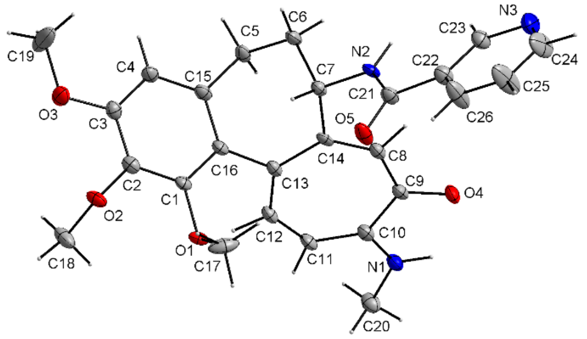

15 (100 K)

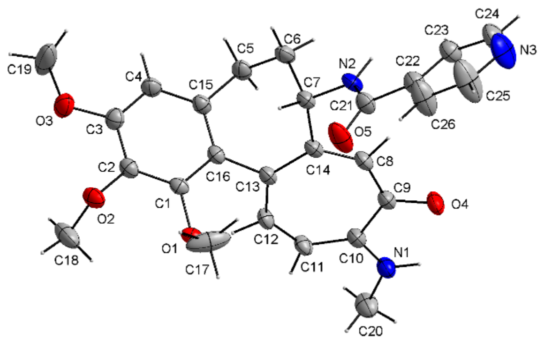

16 (RT)

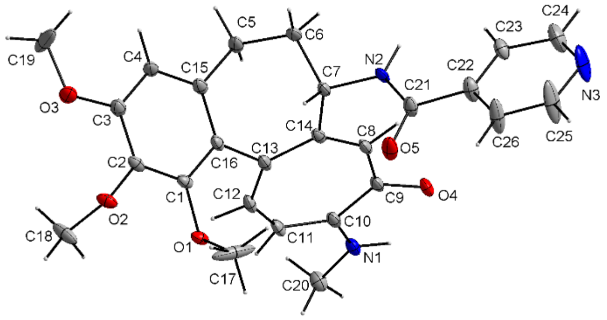

16 (100 K)

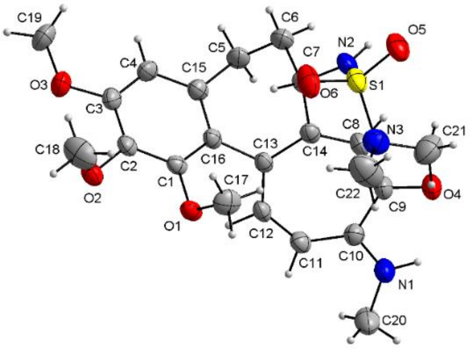

18 (RT)

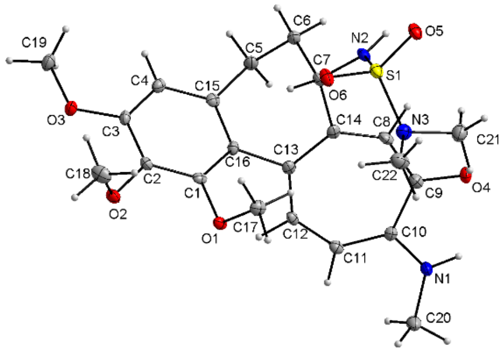

18 (100 K)

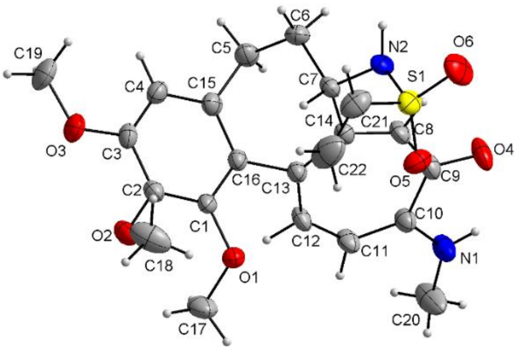

19 (RT)

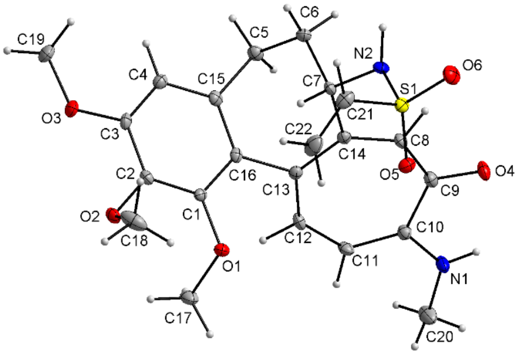

19 (100 K)

Figure S60. Molecular structures of colchicine derivatives (**6**, **11**, **12** ,**14**, **15**, **16**, **18** and **19**) at 295 K and 100 K.
